# Conditional knockout of AIM2 in microglia ameliorates synaptic plasticity and spatial memory deficits in a mouse model of Alzheimer's disease

**DOI:** 10.1111/cns.14555

**Published:** 2023-12-17

**Authors:** Lei Ye, Mengsha Hu, Rui Mao, Yi Tan, Min Sun, Junqiu Jia, Siyi Xu, Yi Liu, Xiaolei Zhu, Yun Xu, Feng Bai, Shu Shu

**Affiliations:** ^1^ Department of Neurology Nanjing Drum Tower Hospital, Affiliated Hospital of Medical School, Nanjing University Nanjing China; ^2^ Department of Neurology, Nanjing Drum Tower Hospital Clinical College of Traditional Chinese and Western Medicine Nanjing University of Chinese Medicine Nanjing China; ^3^ Jiangsu Key Laboratory for Molecular Medicine Medical School of Nanjing University Nanjing China; ^4^ Jiangsu Provincial Key Discipline of Neurology Nanjing China; ^5^ Nanjing Neurology Medical Center Nanjing China; ^6^ Nanjing Neuropsychiatry Clinic Medical Center Nanjing China

**Keywords:** AIM2, Alzheimer's disease, complement, microglia, synaptic loss

## Abstract

**Aims:**

Synaptic dysfunction is a hallmark pathology of Alzheimer's disease (AD) and is strongly associated with cognitive impairment. Abnormal phagocytosis by the microglia is one of the main causes of synapse loss in AD. Previous studies have shown that the absence of melanoma 2 (AIM2) inflammasome activity is increased in the hippocampus of APP/PS1 mice, but the role of AIM2 in AD remains unclear.

**Methods:**

Injection of Aβ_1‐42_ into the bilateral hippocampal CA1 was used to mimic an AD mouse model (AD mice). C57BL/6 mice injected with AIM2 overexpression lentivirus and conditional knockout of microglial AIM2 mice were used to confirm the function of AIM2 in AD. Cognitive functions were assessed with novel object recognition and Morris water maze tests. The protein and mRNA expression levels were evaluated by western blotting, immunofluorescence staining, and qRT‐PCR. Synaptic structure and function were detected by Golgi staining and electrophysiology.

**Results:**

The expression level of AIM2 was increased in AD mice, and overexpression of AIM2 induced synaptic and cognitive impairments in C57BL/6 mice, similar to AD mice. Elevated expression levels of AIM2 occurred in microglia in AD mice. Conditional knockout of microglial AIM2 rescued cognitive and synaptic dysfunction in AD mice. Excessive microglial phagocytosis activity of synapses was decreased after knockout of microglial AIM2, which was associated with inhibiting complement activation.

**Conclusion:**

Our results demonstrated that microglial AIM2 plays a critical role in regulating synaptic plasticity and memory deficits associated with AD, providing a new direction for developing novel preventative and therapeutic interventions for this disease.

## Graphical abstract

Synaptic dysfunction is a hallmark pathology of Alzheimer's disease (AD) and is strongly associated with cognitive impairment. Abnormal phagocytosis by the microglia is one of the main causes of synapse loss in AD. Microglial AIM2 plays a critical role in regulating synaptic plasticity and memory deficits associated with AD, providing a new direction for developing novel preventative and therapeutic interventions for this disease.

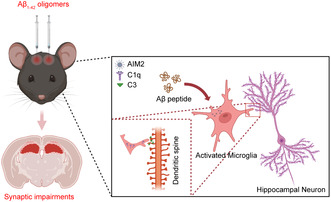

## 1 INTRODUCTION

Alzheimer's disease (AD) is the most common neurodegenerative disorder, is characterized by progressive cognitive decline and memory loss, which are often accompanied by emotional symptoms, and is the leading cause of dementia in elderly individuals worldwide. Extracellular amyloid‐β (Aβ) deposition and intracellular neurofibrillary tau tangles are the neuropathological hallmarks of AD.^1^ Accumulating evidence suggests that Aβ accumulates in toxic forms and damages neural connections, resulting in synaptic dysfunction and the loss of synapses, which are the key pathological correlates of cognitive decline in AD.^2^ Several studies used positron emission tomography (PET) and showed reductions in synaptic vesicle glycoprotein 2A in the hippocampus of APP/PS1 mice and early AD patients, suggesting reduced synaptic density.^3^, ^4^, ^5^ Moreover, neural network activity and consequent cognitive function are disrupted over time, which is attributed to Aβ‐mediated synaptic dysfunction in AD.^6^ Thus, understanding the underlying mechanisms of synaptic impairment in AD will provide novel insights into therapeutic interventions for AD.

Multiple lines of evidence indicate a central role for microglia and associated synaptic abnormalities in AD.^7^ Microglia are the resident immune cells in the central nervous system (CNS) and are responsible for the maintenance of CNS homeostasis. Under physiological conditions, microglia are involved in tissue repair, phagocytic removal of apoptotic cells and debris, and synaptic remodeling.^8^ Microglia play a distinct role in synaptic remodeling by devouring eliminated synapses and remodeling the extracellular matrix (ECM) in the normally developed brain.^9^, ^10^ Recently, evidence has indicated that microglia‐mediated synaptic pruning is involved in synapse loss in AD and correlates with cognitive deficits.^11^, ^12^, ^13^ Studies have shown that the complement system and synaptic pruning by microglia can be excessively activated, resulting in synapse loss in AD mouse models.^13^ Treatment with the metabotropic glutamate receptor 5 (mGluR5) silent allosteric modulator BMS‐984923 reversed these changes.^11^ In addition, neuronal CD47 is believed to protect synapses from excessive pruning by producing a “do not eat me” signal through interactions with its receptor signal regulatory protein α (SIRPα), and specific deletion of microglial SIRPα leads to an increase in synaptic loss mediated by microglia phagocytosis and subsequent cognitive impairment in AD mice.^12^, ^14^

Several studies have confirmed that absence in melanoma 2 (AIM2) participates in the activation of microglia.^15^, ^16^ AIM2, a PYHIN (pyrin and HIN domain‐containing protein) family member, is an essential component of the inflammasome and plays a central role in host immune responses to infections or sterile injuries. AIM2 is activated following the direct recognition of double‐stranded DNA (dsDNA) and interacts with ASC to induce the activation of caspase‐1 and the secretion of bioactive interleukin 1β (IL‐1β) and interleukin 18 (IL‐18).^17^ AIM2 has been reported to be involved in a variety of diseases of the CNS, including stroke, vascular dementia (VaD), and neurodegenerative disorders.^15^, ^18^, ^19^, ^20^, ^21^, ^22^ Our previous studies indicated that AIM2 expression was elevated in the hippocampus of APP/PS1 mice, and complete deletion of AIM2 improved synaptic plasticity and spatial memory in mice, but the detailed mechanism is still unclear.^21^

Therefore, in the present study, we investigated the specific role of AIM2 in regulating microglial function in AD‐associated learning and memory impairment and explored the underlying mechanisms. Our findings suggest that AIM2 modulates microglial phagocytosis of synapses, which is accompanied by the activation of the complement system via the classical pathway, thus leading to cognitive impairment in Aβ_1‐42_‐induced AD mice. These results reveal the precise molecular effect of AIM2 in the regulation of neural synaptic function and provide a direction for potential therapeutic targets for AD.

## 2 MATERIALS AND METHODS

### 2.1 Mouse models and treatment

C57BL/6 mice (8 weeks old), Cx3cr1Cre mice, and AIM2^fl/fl^ were all male and provided by the Model Animal Research Center of Nanjing University. AIM2^fl/fl^ mice were crossed with the Cx3cr1Cre transgenic mice to generate AIM2‐cKO mice. The construction of the AIM2 overexpression lentivirus and the control lentivirus was completed by GeneChem Corporation (Shanghai, China). For virus injection into the hippocampal region, C57BL/6 mice were anesthetized and placed in a stereotaxic frame. The coordinates were 1.82 mm posterior to the bregma, 1.13 mm lateral to the midline, and 1.25 mm below the surface of the skull. Human Aβ_1‐42_ (Millipore, Darmstadt, Germany) was prepared as previously described^23^, ^24^, ^25^, ^26^, ^27^, ^28^ and 4 μg Aβ_1‐42_ was injected into the hippocampal region of AIM2‐cKO mice and AIM2^fl/fl^ mice using a stereotaxic apparatus. Behavioral experiments and electrophysiological recordings were performed 2 weeks after injection. All experiments related to animals were approved by the Institutional Animal Care and Use Committee (IACUC) of Nanjing University.

### 2.2 Behavioral experiments

The open‐field test for the assessment of mobility and anxiety was performed as previously described.^21^ A novel object recognition (NOR) test was conducted to measure the recognition memory of mice. The Morris water maze (MWM) test was performed to evaluate the spatial learning and memory of the mice, as previously described. Details for behavioral tests are included in the Materials and Methods S1.

### 2.3 Quantitative real‐time PCR

Total RNA from tissue was extracted using a TRIzol reagent kit (Invitrogen, USA) according to the standard protocol. The cDNA was synthesized from total RNA using the PrimeScript RT Reagent kit (Takara). Quantitative PCR analysis was performed using an ABI 7500 PCR instrument (Applied Biosystems) with the SYBR Green PCR kit (Takara). The relative expression levels of each gene shown were normalized to glyceraldehyde‐3‐phosphate dehydrogenase (GAPDH). Details for primers are included in Materials and Methods S1.

### 2.4 Western blotting

Brain tissues were lysed with RIPA buffer plus protease inhibitor. The protein concentration was measured using the BCA Assay (Thermo Fisher Scientific). Tissue lysate was subjected to western blotting as previously described.^21^ Details for western blotting and antibodies are included in Materials and Methods S1.

### 2.5 Immunofluorescence staining

For sectioning, the tissues were embedded in OCT and sectioned coronally at 20 μm thickness. Immunofluorescence staining was carried out as described previously.^18^ Details for immunofluorescence staining and antibodies are included in Materials and Methods S1.

### 2.6 Electrophysiology

Acute hippocampal slices (300 μm) were prepared as described previously.^21^ Details for acute hippocampal slice preparation and electrophysiological experiments are included in the Materials and Methods S1.

### 2.7 Golgi staining and Sholl analysis

Golgi staining was performed with an FD Rapid Golgi stain kit (FD Neurotechnologies, Columbia, USA). The brains were immersed in a 1:1 mixture of solutions A and B at room temperature in the dark for 14 days and then transferred into solution C for at least 3 days. Afterward, coronal brain slices (100 μm) were sectioned with a cryostat microtome (Leica, Wetzlar, Germany) and stained according to the manufacturer's protocol. Images were acquired using Olympus IX73 and analyzed with ImageJ software (Fiji, NIH).

### 2.8 Statistical analysis

All statistical analysis results are presented as the means ± SEM, and the analysis was performed with SPSS 17.0 software (SPSS, Chicago, IL, USA). An unpaired Student's *t* test was employed to compare the two datasets, while one‐way or two‐way analysis of variance (ANOVA) with the Bonferroni post hoc test was used for comparisons between more than two groups. Statistical significance was assumed when the *p* < 0.05.

## 3 RESULTS

### 3.1 Synaptic damage and increased AIM2 expression in Aβ_1‐42_‐induced AD mice

**FIGURE 1:**
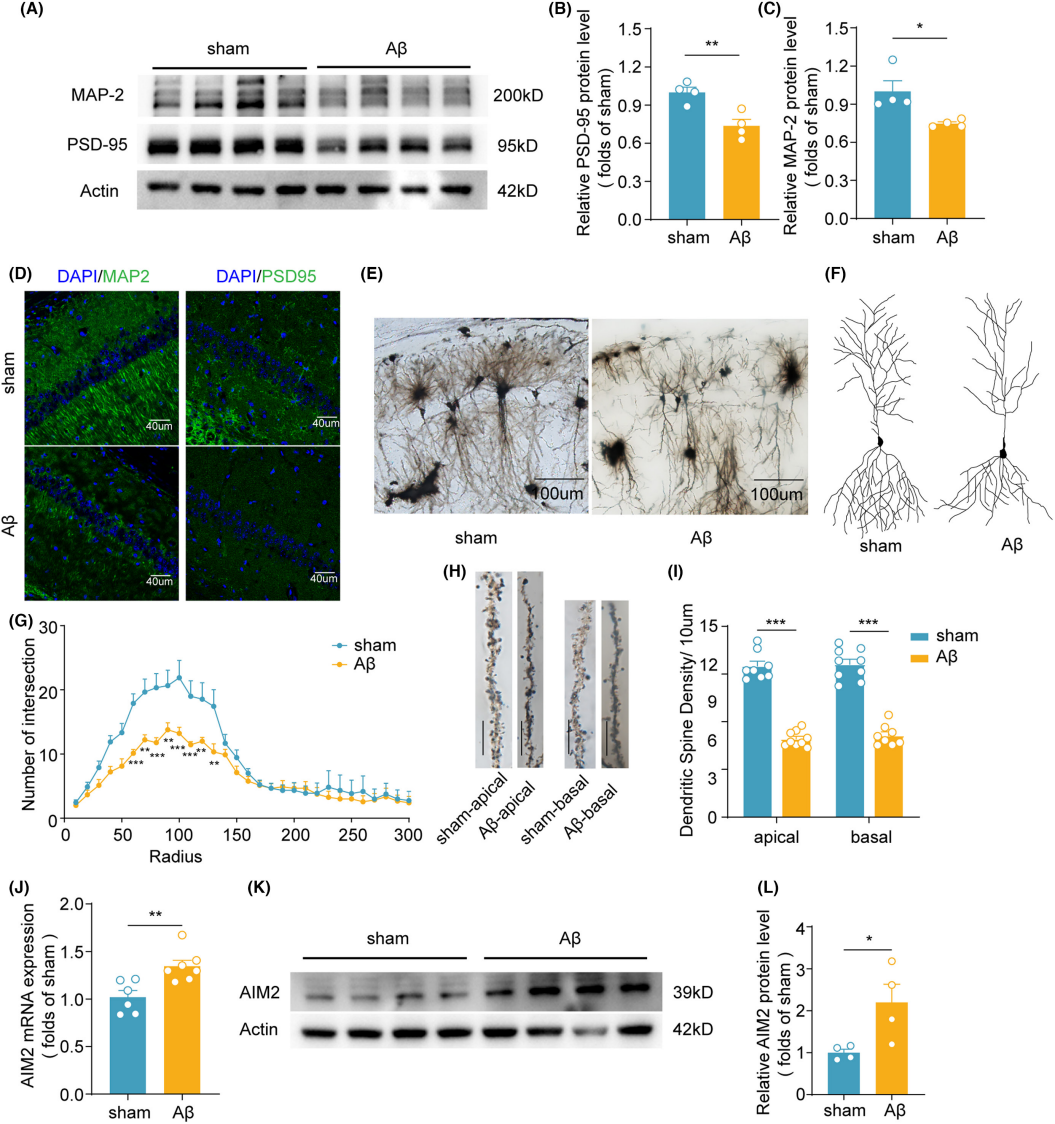
Synaptic damage and increased AIM2 expression in the microglia of Aβ_1‐42_‐induced AD mice. (A–C) The protein levels of PSD95 and MAP2 were assessed by western blotting and normalized to β‐actin as a loading control. *n* = 4 for each group. *t* (6) = 4.006, *p* = 0.0071 for PSD‐95; *p* = 0.0286 for MAP‐2. (D) Immunostaining for PSD‐95 and MAP‐2 in the hippocampal region of sham and AD mice. (E) Overview of hippocampal CA1 neurons at low magnification. (F) Representative reconstructions of the morphology of hippocampal CA1 neurons. (G) The number of intersections quantified by Sholl analysis of neurons in sham (*n* = 9 neurons, 3 mice) and AD mice (*n* = 9 neurons, 3 mice). *F*(1, 16) = 19.02, *p* = 0.0005. (H) Typical Golgi‐stained apical and basal dendrites from hippocampal CA1 neurons. Bar = 10 μm. (I) Quantitative analysis of dendritic spine density in sham (*n* = 8–9 spines, 3 mice) and AD mice (*n* = 8–9 spines, 3 mice). *t*(15) = 13.44, *p* < 0.0001 for apical spines; *t*(15) = 11.03, *p* < 0.0001 for basal spines. (J) The level of AIM2 was measured by qPCR and normalized to GAPDH mRNA. *n* = 6–7 for each group. *t* (11) = 3.507, *p* = 0.0049. (K,L) The expression level of AIM2 was verified by western blotting, and the corresponding quantified results were obtained with β‐actin as a loading control. *n* = 4 for each group. *t* (6) = 2.692, *p* = 0.0359. (M) Representative confocal images showing the colocalization of IBA1 (red) and AIM2 (green) immunofluorescence in the hippocampus of Aβ_1‐42_‐induced AD mice and sham mice. The data are shown as the mean ± SEM. Shapiro–Wilk test for (B), (C), (G), (I), (J) and (L), *w* = 0.9504, *p* = 0.7188 for sham in (B); *w* = 0.9799, *p* = 0.9015 for Aβ in (B); *w* = 0.7062, *p* = 0.0137 for sham in (C); *w* = 0.8752, *p* = 0.3185 for Aβ in (C); *w* = 0.8439, *p* = 0.0825 for apical spines of sham group in (I); *w* = 0.9222, *p* = 0.4109 for apical spines of Aβ group in (I); *w* = 0.9466, *p* = 0.6526 for basal spines of sham group in (I); *w* = 0.8854, *p* = 0.2121 for basal spines of Aβ group in (I); *w* = 0.8644, *p* = 0.2048 for sham in (J); *w* = 0.8684, *p* = 0.1797 for Aβ in (J); *w* = 0.8723, *p* = 0.3067 for sham in (L); *w* = 0.9762, *p* = 0.8795 for Aβ in (L). Unpaired two‐tailed *t* test for (B), (I), (J) and (L). Mann–Whitney test for (C). Two‐way ANOVA followed by Bonferroni post hoc correction for G. *p < 0.05, **p < 0.01, ***p < 0.001.

In this study, we used an Aβ_1‐42_‐induced AD mouse model (AD mice)^23^, ^24^, ^25^, ^26^, ^27^, ^28^ and performed behavioral experiments. In open‐field tests, the results revealed that AD mice did not display significant differences in locomotor activity and anxiety‐related behaviors compared to sham mice (Figure S1A–D). During the recognition phase of the NOR test, AD mice spent significantly less time exploring the novel object than sham mice (Figure S1E,F). During the training trial of the MWM test, the escape latency was increased in AD mice compared with that in sham mice (Figure S1G). There were no differences between AD mice and sham mice in swimming speed and latency to reach the target quadrant (Figure S1H,L), while AD mice exhibited increased latency to reach the hidden platform, as well as decreased platform crossings and time in the target quadrant (Figure S1I–K,M). Given the links between synaptic plasticity in the hippocampus and AD, we evaluated the effects of Aβ_1‐42_ treatment on synaptic structures in the hippocampus. The levels of the synaptic markers PSD‐95 and MAP‐2 were significantly decreased in AD mice compared with sham mice (Figure 1A–D). In addition, treatment with Aβ_1‐42_ led to a significant decrease in dendritic complexity (Figure 1E–G) and spine density (Figure 1H,I) by Golgi staining.

Our previous study demonstrated that AIM2 expression was upregulated in the hippocampus in 6‐month‐old APP/PS1 mice compared to wild‐type littermates.^21^ Consistent with our prior study, the qPCR (Figure 1J) and WB (Figure 1K,L) data showed that the level of AIM2 was significantly increased in the hippocampus of AD mice compared with the sham group. These results indicated cognitive and synaptic impairment and increased expression of AIM2 in AD mice.

### 3.2 Overexpression of AIM2 contributed to disruption of synaptic structure and function

**FIGURE 2:**
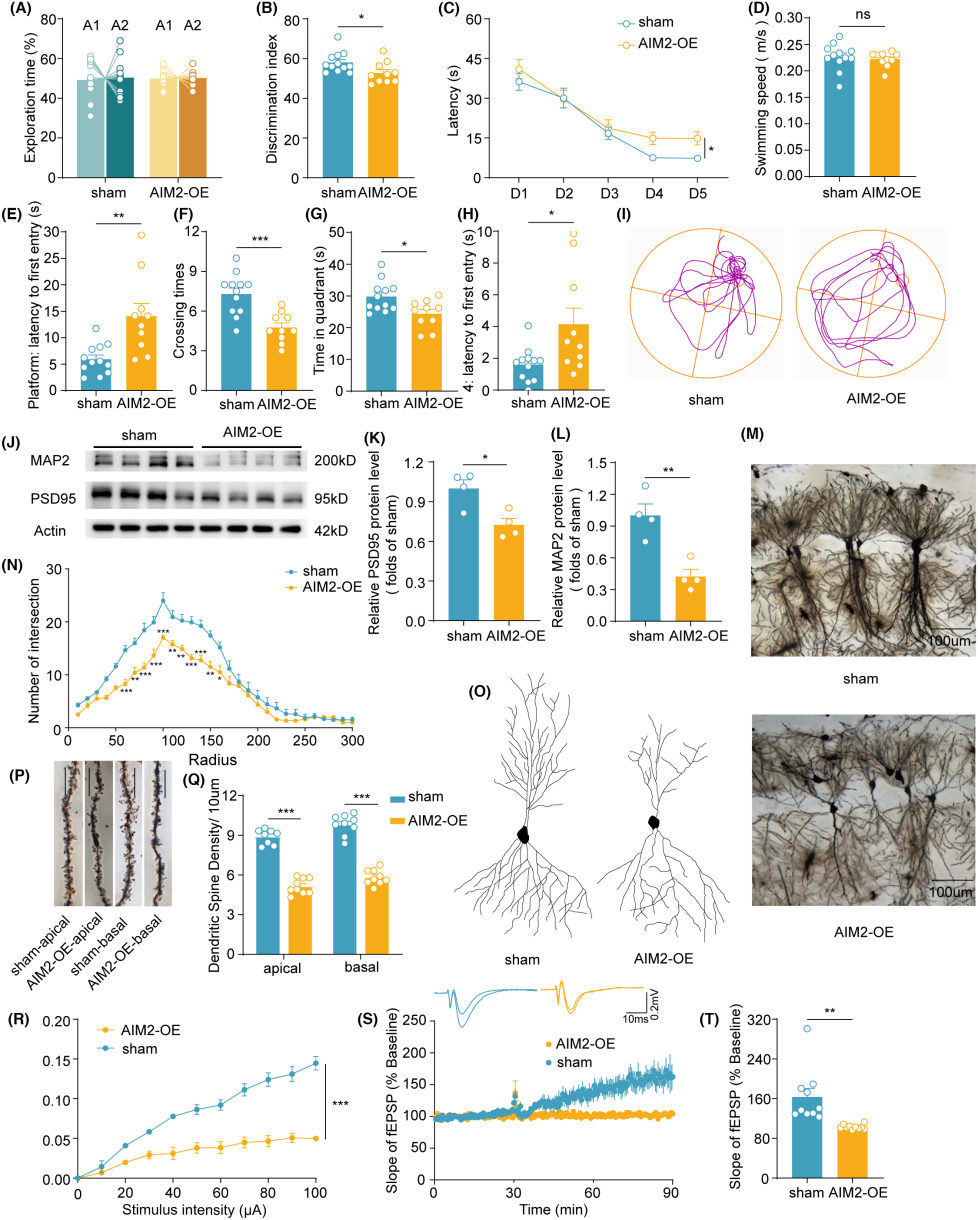
Overexpression of AIM2 contributed to disrupted synaptic structure and function. (A, B) The ratio of time spent exploring the same object (A) and the novel object (B) was measured in NOR tests. *n* = 10–12 for each group. *t*(20) = 2.366, *p* = 0.0282. (C) The escape latency in the training session of the MWM test was analyzed. *n* = 10–12 for each group. *F*(1, 20) = 4.483, *p* = 0.0470. (D–H) In the probe session, the swimming speed (D), the escape latency to reach the platform (E), the number of platform crossings (F), time in the target quadrant (G), and the latency to find the target quadrant (H) were recorded. *n* = 10–12 for each group. *t*(20) = 0.4869, *p* = 0.6316 for swimming speed; *t*(20) = 3.515, *p* = 0.0022 for latency to platform; *t*(20) = 4.315, *p* = 0.0003 for the number of platform crossings; *t*(20) = 2.758, *p* = 0.0121 for time in target quadrant; *p* = 0.0286 for latency to target quadrant. (I) Representative movement tracks of each group during the probe phase. (J–L) The protein levels of PSD95 and MAP2 were assessed by western blotting and normalized to β‐actin as a loading control. *n* = 4 for each group. *t* (6) = 3.389, *p* = 0.0147 for PSD‐95; *t*(6) = 4.516, *p* = 0.0040 for MAP‐2. (M) Overview of hippocampal CA1 neurons at low magnification. (N) Representative reconstructions of the morphology of hippocampal CA1 neurons. (O) The number of intersections quantified by Sholl analysis of neurons in sham (*n* = 12 neurons, 4 mice) and AIM2‐OE mice (*n* = 8 neurons, 3 mice). *F*(1, 18) = 21.51, *p* = 0.0002. (P) Typical Golgi‐stained apical and basal dendrites from hippocampal CA1 neurons. Bar = 10 μm. (Q) Quantitative analysis of dendritic spine density in sham (*n* = 8–9 spines, 4 mice) and AIM2‐OE mice (*n* = 9 spines, 3 mice). *t*(15) = 13.36, *p* < 0.0001 for apical spines; *t*(16) = 12.65, *p* < 0.0001 for basal spines. (R) The input–output relationship (I‐O curve) in the hippocampal CA1 region of sham (*n* = 7 slices, 4 mice) and AIM2‐OE (*n* = 7 slices, 4 mice) mice. *F*(1, 12) = 33.62, *p* < 0.0001. (S) Representative traces of fEPSCs recorded during baseline and 60 min post‐HFS in the hippocampal CA1 of sham and AIM2‐OE mice. (T) The average normalized fEPSP slope in the last 5 min of LTP recordings in sham (*n* = 10 slices, 4 mice) and AIM2‐OE mice (*n* = 10 slices, 4 mice). *p* < 0.0001. Data are shown as the mean ± SEM. Shapiro–Wilk test for (B), (D–H), (K), (L), (Q) and (T), *w* = 0.8670, *p* = 0.0598 for sham in B; *w* = 0.9003, *p* = 0.2207 for AIM2‐OE in (B); *w* = 0.9327, *p* = 0.4095 for sham in (D); *w* = 0.8685, *p* = 0.0960 for AIM2‐OE in (D); *w* = 0.9519, *p* = 0.6652 for sham in (E); *w* = 0.8950, *p* = 0.1931 for AIM2‐OE in (E); *w* = 0.9614, *p* = 0.8038 for sham in (F); *w* = 0.9639, *p* = 0.8298 for AIM2‐OE in (F); *w* = 0.9057, *p* = 0.1880 for sham in (G); *w* = 0.9209, *p* = 0.3646 for AIM2‐OE in G; *w* = 0.9224, *p* = 0.3064 for sham in (H); *w* = 0.8274, *p* = 0.0311 for AIM2‐OE in (H); *w* = 0.8257, *p* = 0.1569 for sham in (K); *w* = 0.9357, *p* = 0.6282 for AIM2‐OE in (K); *w* = 0.9695, *p* = 0.8382 for sham in (L); *w* = 0.8713, *p* = 0.3027 for AIM2‐OE in (L); *w* = 0.8814, *p* = 0.1940 for apical spines of sham group in (Q); *w* = 0.9086, *p* = 0.3064 for apical spines of AIM2‐OE group in (Q); *w* = 0.9251, *p* = 0.4364 for basal spines of sham group in (Q); *w* = 0.9658, *p* = 0.8566 for basal spines of AIM2‐OE group in (Q); *w* = 0.7259, *p* = 0.0018 for sham in (T); *w* = 0.9135, *p* = 0.3057 for AIM2‐OE in (T). Unpaired two‐tailed *t* test for (B), (D–G), (K), (L) and (Q). Mann–Whitney test for (H) and (T). Two‐way ANOVA followed by Bonferroni post hoc correction for N and R. *p < 0.05, **p < 0.01, ***p < 0.001; ns, no significance.

Next, we overexpressed AIM2 in the hippocampus of 8‐week‐old C57BL/6 mice to explore the impact of AIM2 on the structural and functional properties of synapses in AD pathogenesis. Viral infection efficiency was verified by GFP fluorescence intensity (Figure S2A). On the other hand, both qPCR (Figure S2B) and WB (Figure S2C,D) data indicated that the level of AIM2 was significantly increased in the hippocampus. To probe the effect of AIM2 on cognitive function, open field, Y maze, new object recognition (NOR), and MWM tests were carried out 1 week after viral injection. No differences in mean speed or time spent in the center and corner zones were found between AIM2‐OE mice and sham mice, suggesting that AIM2 overexpression did not affect general motor function or anxiety‐like behavior (Figure S2E–H). In addition, we found that preference for the novel object was damaged in AIM2‐OE mice during the testing phase of the NOR test (Figure 2A,B). The MWM test was performed to determine the spatial learning capacity. The escape latency of AIM2‐OE mice was significantly prolonged during the training trial (Figure 2C). During the probe test, the swimming speed was comparable between groups (Figure 2D), while the latency to reach the platform and target quadrant were significantly increased, and the number of platform crossings and time spent in the target quadrant declined clearly with the overexpression of AIM2 (Figure 2E–I). It was thus evident that overexpression of AIM2 in the hippocampus accentuated learning and memory impairment.

Given that synaptic plasticity is considered the basis of learning and memory functions, we next determined whether overexpression of AIM2 affected the synaptic structure and function of hippocampal neurons. The overexpression of AIM2 led to a significant decrease in the levels of PSD95 and MAP2 by western blot analysis (Figure 2J–L). Furthermore, we performed Golgi staining and found that neuronal complexity (Figure 2M–O) as well as apical and basal dendritic spine density (Figure 2P,Q) decreased in hippocampal CA1 neurons of AIM2‐OE mice using Sholl analysis. We carried out electrophysiological analysis of the hippocampal CA1 region in AIM2‐OE mice and sham mice. With increasing stimulus intensity, the fEPSP slope of hippocampal slices from AIM2‐OE mice was significantly reduced (Figure 2R). Additionally, we observed a significant reduction in LTP magnitudes in AIM2‐OE mice compared to sham mice after high‐frequency trains (Figure 2S,T). Altogether, these data strongly suggest that AIM2 overexpression in the hippocampus disrupted basal synaptic transmission and synaptic plasticity.

### 3.3 AIM2 deficiency in microglia rescued cognitive impairment in the Aβ_1‐42_‐induced AD model

**FIGURE 3:**
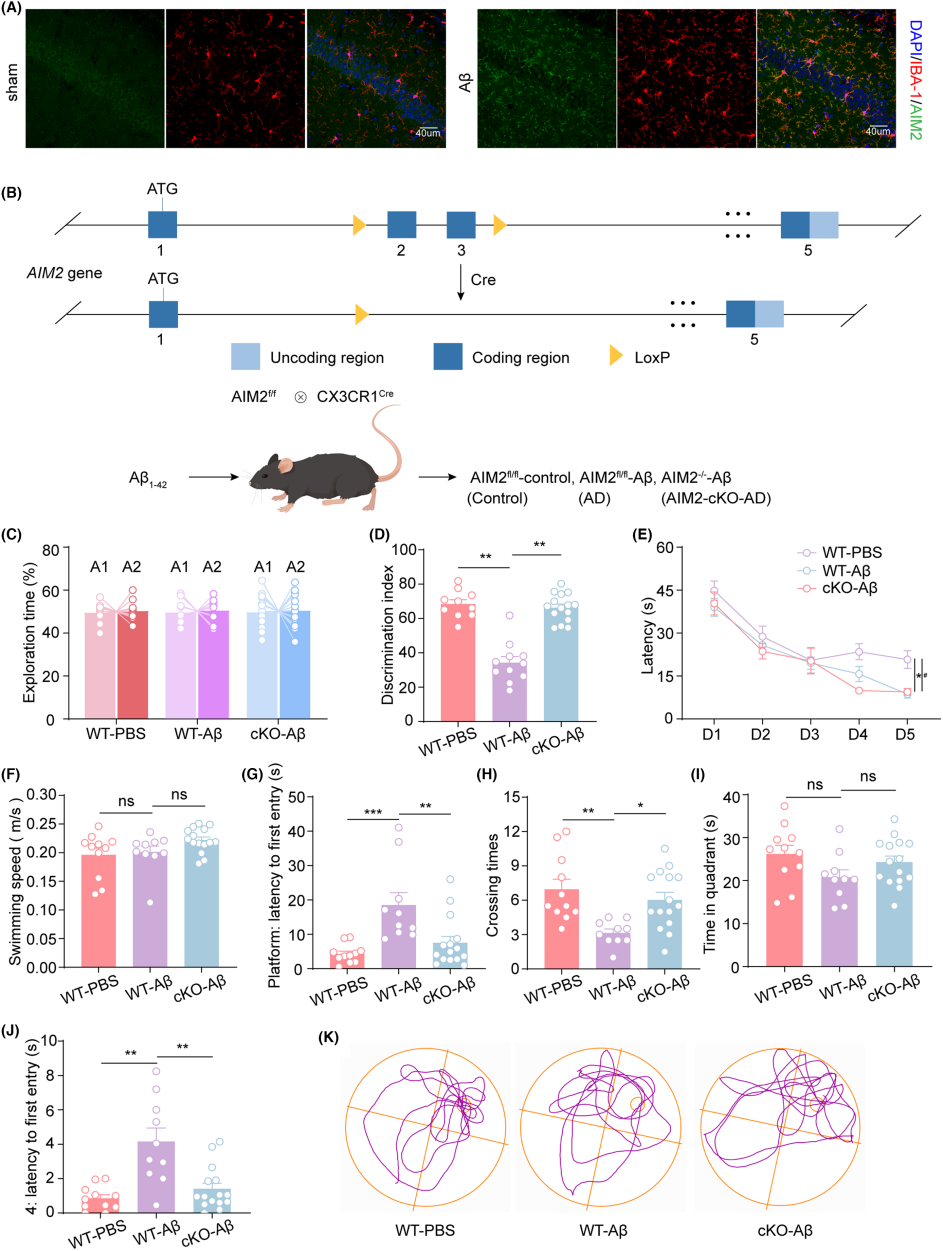
AIM2 deficiency in microglia rescued cognitive impairment in the Aβ_1‐42_‐induced AD model. (A) Immunostaining for IBA‐1 (red) and AIM2 (green) in the hippocampal region in Aβ_1‐42_‐induced AD mice and sham mice. (B) Schematic diagram for generating the microglial AIM2 conditional knockout mice. (C, D) The percentage of time spent exploring the same object (C) and a novel object (D) was measured in the NOR tests. *n* = 10–15 for each group. *F*(2,33) = 0.1612, AD versus sham group: *p* = 0.0022, AIM2‐cKO‐AD versus AD group: *p* = 0.0053. (E) The escape latency during the training period in MWM tests was detected. *n* = 10–15 for each group. *F*(2, 33) = 4.032, AD versus sham group: *p* = 0.0268, AIM2‐cKO‐AD versus AD group: *p* = 0.0459. (F–J) The swimming speed (F), latency to reach the platform (G), number of platform crossings (H), time spent in the target quadrant (I), and latency to reach the target quadrant (J) were evaluated during the probe trial in MWM tests. *n* = 10–15 for each group. AD versus Sham group: *p* > 0.9999, AIM2‐cKO‐AD versus AD group: *p* = 0.1257 for swimming speed; *F*(2,33) = 2.329, AD versus Sham group: *p* = 0.0004, AIM2‐cKO‐AD versus AD group: *p* = 0.0040 for latency to platform; *F*(2,33) = 2.172, AD versus Sham group: *p* = 0.0023, AIM2‐cKO‐AD versus AD group: *p* = 0.0142 for the number of platform crossings; *F*(2,33) = 0.2824, AD versus Sham group: *p* = 0.1053, AIM2‐cKO‐AD versus AD group: *p* = 0.3232 for time in target quadrant; AD versus Sham group: *p* = 0.0011, AIM2‐cKO‐AD versus AD group: *p* = 0.0100 for latency to target quadrant. (K) Representative locomotor traces in the MWM tests. The data are shown as the mean ± SEM. Shapiro–Wilk test for (D) and (F–J). *w* = 0.9858, *p* = 0.9887 for sham in (D), *w* = 0.8957, *p* = 0.1636 for AD in (D), *w* = 0.9536, *p* = 0.5821 for AIM2‐cKO‐AD in (D); *w* = 0.8568, *p* = 0.0524 for sham in (F), *w* = 0.7361, *p* = 0.0024 for AD in (F), *w* = 0.9383, *p* = 0.3612 for AIM2‐cKO‐AD in (F); *w* = 0.9102, *p* = 0.2449 for sham in (G), *w* = 0.7866, *p* = 0.0100 for AD in (G), *w* = 0.7901, *p* = 0.0028 for AIM2‐cKO‐AD in (G); *w* = 0.8828, *p* = 0.1129 for sham in (H), *w* = 0.9310, *p* = 0.4578 for AD in (H), *w* = 0.9649, *p* = 0.7769 for AIM2‐cKO‐AD in (H); *w* = 0.9638, *p* = 0.8178 for sham in (I), *w* = 0.9292, *p* = 0.4405 for AD in (I), *w* = 0.9680, *p* = 0.8276 for AIM2‐cKO‐AD in (I); *w* = 0.9197, *p* = 0.3165 for sham in (J), *w* = 0.9678, *p* = 0.8697 for AD in (J), *w* = 0.8441, *p* = 0.0144 for AIM2‐cKO‐AD in (J); One‐way ANOVA followed by Dunnett's post hoc test for (C), (E), (G), (H), and (I). One‐way ANOVA followed by Dunn's Test for (F), (G) and (J). Two‐way ANOVA followed by Bonferroni post hoc correction for D. *p < 0.05, **p < 0.01, ***p < 0.001; ns no significance.

To confirm that high expression of AIM2 mainly occurs in microglia in AD, we performed immunofluorescence in AD mice, and the results revealed elevated AIM2 staining predominantly in microglia (Figure 3A and Figure S3). Furthermore, to investigate the role of AIM2 in microglia in AD, we constructed microglial AIM2 conditional knockout mice (AIM2‐cKO mice; Figure 3B and Figure S4). We injected Aβ_1‐42_ into AIM2‐cKO mice to induce the AD model (AIM2‐cKO‐AD mice) and then performed open field, Y maze, NOR and MWM tests to explore the cognitive function of AIM2‐cKO‐AD mice. In the open field test, the movement speed and time spent in the corner or center were not significantly different, which suggested similar motor abilities and no anxiety‐related behaviors in each group of mice (Figure S5). During the recognition phase of the NOR test, AIM2‐cKO‐AD mice spent significantly more time exploring the novel object than AD mice (Figure 3C,D). The escape latency of the MWM test in the training sessions was increased in AD mice compared with sham mice, whereas it was significantly reduced after microglia‐specific AIM2 deletion (Figure 3E). No differences were observed in each group regarding swimming speed or time in the target quadrant, while AIM2‐cKO‐AD mice exhibited increased numbers of platform crossings and decreased latency to reach the hidden platform and the target quadrant during the probe trial test compared with AD mice (Figure 3F–K). The above results suggested that AIM2 deficiency in microglia attenuated the impairment in learning and memory in AD mice.

### 3.4 AIM2 deficiency in microglia ameliorated synaptic dysfunction

**FIGURE 4:**
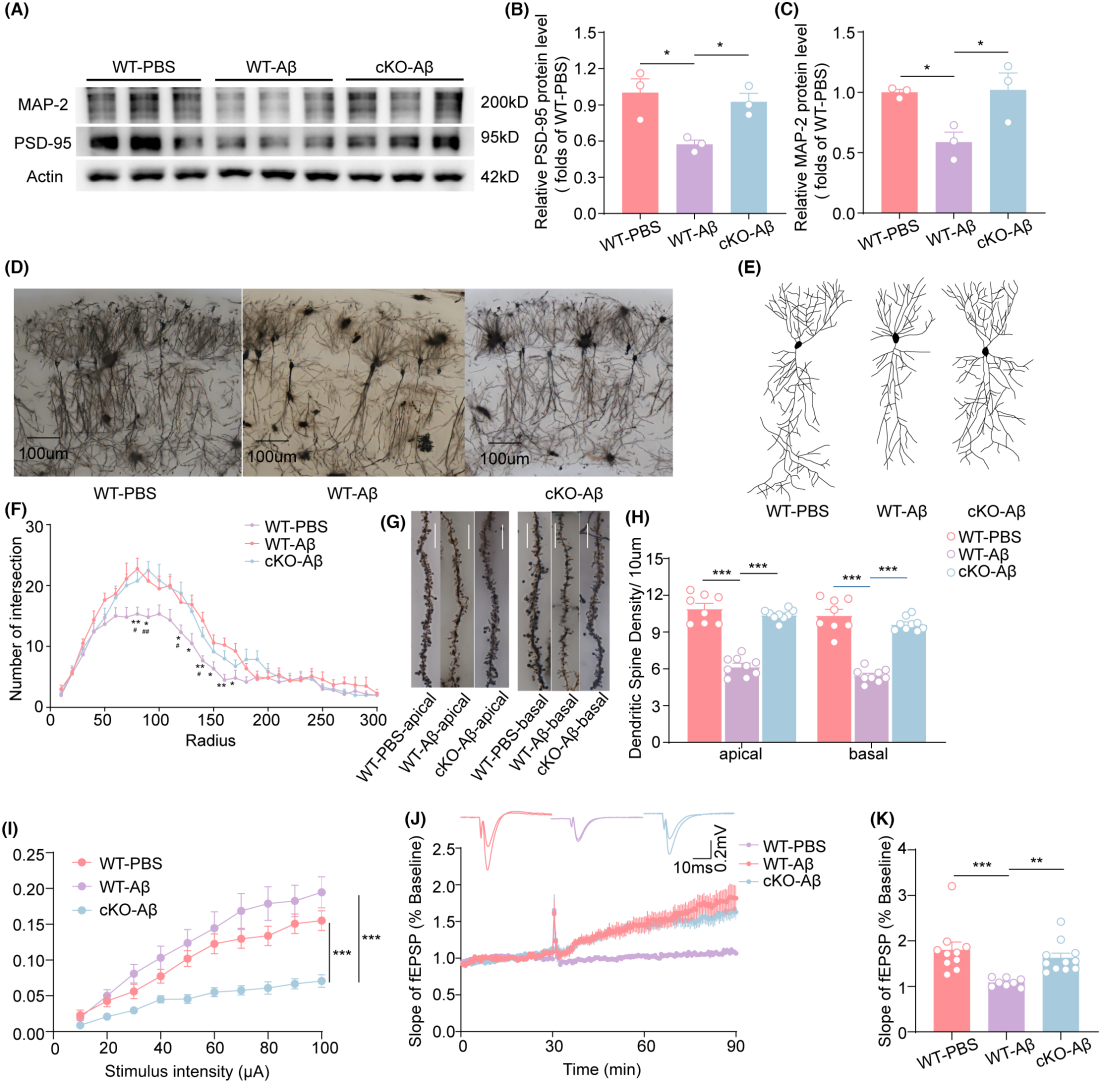
Knockout of microglial AIM2 ameliorated synaptic dysfunction. (A) The protein expression of PSD95 and MAP2 was determined by western blotting. (B, C) Quantitative analysis of PSD95 (B) and MAP2 (C) protein levels normalized to β‐actin. *n* = 6 for each group. *F*(2, 6) = 0.6151, AD versus sham group: *p* = 0.0169, AIM2‐cKO‐AD versus AD group: *p* = 0.0374 for PSD95; *F*(2, 6) = 0.9634, AD versus sham group: *p* = 0.0380, AIM2‐cKO‐AD versus AD group: *p* = 0.0313 for MAP2. (D) Representative Golgi staining showing an overview of hippocampal CA1 neurons. (E) Representative traces of CA1 pyramidal neurons in sham, AD and AIM2‐cKO‐AD mice. (F) The number of Golgi‐stained dendritic intersections was counted in sham (*n* = 8 neurons, 3 mice), AD (*n* = 6 neurons, 3 mice), and AIM2‐cKO‐AD (*n* = 8 neurons, 3 mice) mice. *F*(2, 19) = 10.04, AD versus sham group: *p* < 0.0001, AIM2‐cKO‐AD versus AD group: *p* < 0.0001. (G) Representative images of Golgi‐stained apical and basal dendritic spines of CA1 pyramidal neurons in sham, AD, and AIM2‐cKO‐AD mice. Bar = 10 μm. (H) Quantification of dendritic spine density in CA1 pyramidal neurons from sham (*n* = 8 spines, 3 mice), AD (*n* = 9 spines, 3 mice), and AIM2‐cKO‐AD mice (*n* = 9 spines, 3 mice). *F*(2, 23) = 8.590, AD versus sham group: *p* < 0.0001, AIM2‐cKO‐AD versus AD group: *p* < 0.0001 for apical spines; *F*(2, 23) = 7.726, AD versus sham group: *p* < 0.0001, AIM2‐cKO‐AD versus AD group: *p* < 0.0001 for basal spines. (I) The fEPSP amplitude of hippocampal slices in sham (*n* = 9 slices, 4 mice), AD (*n* = 9 slices, 3 mice), and AIM2‐cKO‐AD mice (*n* = 9 slices, 4 mice). *F*(2, 24) = 10.30, AD versus sham group: *p* < 0.0001, AIM2‐cKO‐AD versus AD group: *p* < 0.0001. (J, K) LTP induced by high‐frequency stimulation in sham (*n* = 10 slices, 4 mice), AD (*n* = 8 slices, 3 mice), and AIM2‐cKO‐AD mice (*n* = 11 slices, 4 mice) was evaluated in hippocampal CA1. *F*(2, 26) = 1.427, AD versus sham group: *p* = 0.0010, AIM2‐cKO‐AD versus AD group: *p* = 0.0095. Data are shown as the mean ± SEM. Shapiro–Wilk test for (B), (C), (H) and (K). *w* = 0.9282, *p* = 0.4819 for sham in (B), *w* = 0.9976, *p* = 0.9056 for AD in (B), *w* = 0.9690, *p* = 0.6619 for AIM2‐cKO‐AD in (B); *w* = 0.8776, *p* = 0.3173 for sham in (C), *w* = 0.9986, *p* = 0.9277 for AD in (C), *w* = 0.9299, *p* = 0.4883 for AIM2‐cKO‐AD in (C); *w* = 0.8566, *p* = 0.1111 for apical spines of sham group in (H), *w* = 0.9626, *p* = 0.8250 for apical spines of AD group in H, *w* = 0.9299, *p* = 0.4883 for apical spines of AIM2‐cKO‐AD group in (H);*w* = 0.9163, *p* = 0.4006 for basal spines of sham group in H, *w* = 0.9721, *p* = 0.9118 for basal spines of AD group in (H), *w* = 0.8936, *p* = 0.2172 for basal spines of AIM2‐cKO‐AD group in (H); *w* = 0.7692, *p* = 0.0061 for sham in (K), *w* = 0.9348, *p* = 0.5607 for AD in (K), *w* = 0.8364, *p* = 0.0283 for AIM2‐cKO‐AD in (K). One‐way ANOVA followed by Dunnett's post hoc test for (B), (C), and (H). One‐way ANOVA followed by Dunn's Test for (K). Two‐way ANOVA followed by Bonferroni post hoc correction for (F) and (I). *p < 0.05, **p < 0.01, ***p < 0.001; #*p* < 0.05, ##*p* < 0.01, ns no significance.

Synaptic plasticity is considered the basis of learning and memory functions, and we next examined the impact of a microglia‐specific knockout of AIM2 on synaptic structure and function in the hippocampus. The WB results showed that the protein levels of PSD95 and MAP2 in AD mice were decreased compared with those in sham mice, and this effect was significantly reversed in AIM2‐cKO‐AD mice (Figure 4A–C). Furthermore, we performed Golgi staining and found that neuronal complexity (Figure 4D–F) and dendritic spine density (Figure 4G,H) in hippocampal CA1 neurons were rescued in AIM2‐cKO‐AD mice, as shown by Sholl analysis. Furthermore, we performed electrophysiological recording and analysis of the hippocampal CA1 region in each group of mice. With increasing stimulus intensity, the slope of fEPSPs increased more in AIM2‐cKO‐AD mice than in AD mice (Figure 4I). In addition, microglia‐specific AIM2 deletion led to a significant increase in LTP magnitudes induced by high‐frequency stimulation (Figure 4J,K). These results suggested that knockout of microglial AIM2 could ameliorate changes in synaptic structure and function, thereby improving memory impairment in an Aβ_1‐42_‐induced AD model.

### 3.5 AIM2 deficiency in microglia modulated microglial phagocytosis and synaptic elimination by microglia

**FIGURE 5:**
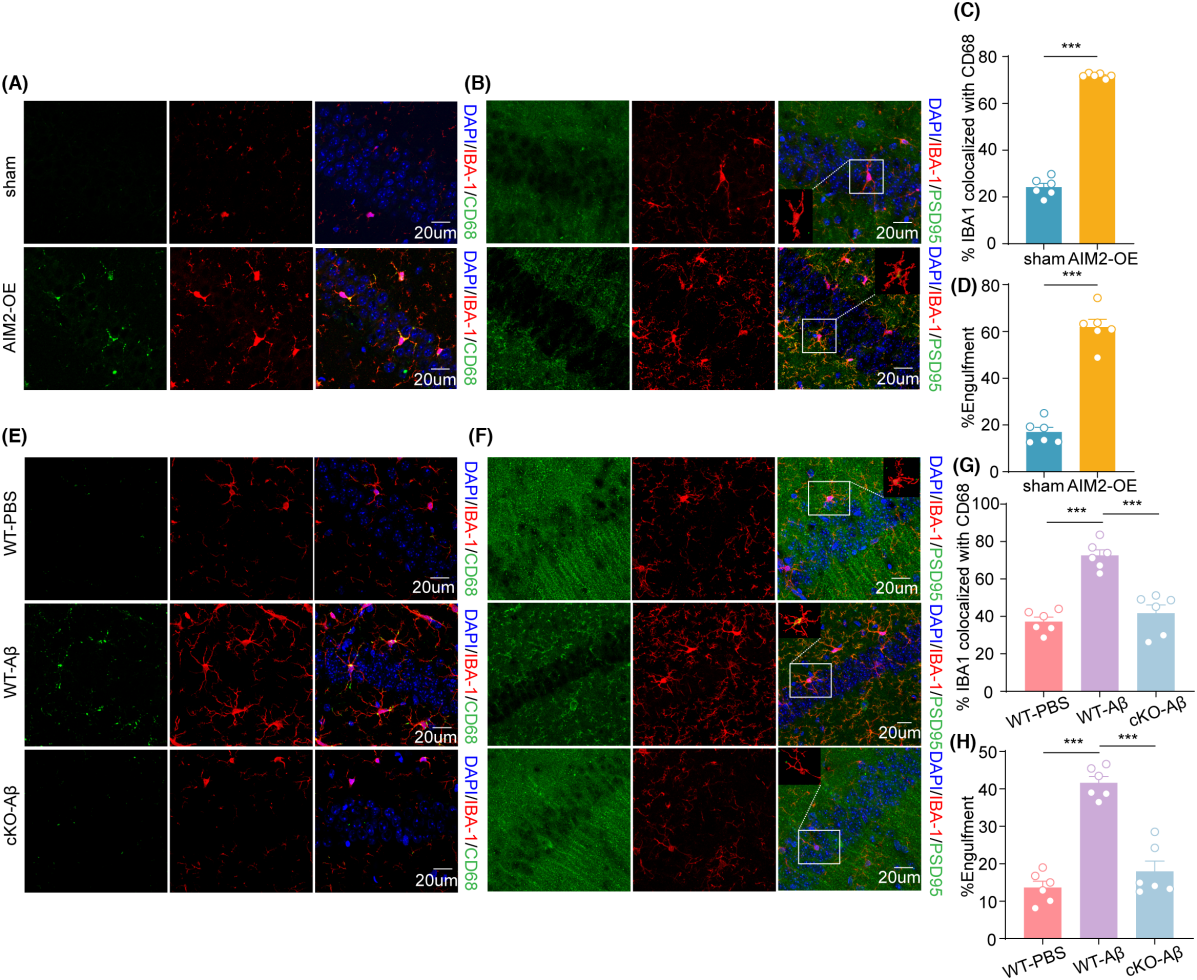
Microglia‐specific AIM2 deletion modulated microglial phagocytosis and synaptic elimination by microglia. (A) Immunostaining for IBA‐1 (red) and CD68 (green) in the hippocampal region in sham and AIM2‐OE mice. (B) Confocal images showing the presence of PSD‐95+ (green) puncta around IBA‐1+ (red) microglia and the corresponding 3D reconstructions. (C) Quantitative analysis of colocalization of IBA‐1 and CD68 in sham and AIM2‐OE mice. *n* = 6 for each group. *t* (10) = 28.45, *p* < 0.0001. (D) Quantitative analysis of phagocytic synapse in sham and AIM2‐OE mice. *n* = 6 for each group. *t* (10) = 11.52, *p* < 0.0001. (E) Immunostaining for IBA‐1 (red) and CD68 (green) in the hippocampal region in sham, AD, and AIM2‐cKO‐AD mice. (F) Confocal images showing the presence of PSD‐95+ (green) puncta around IBA‐1+ (red) microglia and the corresponding 3D reconstructions. (G) Quantitative analysis of the colocalization of IBA‐1 and CD68 in sham, AD and AIM2‐cKO‐AD mice. *n* = 6 for each group. *F*(2, 15) = 0.4746, AD versus sham group: *p* < 0.0001, AIM2‐cKO‐AD versus AD group: *p* < 0.0001. (H) Quantitative analysis of phagocytic synapses in sham, AD and AIM2‐cKO‐AD mice. *n* = 6 for each group. *F*(2, 15) = 0.2278, AD versus sham group: *p* < 0.0001, AIM2‐cKO‐AD versus AD group: *p* < 0.0001. Data are shown as the mean ± SEM. Shapiro–Wilk test for (C), (D), (G) and (H). *w* = 0.9882, *p* = 0.9844 for sham in (C), *w* = 0.9261, *p* = 0.5505 for AIM2‐OE in (C); *w* = 0.8664, *p* = 0.2123 for sham in D, *w* = 0.9246, *p* = 0.5390 for AIM2‐OE in D; *w* = 0.9395, *p* = 0.6549 for sham in (G), *w* = 0.9934, *p* = 0.9958 for AD in (G), *w* = 0.8092, *p* = 0.0710 for AIM2‐cKO‐AD in (G); *w* = 0.9763, *p* = 0.9319 for sham in (H), *w* = 0.9139, *p* = 0.4626 for AD in (H), *w* = 0.7945, *p* = 0.0524 for AIM2‐cKO‐AD in (H). Unpaired two‐tailed *t* test for C and (D). One‐way ANOVA followed by Dunnett's post hoc test for (G) and (H). ***p < 0.001.

It has been shown that microglia engage in the pruning of synapses by phagocytosis, thus affecting synaptic plasticity. We wanted to determine whether elevated expression of AIM2 could influence microglial function. Immunofluorescence staining demonstrated that CD68‐immunoreactive puncta colocalized with Iba1‐positive microglia increased appreciably in AIM2‐OE mice (Figure 5A,C). Similarly, AIM2 overexpression resulted in more PSD95 immunosignals colocalized with Iba1‐positive microglia with immunofluorescence imaging and 3D reconstruction analysis (Figure 5B,D). Consequently, these results indicated the important role of AIM2 in microglial phagocytic activity and synaptic pruning. To further evaluate the effect of conditional knockout of AIM2 on microglial function, we performed immunofluorescence staining. The results suggested an increase in Iba1+ microglia colocalized with CD68 cells in AD mice compared to sham mice, and this effect was reversed in AIM2‐cKO‐AD mice (Figure 5E,G). Furthermore, immunofluorescence and 3D reconstruction analysis revealed an increase in PSD95 immunosignals colocalized with Iba1+ microglia in AIM2^fl/fl^ mice after Aβ_1‐42_ treatment, while knockout of microglial AIM2 in the Aβ_1‐42_‐induced AD model reduced PSD95+ puncta in Iba1+ microglia (Figure 5F,H). These results confirmed that microglia‐specific knockout of AIM2 could inhibit the excessive engulfment of synapses through microglial phagocytosis.

### 3.6 AIM2 modulated microglial phagocytosis of synapse elimination via complement activation

**FIGURE 6:**
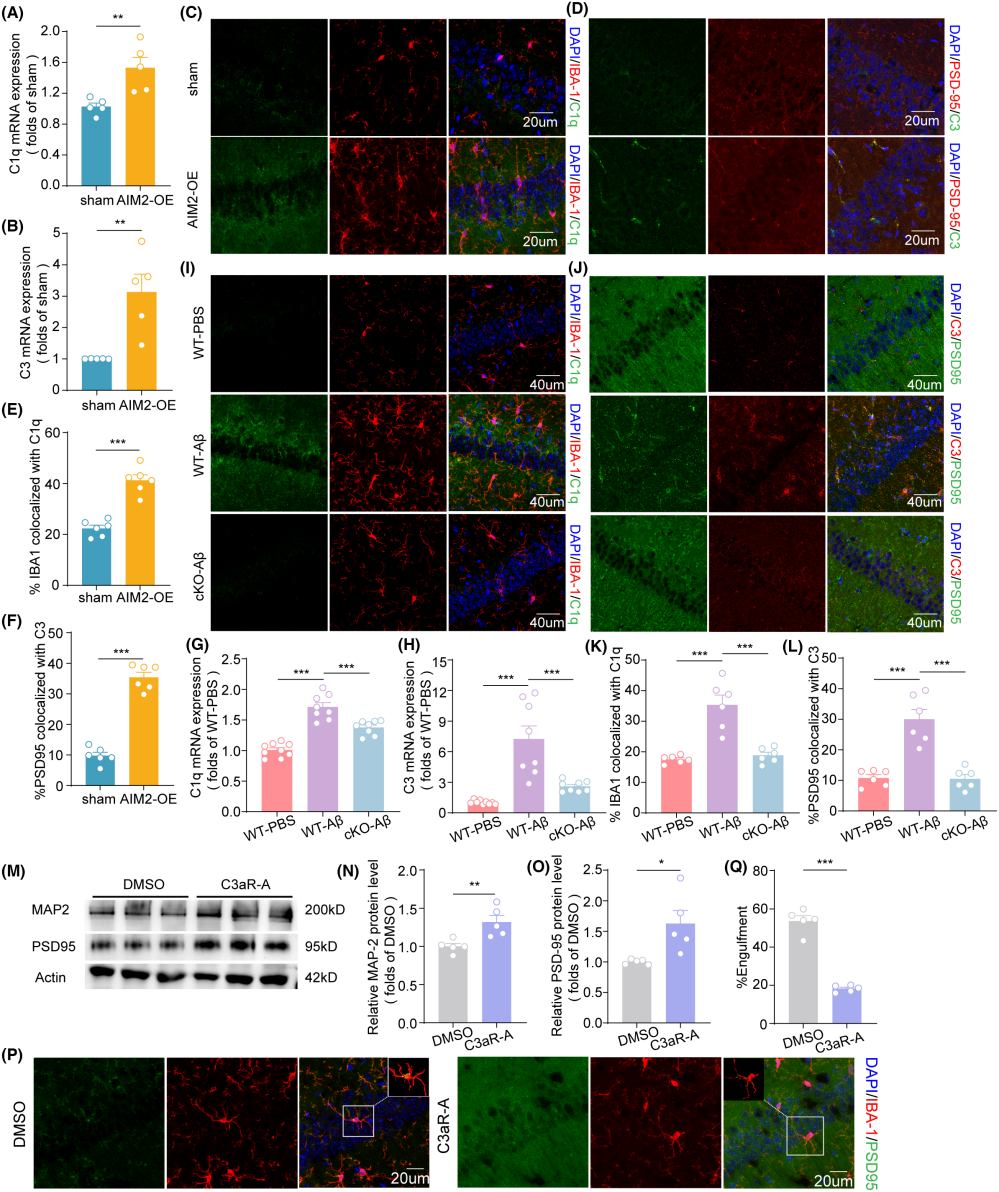
AIM2 modulated microglial phagocytosis of synapse elimination via complement activation. (A) The mRNA level of C1q in the hippocampal region in sham and AIM2‐OE mice was determined by quantitative RT–PCR. *n* = 5 for each group. *t*(8) = 3.499, *p* = 0.0081. (B) The mRNA level of C3 in the hippocampal region in sham and AIM2‐OE mice was determined by quantitative RT–PCR. *n* = 5 for each group. *t*(8) = 3.771, *p* = 0.0055. (C) The colocalization of C1q (green) with IBA‐1 (red) in the hippocampal CA1 region in sham and AIM2‐OE mice. (D) The colocalization of C3 (green) with PSD‐95 (red) in the hippocampal CA1 region in sham and AIM2‐OE mice. (E) Quantitative analysis of the colocalization of IBA‐1 and C1q in sham and AIM2‐OE mice. *n* = 6 for each group. t (10) = t = 7.575, *p* < 0.0001. (F) Quantitative analysis of the colocalization of PSD‐95 and C3 in sham and AIM2‐OE mice. *n* = 6 for each group. *t*(10) = t = 12.84, *p* < 0.0001. (G) The mRNA level of C1q in the hippocampal region in sham, AD and AIM2‐cKO‐AD mice was determined by quantitative RT–PCR. *n* = 8–9 for each group. *F*(2, 22) = 2.134, AD versus sham group: *p* < 0.0001, AIM2‐cKO‐AD versus AD group: *p* = 0.0004. (H) The mRNA level of C3 in the hippocampal region in sham, AD and AIM2‐cKO‐AD mice was determined by quantitative RT–PCR. *n* = 8–9 for each group. *F*(2, 22) = 30.36, AD versus sham group: *p* < 0.0001, AIM2‐cKO‐AD versus AD group: *p* = 0.0003. (I) The colocalization of C1q (green) with IBA‐1 (red) in the hippocampal CA1 region in sham, AD, and AIM2‐cKO‐AD mice. (J) The colocalization of C3 (green) with PSD‐95 (red) in the hippocampal CA1 region in sham, AD, and AIM2‐cKO‐AD mice. (K) Quantitative analysis of the colocalization of IBA‐1 and C1q in sham, AD and AIM2‐cKO‐AD mice. *n* = 6 for each group. *F*(2, 15) = 6.837, AD versus sham group: *p* < 0.0001, AIM2‐cKO‐AD versus AD group: *p* < 0.0001. (L) Quantitative analysis of the colocalization of PSD‐95 and C3 in sham, AD , and AIM2‐cKO‐AD mice. *n* = 6 for each group. *F*(2, 15) = 8.637, AD versus sham group: *p* < 0.0001, AIM2‐cKO‐AD versus AD group: *p* < 0.0001. (M–O) The protein levels of PSD95 and MAP2 were assessed by western blotting and normalized to β‐actin as a loading control. *n* = 5 for each group. *t*(8) = 3.382, *p* = 0.0096 for MAP‐2; *t*(8) = 2.901, *p* = 0.0199 for PSD‐95. (P) Confocal images showing the presence of PSD‐95+ (green) puncta around IBA‐1+ (red) microglia and the corresponding 3D reconstructions. (Q) Quantitative analysis of phagocytic synapses in DMSO and C3aR‐A mice. *n* = 5 for each group. *t*(8) = 12.25, *p* < 0.0001. The data are shown as the mean ± SEM. Shapiro‐Wilk test for A, B, E–H, K, L, N, O, and Q. *w* = 0.9680, *p* = 0.8625 for sham in A, *w* = 0.9221, *p* = 0.5436 for AIM2‐OE in A; *w* = 0.9378, *p* = 0.6503 for sham in B, *w* = 0.9749, *p* = 0.9059 for AIM2‐OE in B; *w* = 0.9490, *p* = 0.7321 for sham in E, *w* = 0.9760, *p* = 0.9302 for AIM2‐OE in E; *w* = 0.9629, *p* = 0.8421 for sham in F, *w* = 0.8610, *p* = 0.2702 for AIM2‐OE in F; *w* = 0.9437, *p* = 0.6217 for sham in G, *w* = 0.9583, *p* = 0.7937 for AD in G, *w* = 0.8702, *p* = 0.1514 for AIM2‐cKO‐AD in G; *w* = 0.9234, *p* = 0.4210 for sham in H, *w* = 0.8778, *p* = 0.1796 for AD in H, *w* = 0.8895, *p* = 0.2317 for AIM2‐cKO‐AD in H; *w* = 0.9832, *p* = 0.9661 for sham in K, *w* = 0.9675, *p* = 0.8749 for AD in K, *w* = 0.9714, *p* = 0.9016 for AIM2‐cKO‐AD in K; *w* = 0.8561, *p* = 0.1763 for sham in L, *w* = 0.9188, *p* = 0.4967 for AD in L, *w* = 0.9432, *p* = 0.6849 for AIM2‐cKO‐AD in L; *w* = 0.9466, *p* = 0.7132 for DMSO in N, *w* = 0.9210, *p* = 0.5363 for C3aR‐A in N; *w* = 0.9010, *p* = 0.4155 for DMSO in O, *w* = 0.9291, *p* = 0.5902 for C3aR‐A in O; *w* = 0.8943, *p* = 0.3675 for DMSO in Q, *w* = 0.8439, *p* = 0.2242 for C3aR‐A in Q; Unpaired two‐tailed *t* test for A, B, E, F, N, O, and Q. One‐way ANOVA followed by Dunnett's post hoc test for G, H, K, and L. **p < 0.01, ***p < 0.001.

The classical complement pathway may be important for microglia‐mediated synaptic pruning.^13^ Thus, we wanted to explore the role of the complement pathway in microglia‐mediated synaptic engulfment in AD pathology. We then analyzed the expression and distribution of C1q and C3 in sham and AIM2‐OE mice. The results revealed that AIM2 overexpression in the hippocampus led to increased AIM2 expression and colocalization with IBA‐1^+^ microglia (Figure 6A,C,E). Similarly, C3 expression was increased and colocalized with PSD‐95 in the hippocampus of AIM2‐OE mice (Figure 6B,D,F). In addition, our results revealed a significant increase in C1q levels (Figure 6G) and the colocalization of C1q with IBA‐1+ microglia in AD mice compared to sham mice, and deletion of microglial AIM2 reversed these effects (Figure 6I,K). Similarly, the increase in C3 expression (Figure 6H) was accompanied by increased C3 deposition on synapses in AD mice, and this deposition was significantly reduced in AIM2‐cKO‐AD mice (Figure 6J,L). Overall, these results indicated that the complement pathway was necessary for synaptic pruning by microglia.

Given the important role of C3‐C3aR signaling in synaptic function,^29^ we wanted to examine the effects of C3aR antagonists (C3aR‐A) on microglial synaptic phagocytosis and memory functions in AIM2‐OE mice (Figure S6A). The results revealed that C3aR‐A treatment did not affect the general locomotor activity or anxiety levels of AIM2‐OE mice (Figure S6B–E) but significantly increased the discrimination index in the NOR test (Figure S6F–G). The escape latency of C3aR‐A‐treated mice significantly declined during the training trial (Figure S6H). During the probe test, the swimming speed was comparable between groups, while the latency to the platform and n target quadrant was moderately decreased, and the number of platform crossings and time spent in the target quadrant increased with C3aR‐A treatment (Figure S6I–N). Overall, these data demonstrated that C3aR‐A treatment ameliorated memory impairment in AIM2‐OE mice. Similarly, we examined the effects of C3aR‐A on synaptic proteins in the hippocampus of AIM2‐OE mice. As evident from the western blot analyses, C3aR‐A treatment resulted in increased levels of PSD‐95 and MAP‐2 (Figure 6M–O). Furthermore, immunofluorescence and 3D reconstruction analysis revealed a decrease in PSD95 immunosignals colocalized with Iba1+ microglia in AIM2‐OE mice after C3aR‐A treatment (Figure 6P–Q). Taken together, these results indicated that C3aR‐A treatment improved synaptic and memory functions in AIM2‐OE mice.

## 4 DISCUSSION

Our study has uncovered a novel role for AIM2 in the pathogenesis of AD. Here, we revealed that AIM2 expression was significantly increased in the microglia of AD mice. Conditional deletion of AIM2 in microglia ameliorated cognitive impairment and synaptic deficits in an Aβ_1‐42_‐induced AD model. Moreover, microglial activation and microglial phagocytic activity toward synapses were suppressed after knockout of AIM2, which was accompanied by inhibition of the complement system via the classical pathway. Thus, our results demonstrated that AIM2 plays an important role in AD pathogenesis and may be a novel therapeutic target for AD.

Previous studies have demonstrated that AIM2 has multiple roles in the pathogenesis of several diseases, including ischemic stroke and VaD. AIM2 expression is upregulated after ischemic stimuli in a mouse middle cerebral artery occlusion (MCAO) model, while AIM2 deletion reduces cerebral infarct volume and improves motor and cognitive function.^18^, ^20^ In addition, studies have shown that AIM2 inflammasome activation can promote neuronal loss and white matter injury during cerebral hypoperfusion, leading to cognitive impairment in a mouse model of VaD.^19^ Our previous study showed the upregulation of AIM2 expression in the hippocampus in APP/PS1 mice, and AIM2 deletion markedly ameliorated changes in synaptic plasticity and memory function, but the specific mechanism remains unclear.^21^ In this study, we first demonstrated that AIM2 mediated memory deficits in AD model mice by regulating microglial phagocytosis of neuronal synapses. We injected Aβ_1‐42_ into the bilateral hippocampus of C57BL/6 mice to induce AD and observed that the increase in AIM2 expression occurred predominantly in microglia. Furthermore, the behavioral results revealed that Aβ_1‐42_ treatment impaired synaptic function and induced memory deficits, while knockout of AIM2 in microglia rescued synaptic plasticity dysfunction and cognitive dysfunction.

As resident immune cells in the brain, microglia play a complex and key role in AD pathogenesis. On the one hand, microglial activation might be beneficial for reducing Aβ aggregation in AD. On the other hand, excessive activation of microglia is responsible for the overexpression of inflammatory factors, contributing to neuronal toxicity and synaptic dysfunction.^30^, ^31^ Microglial activation‐mediated neuroinflammation plays an important role in the pathogenesis of AD. AIM2 controls microglial inflammation.^32^ In addition, AIM2 knockout reduced microglial activation in different models of neurological disease, including ischemic stroke, vascular dementia, and AD.^19^, ^33^ It has been shown that complete deletion of AIM2 mitigates microglial activation but has no beneficial effects on spatial memory in 5 × FAD mice.^16^ However, there has been no direct evidence of the role of AIM2 in regulating microglial phagocytic function. Our data revealed that conditional knockout of AIM2 in microglia could effectively suppress microglial phagocytic capacity and the phagocytosis of synapses in AD mice.

Complement cascades are involved in microglial phagocytosis and selective synaptic pruning. C1q, which is the initiating protein in the classical complement pathway, is localized at synapses, resulting in the deposition of the downstream molecule C3. Deposited C3 can activate C3 receptors, which are only expressed by activated microglia, thereby contributing to synapse phagocytosis.^34^, ^35^ It has been shown that the increased expression of C1q in AD mouse models is associated with impaired synapses and hippocampal LTP.^13^ Moreover, inhibiting C1q or C3 reduces the phagocytic ability of microglia and microglial synapse removal, rescuing synapse loss.^36^, ^37^ In our study, we showed that the expression of C1q and C3 was increased in the hippocampus, and these factors were deposited on synapses after Aβ_1‐42_ treatment, which could be reversed by microglia‐specific AIM2 deletion. Although our study identifies that AIM2 plays a critical role in regulating synaptic plasticity and memory deficits associated with AD, a limitation is that our mechanistic studies were performed primarily in mouse models. Therefore, a subsequent validation study in human postmortem tissue is warranted. In addition, longitudinal studies would be needed to better describe if the level of AIM2 protein changes in the biofluids of patients with AD, which could validate the relevance of AIM2 dysfunction to AD and additionally hold promise as diagnostic biomarkers for AD. The mechanism and progression of AIM2 dysfunction and their contributions to the complement system are poorly defined and require further investigation.

### 4.1 Limitation

Several limitations of our mouse model should be considered. The mouse model of AD induced by the Aβ_1‐42_ peptide is unable to recapitulate the complex pathology of human AD. Moreover, additional critical risk factors, including neurofibrillary tangles of tau, aging, and perturbations in the vasculature, are disregarded.^38^ In addition, the Aβ_1‐42_ peptide localizes exclusively at the site of injection, while Aβ deposition in the brains of patients with AD is heterogeneous. Further work is required to translate our findings into a clinical setting.

### 4.2 Conclusion

In summary, this study demonstrates that an increase in AIM2 expression mediates aberrant synaptic elimination via microglial activation and phagocytosis, and the complement system is activated through the classical pathway, contributing to impaired hippocampal synaptic plasticity and cognitive dysfunction in Aβ_1‐42_‐induced AD mice. These results demonstrate a key role for AIM2 in regulating microglial function in the pathogenesis of AD and suggest a potential target for the treatment of AD.

## AUTHOR CONTRIBUTIONS

Shu Shu, Feng Bai, Lei Ye, and Mengsha Hu initiated, designed the study, and wrote the manuscript. Lei Ye and Mengsha Hu conducted behavioral experiments, electrophysiological experiments, and data analysis. Lei Ye, Rui Mao, Yi Tan, Min Sun, Junqiu Jia, and Siyi Xu performed molecular biology experiments. Yi Liu, Xiaolei Zhu, and Yun Xu performed the sample collection. All authors approved the manuscript prior to submission.

## FUNDING INFORMATION

This study was funded by the National Natural Science Foundation of China (no. 82271480, 81901091) and the Natural Science Foundation of Jiangsu Province of China (BK20190124).

## CONFLICT OF INTEREST STATEMENT

Xu, Yun is an Editorial Board member of CNS Neuroscience and Therapeutics and a coauthor of this article. To minimize bias, they were excluded from all editorial decision‐making related to the acceptance of this article for publication.

## Supporting information


Data S1.

Data S2.

Data S3.

## Data Availability

The data support the findings of the current study are available from the corresponding author upon reasonable request.
