# Supplementary material for: Conditional knockout of AIM2 in microglia ameliorates synaptic plasticity and spatial memory deficits in a mouse model of Alzheimer's disease
Source: CNS Neurosci Ther. 2023 Dec 17;30(6):e14555. doi: 10.1111/cns.14555 (PMC11163192; doi:10.1111/cns.14555)
Supplement: Supplementary file 1 — Data S1. [file CNS-30-e14555-s001.docx]

**Additional file 1**

Conditional knockout of AIM2 in microglia ameliorates synaptic plasticity and spatial memory deficits in a mouse model of Alzheimer's disease

Lei Ye^1#^, Mengsha Hu^1,2#^, Rui Mao^1^, Yi Tan^1^, Min Sun^1^, Junqiu Jia^1^, Siyi Xu^1^, Yi Liu^1^, Xiaolei Zhu^1*^, Yun Xu^1, 3, 4, 5, 6^, Feng Bai^1*^, Shu Shu^1*^

**Supplementary Materials and Methods**

**Open field**

The open-field test for the assessment of mobility and anxiety was performed as previously described. Each individual mouse from the different groups was placed in a 48 cm × 48 cm ×36 cm open field box that was divided into 16 squares of equal area and recorded for 10 min. The open field area was cleaned with 75% ethanol to minimize olfactory cues. Locomotor activity measurements and time spent in the center and corner zone were quantified by ANY-maze software (Stoelting, USA).

**New object recognition (NOR)**

Novel object recognition (NOR) test was conducted to measure the recognition memory of mice in a nontransparent box measuring 30 × 30×45 cm high. Prior to testing, mice were habituated to the behavioral testing environment for 3 consecutive days (10 min per day). The mice were placed in the box containing two identical objects (A1 and A2) placed symmetrically during the 10-min training session. During the test session, one of the two identical objects (A) was replaced with a novel object (B) and the mice were allowed to freely explore the objects for 5 min. The time spent in exploring the novel object was analyzed and the discrimination index was calculated as time spent in exploring the novel object / total time spent in exploring objects during the test phase.

**Morris water maze**

The Morris water maze test was performed to evaluate spatial learning and memory of the mice as previously described. Briefly, the mice were trained to find the hidden platform submerged 1cm below the surface for 5 consecutive days. The latency in the training stage was recorded and analyzed using ANY-maze software. For probe trials, the mice were allowed to swim for 60 s freely with the platform removed. Then the swimming speed, platform crossings, the escape latency and time spent in the target quadrant were recorded.

**Quantitative real-time PCR**

Primers used were as follows:

| Gene | Primer |
| --- | --- |
| GAPDH | F: GCCAAGGCTGTGGGCAAGGT  R: TCTCCAGGCGGCACGTCAGA |
| AIM2 | F: CTCAGGAAGGAAGACAAGA  R: GATTCAACATCAACCACAAC |
| C1Q | F: CACCGTGCTTCAGCTGCGACGAG  R: TTGCGGGGTCCTTTTCGATCCAC |
| C3 | F: ACTGTGGACAACAACCTACTGC  R: GCATGTTCGTAAAAGGCTCGG |

**Western blotting**

Equal amounts of protein samples were subjected to 10% SDS-PAGE and transferred to polyvinylidene difluoride (PVDF) membrane (EMD Millipore). Membranes were blocked for 2 h at room temperature using 5% non-fat milk in Tris-Buffered Saline Tween 20 (TBST), subsequently incubated overnight at 4°C with the following primary antibodies: mouse anti-MAP 2 (1:1000, Abcam, ab11267), rabbit anti-MAP 2 (1:1000, Bioworld, BS3487), rabbit anti-PSD 95 (1:1000, Abcam, ab18258), mouse anti-PSD 95 (1:1000, Abcam, ab2723), rabbit anti β-actin (1:1000, Bioworld, AP-0060), and rabbit anti-AIM2 (1:500, Abcam, ab119791). The membranes were then incubated for 2 h at room temperature with HRP-conjugated secondary antibodies (1:5000). Bands of western blotting were visualized in a Gel-Pro system (Tanon Technologies, Shanghai, China), and protein density was analyzed and quantified using ImageJ software.

**Immunofluorescence** **staining**

Brain sections were permeabilized using PBS containing 0.25% triton X-100 (PBST) and blocked with 2% BSA at room temperature for 2 h. Subsequently, the brain sections were incubated with primary antibodies as follows at 4 °C overnight: rabbit anti IBA-1 (1:500, Abcam, ab178846); mouse anti-AIM2 (1:200, Santa Cruz Biotechnology, sc-515514); rat anti-CD68 (1:500, Abcam, ab53444); mouse anti-PSD 95 (1:1000, Abcam, ab2723); chicken anti-MAP 2 (1:1000, Abcam, ab5392); rat anti-C1q (1:500, Abcam, ab11861); rat anti-C3 (1:500, Abcam, ab11862). The sections were then incubated with secondary antibodies (Invitrogen, 1:500) at room temperature for 2 h and counterstained with DAPI (1:1000, Bioworld, Louis Park, MN, USA) for 20 min. The images were captured using a fluorescence microscope (Olympus IX73) or confocal laser-scanning microscope (Olympus FV3000) and analyzed with Image J software. Three-dimensional (3D) reconstruction was obtained using the Imaris software (Bitplane).

**Electrophysiology**

Before recordings, the slices were incubated in circulating artificial cerebrospinal fluid (ACSF) gassed with 95% O_2_ and 5% CO_2_ at room temperature for at least 2 h. Slices were then transferred into the microelectrode array continuously perfused with oxygenated ACSF (32°C) at a flow rate of 2 ml/min. Field excitatory post-synaptic potentials (fEPSPs) from the stratum radiatum of CA1 were recorded using MEA-2100-60-System (Multi Channel Systems, Reutlingen, Germany). To evaluate the input-output relationships, the slope of fEPSPs was recorded. For LTP experiments, half of the maximum evoked response was utilized as the stimulation intensity. After 30 min of stable baseline fEPSPs, the LTP was induced with high-frequency stimulation (100 Hz, three trains, 1-s duration, 10-s interval). We measured the initial slopes of fEPSP and normalized them to the average fEPSP slope during baseline period. Data acquisition was done on the LTP-Director software and data analysis with the LTP-Analyzer software.

**Supplemental Figure**

**Figure S1 Aβ_1-42_ treatment led to deficits in learning and memory**


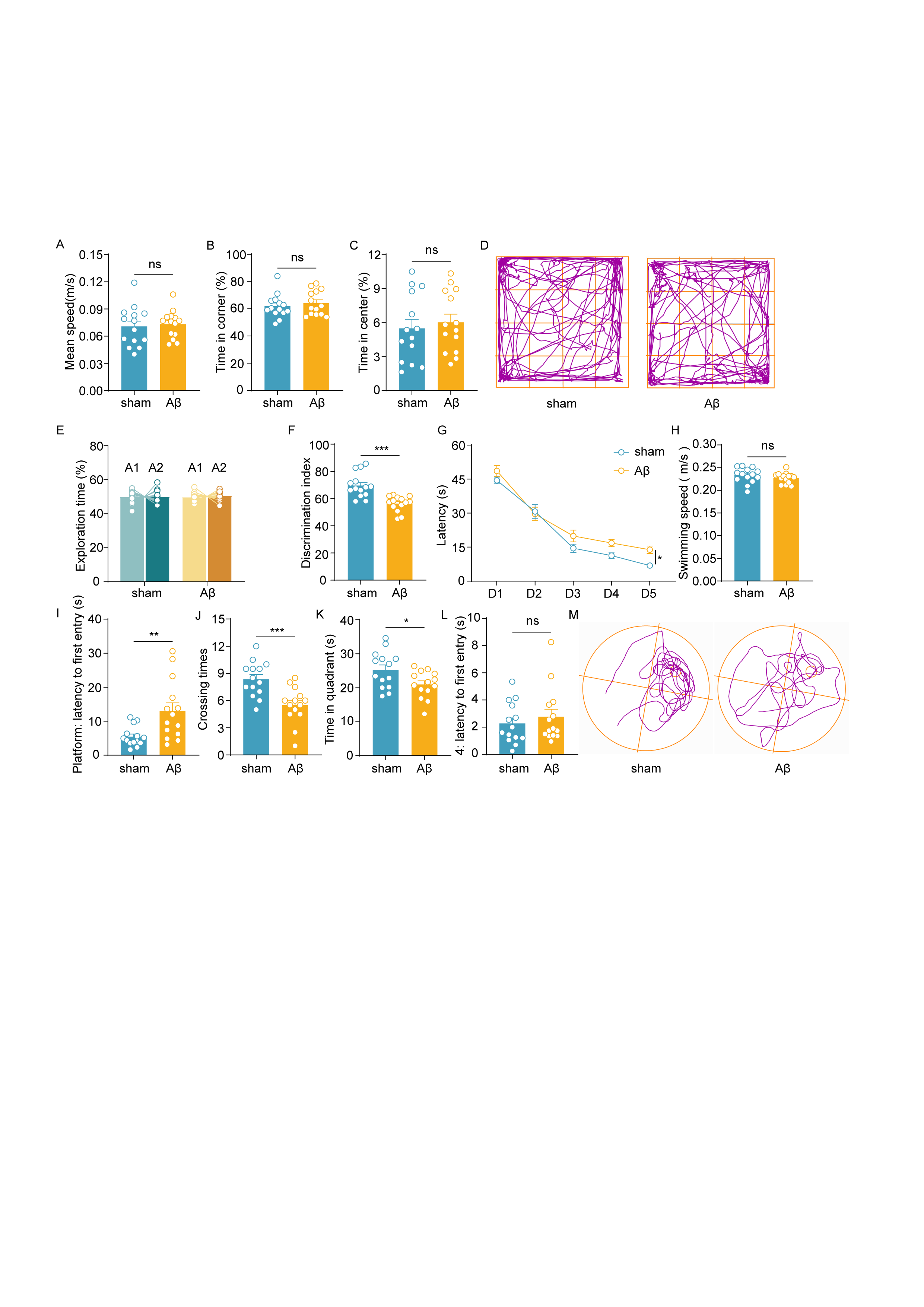


(A-C) The locomotion speed and the ratio of time spent in the center and corner zones were measured in the open field test. n = 14 for each group. t(26) = 0.3516, p = 0.7279 for locomotion speed; t(26) = 0.6827, p = 0.5008 for time spent in the corner; t(26) = 0.5062, p = 0.6170 for time spent in the center. (D) Representative movement tracks in the open field test. (E and F) Ratio of time spent exploring the same object (E) and the novel object (F) was measured in NOR tests. n = 10-12 for each group. t(26) = 4.687, p < 0.0001. (G) The escape latency in the training session of MWM tests was analyzed. n = 14 for each group. F (1, 26) = 5.142, p = 0.0319. (H-L) In the probe session, the swimming speed (H), the escape latency to reach the platform (I), the number of platform crossings (J), time in target quadrant (K) and the latency to find the target quadrant (L) were recorded. n = 14 for each group. t(26) = 1.098, p = 0.2823 for swimming speed; t(26) = 3.058, p = 0.0051 for latency to platform; t(26) = 3.841, p = 0.0007 for the number of platform crossings; t(26) = 2.399, p = 0.0239 for time in target quadrant; p = 0.5184 for latency to target quadrant. (M) Representative movement tracks of each group during the Probe phase. The data are shown as the mean ± SEM. Shapiro-Wilk test for A-C, F, H-L, w = 0.9441, p = 0.4735 for sham in A, w = 0.9410, p = 0.4311 for Aβ in A; w = 0.9226, p = 0.2394 for sham in B, w = 0.8897, p = 0.0800 for Aβ in B; w = 0.9195, p = 0.2163 for sham in C, w = 0.9273, p = 0.2799 for Aβ in C; w = 0.9077, p = 0.1458 for sham in F, w = 0.8898, p = 0.0802 for Aβ in F; w = 0.9462, p = 0.5039 for sham in H, w = 0.9350, p = 0.3585 for Aβ in H; w = 0.8821, p = 0.0623 for sham in I, w = 0.8911, p = 0.0837 for Aβ in I; w = 0.9833, p = 0.9897 for sham in J, w = 0.9423, p = 0.4491 for Aβ in J; w = 0.9512, p = 0.5795 for sham in K, w = 0.9580, p = 0.6908 for Aβ in K; w = 0.9356, p = 0.3654 for sham in L, w = 0.7829, p = 0.0031 for Aβ in L; Unpaired two-tailed t test for A-C, F, H-K. Mann - Whitney test for L. Two-way ANOVA followed by Bonferroni’s post hoc test for G. *p < 0.05, **p < 0.01, ***p < 0.001; ns no significance.

**Figure S2 AIM2 overexpression in vivo did not affect motor activity and anxiety performance**


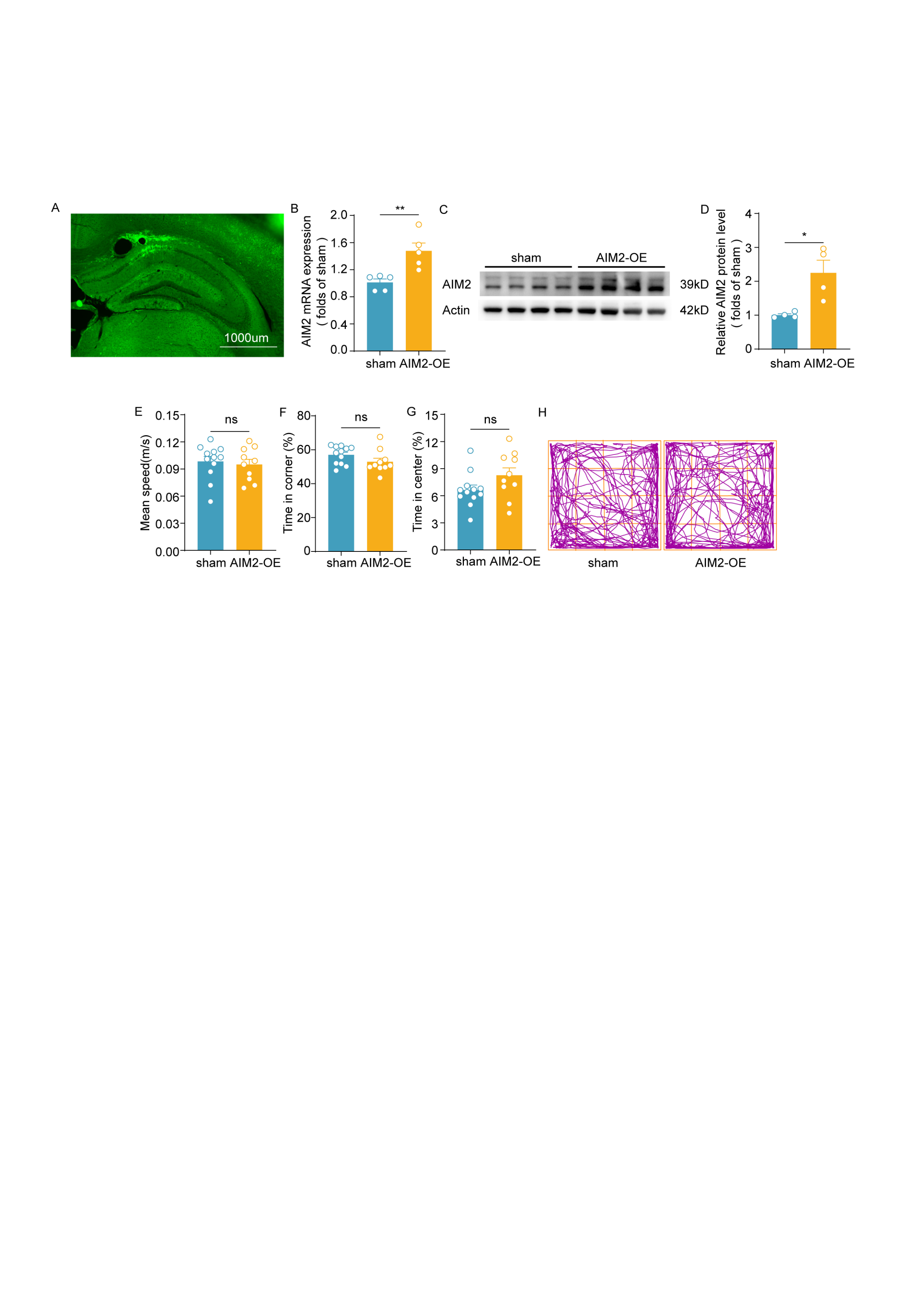


(A) Representative images of immunofluorescence for GFP expression in the hippocampus. (B) AIM2 mRNA expression was quantified by qPCR relative to GAPDH mRNA. n = 5 for each group. p = 0.0079. (C and D) The level of AIM2 in the hippocampus was determined by western blotting and quantified with β-Actin as a loading control. n = 4 for each group. t (6) = t=3.301, p = 0.0164. (E-G) The locomotion speed and the ratio of time spent in the center and corner zones were measured in the open field test. n = 10-12 for each group. t (20) = 0.4241, p = 0.6760 for locomotion speed; t (20) = 1.584, p = 0.1289 for time spent in the corner; t (20) = 1.712, p = 0.1024 for time spent in the center. (H) Representative movement tracks in the open field test. The data are shown as the mean ± SEM. Shapiro-Wilk test for B, D-G, w = 0.7447, p = 0.0266 for sham in B, w = 0.9612, p = 0.8164 for AIM2-OE in B; w = 0.8817, p = 0.3458 for sham in D, w = 0.8892, p = 0.3793 for AIM2-OE in D; w = 0.8905, p = 0.1196 for sham in E, w = 0.9494, p = 0.6619 for AIM2-OE in E; w = 0.9100, p = 0.2136 for sham in F, w = 0.9735, p = 0.9215 for AIM2-OE in F; w = 0.8935, p = 0.1308 for sham in G, w = 0.8739, p = 0.1109 for AIM2-OE in G; Unpaired two-tailed t test for D-G. Mann - Whitney test for B. *p < 0.05, **p < 0.01; ns no significance.

**Figure S3 The expression of AIM2 in different cell types in CA1 region of sham and AD mice.**


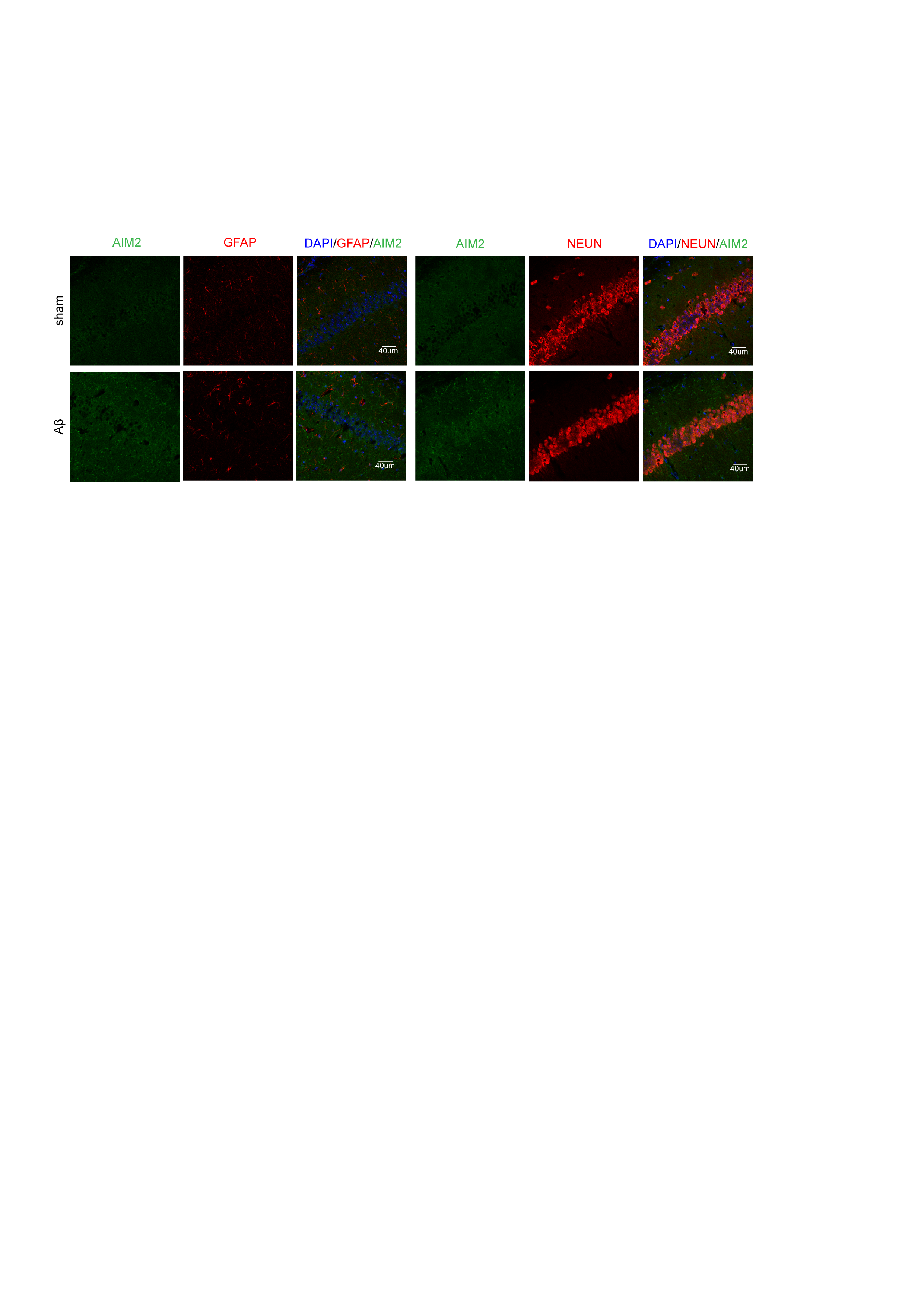


Double immunofluorescence staining of AIM2 (green) with NEUN (red) and GFAP (red) in CA1 region of sham and AD mice.

**Figure S4 AIM2-cKO mice displayed normal body weight and gross brain structure**


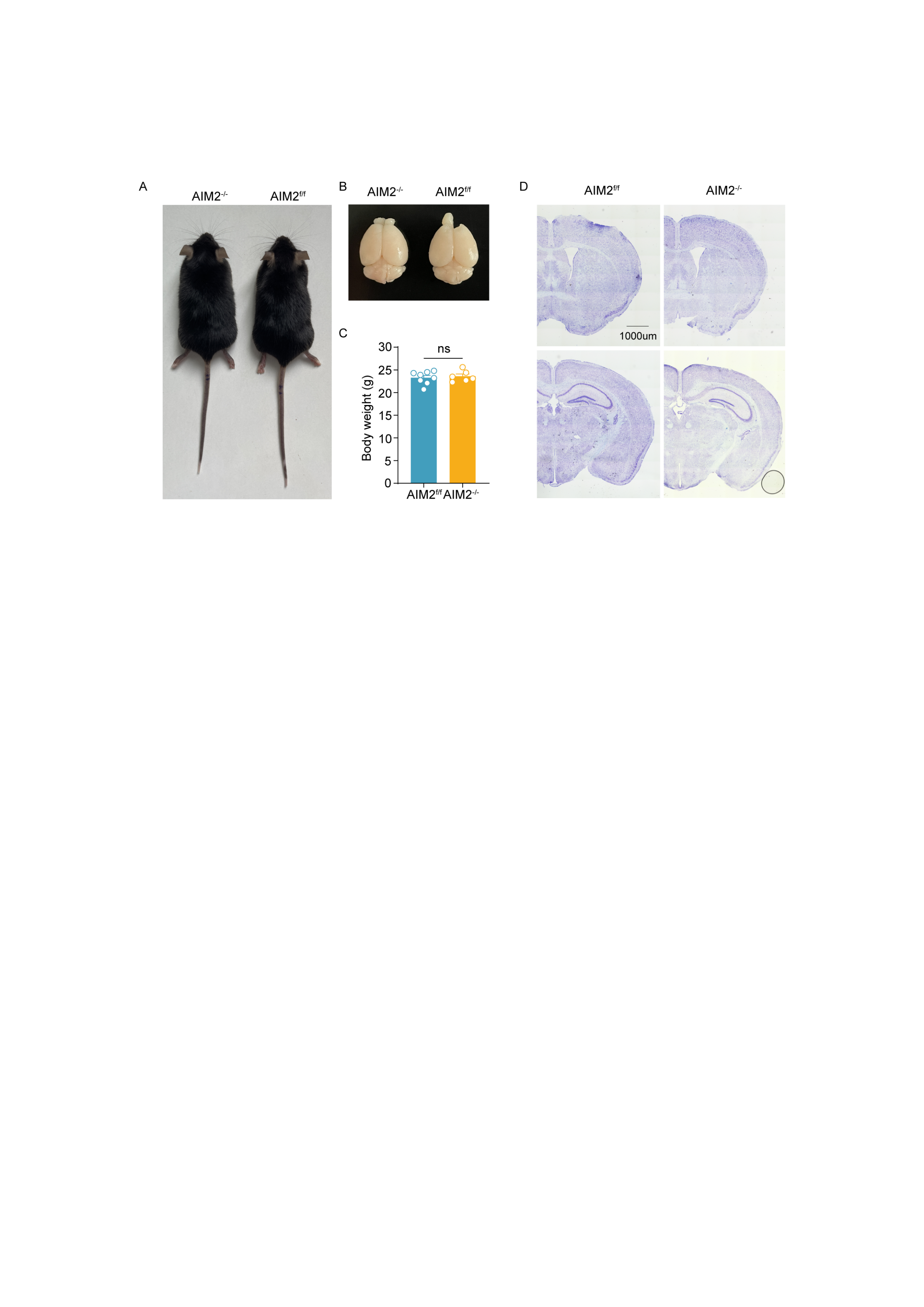


(A-B) Representative images showing overall appearance and brain of AIM2-cKO and control mice. (C) Quantification of body weight for AIM2-cKO and control mice. n = 6-8 for each group. t (12) =0.4547, p = 0.6574. (D) Representative Nissl-staining images of brains from AIM2-cKO and control mice. The data are shown as the mean ± SEM. Shapiro-Wilk test for C. w = 0.9306, p = 0.5219 for AIM2^f/f^ in C, w = 0.9378, p = 0.6414 for AIM2^-/-^ in C. Unpaired two-tailed t test for C. ns no significance.

**Figure S5 AIM2 deficiency in microglia didn't affect motor activity and anxiety performance**


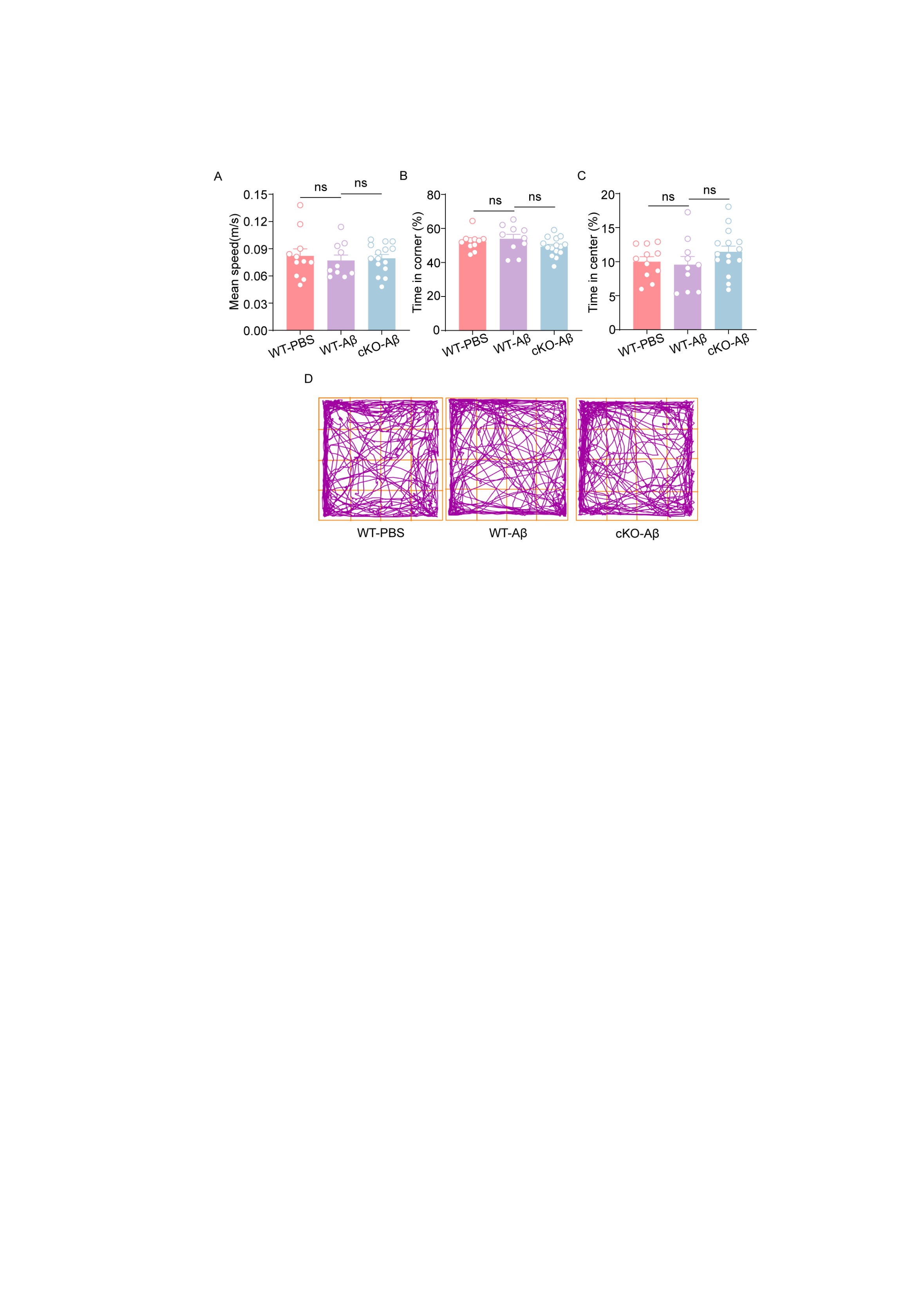


(A-C) The locomotion speed and the ratio of time spent in the center and corner zones were measured in the open field test. n = 10-15 for each group. F(2,33) = 0.3216, AD vs. control group: p = 0.8470, AIM2-cKO-AD vs. AD group: p = 0.9564 for locomotion speed; F(2,33) = 1.827, AD vs. control group: p = 0.7976, AIM2-cKO-AD vs. AD group: p = 0.1707 for time spent in the corner; F(2,33) = 0.5359, AD vs. control group: p = 0.9462, AIM2-cKO-AD vs. AD group: p = 0.3360 for time spent in the center. (D) Representative movement tracks in the open field test. The data are shown as the mean ± SEM. Shapiro-Wilk test for A-C. w = 0.9014, p = 0.1924 for sham in A, w = 0.8697, p = 0.0992 for AD in A, w = 0.9383, p = 0.3617 for AIM2-cKO-AD in A; w = 0.8722, p = 0.0827 for sham in B, w = 0.9410, p = 0.5645 for AD in B, w = 0.9855, p = 0.9940 for AIM2-cKO-AD in B; w = 0.9323, p = 0.4344 for sham in C, w = 0.9246, p = 0.3969 for AD in C, w = 0.9760, p = 0.9348 for AIM2-cKO-AD in C. One-way ANOVA followed by Dunnett’s post hoc test for A-C. ns no significance.

**Figure S6 C3aR antagonist improved cognitive deficits in AIM2-OE mice.**


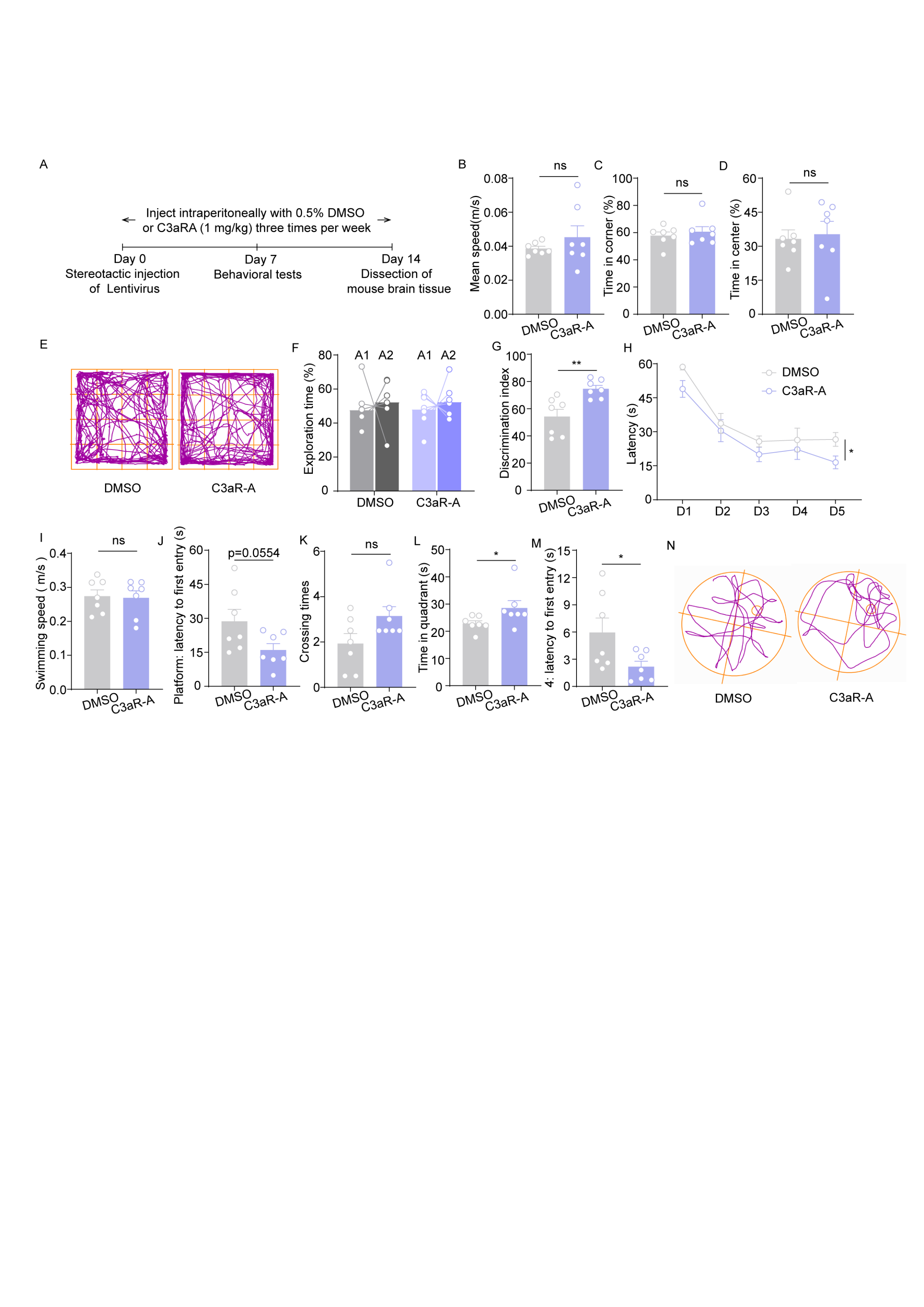


(A) Flow chart of the experimental design. (B-D) The locomotion speed and the ratio of time spent in the center and corner zones were measured in the open field test. n = 7 for each group. t(12) = 0.9796, p = 0.3466 for locomotion speed; t(12) = 0.6202, p = 0.5467 for time spent in the corner; t(12) = 0.3021, p = 0.7678 for time spent in the center. (E) Representative movement tracks in the open field test. (F and G) Ratio of time spent exploring the same object (F) and the novel object (G) was measured in NOR tests. n = 7 for each group. t(12) = 3.514, p = 0.0043. (H) The escape latency in the training session of MWM tests was analyzed. n = 7 for each group. F (1, 12) = 6.861, p = 0.0224. (I-M) In the probe session, the swimming speed (I), the escape latency to reach the platform (J), the number of platform crossings (K), time in target quadrant (L) and the latency to find the target quadrant (M) were recorded. n = 7 for each group. t(12) = 0.1899, p = 0.8526 for swimming speed; t(12) = 2.122, p = 0.0554 for latency to platform; p = 0.1212 for the number of platform crossings; p = 0.0157 for time in target quadrant; t(12) =2.208, p = 0.0475 for latency to target quadrant. (N) Representative movement tracks of each group during the Probe phase. The data are shown as the mean ± SEM. Shapiro-Wilk test for B-D, G, I-M. w = 0.9481, p = 0.7123 for DMSO in B, w = 0.8846, p = 0.2476 for C3aR-A in B; w = 0.9043, p = 0.3577 for DMSO in C, w = 0.8125, p = 0.0544 for C3aR-A in C; w = 0.8630, p = 0.1610 for DMSO in D, w = 0.8735, p = 0.1991 for C3aR-A in D; w = 0.8528, p = 0.1302 for DMSO in G, w = 0.9259, p = 0.5167 for C3aR-A in G; w = 0.9177, p = 0.4517 for DMSO in I, w = 0.8170, p = 0.0600 for C3aR-A in I; w = 0.8952, p = 0.3031 for DMSO in J, w = 0.8973, p = 0.3147 for C3aR-A in J; w = 0.9327, p = 0.5745 for DMSO in K, w = 0.6838, p = 0.0025 for C3aR-A in K; w = 0.9000, p = 0.3309 for DMSO in L, w = 0.7919, p = 0.0340 for C3aR-A in L; w = 0.8575, p = 0.1438 for DMSO in M, w = 0.8459, p = 0.1127 for C3aR-A in M; Unpaired two-tailed t test for B-D, G, I, J, M. Mann - Whitney test for K and L. Two-way ANOVA followed by Bonferroni’s post hoc test for H. *p < 0.05, **p < 0.01; ns no significance.
