# Supplementary material for: Conditional knockout of AIM2 in microglia ameliorates synaptic plasticity and spatial memory deficits in a mouse model of Alzheimer's disease
Source: CNS Neurosci Ther. 2023 Dec 17;30(6):e14555. doi: 10.1111/cns.14555 (PMC11163192; doi:10.1111/cns.14555)

original pictures for Figure 1E

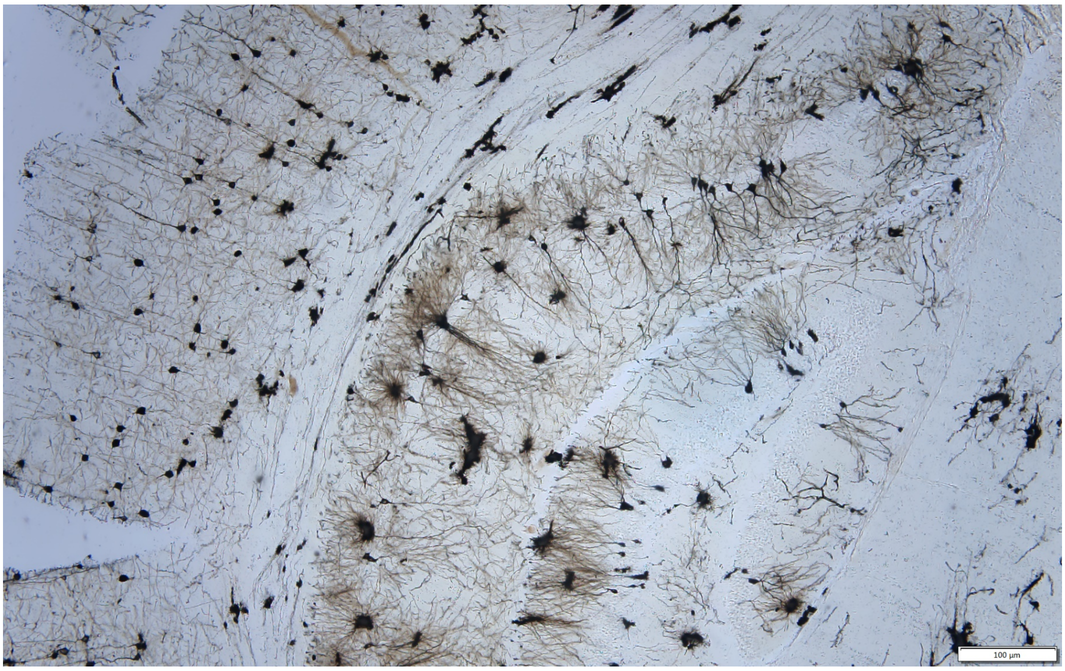

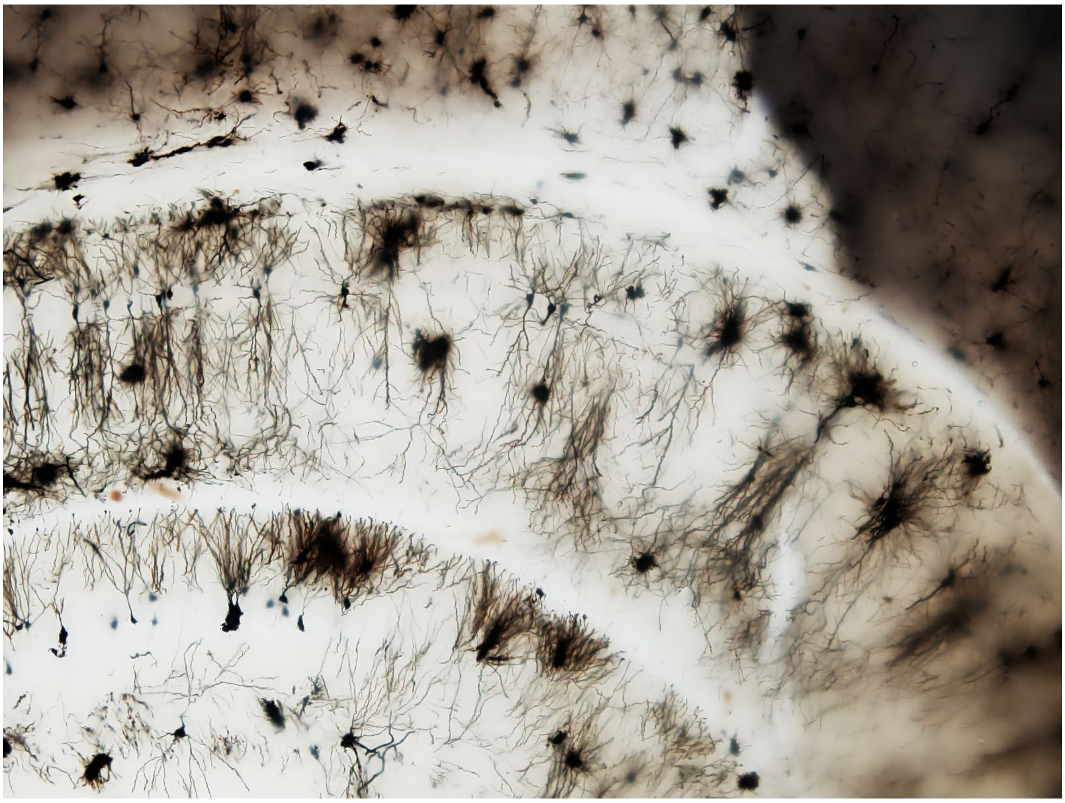

original pictures for AD group in Figure 1G

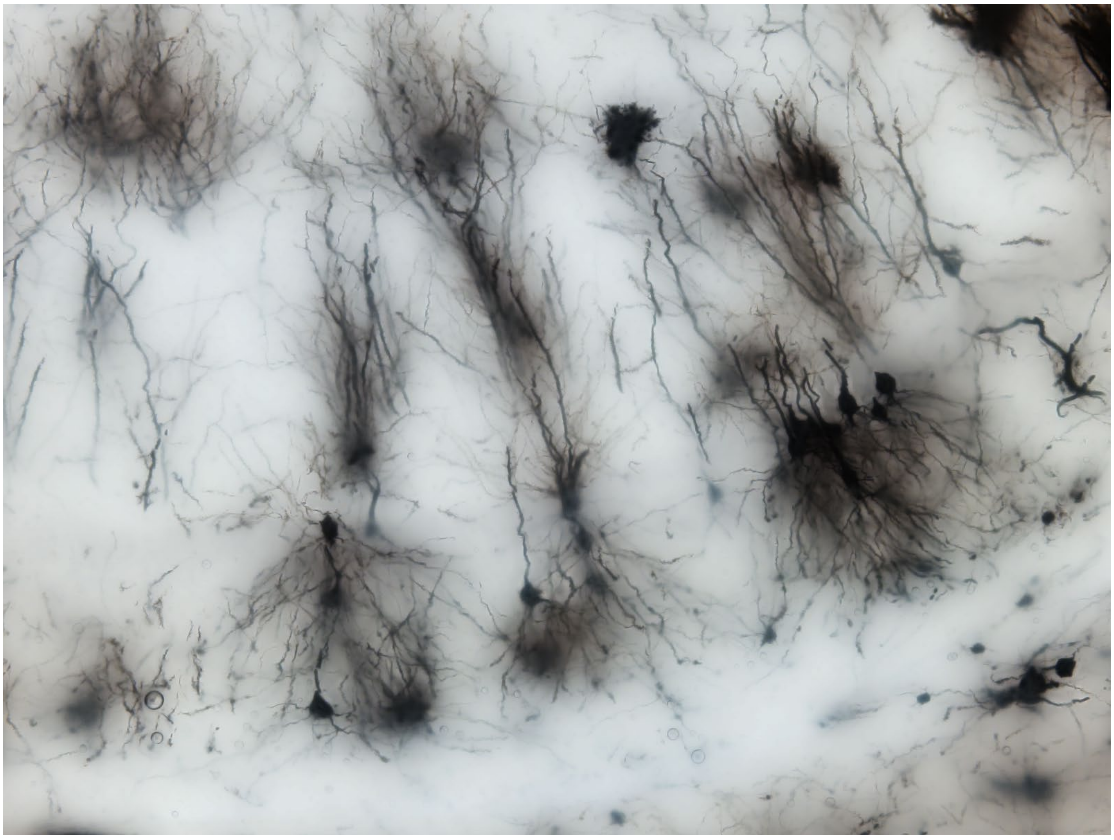

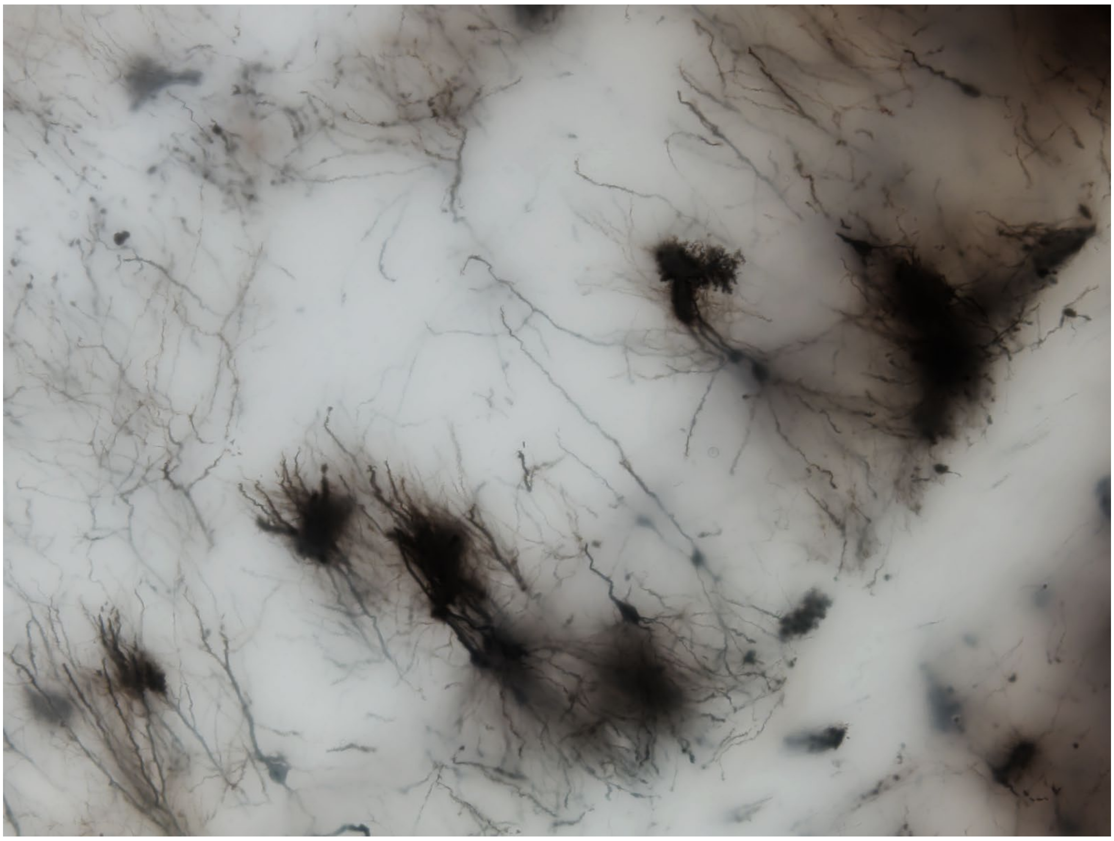

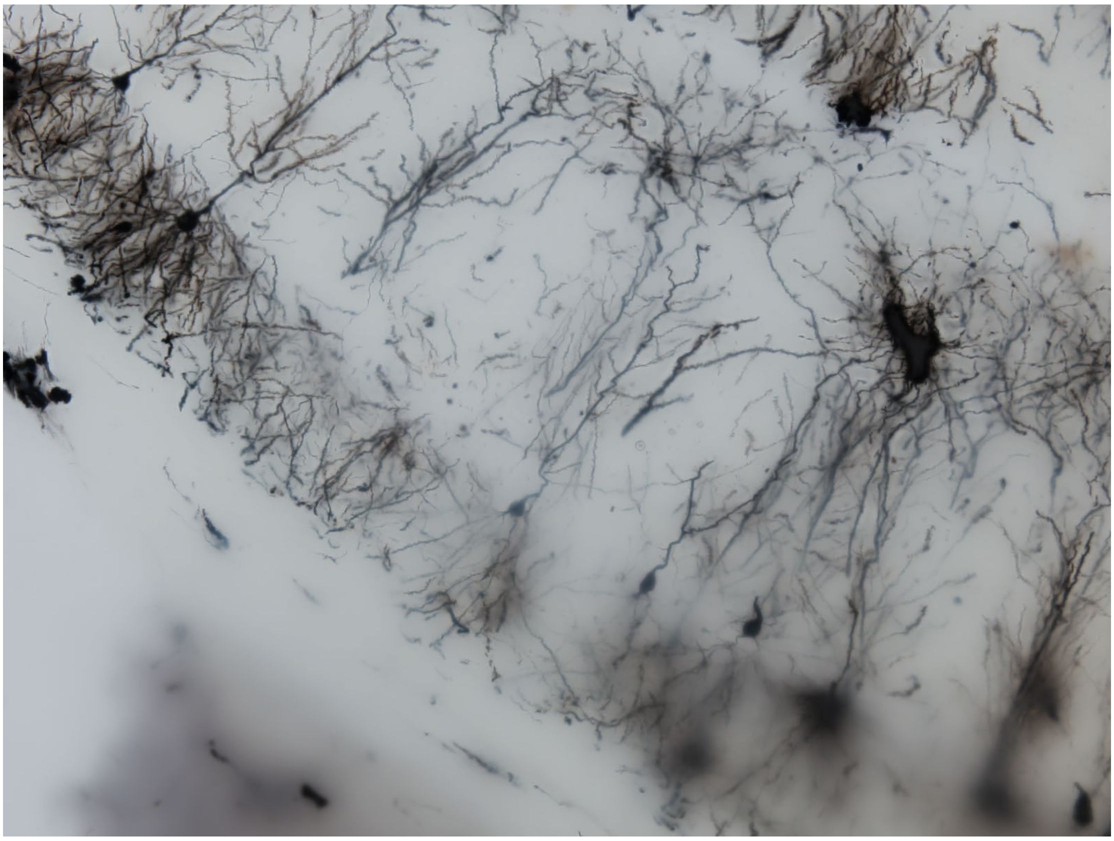

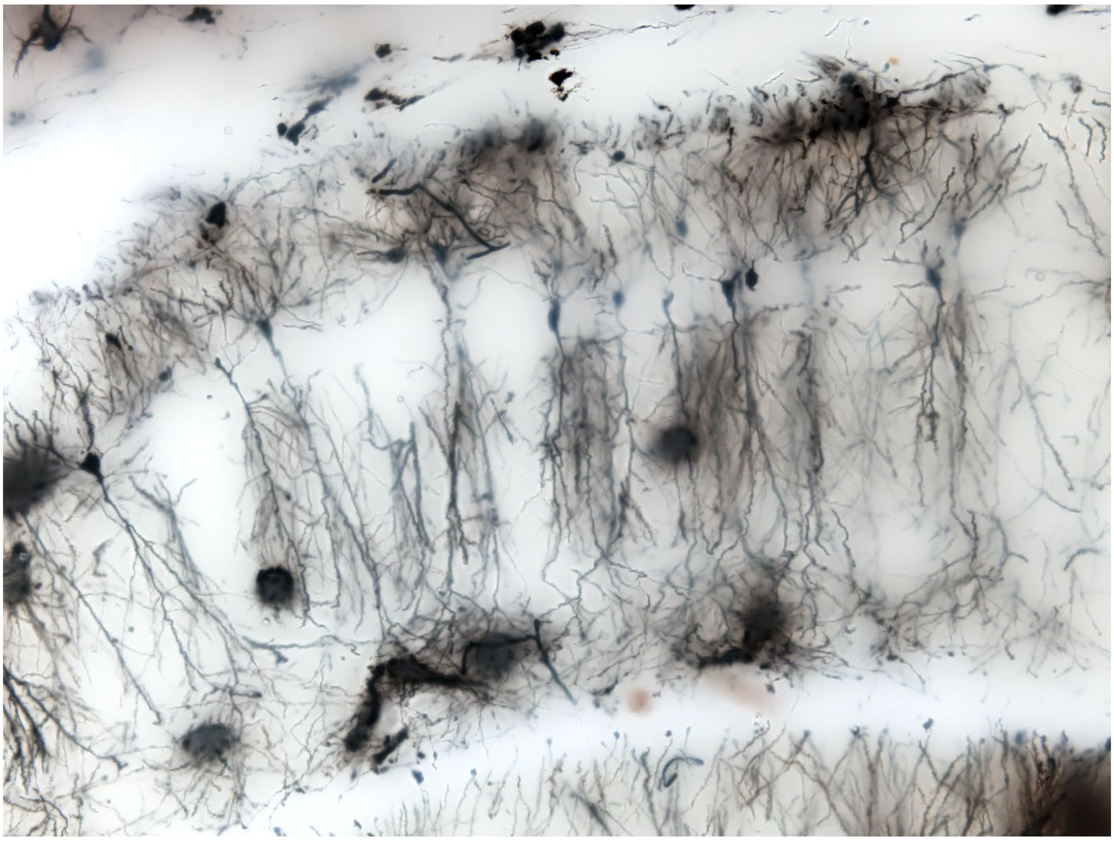

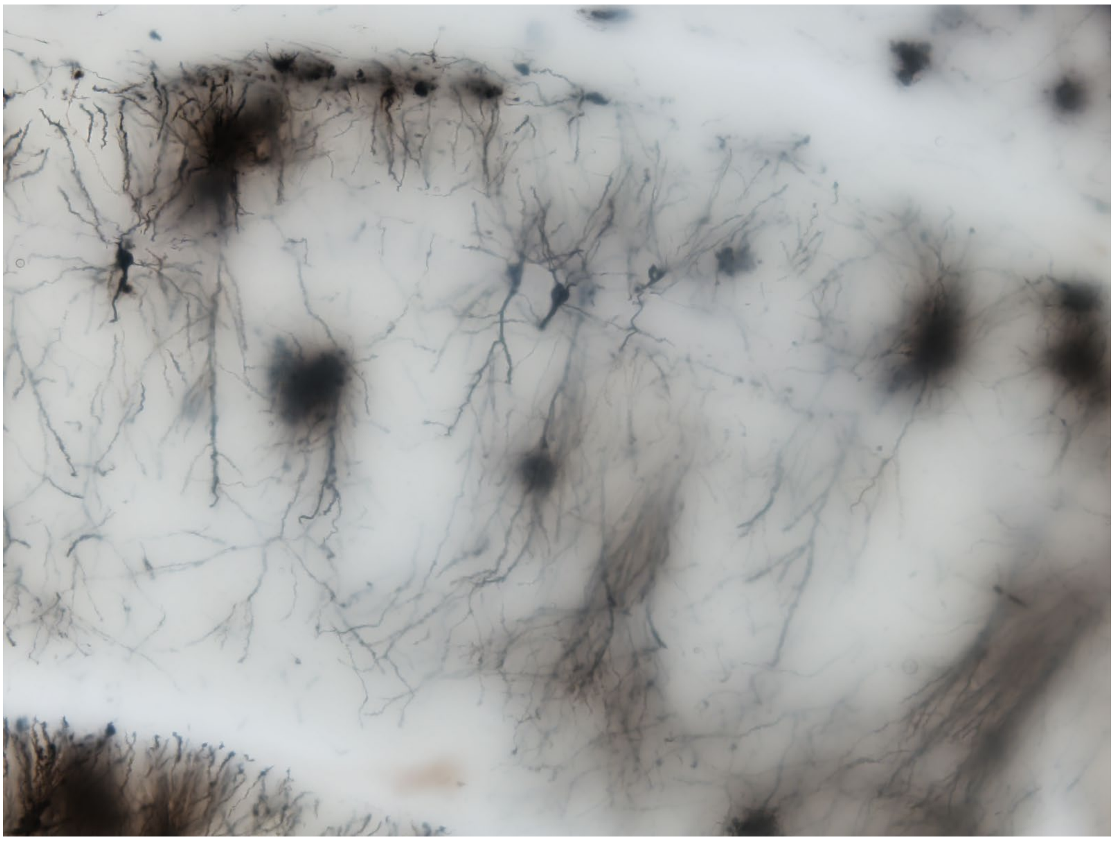

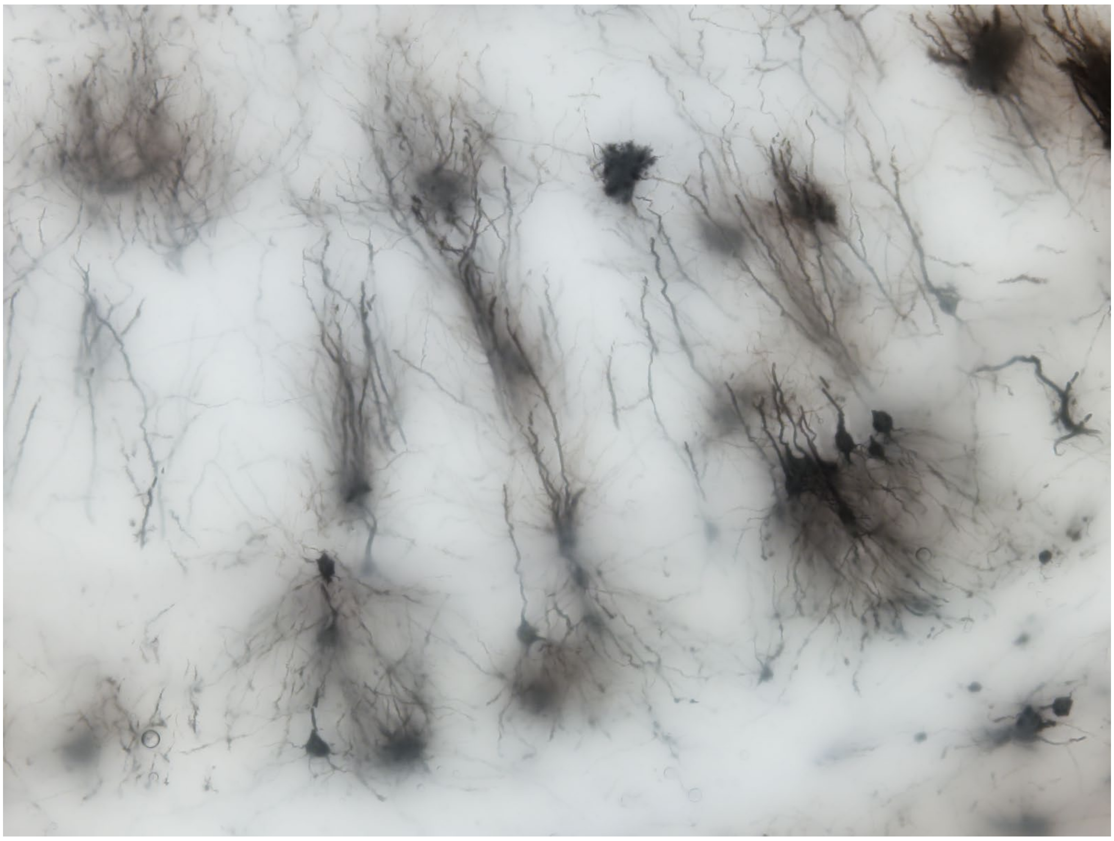

original pictures for sham group in Figure 1G

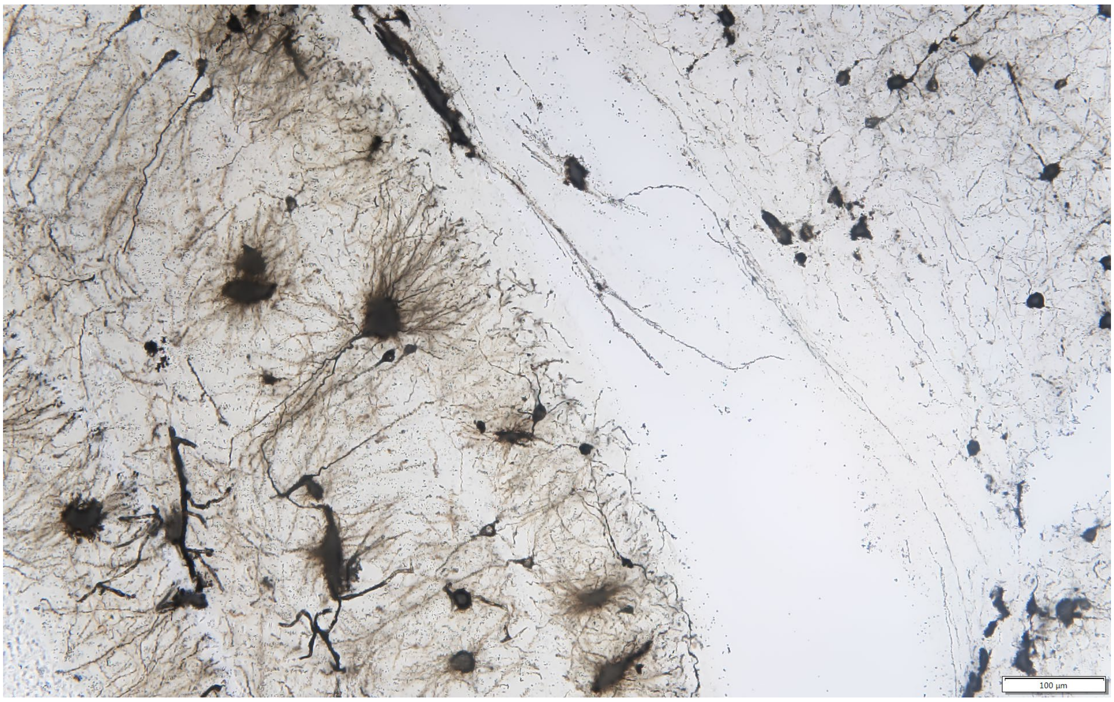

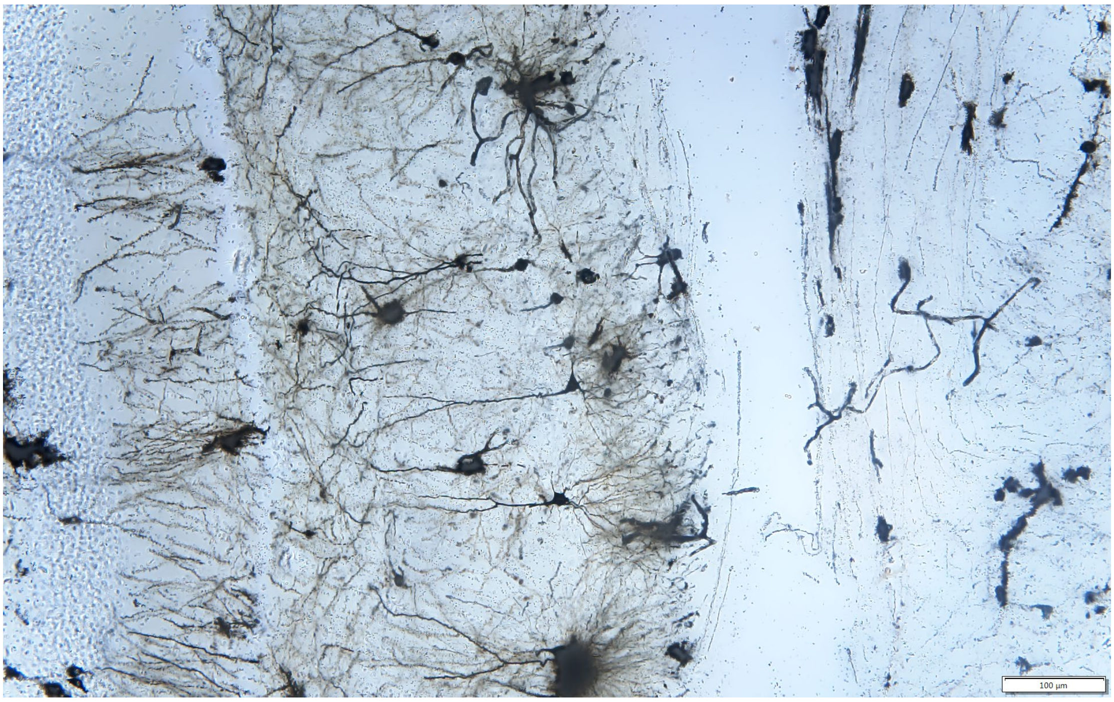

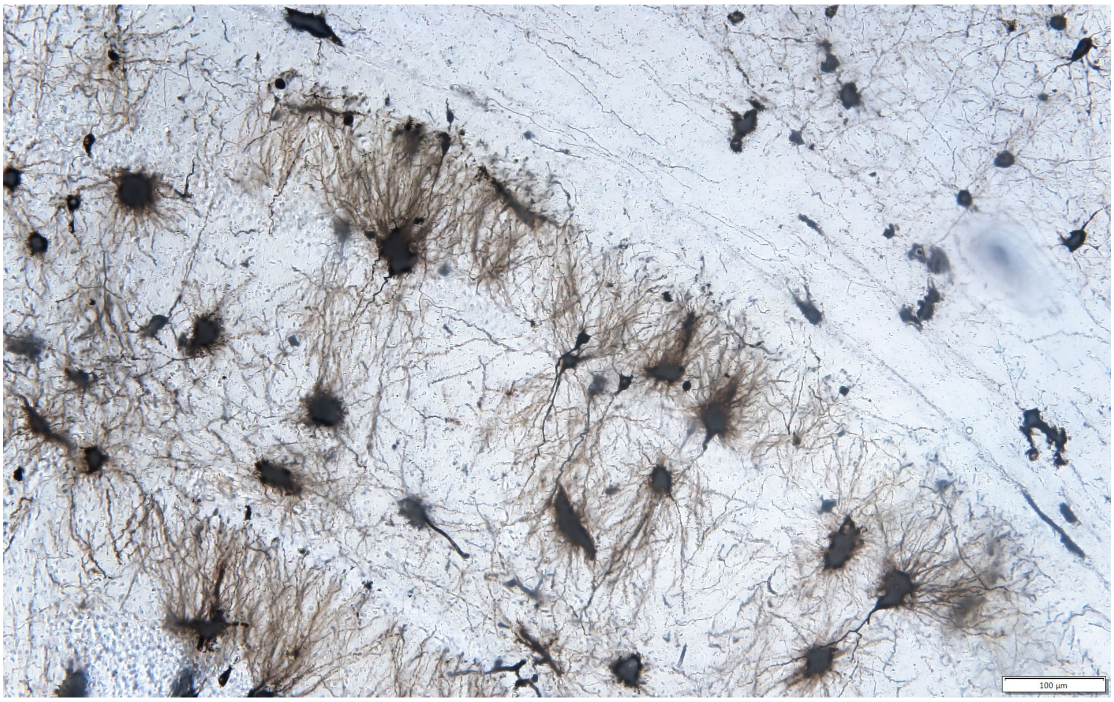
100 μm

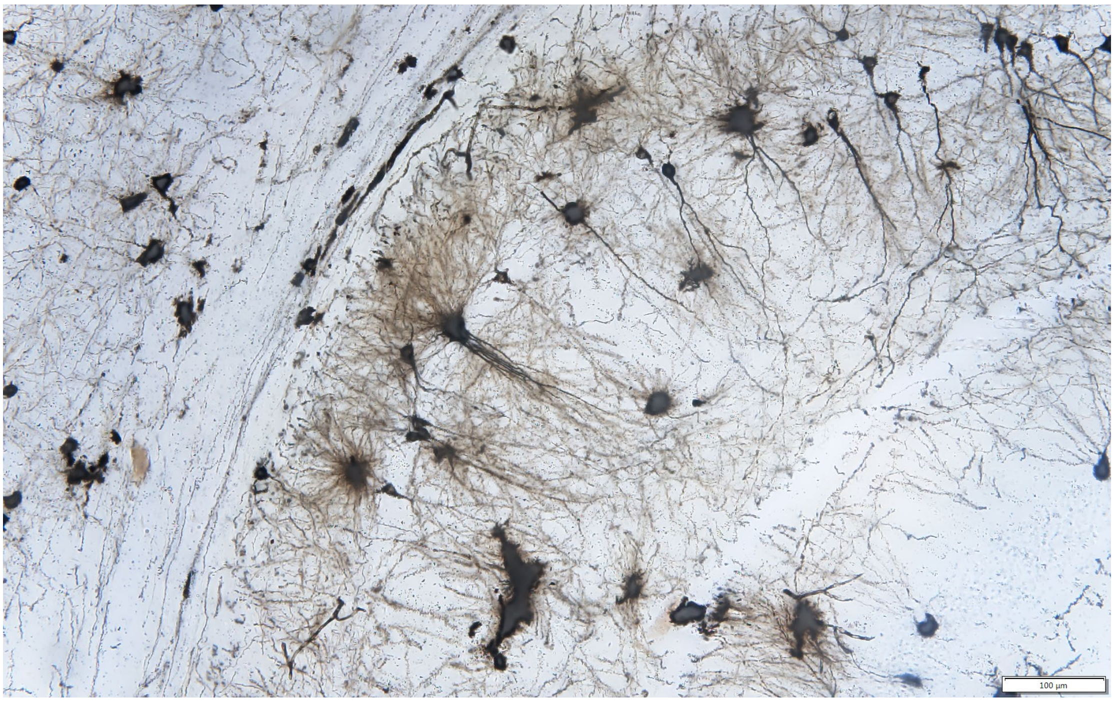
100 μm

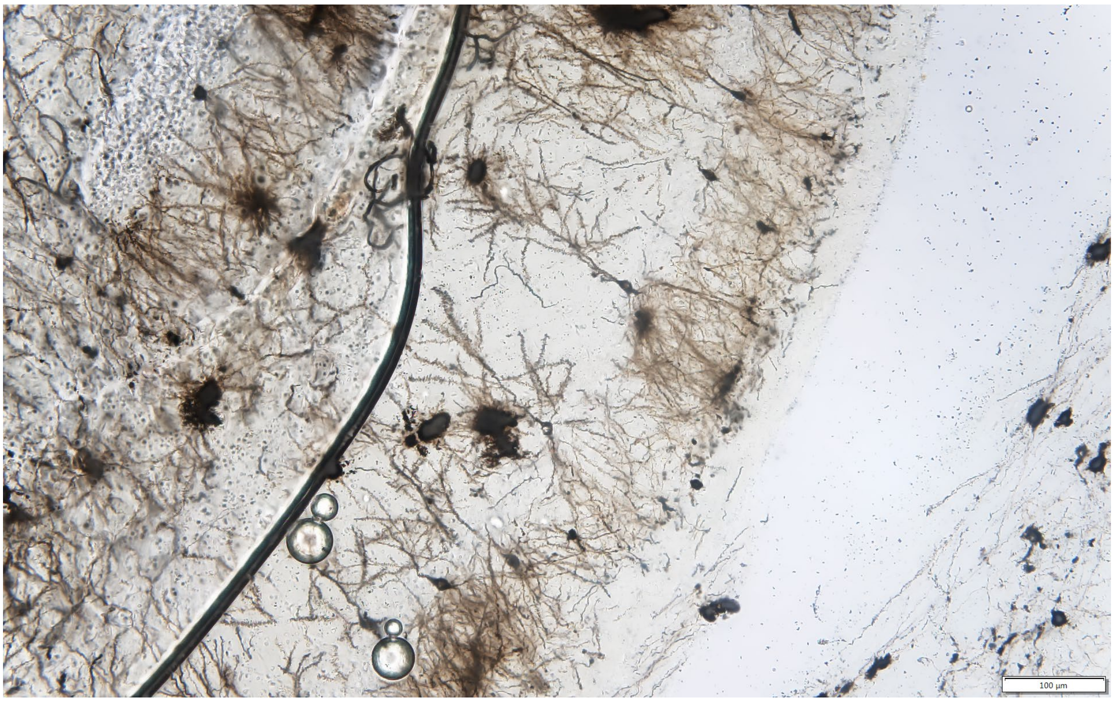
100 µm

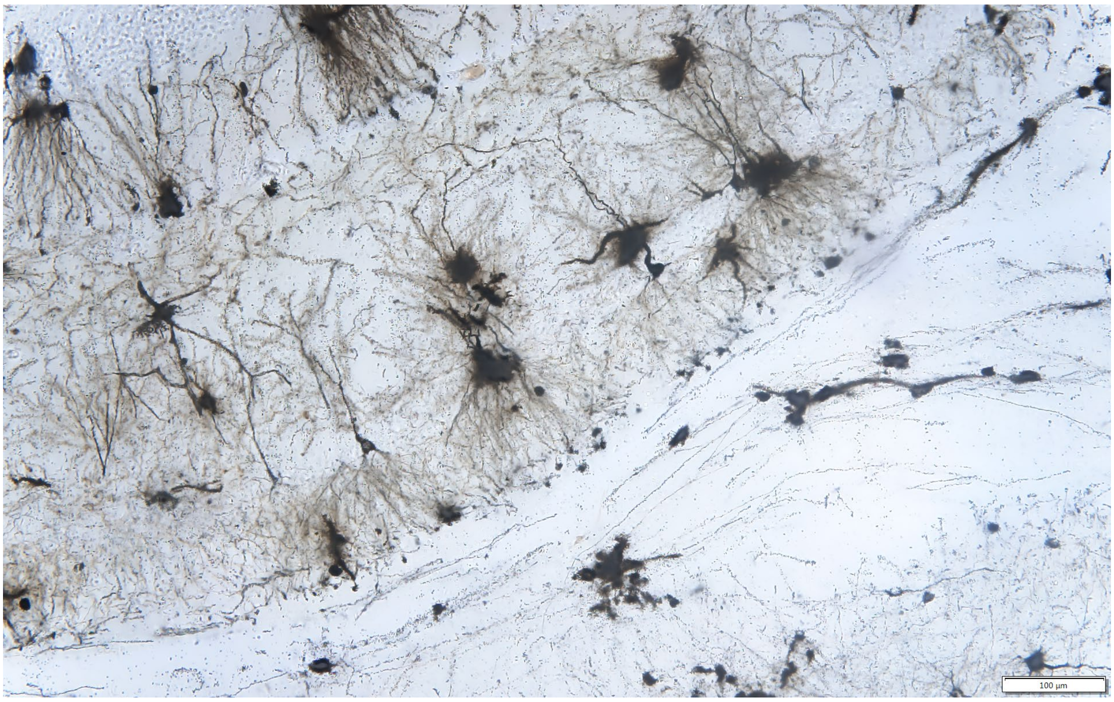
100 µm

original pictures for apical spines of AD group in Figure 1H and I

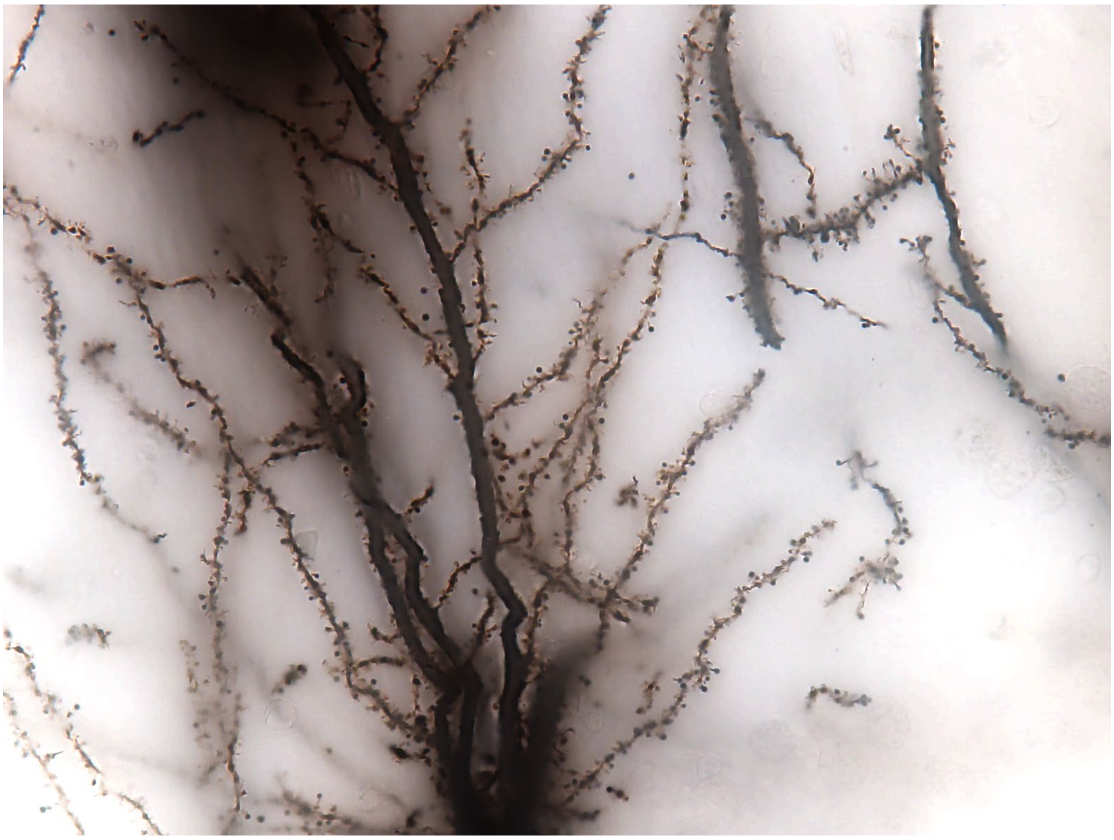

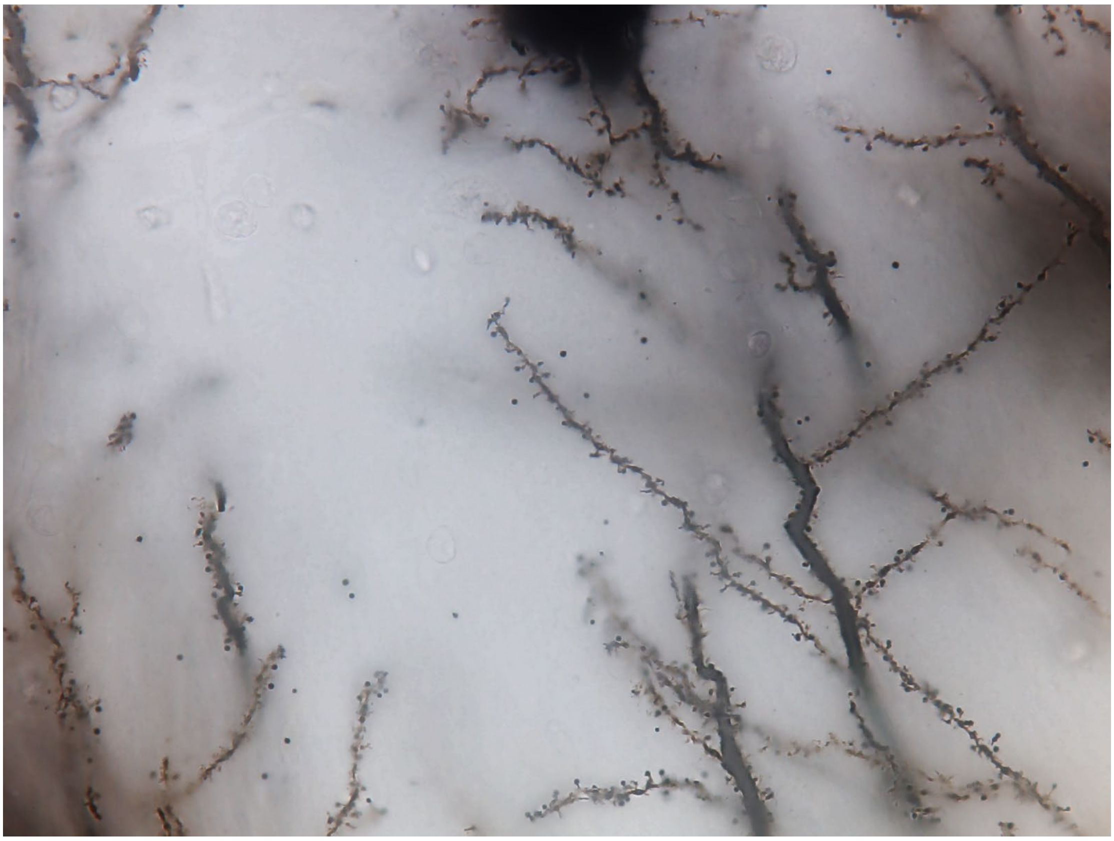

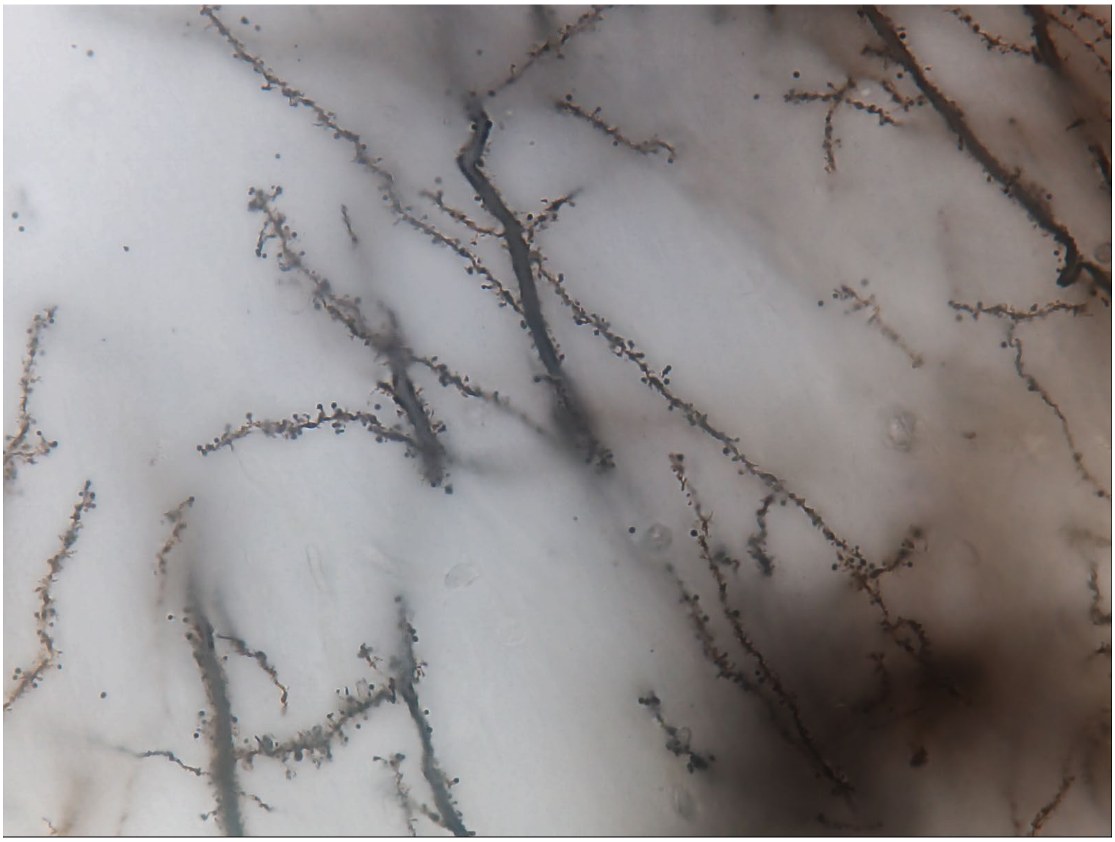

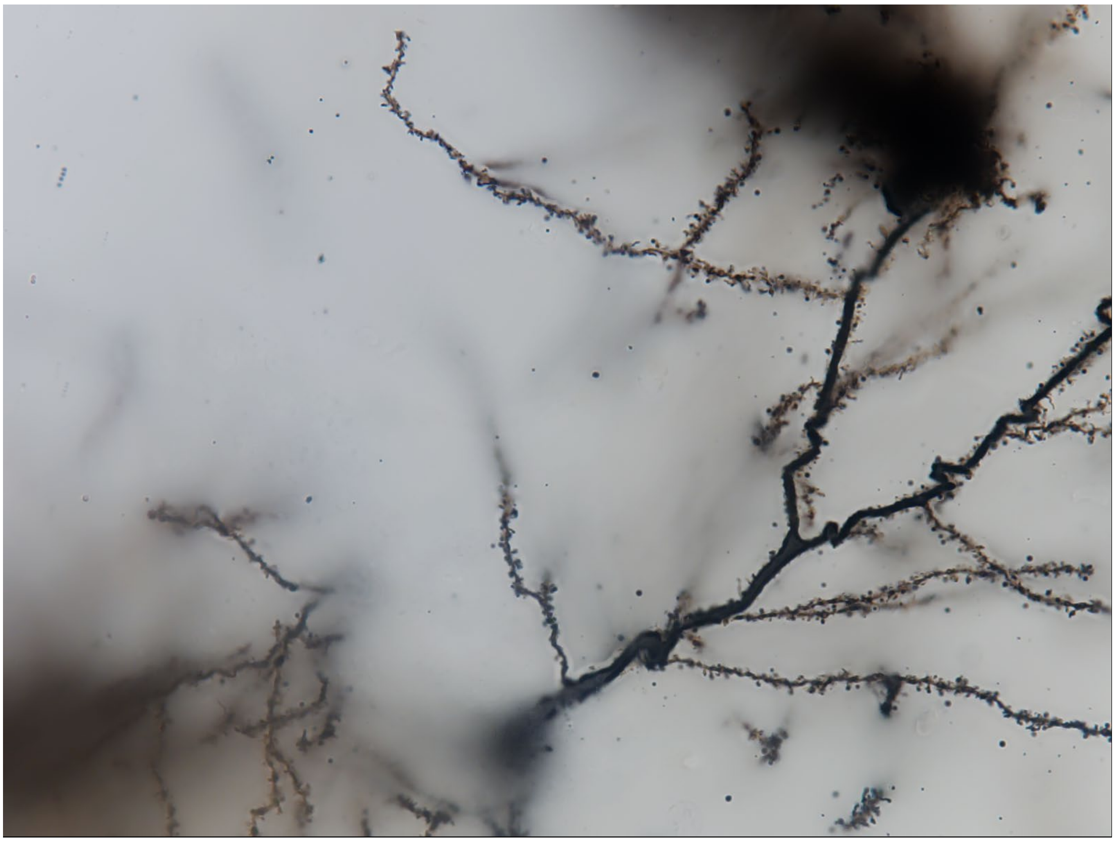

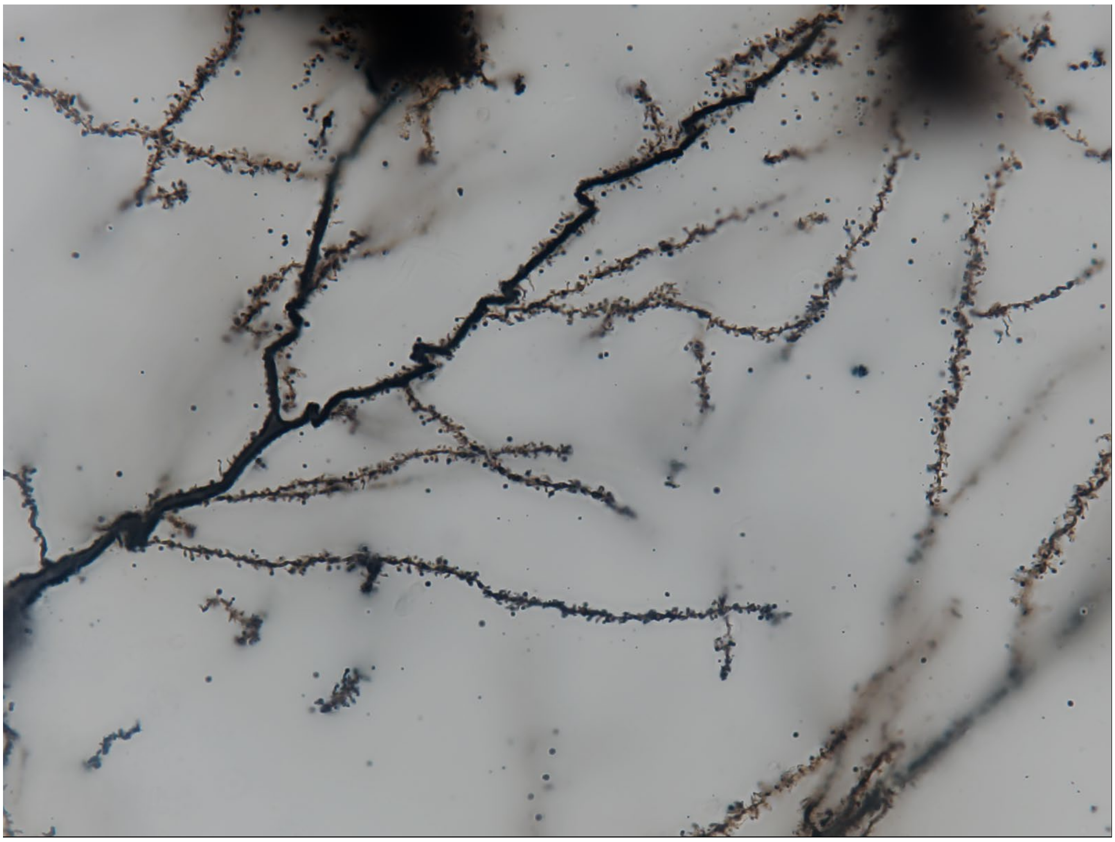

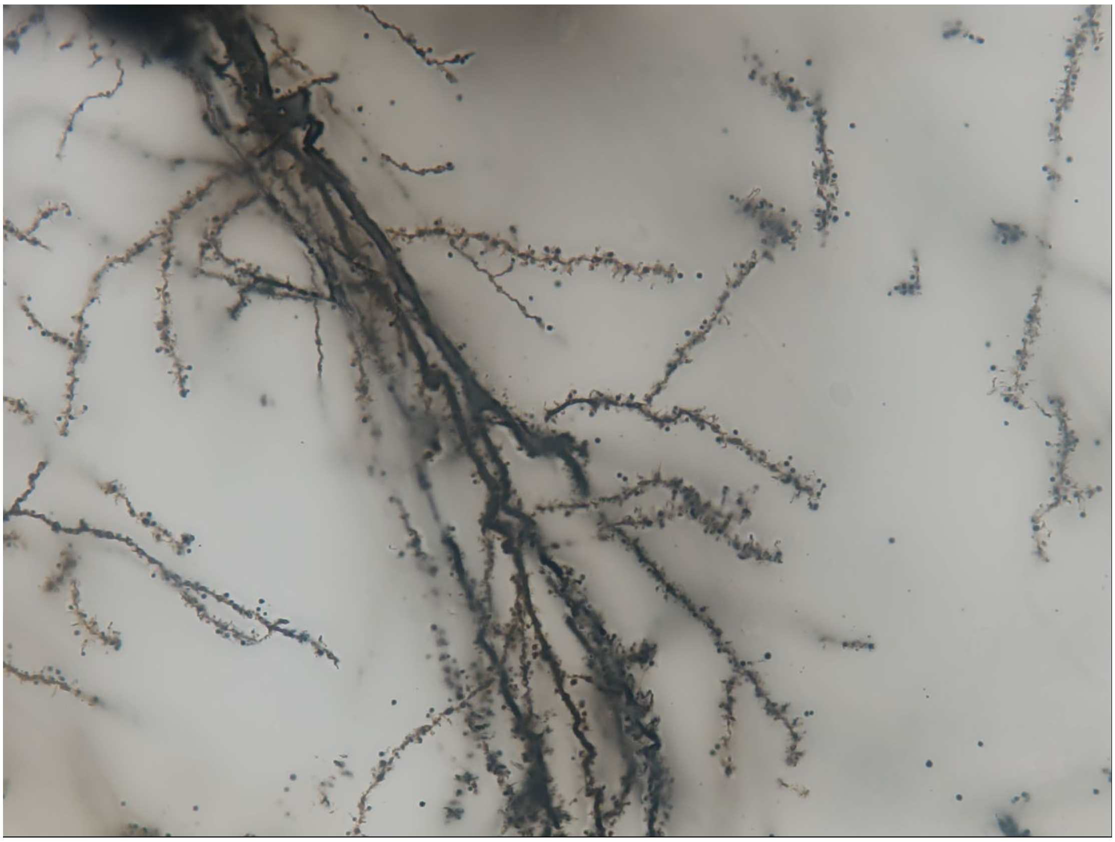

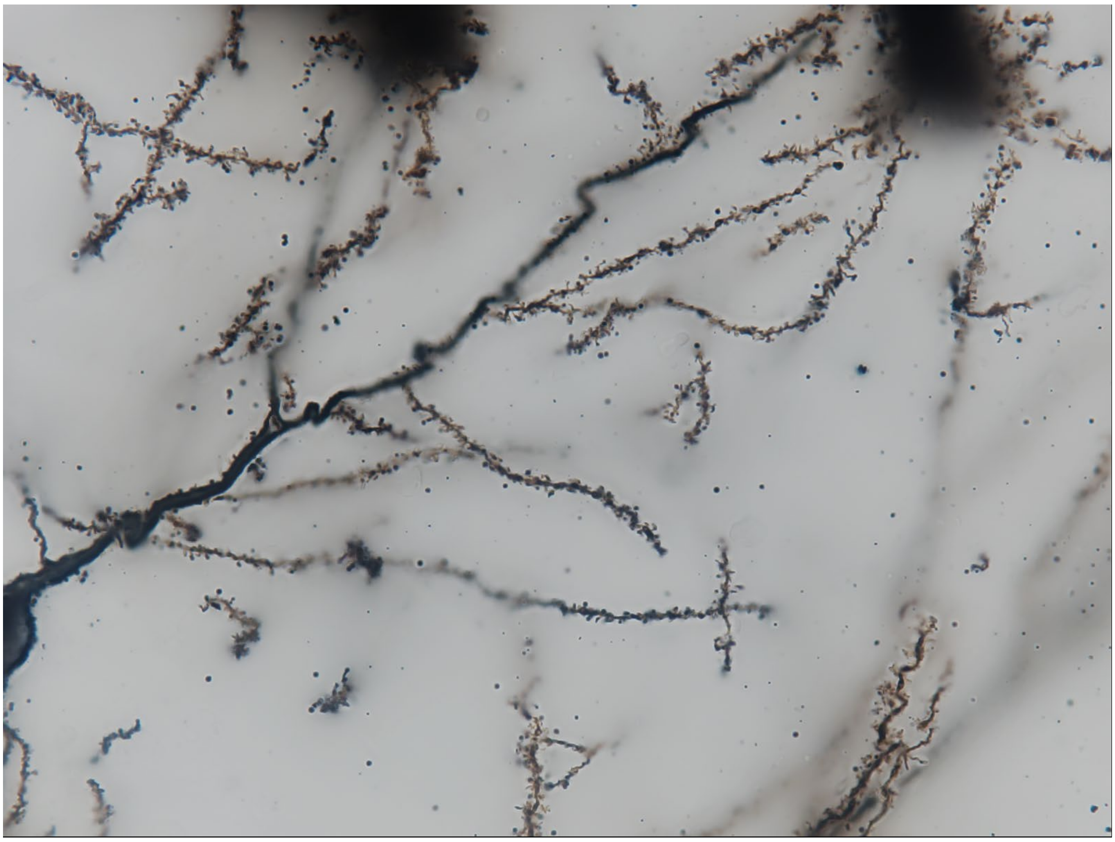

original pictures for basal spines of AD group in Figure 1H and I

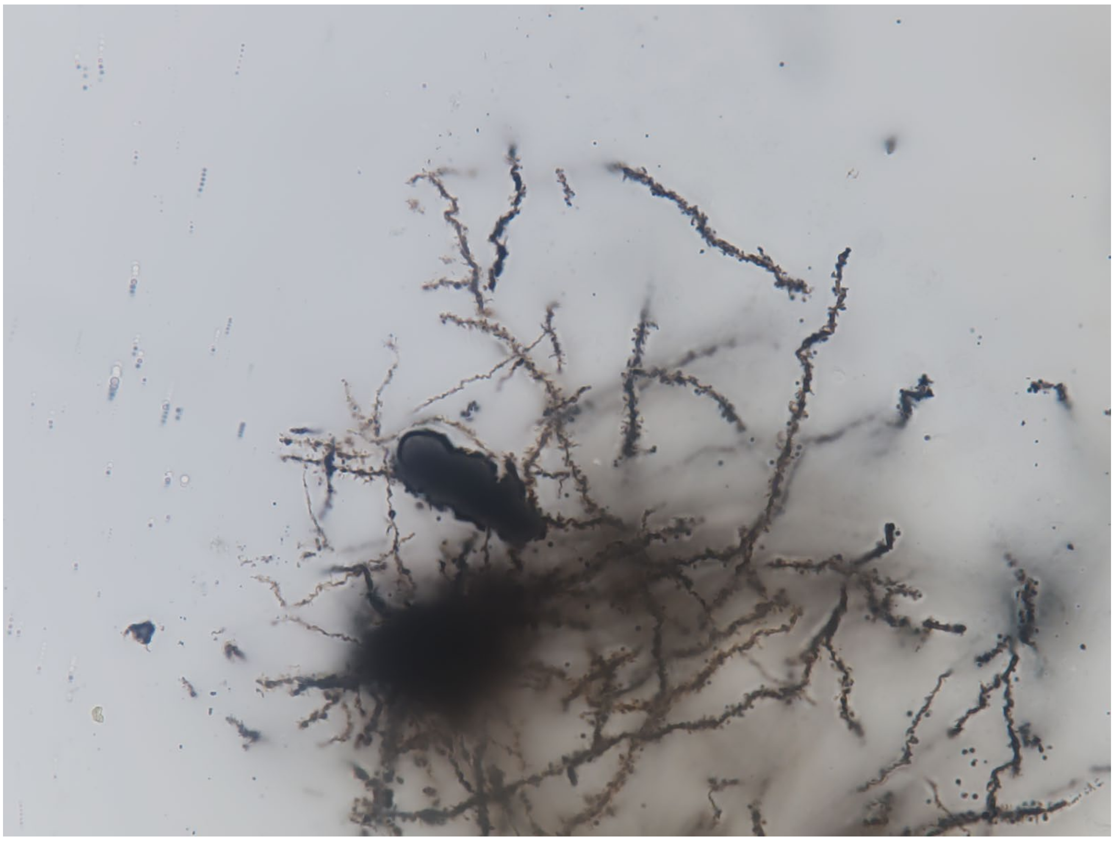

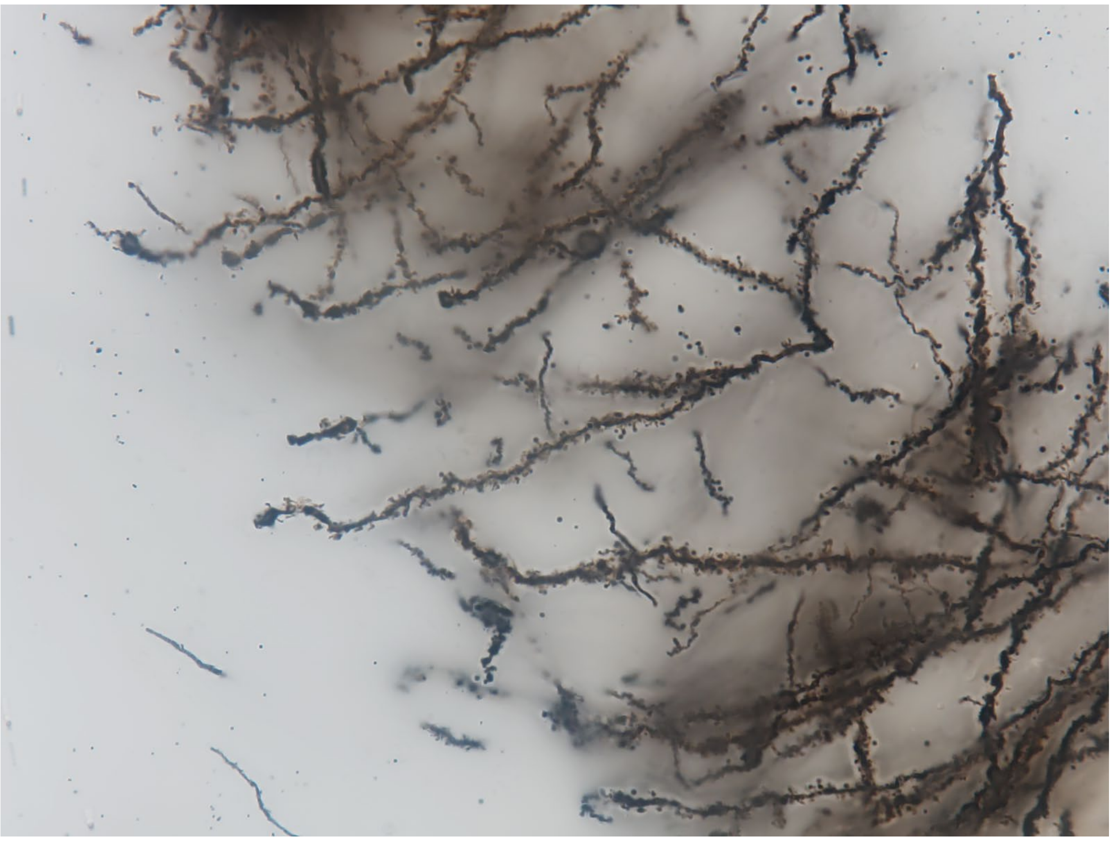

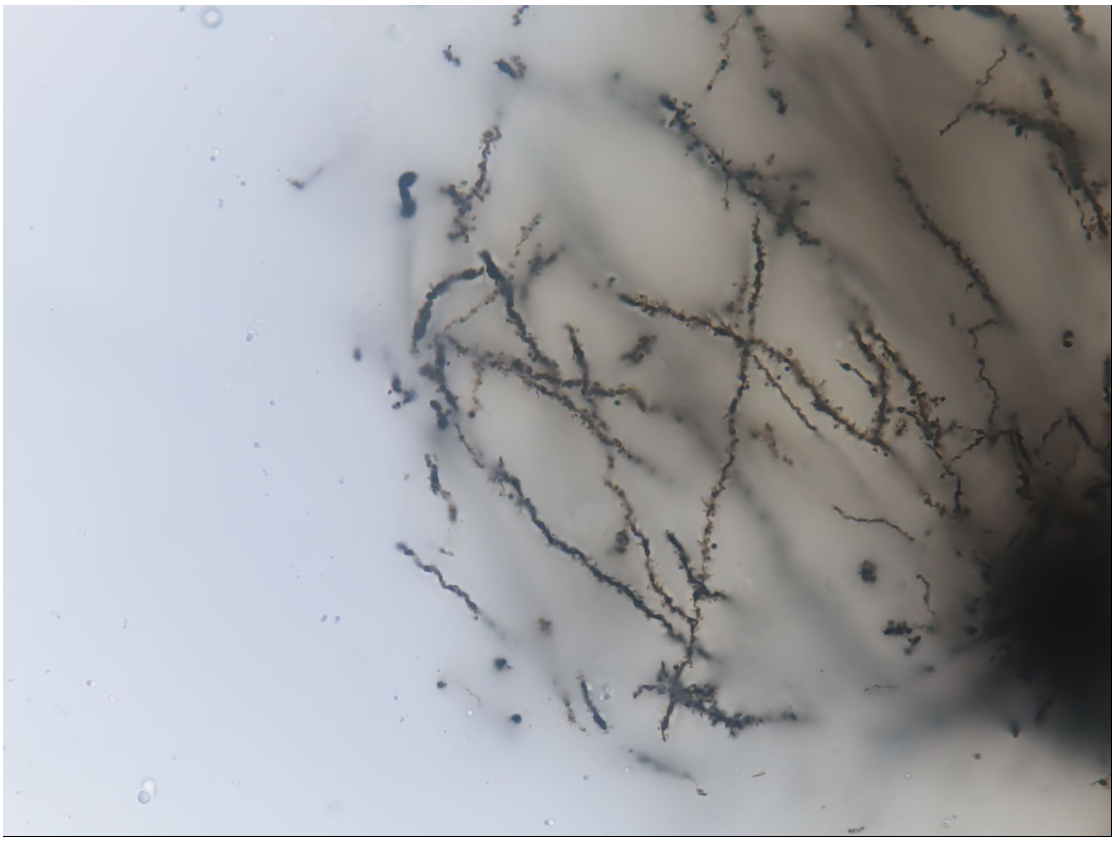

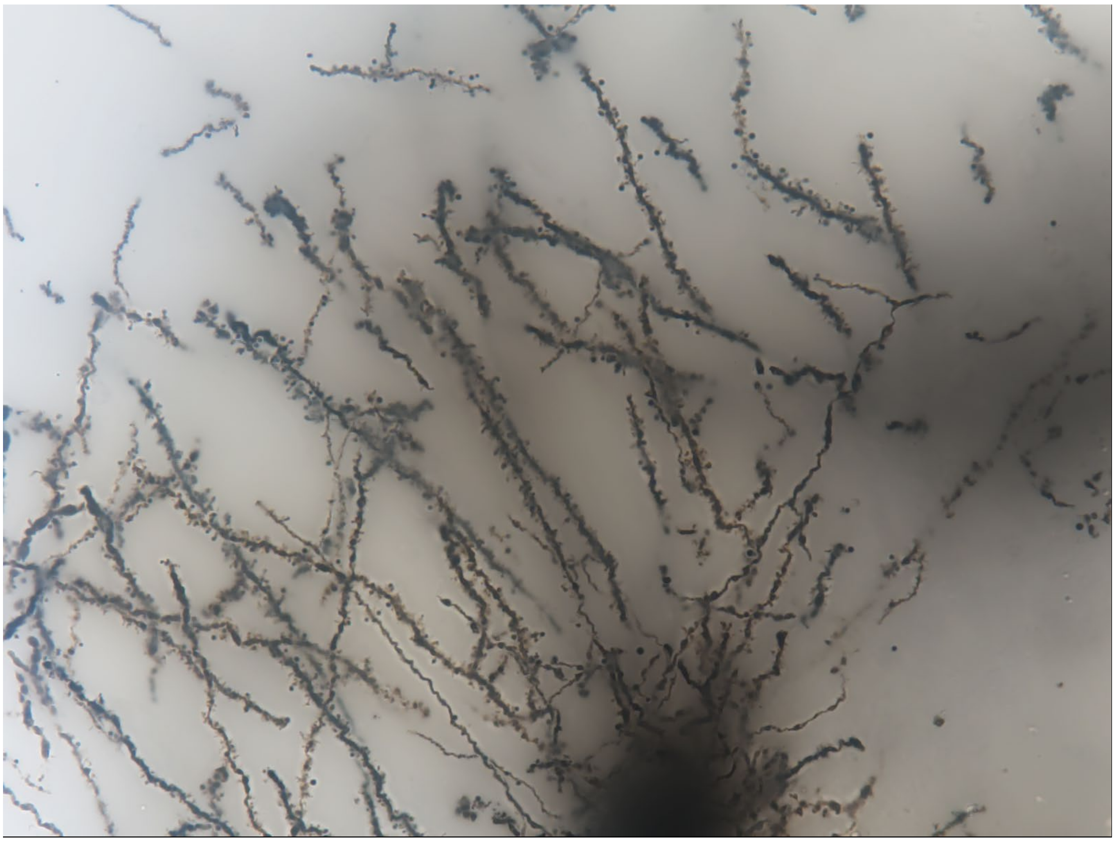
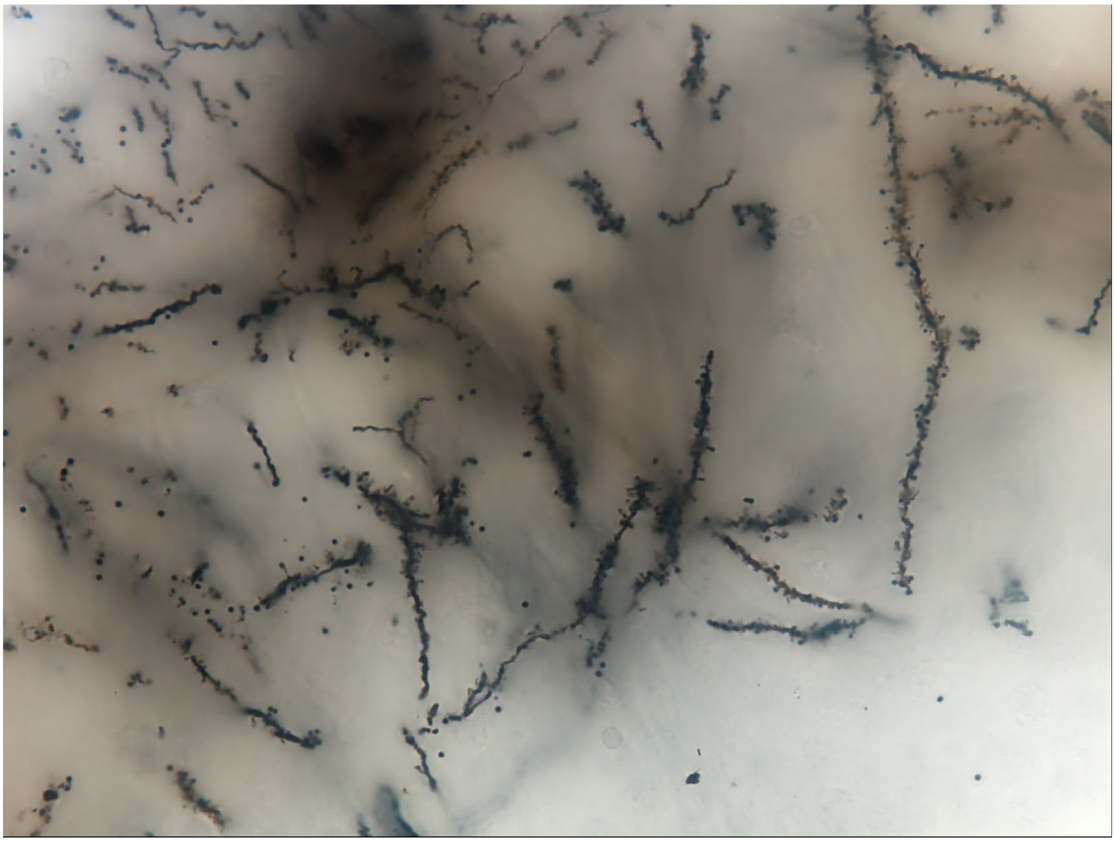

original pictures for apical spines of sham group in Figure 1H and I

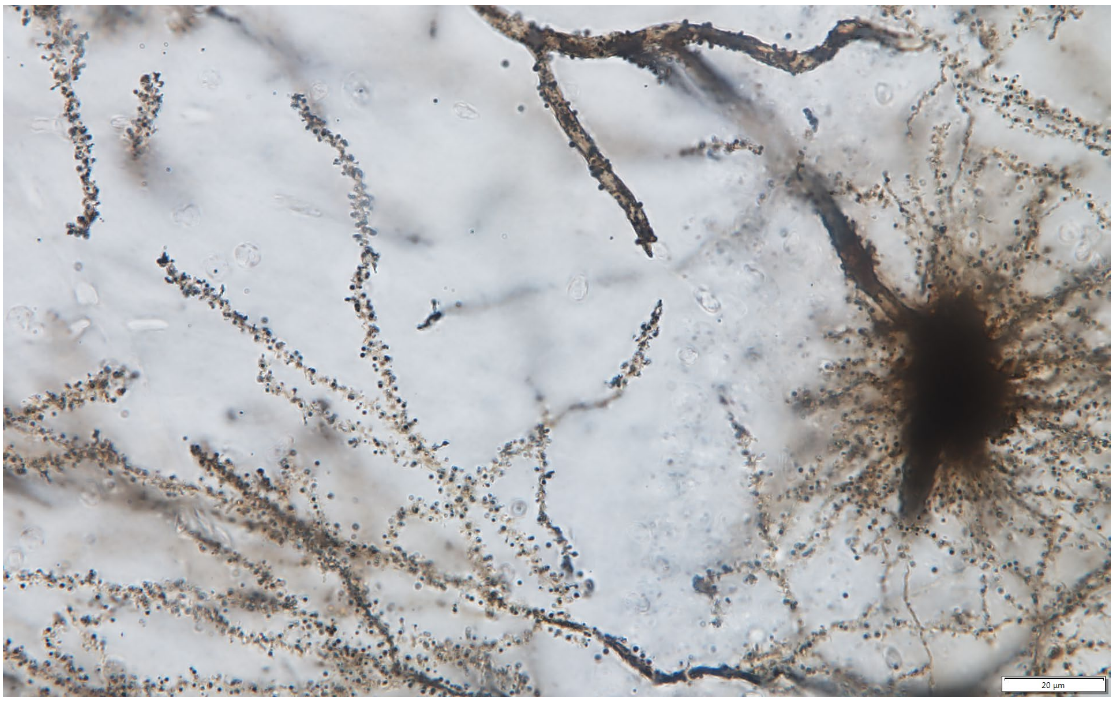

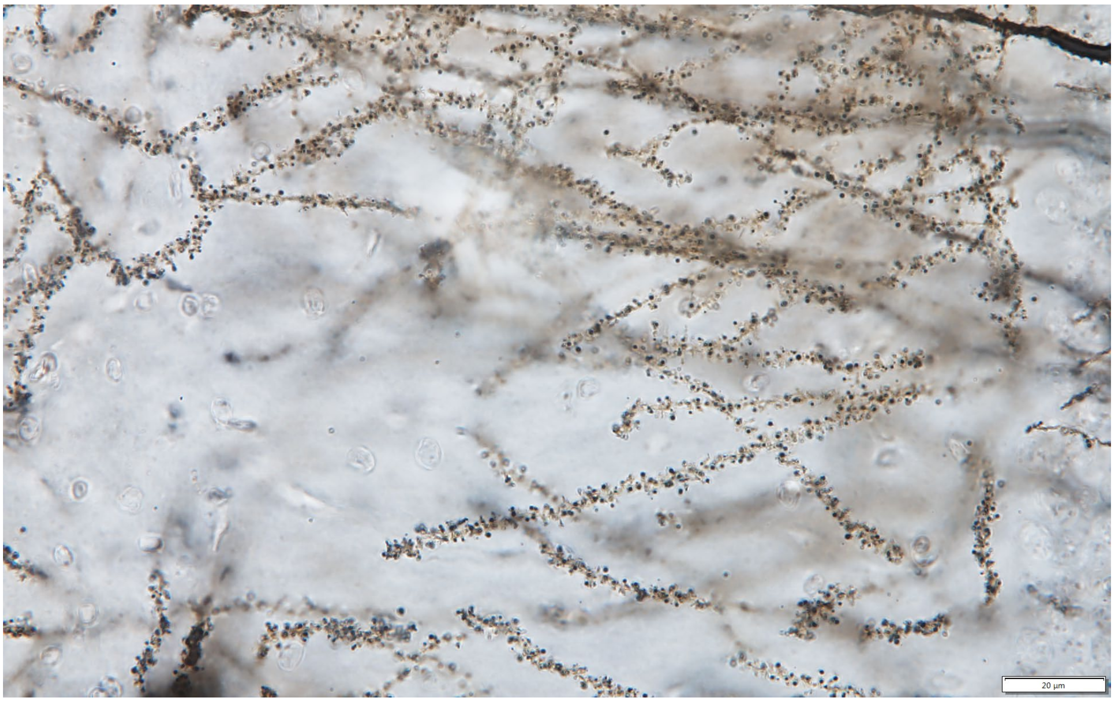

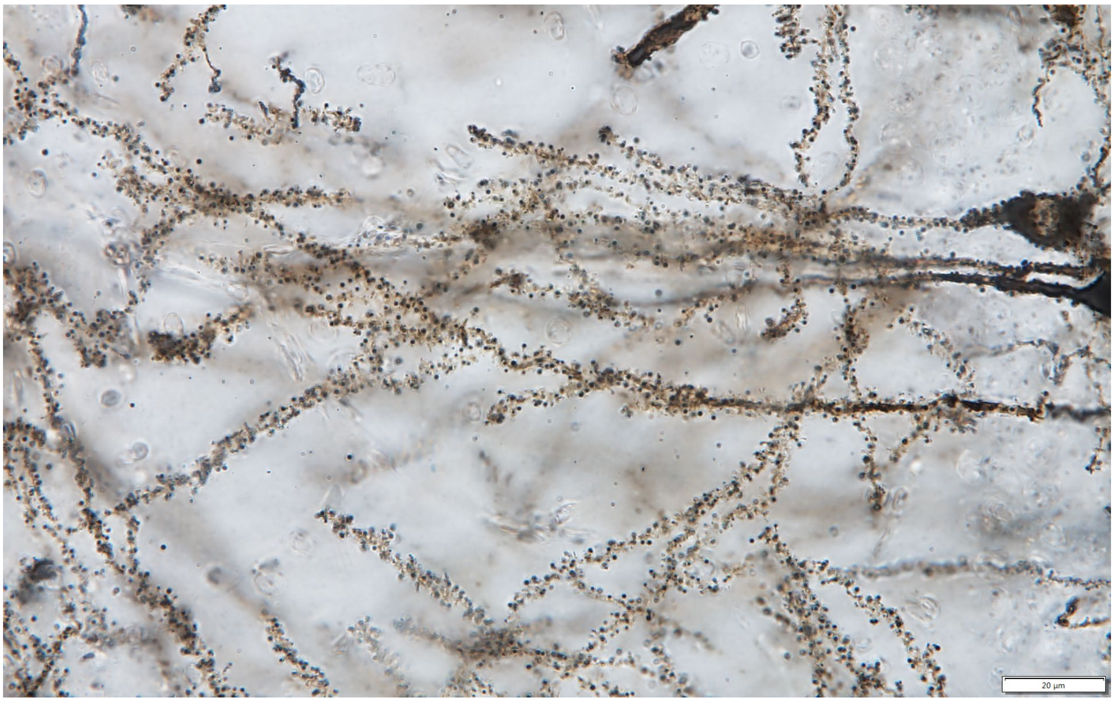
20 µm

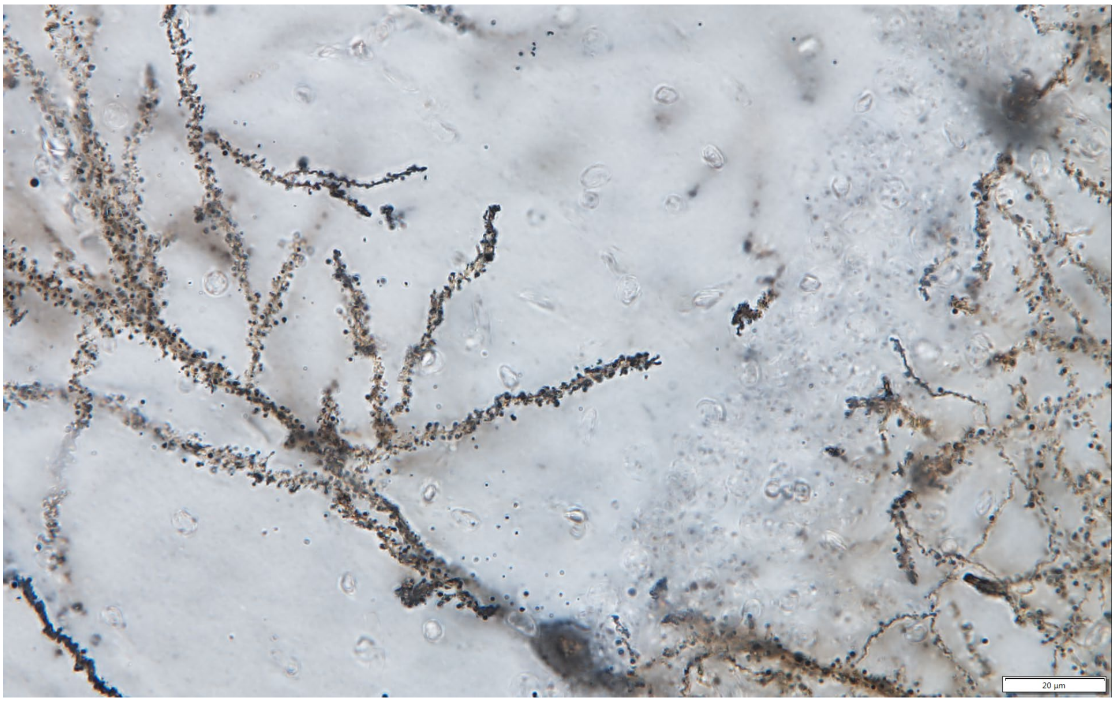
20 µm

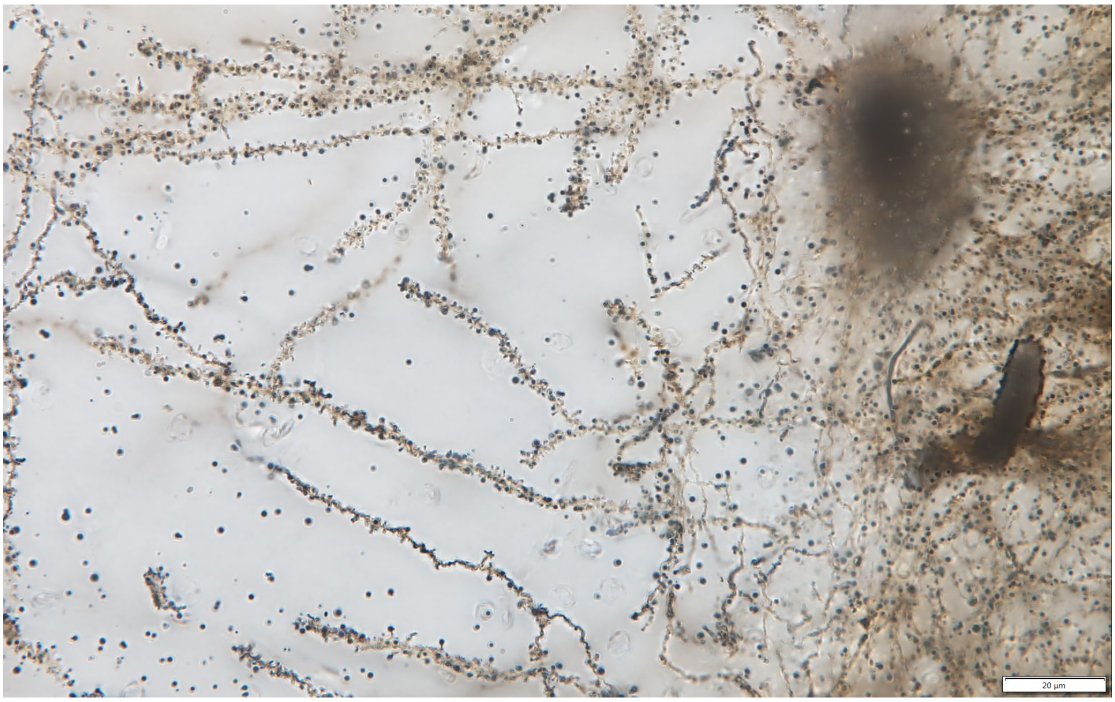

original pictures for basal spines of sham group in Figure 1H and I

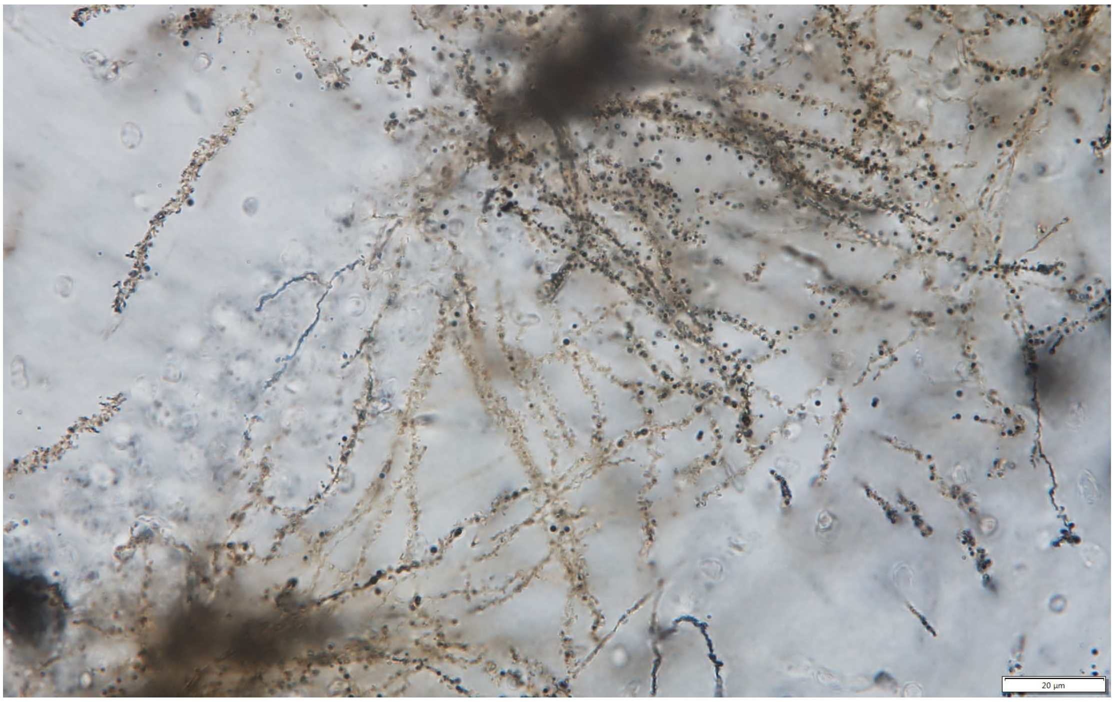

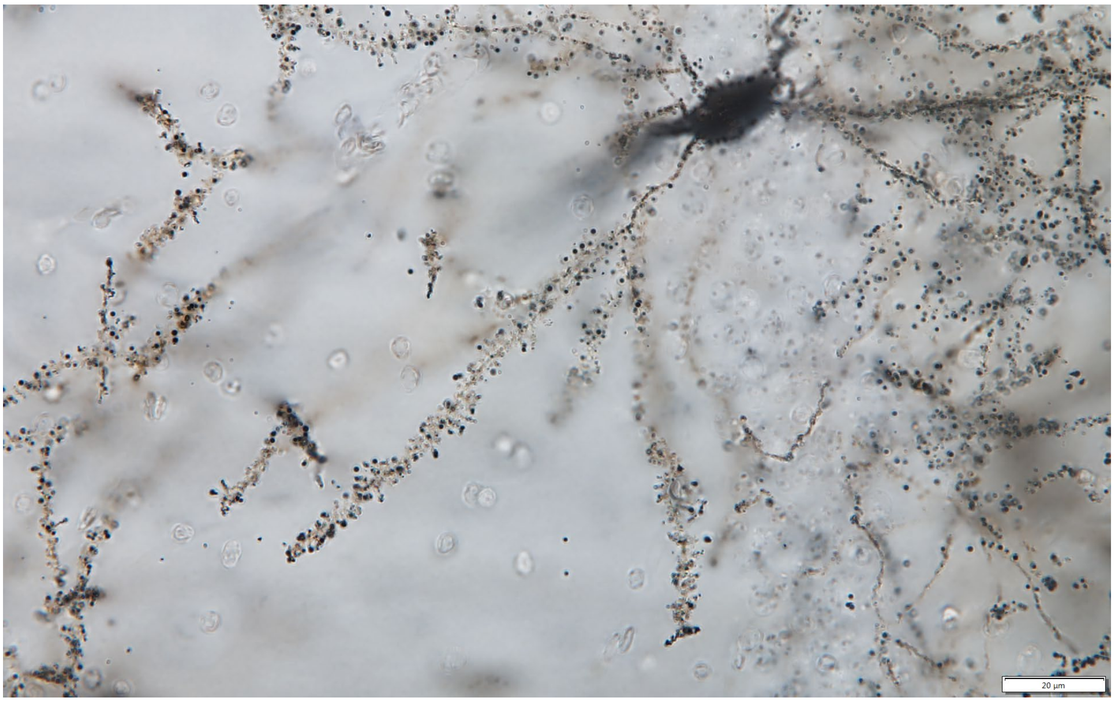
20 µm

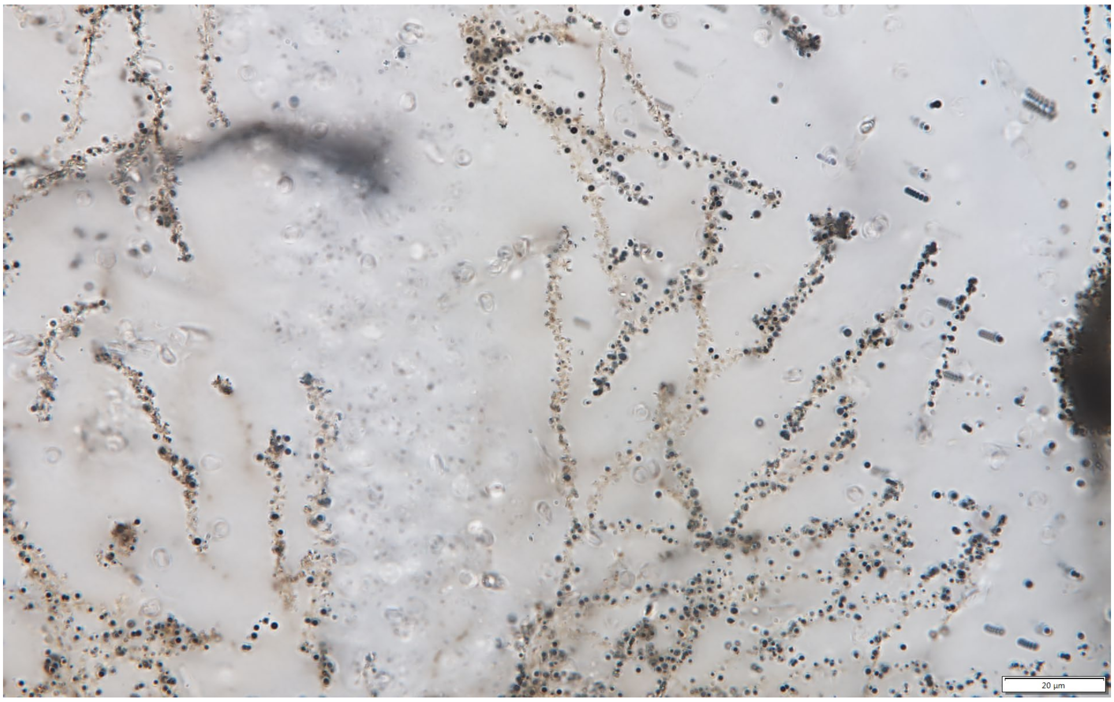
20 µm

original pictures for Figure 2M

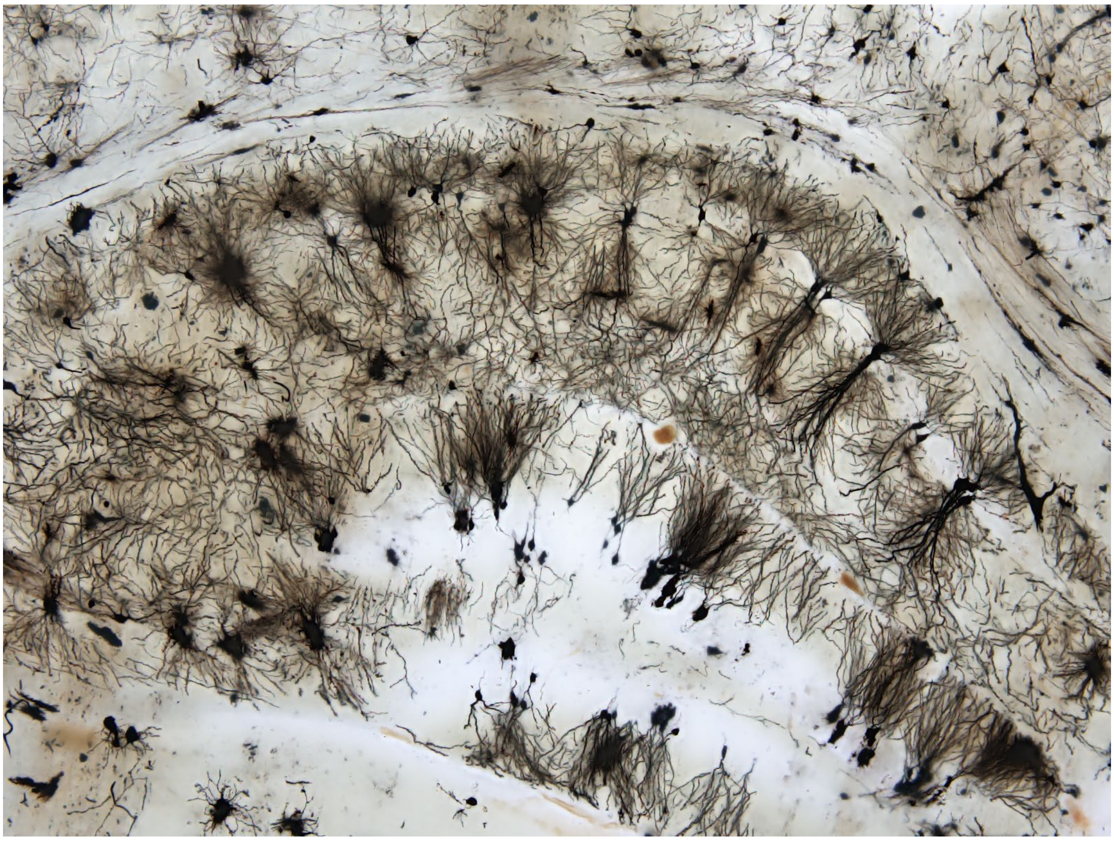

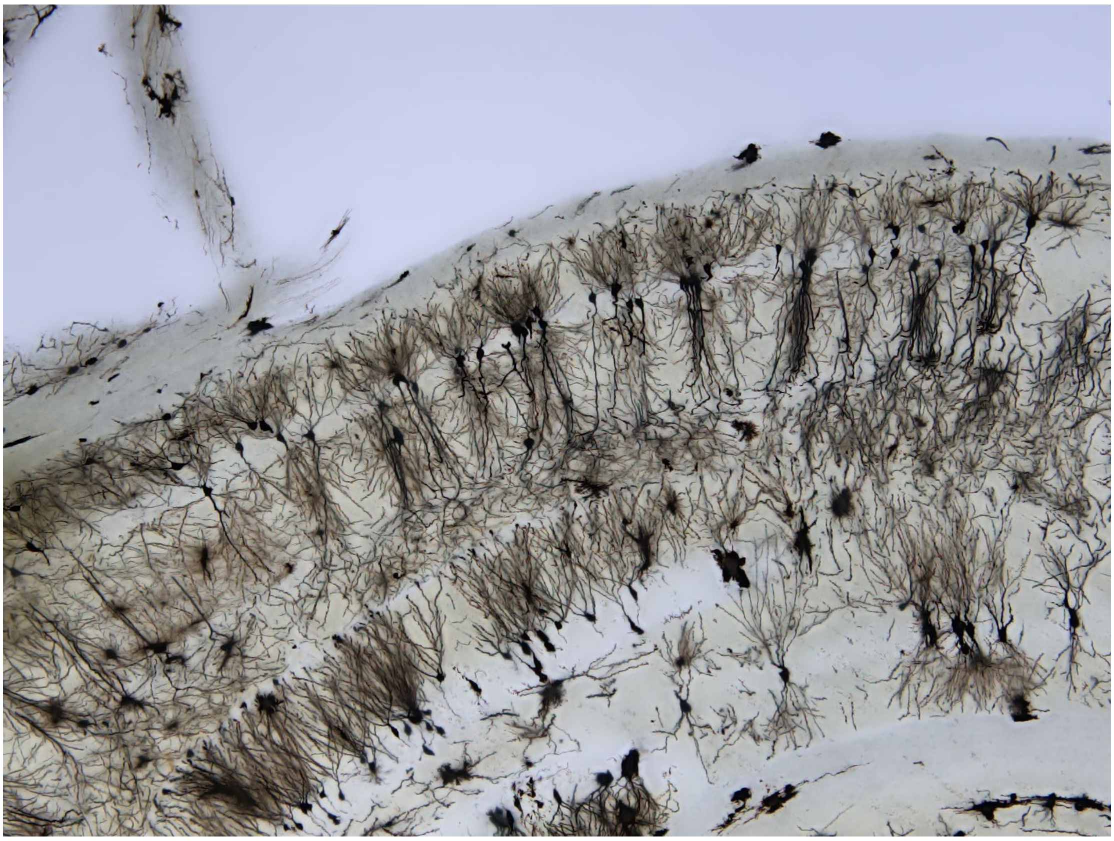

original pictures for sham group in Figure 2N

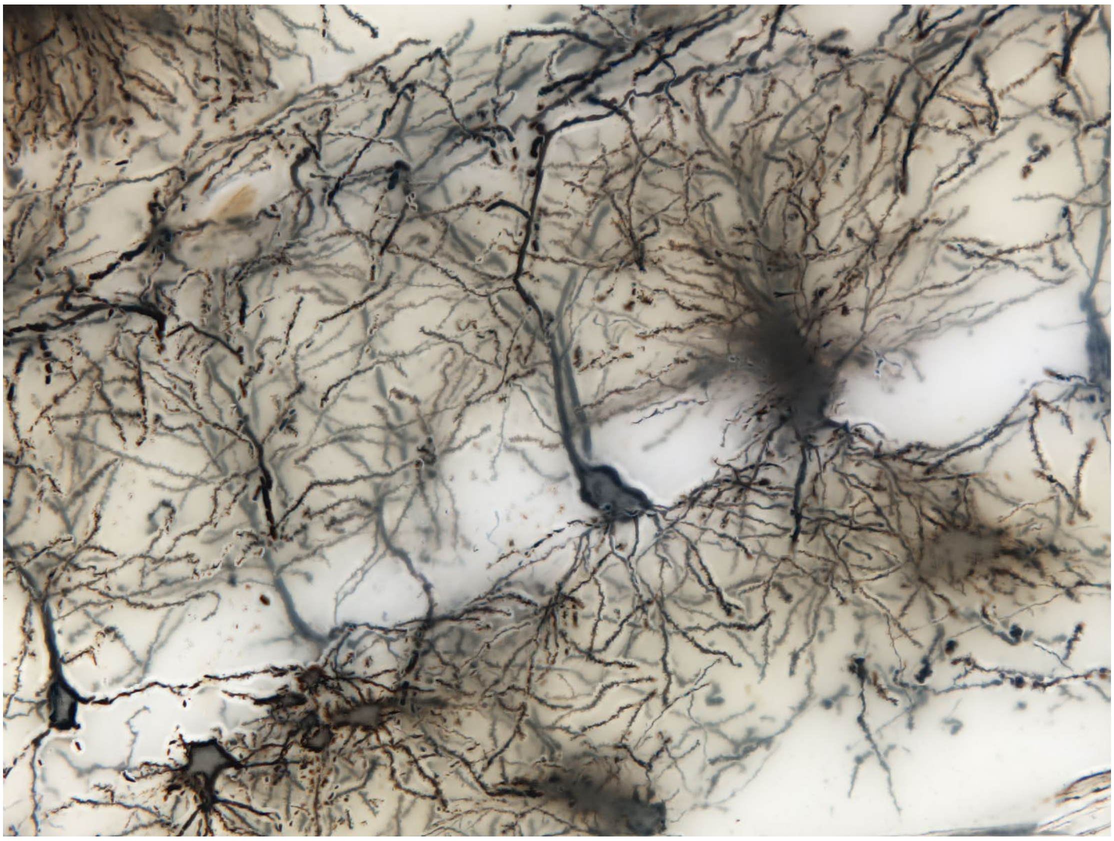

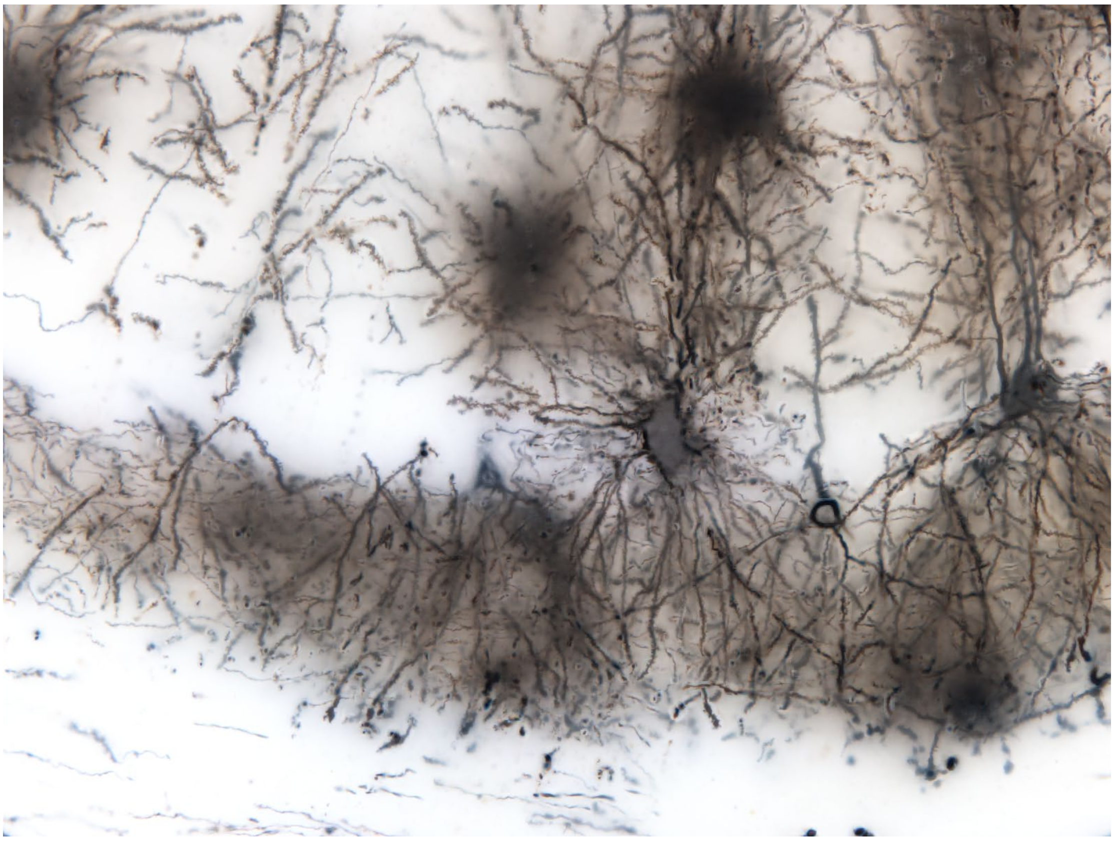

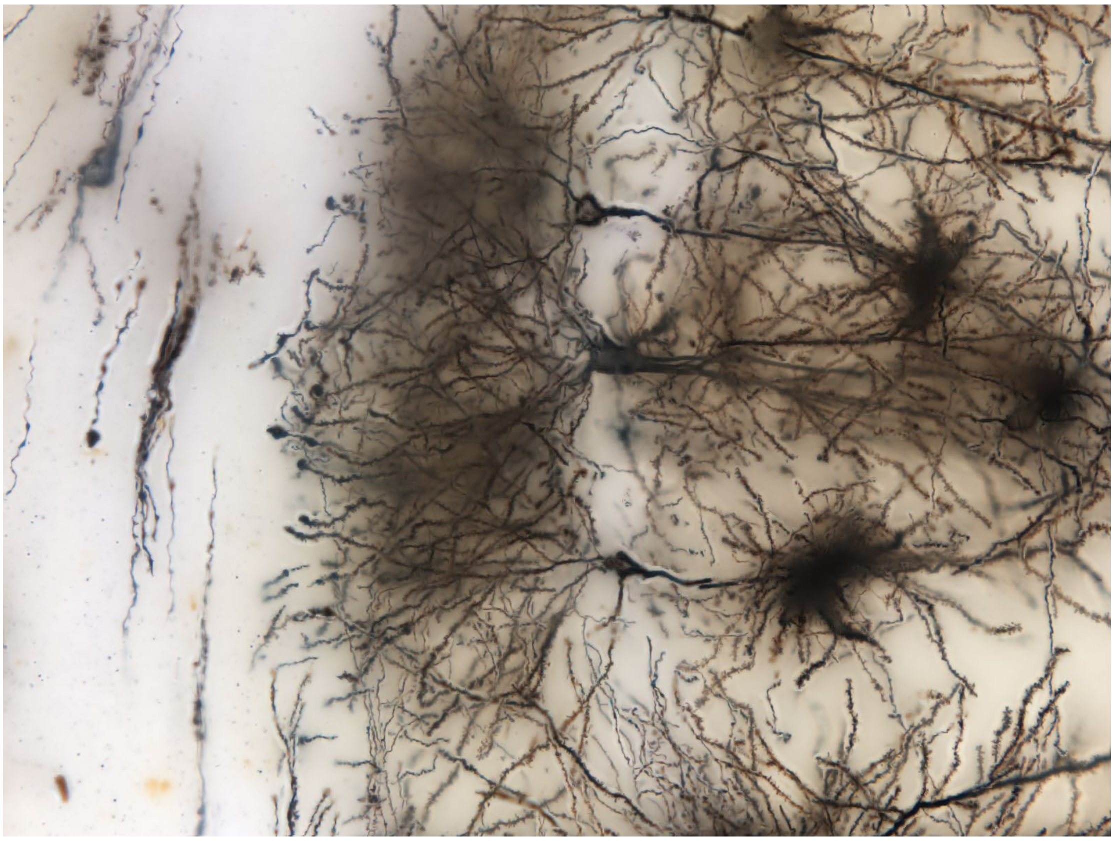

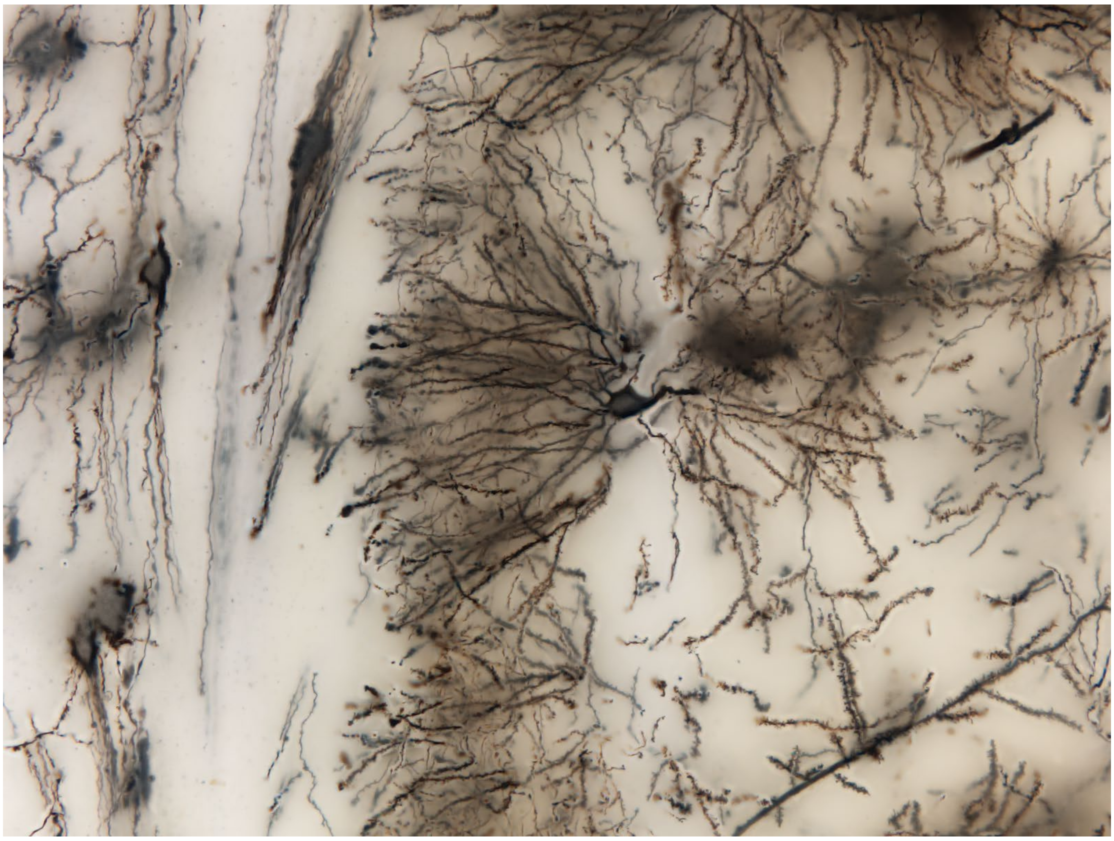

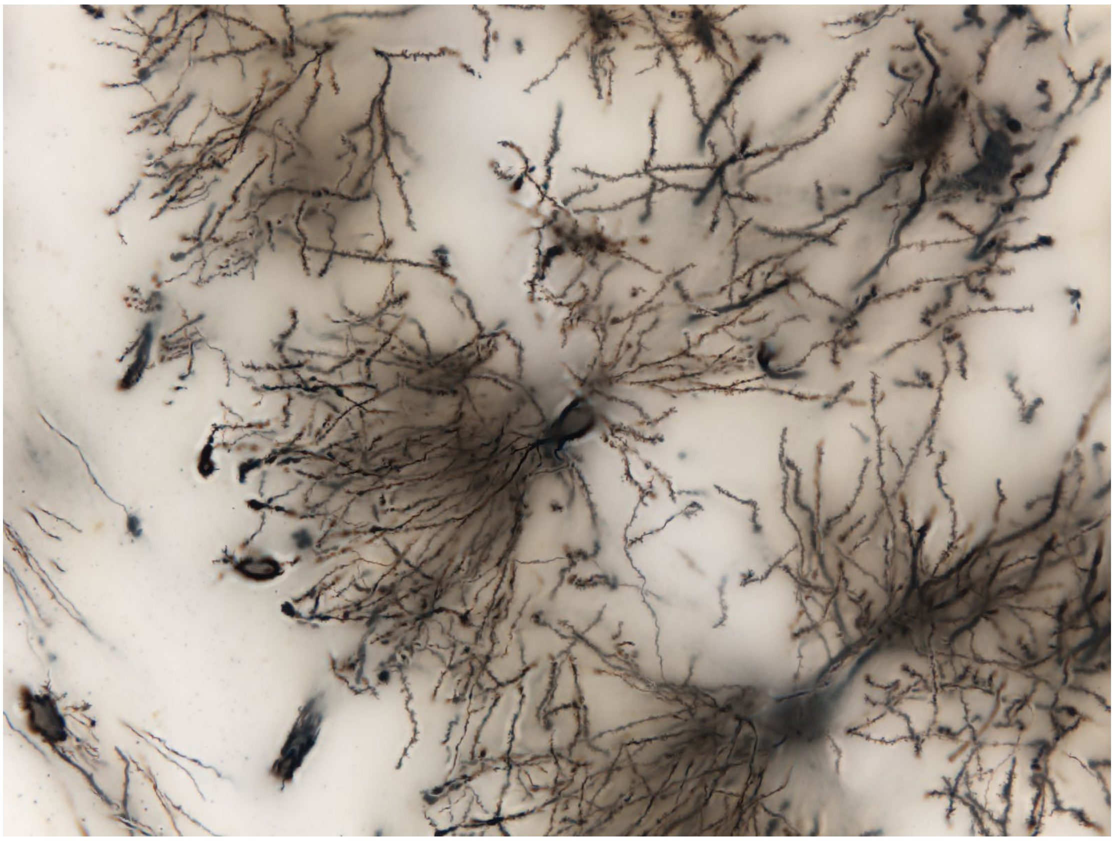

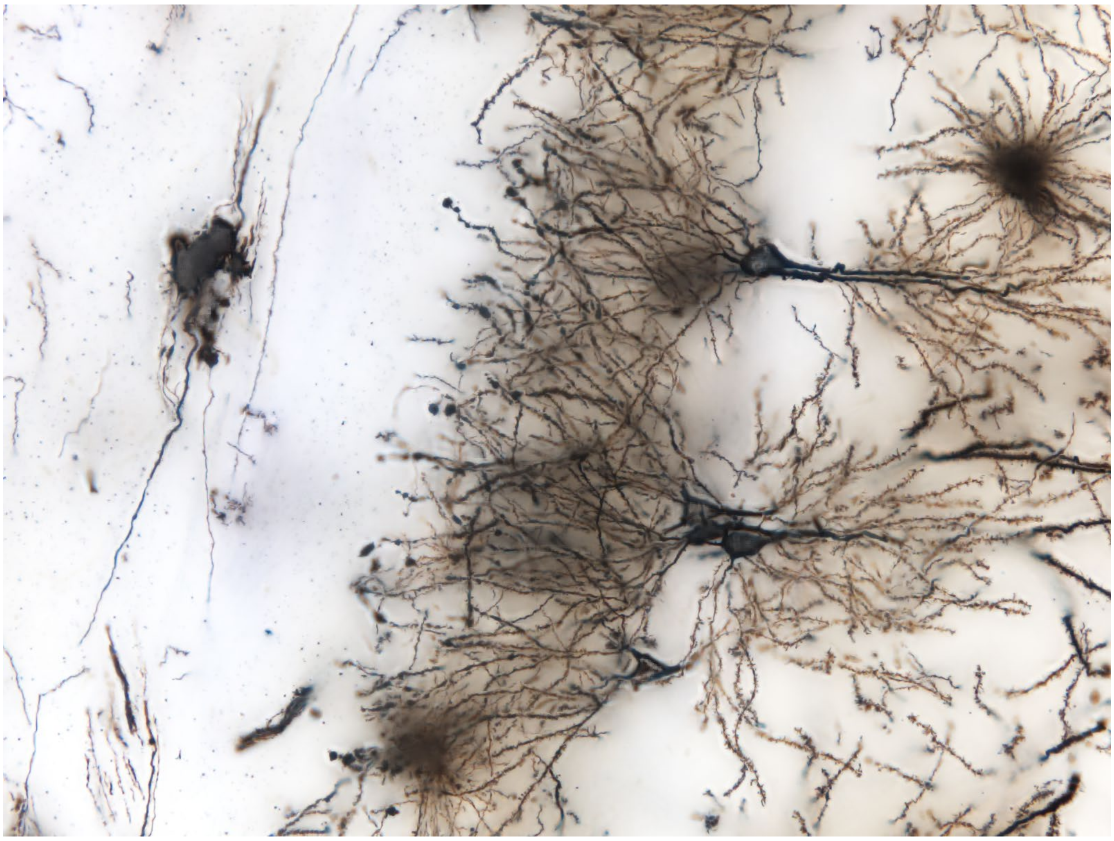

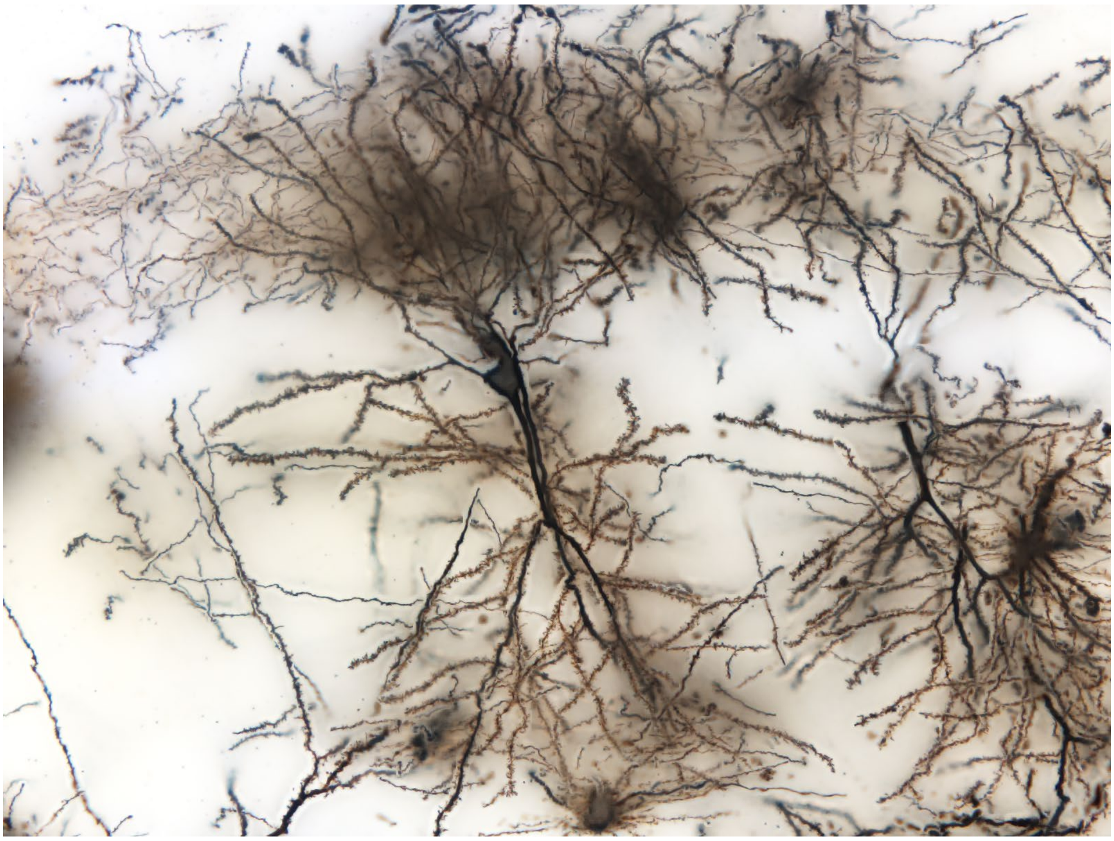

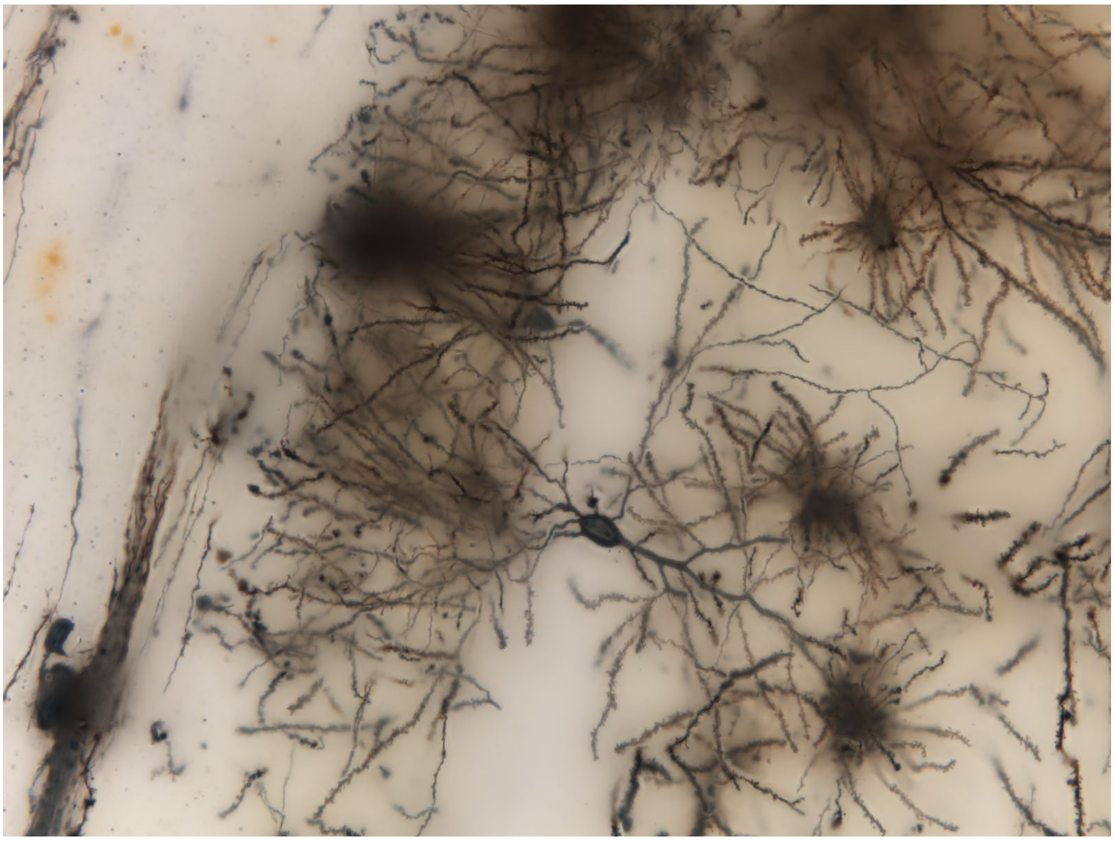

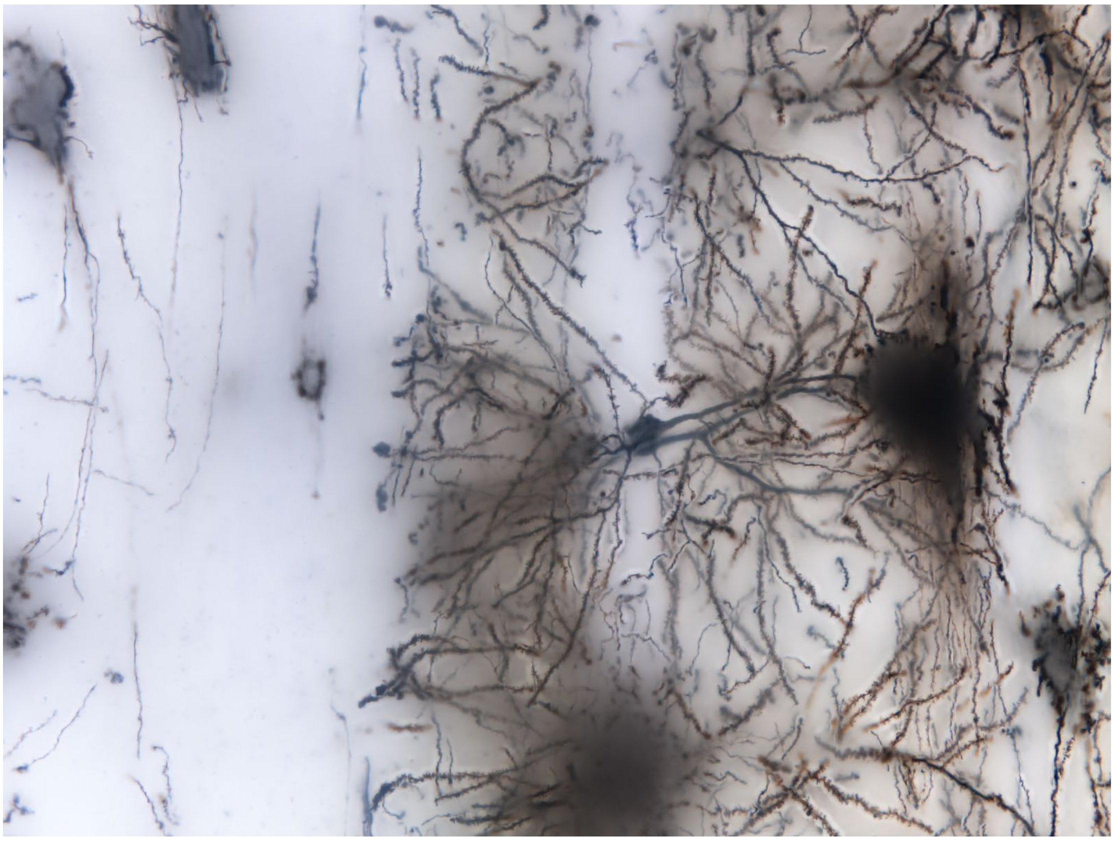

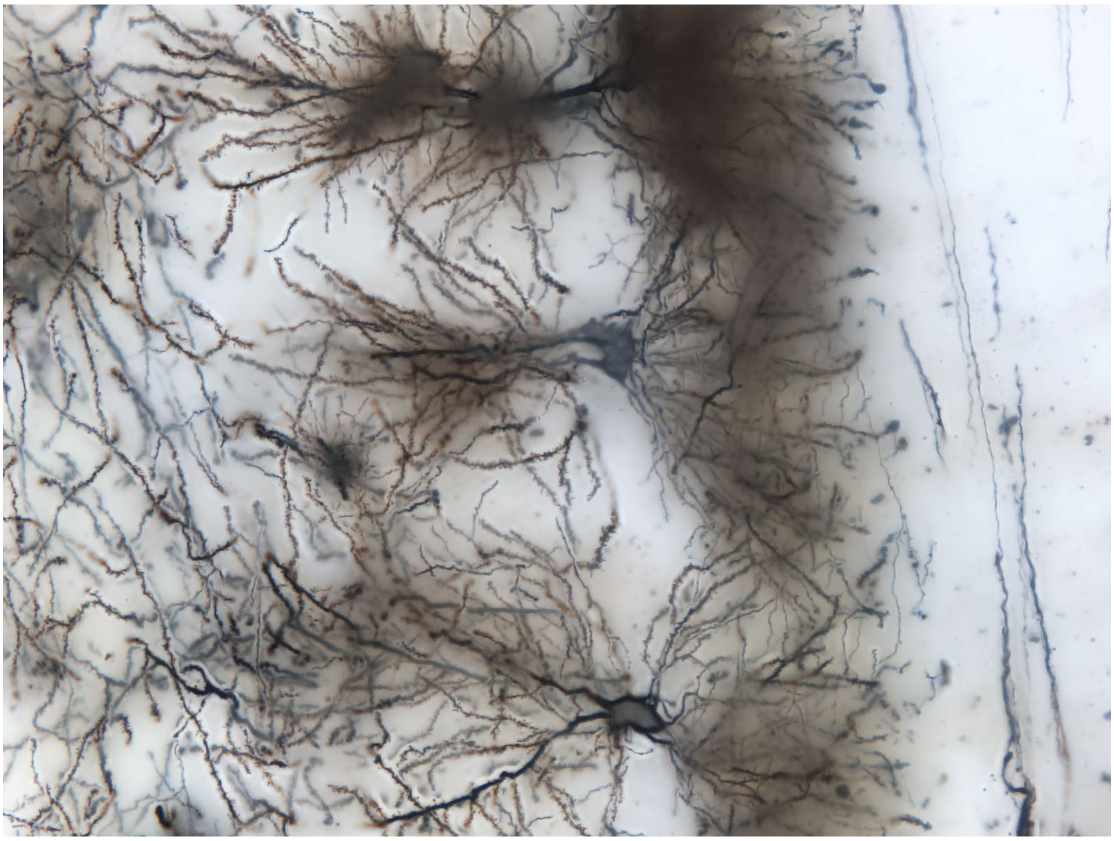

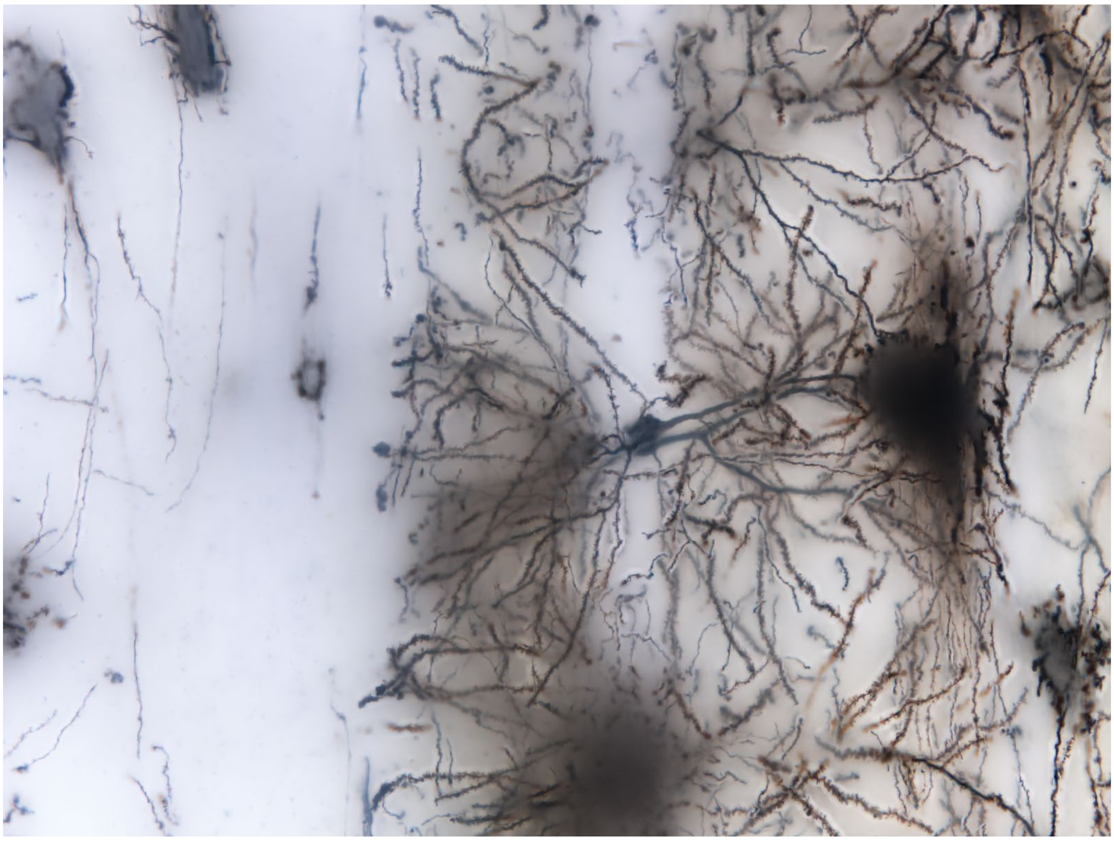

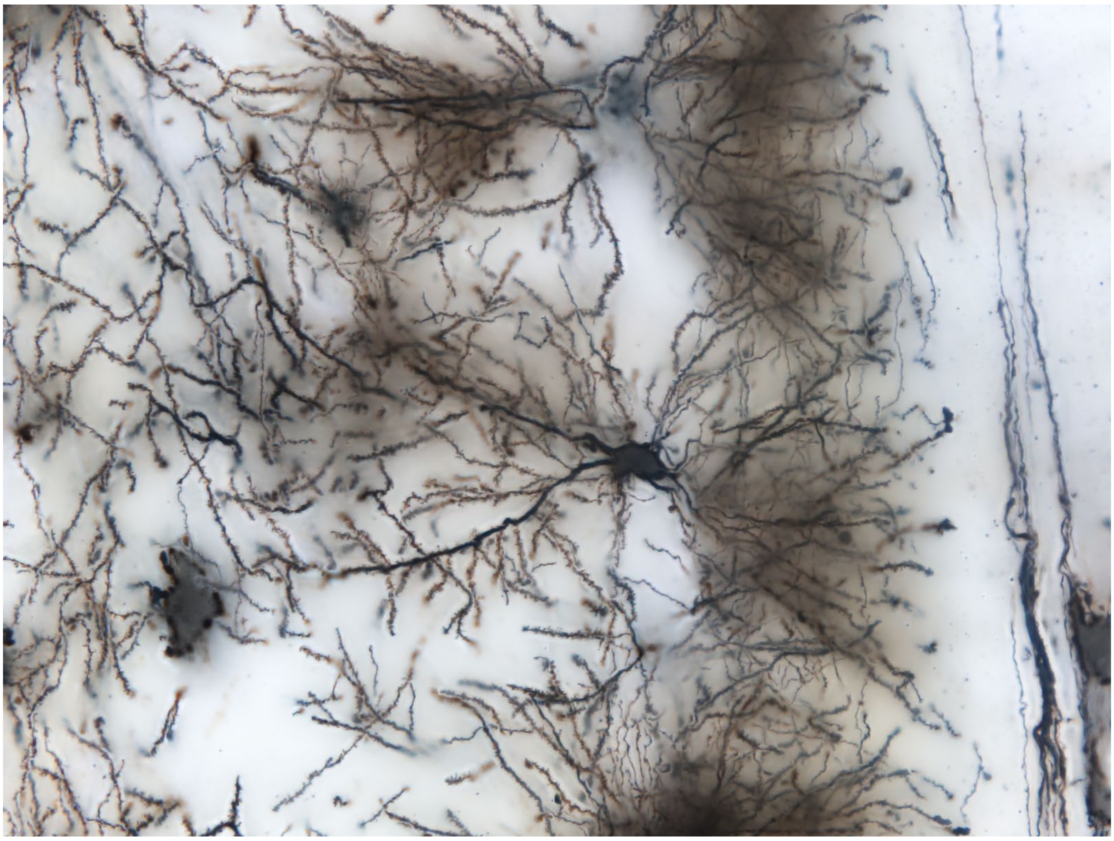

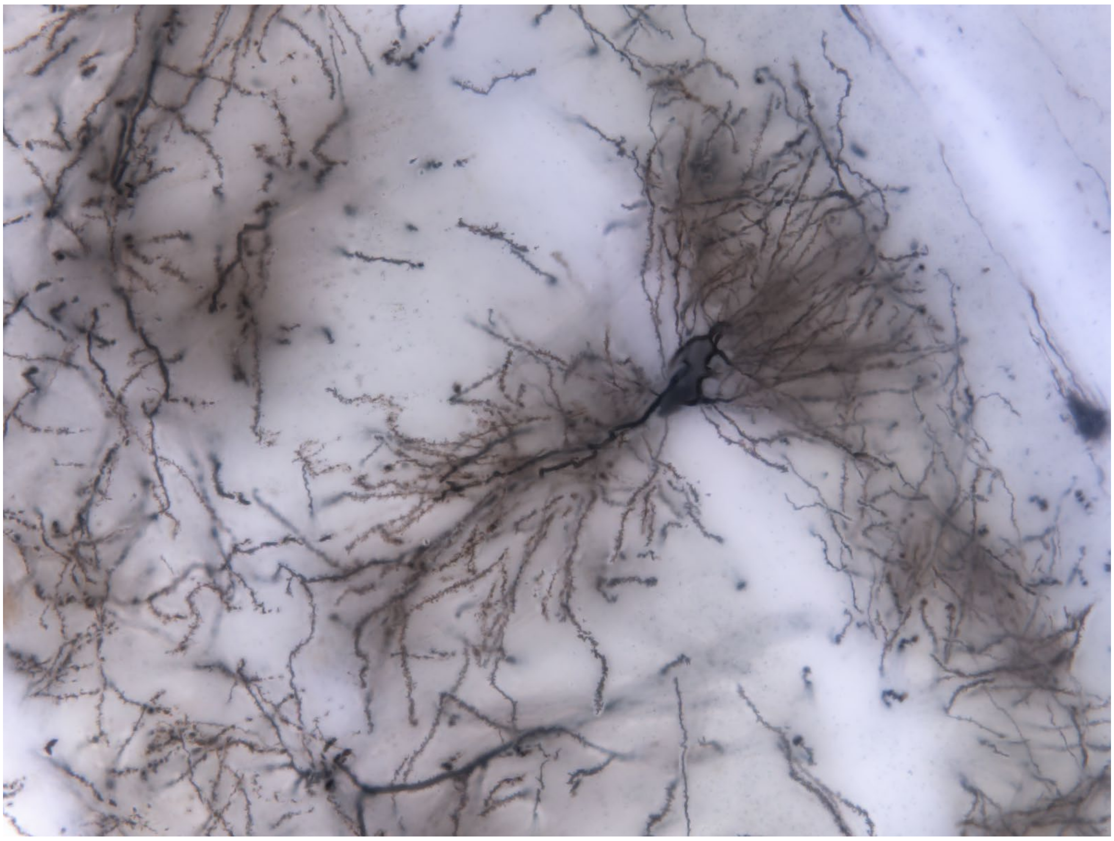

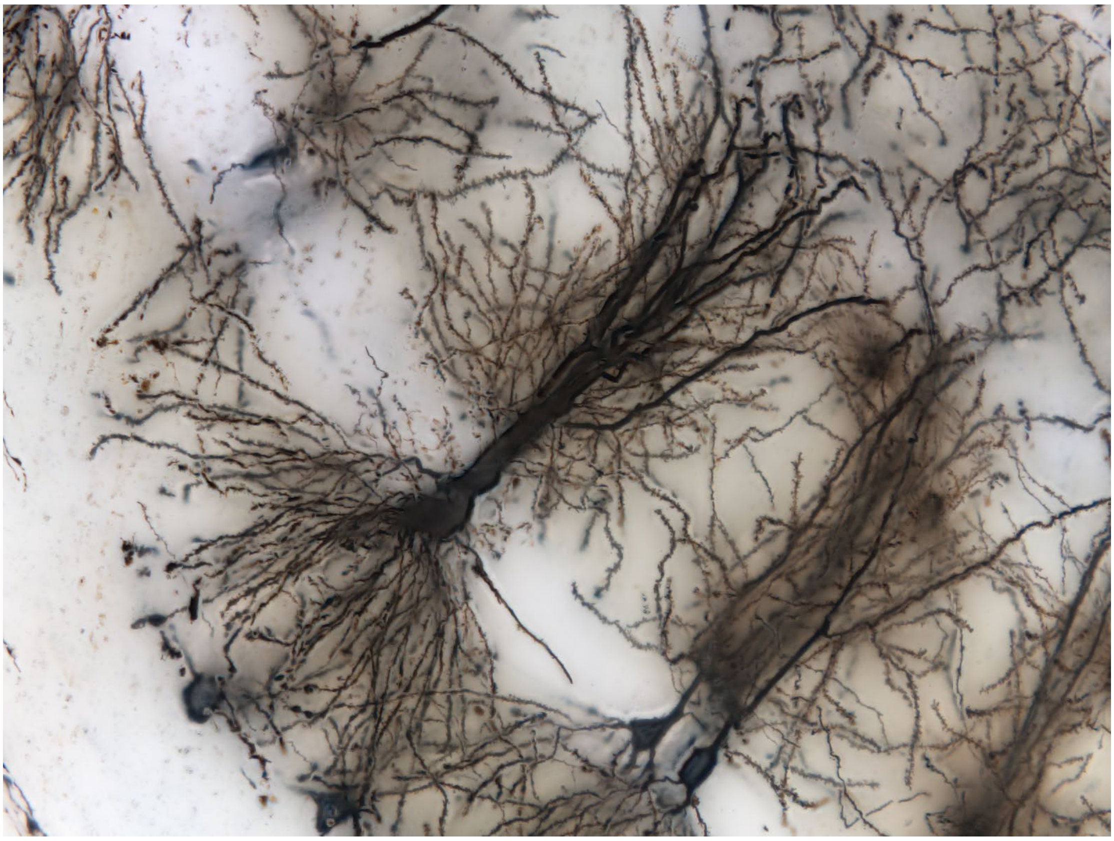

original pictures for AIM2-OE group in Figure 2N

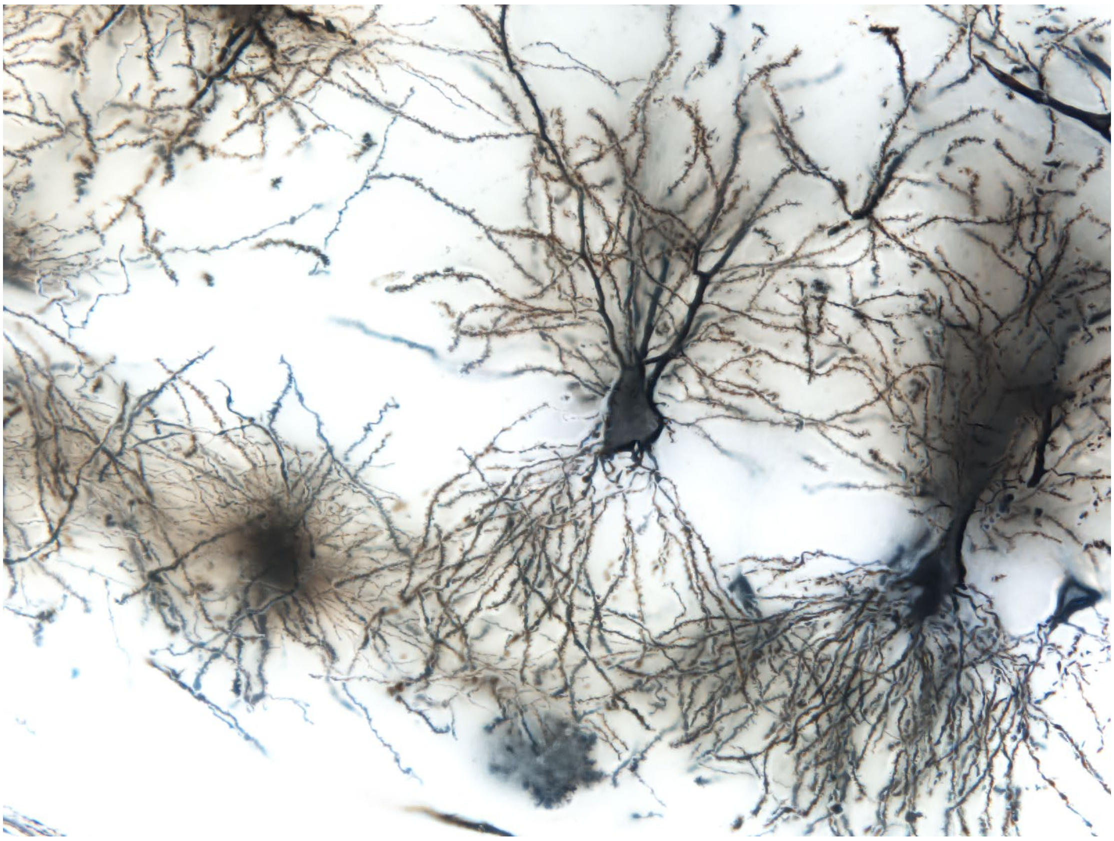

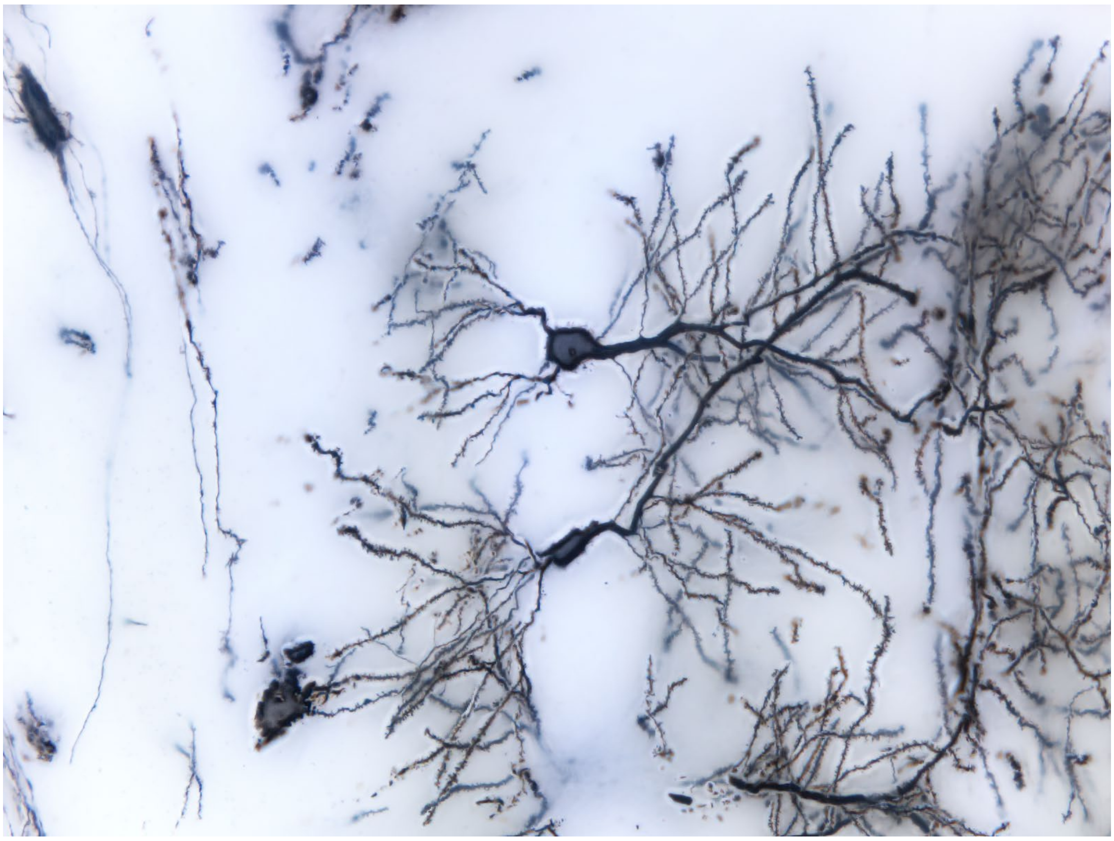

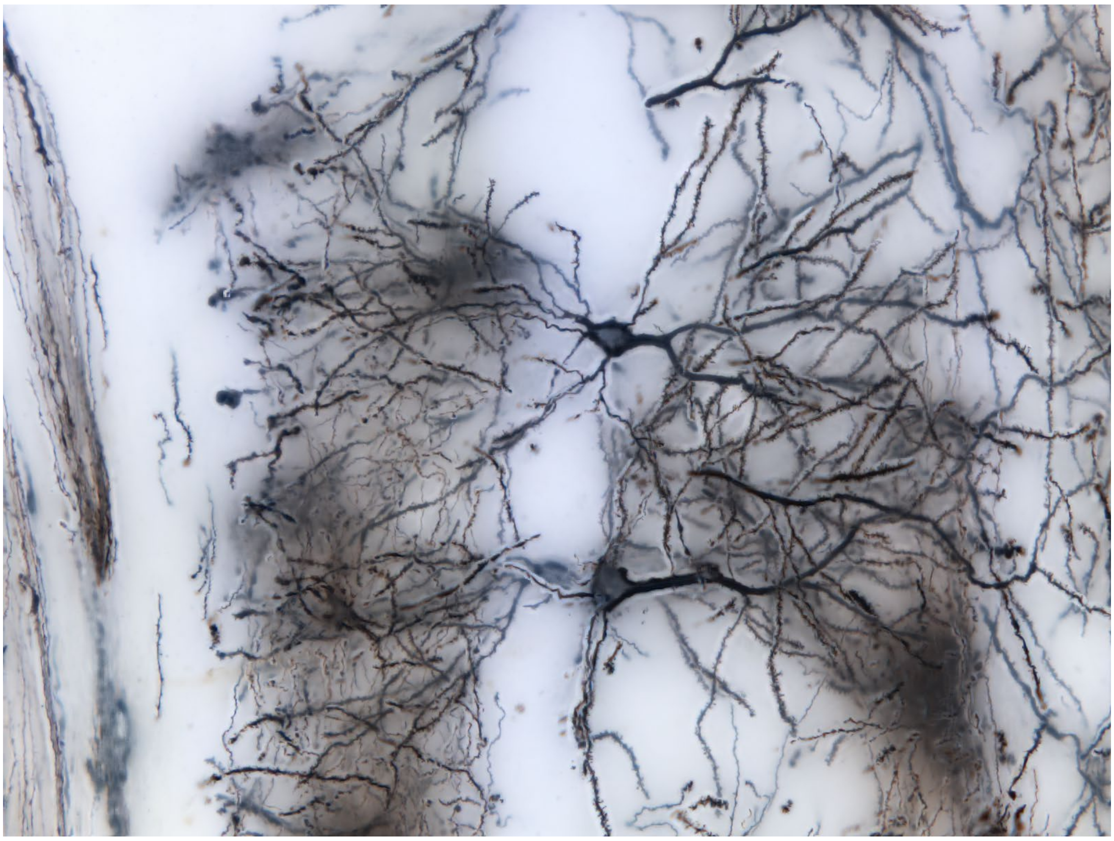

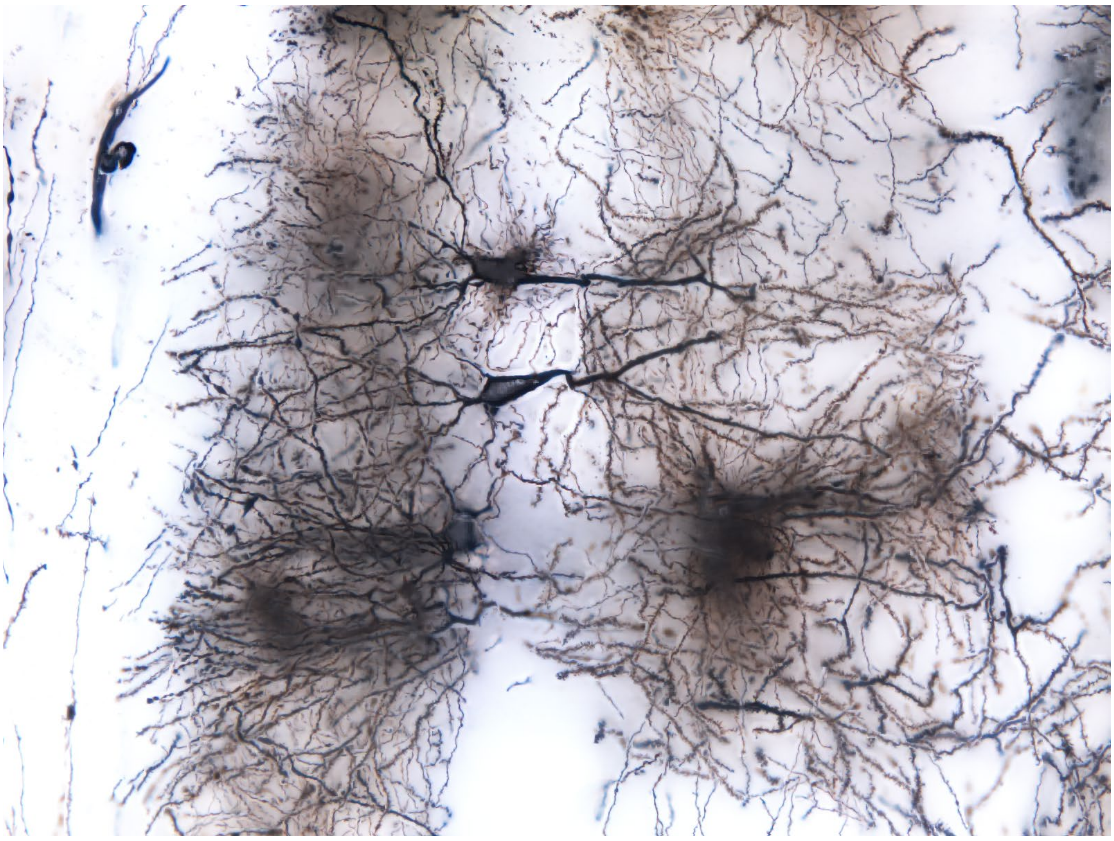

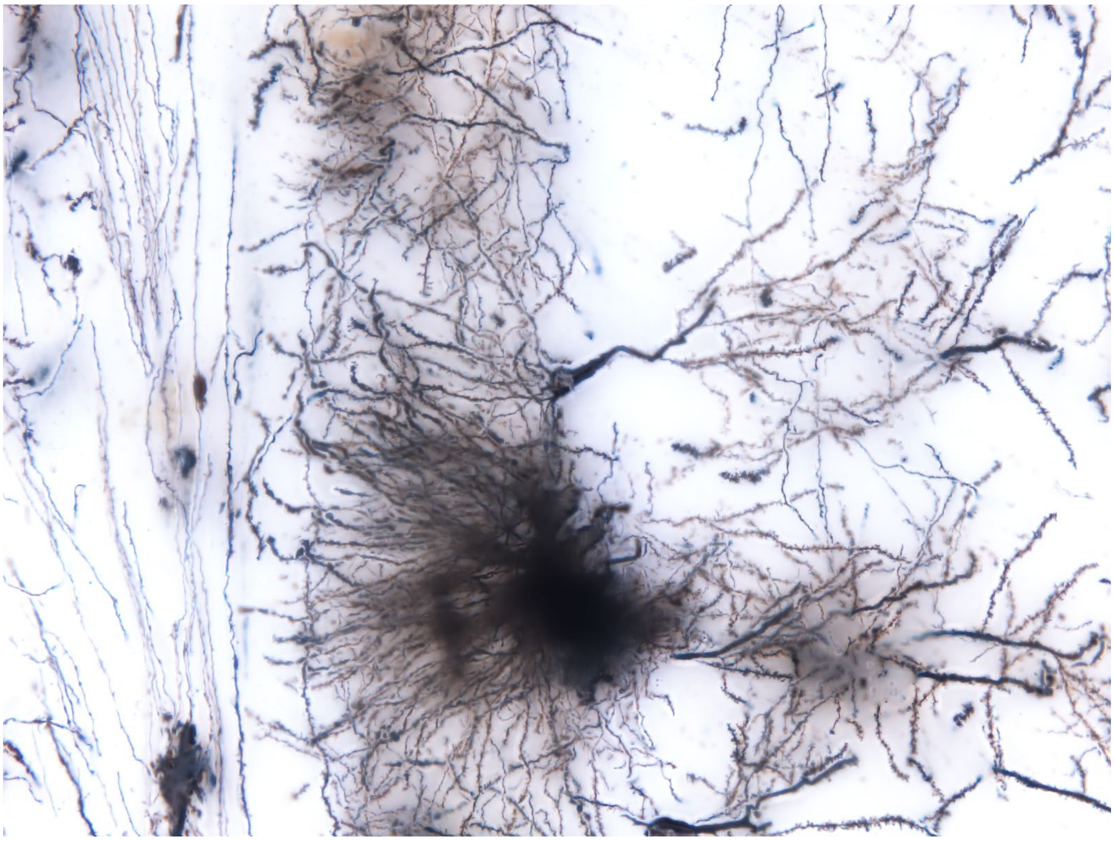

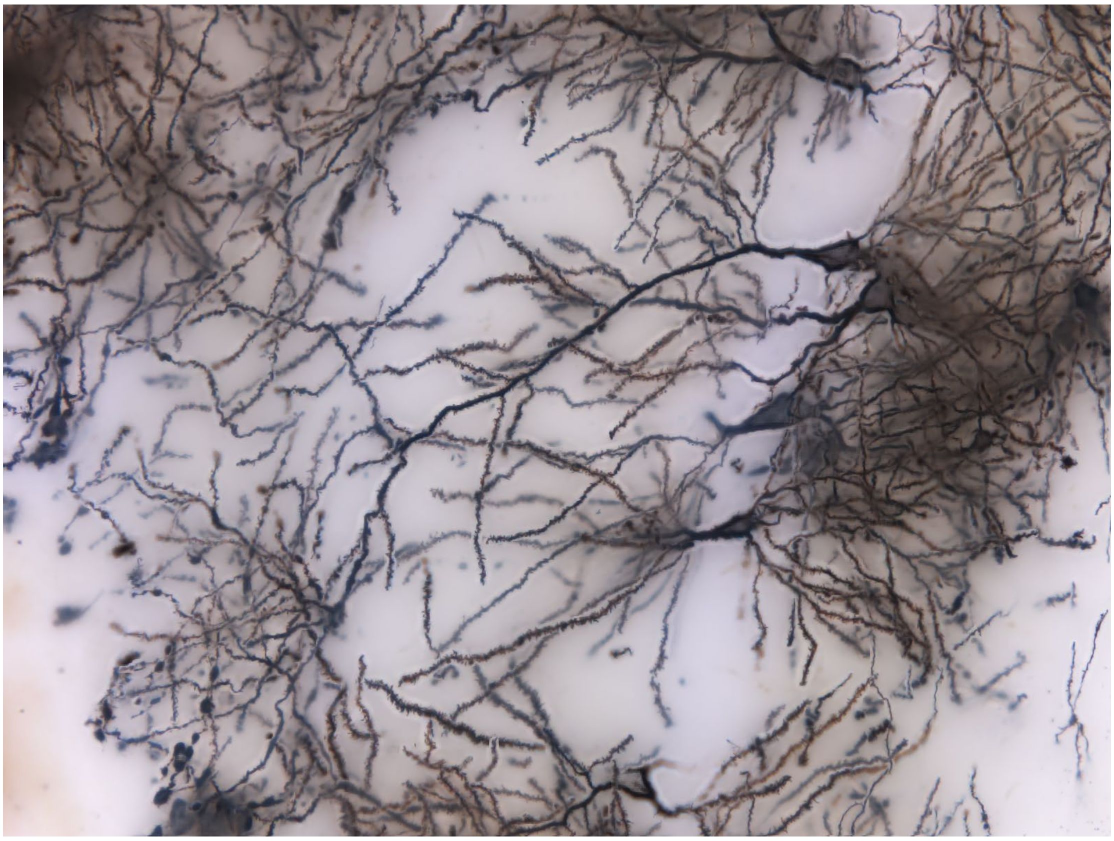

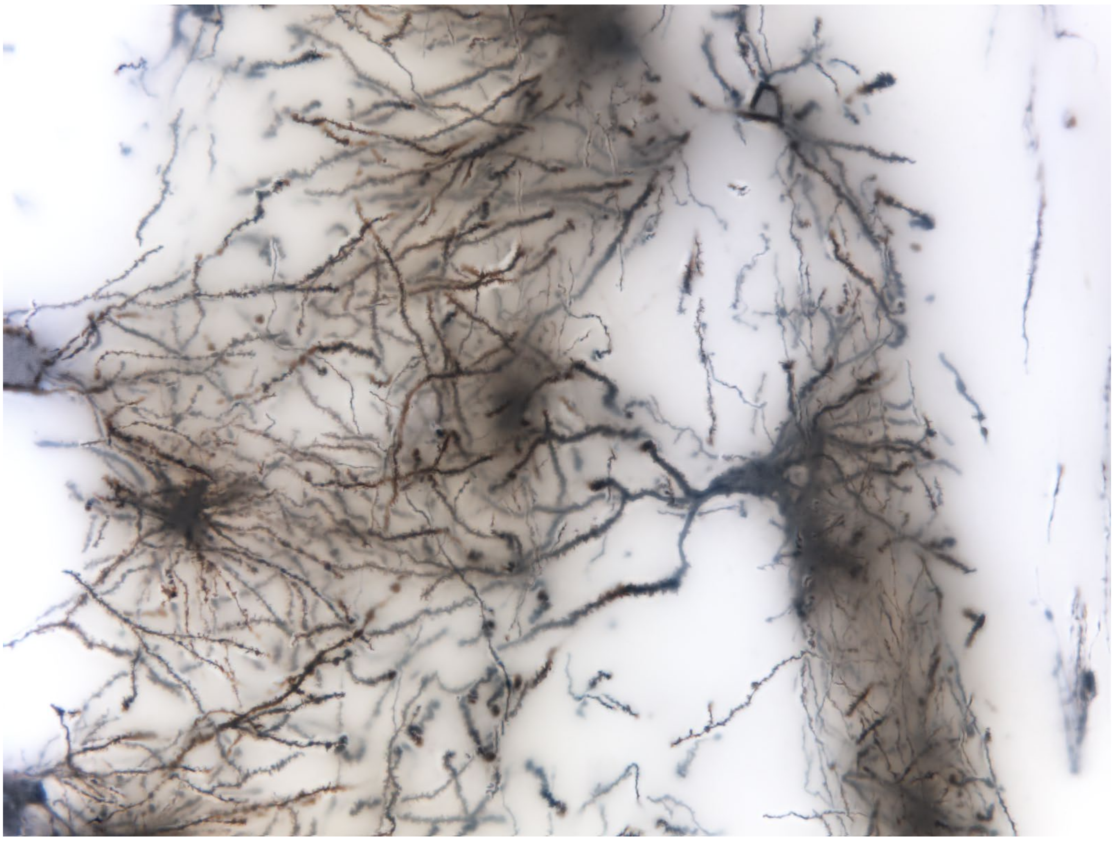

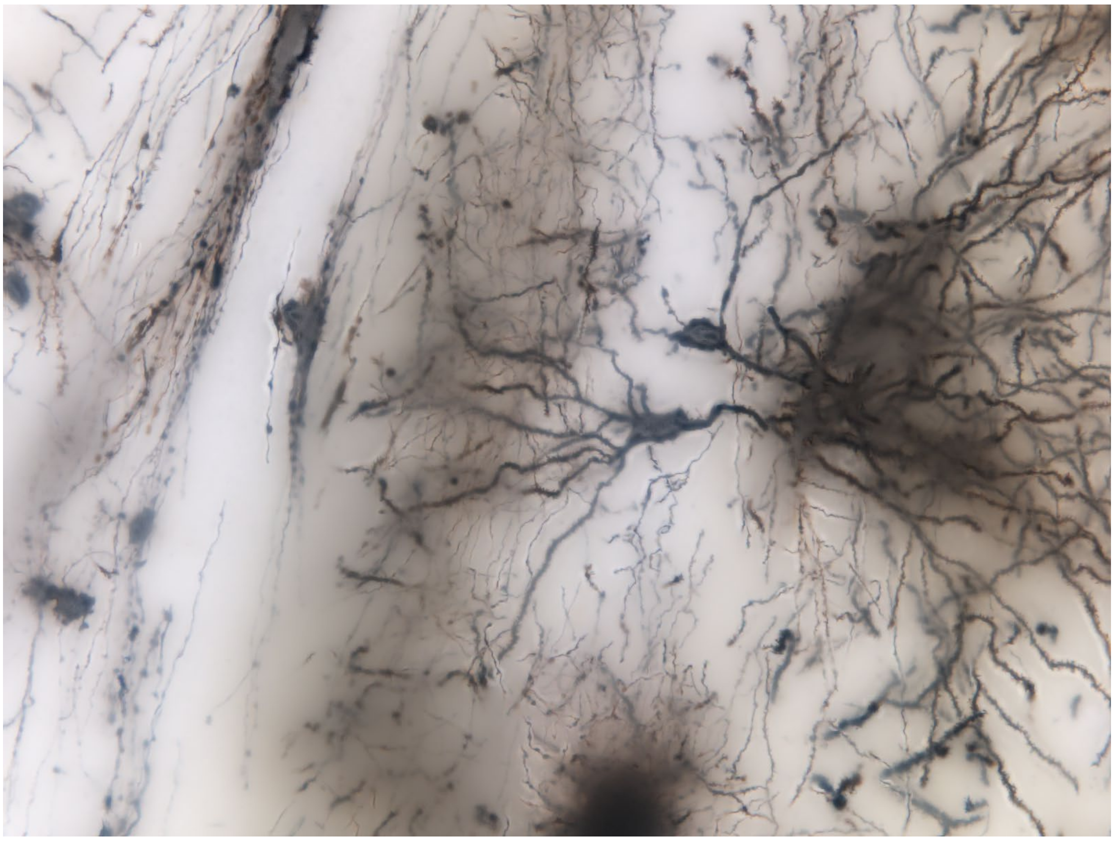

original pictures for apical spines of control group in Figure 2P and Q

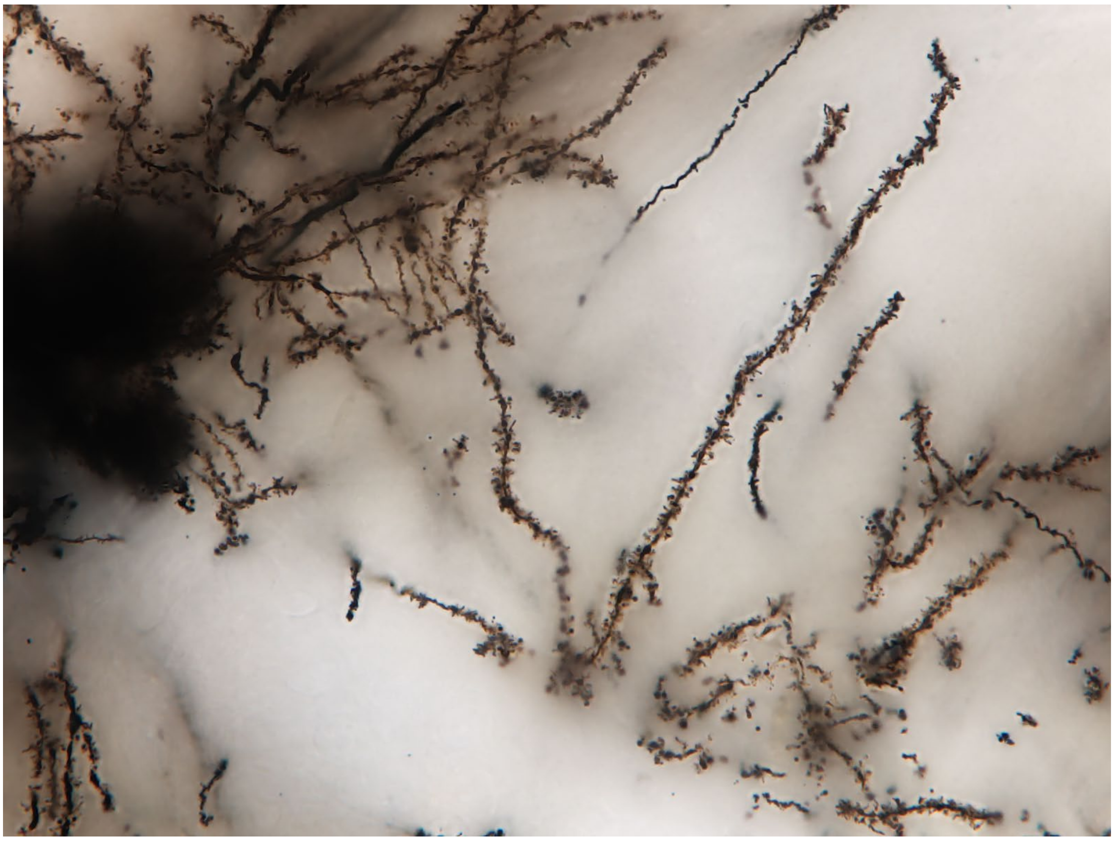

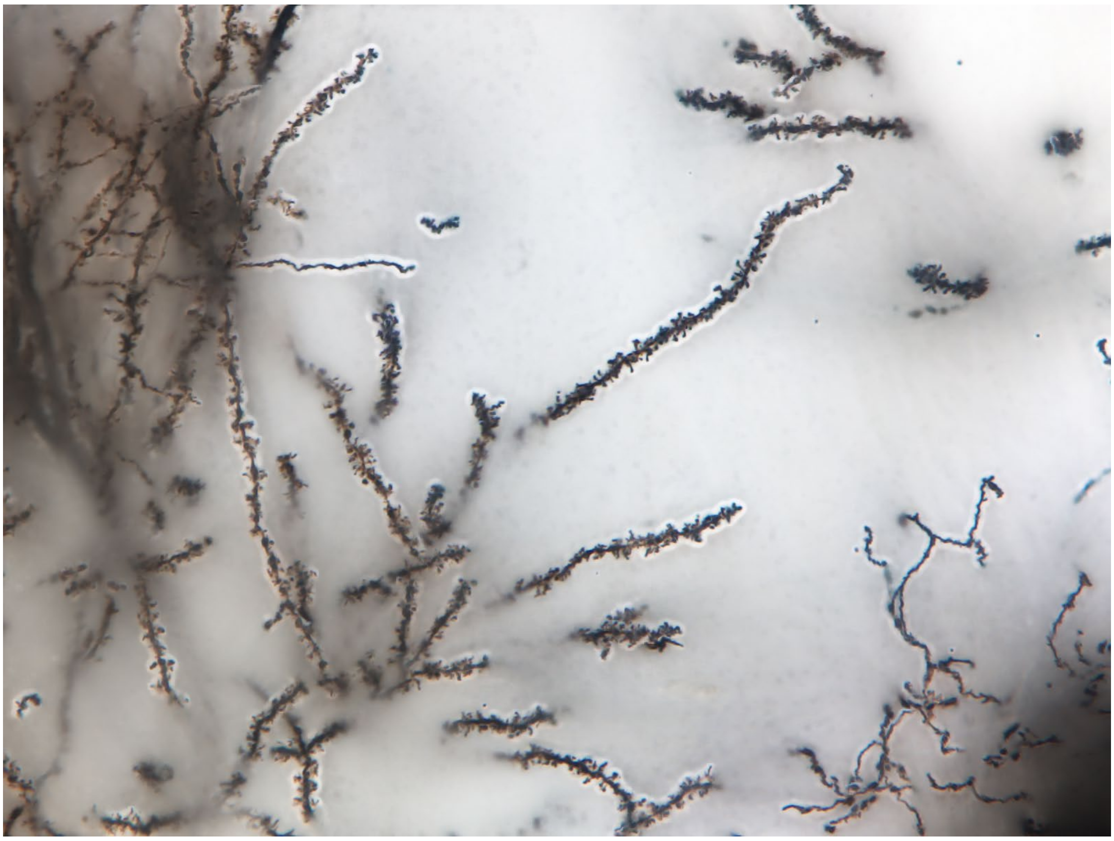

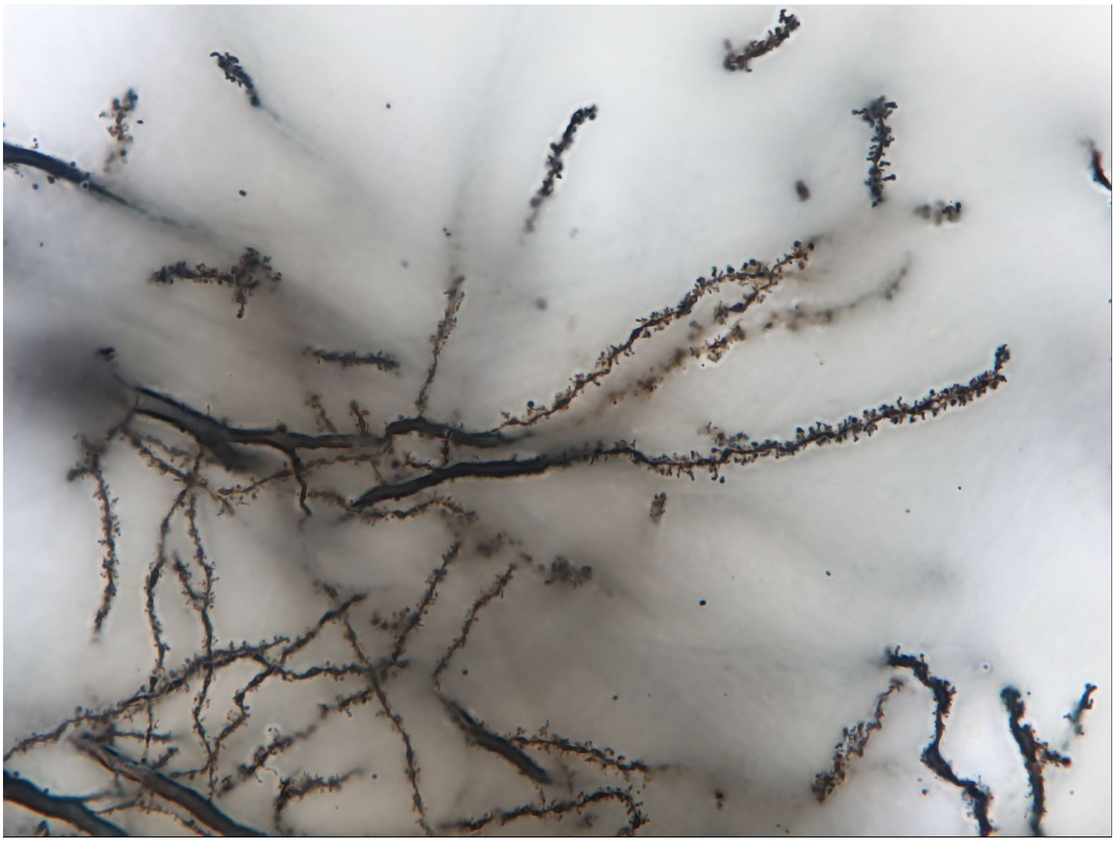

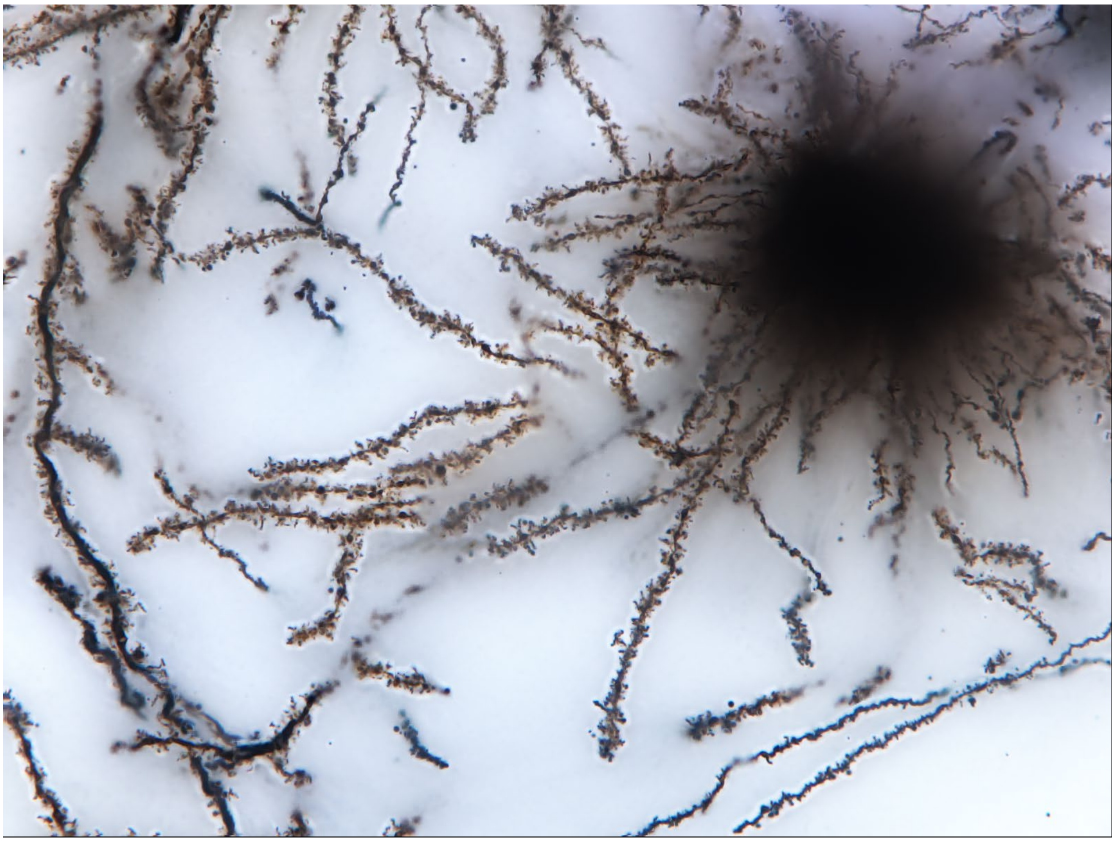

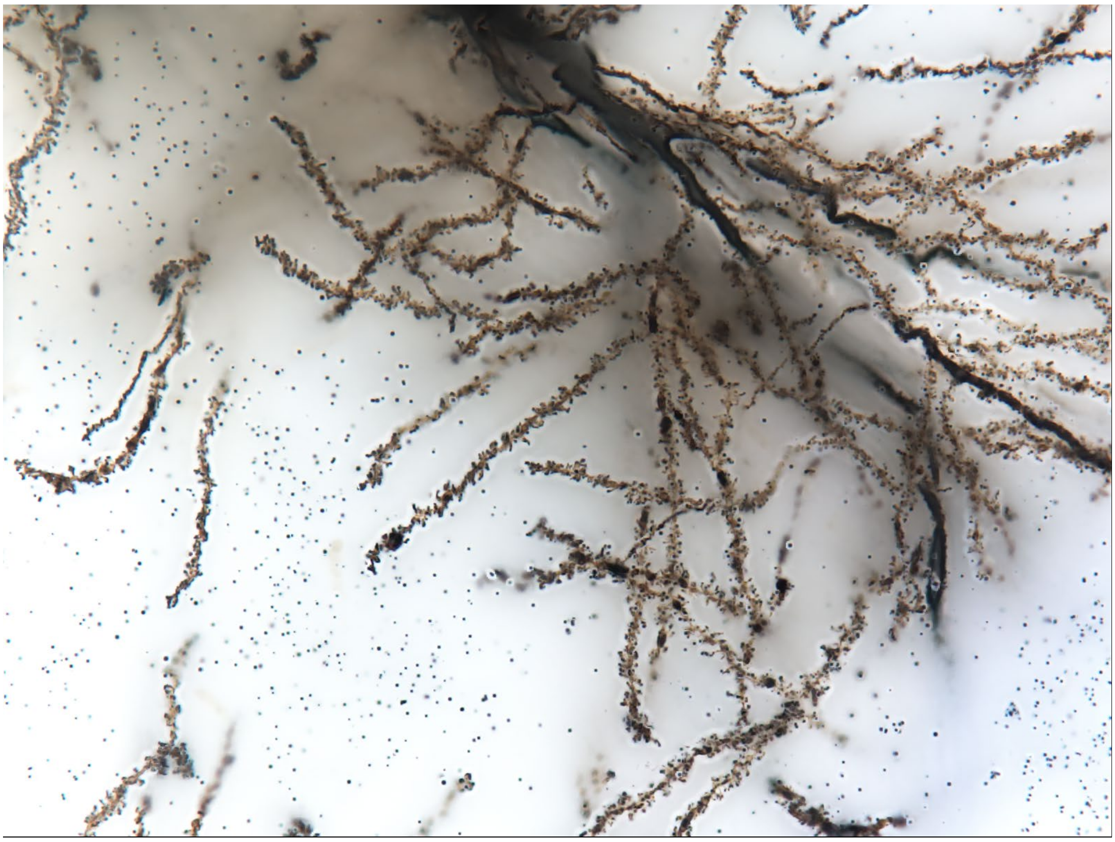

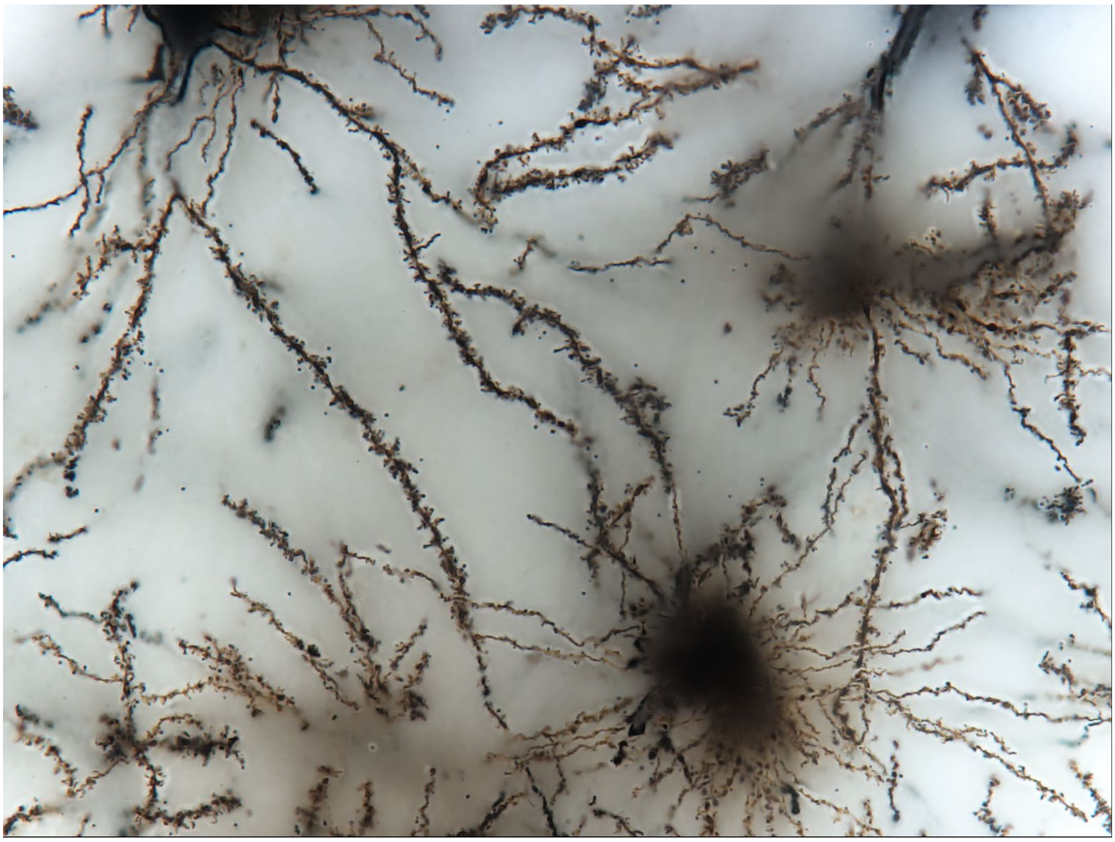

original pictures for basal spines of control group in Figure 2P and Q

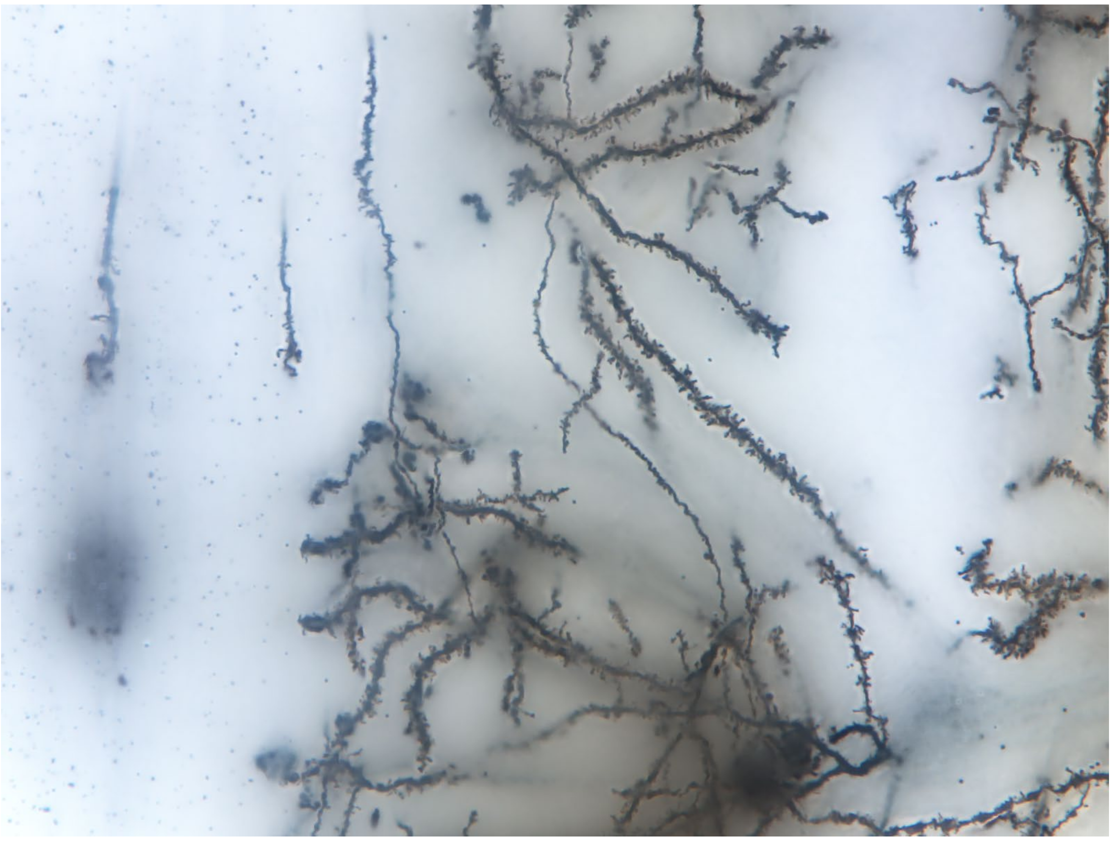

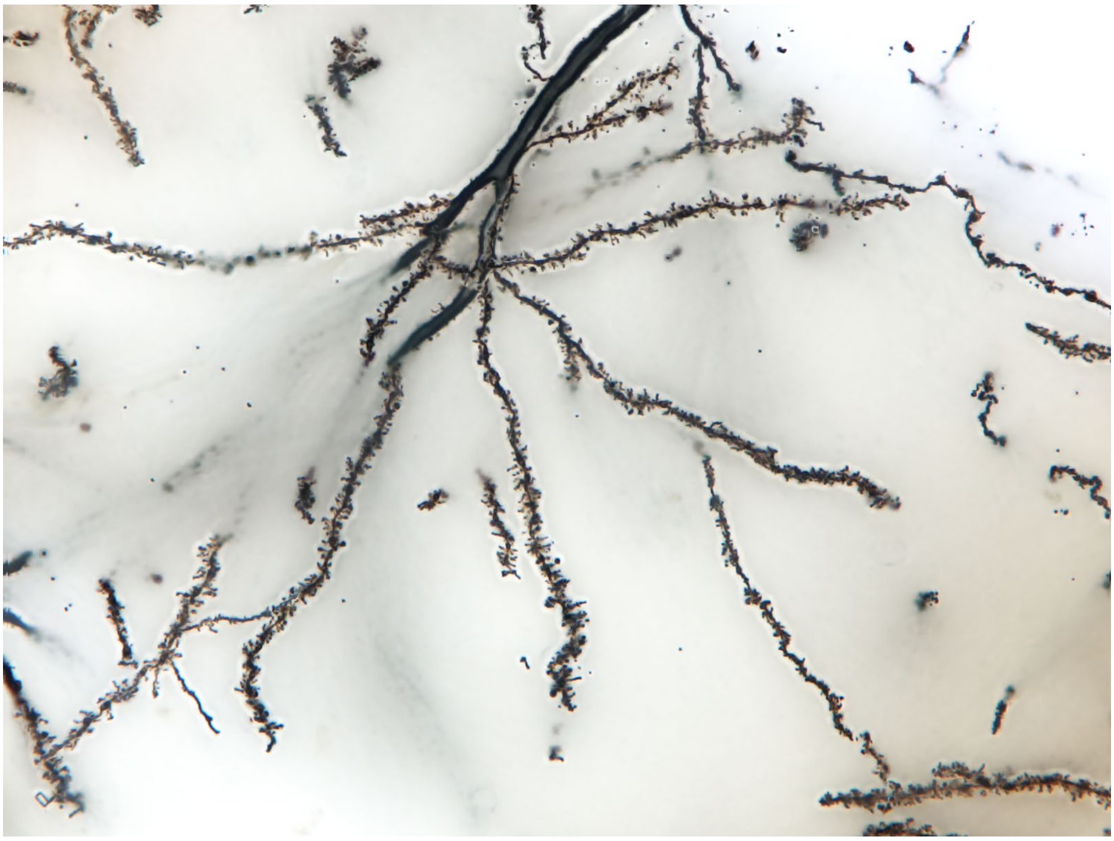

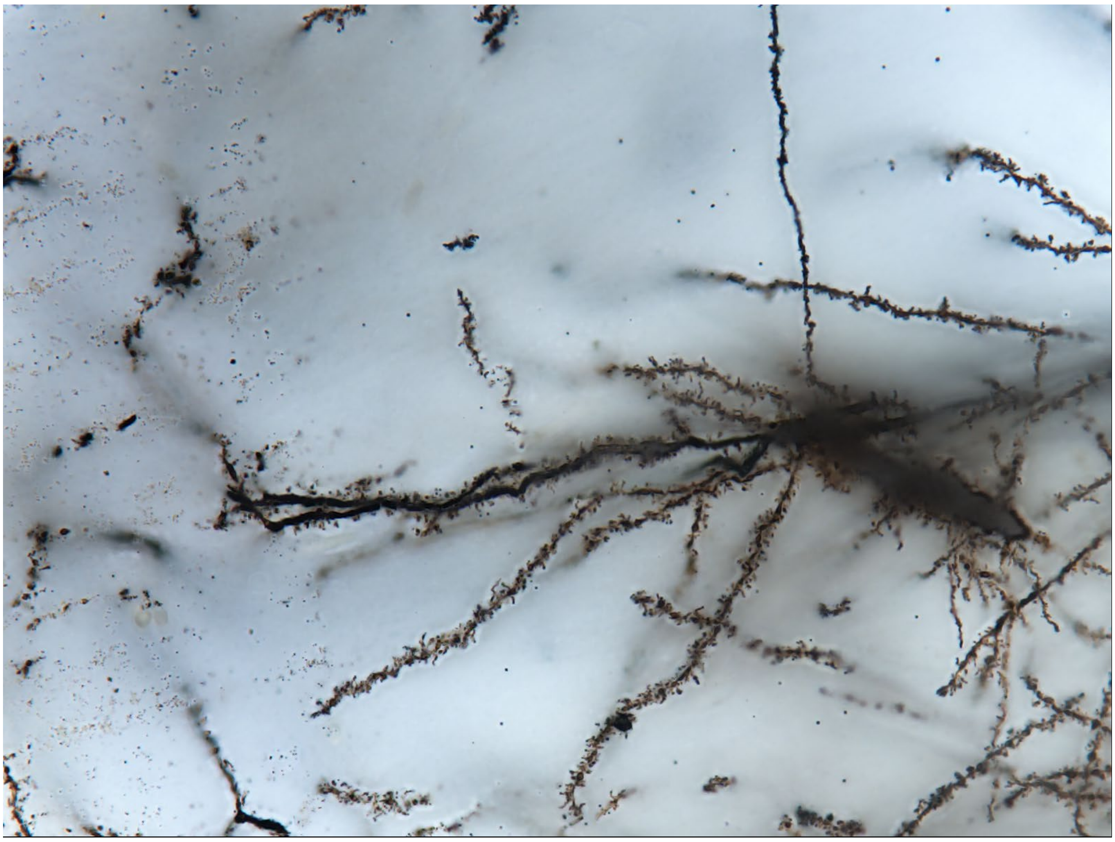

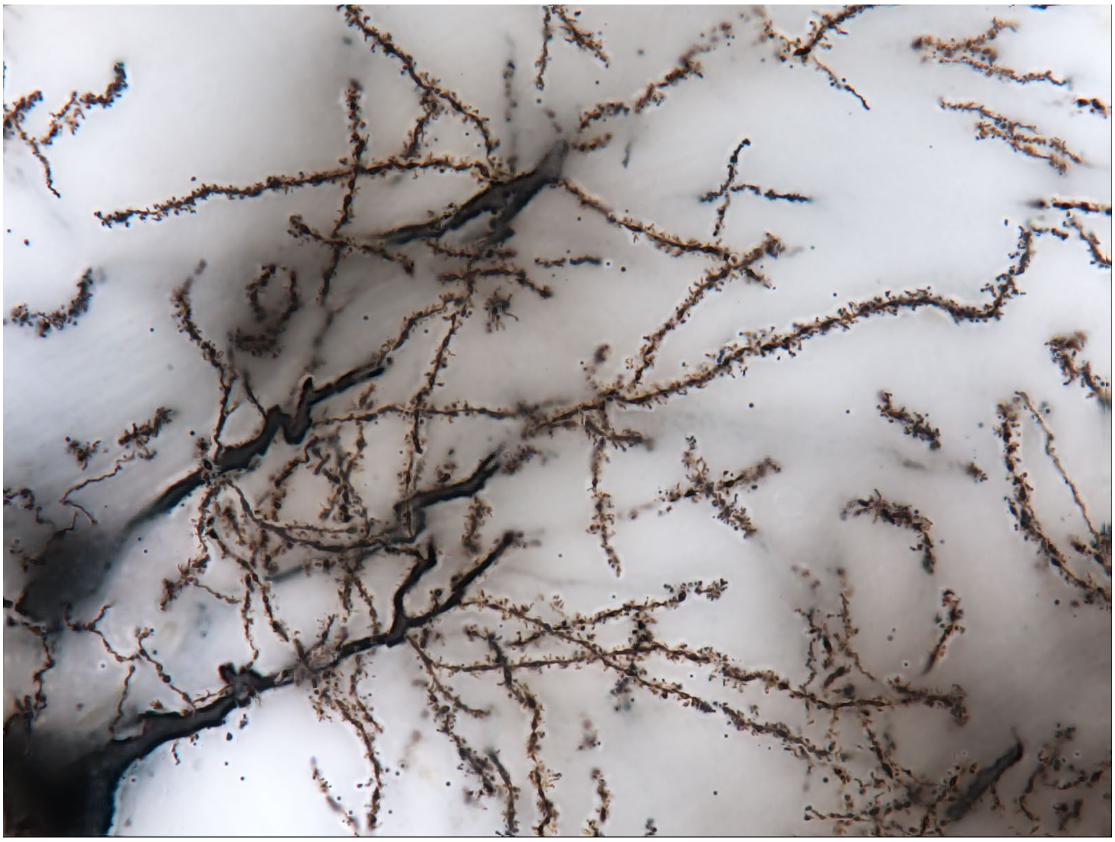

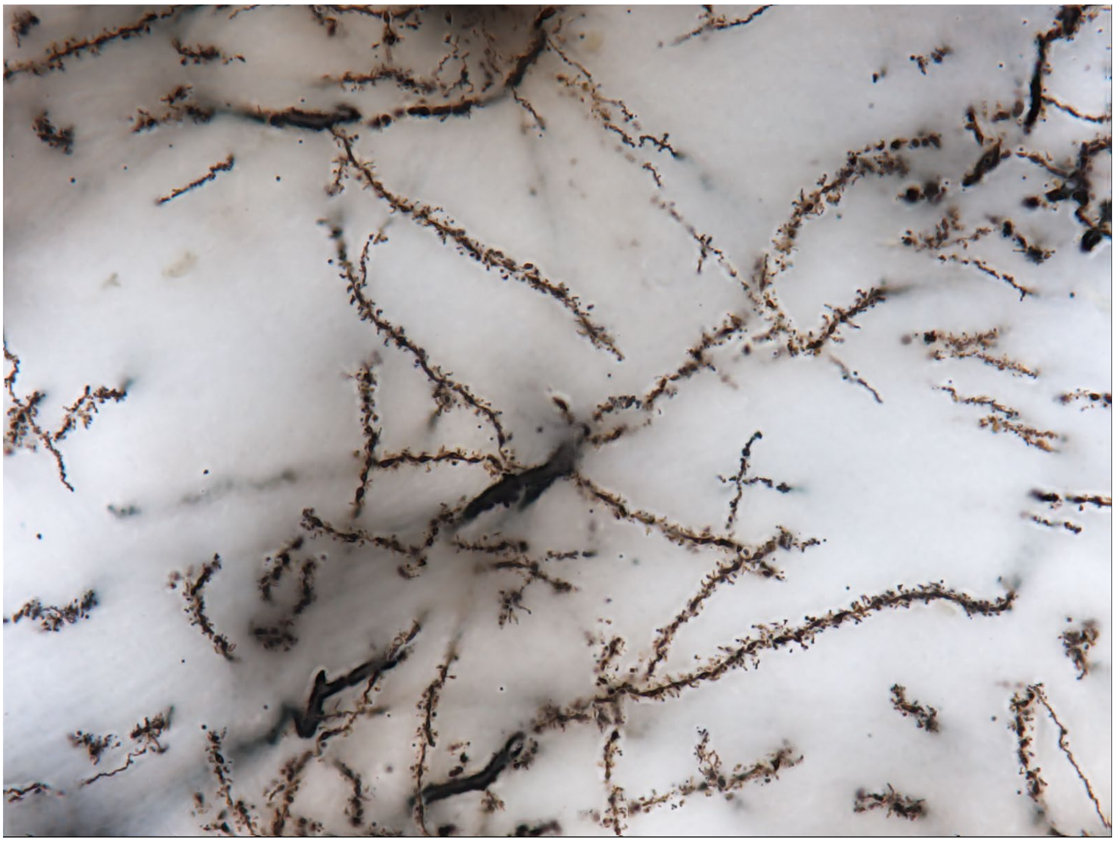

original pictures for apical spines of AIM2-OE group in Figure 2P and Q

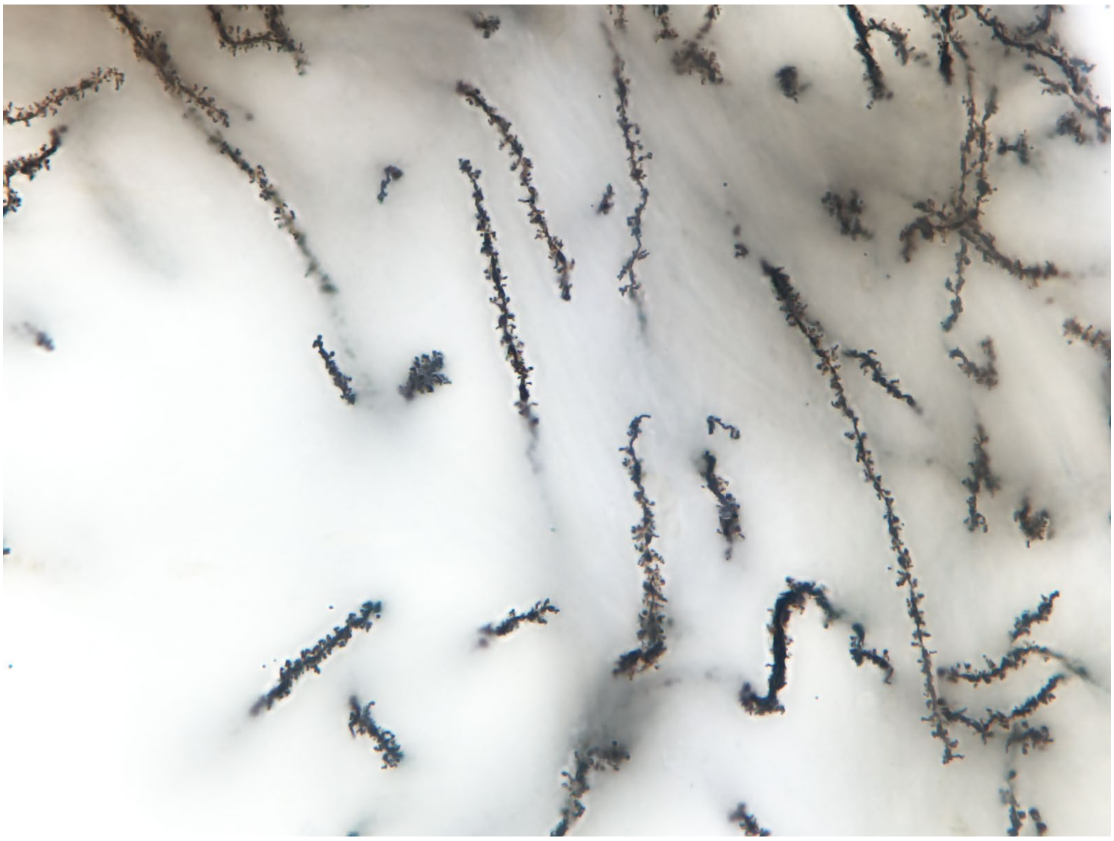

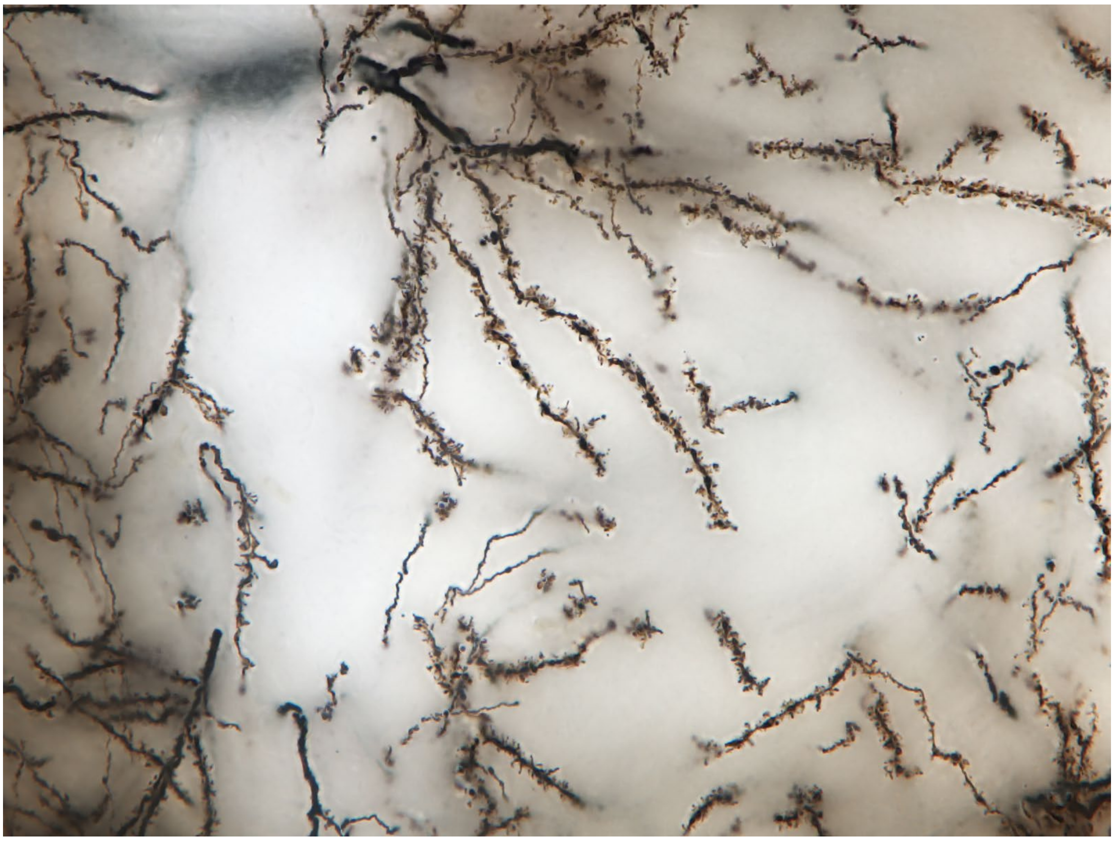

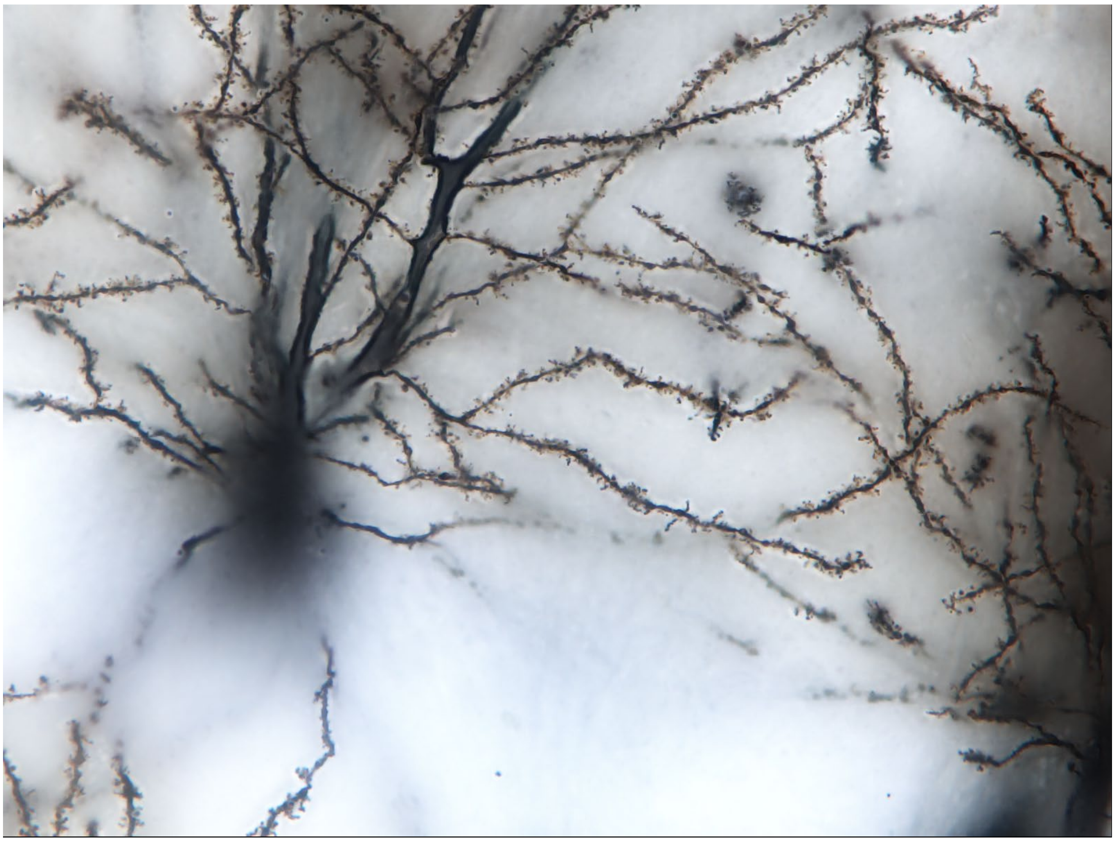

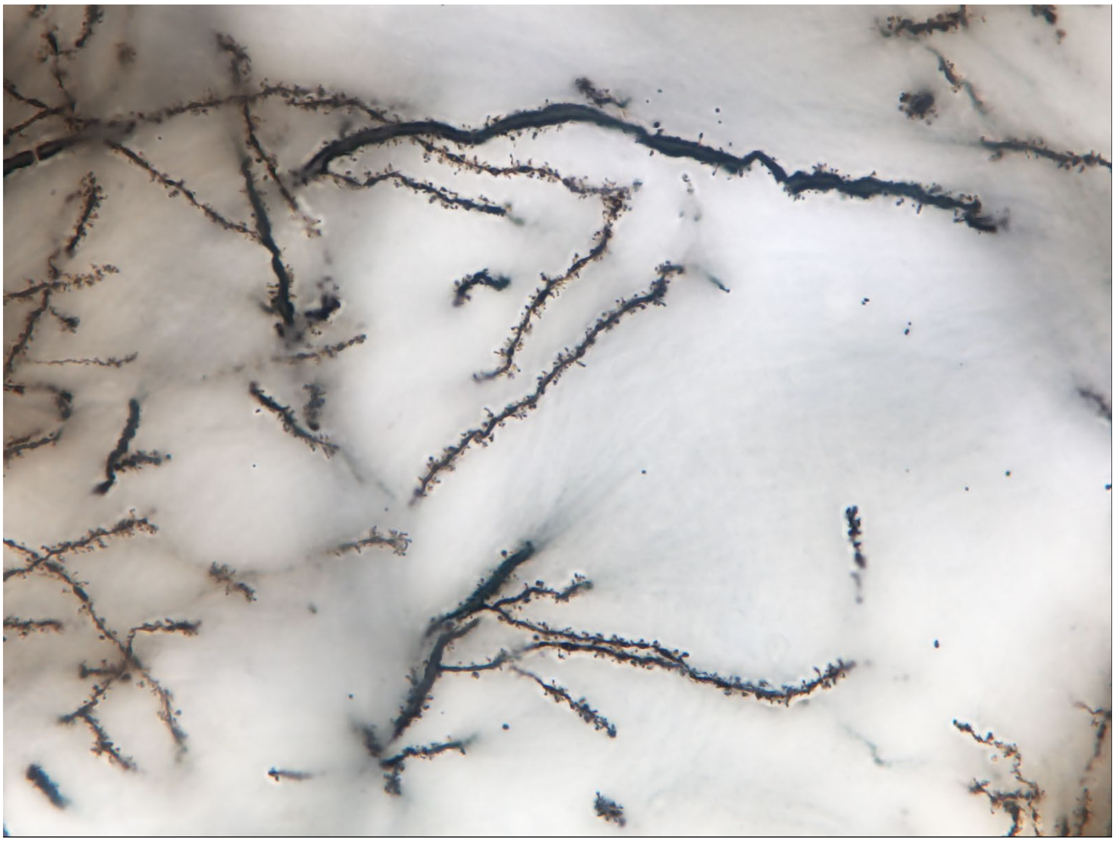

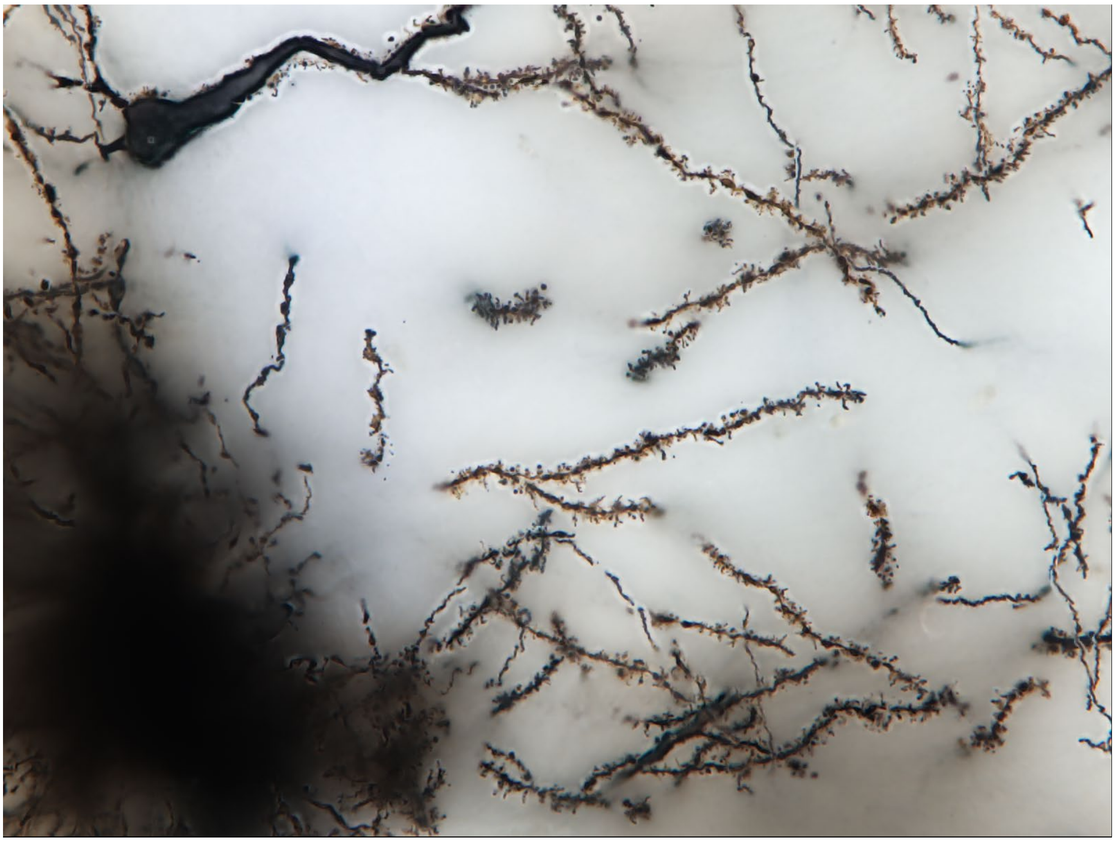

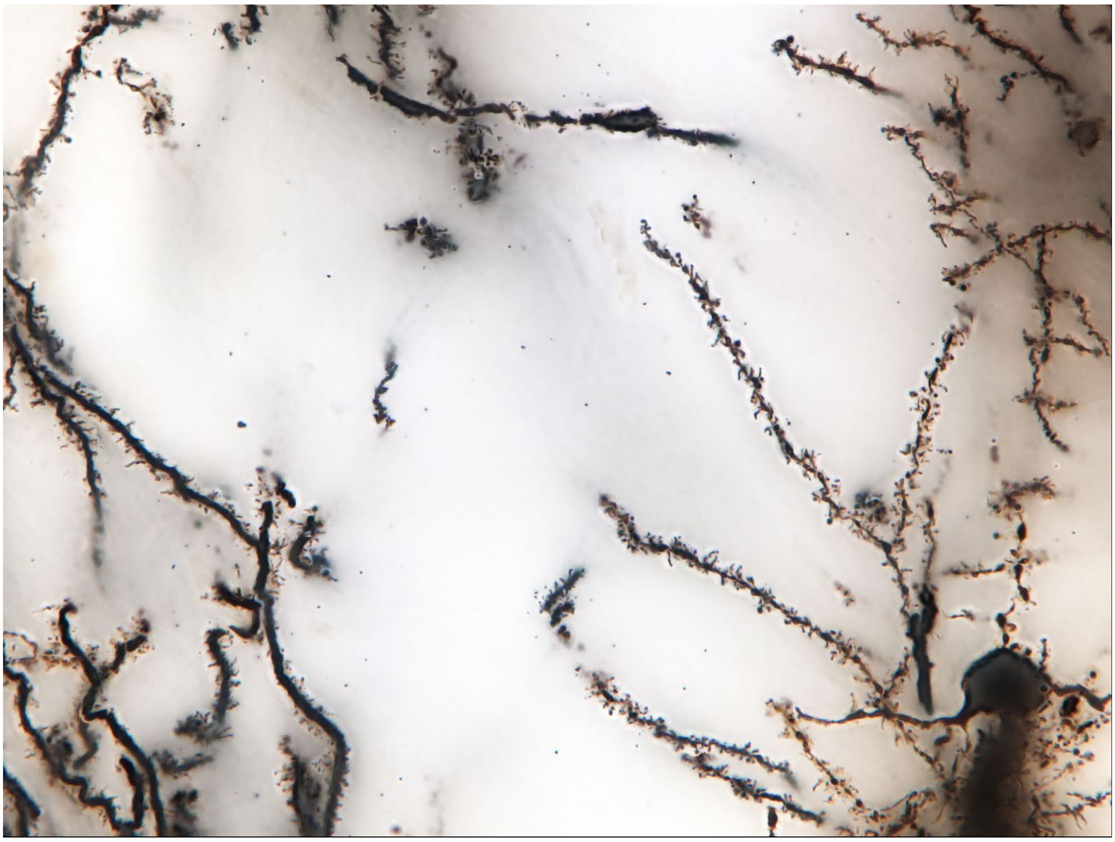

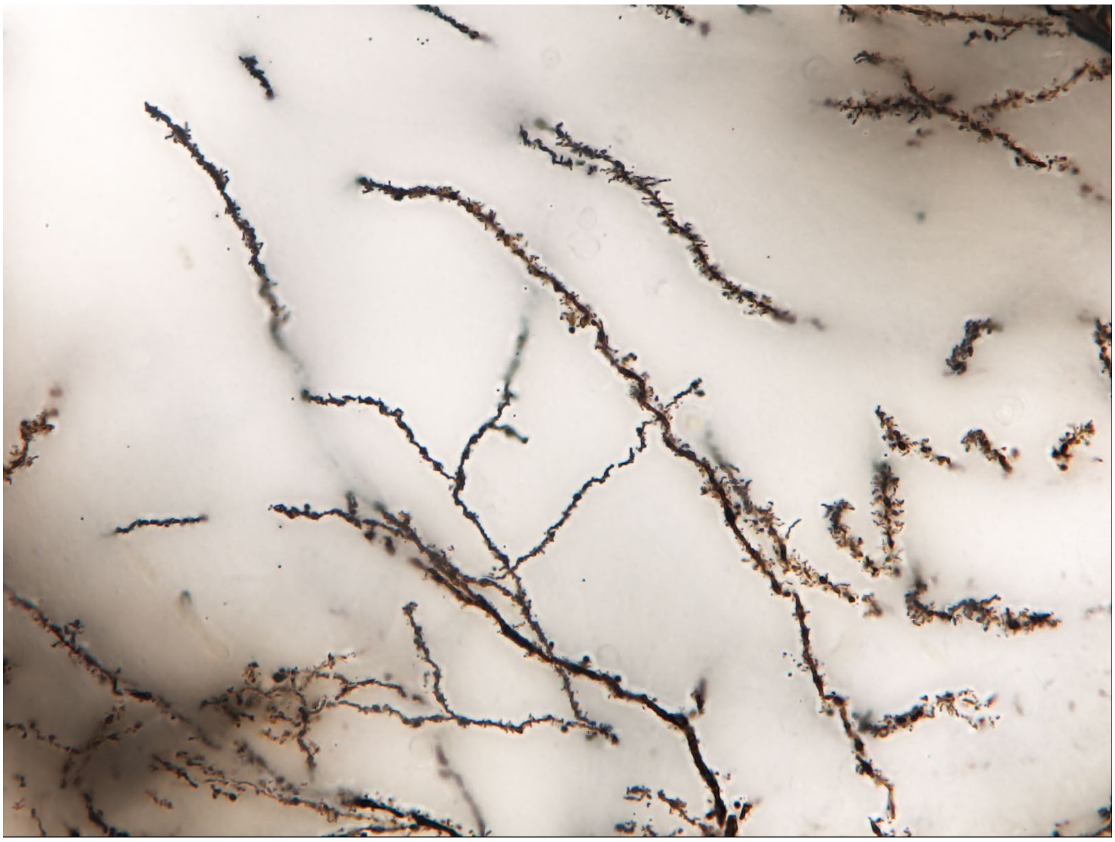

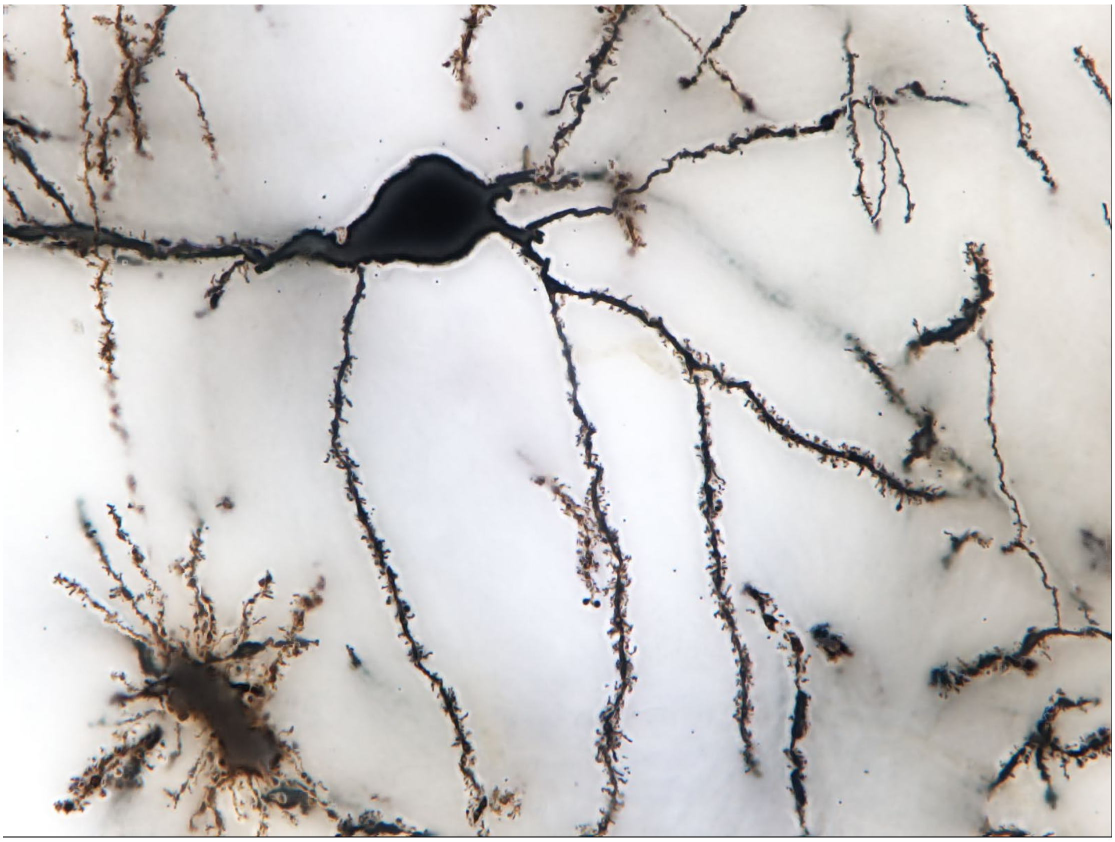

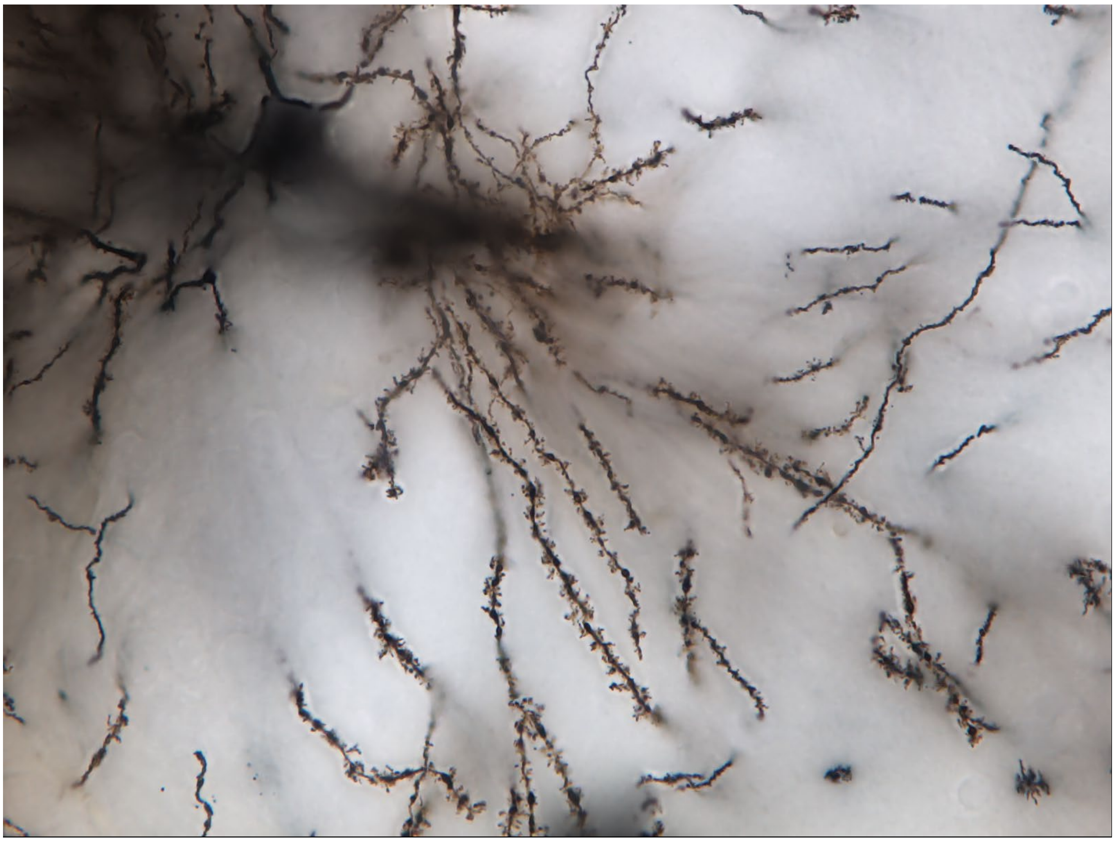

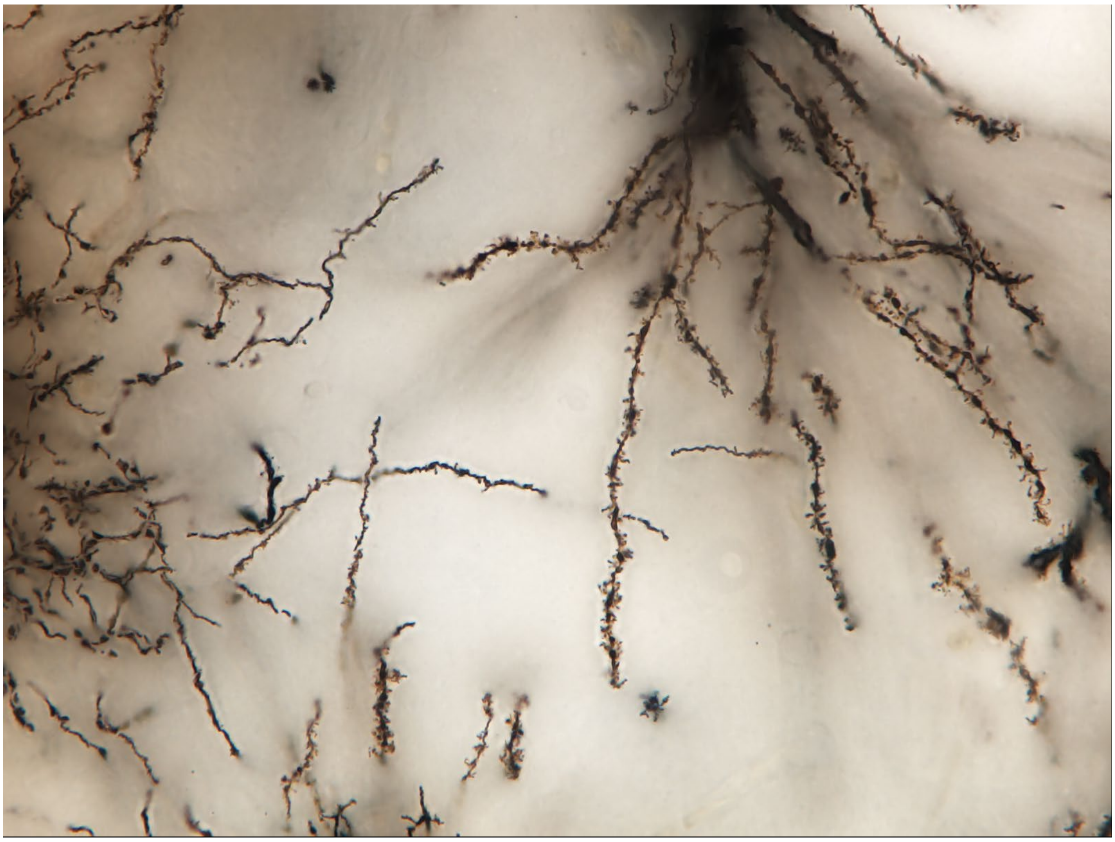

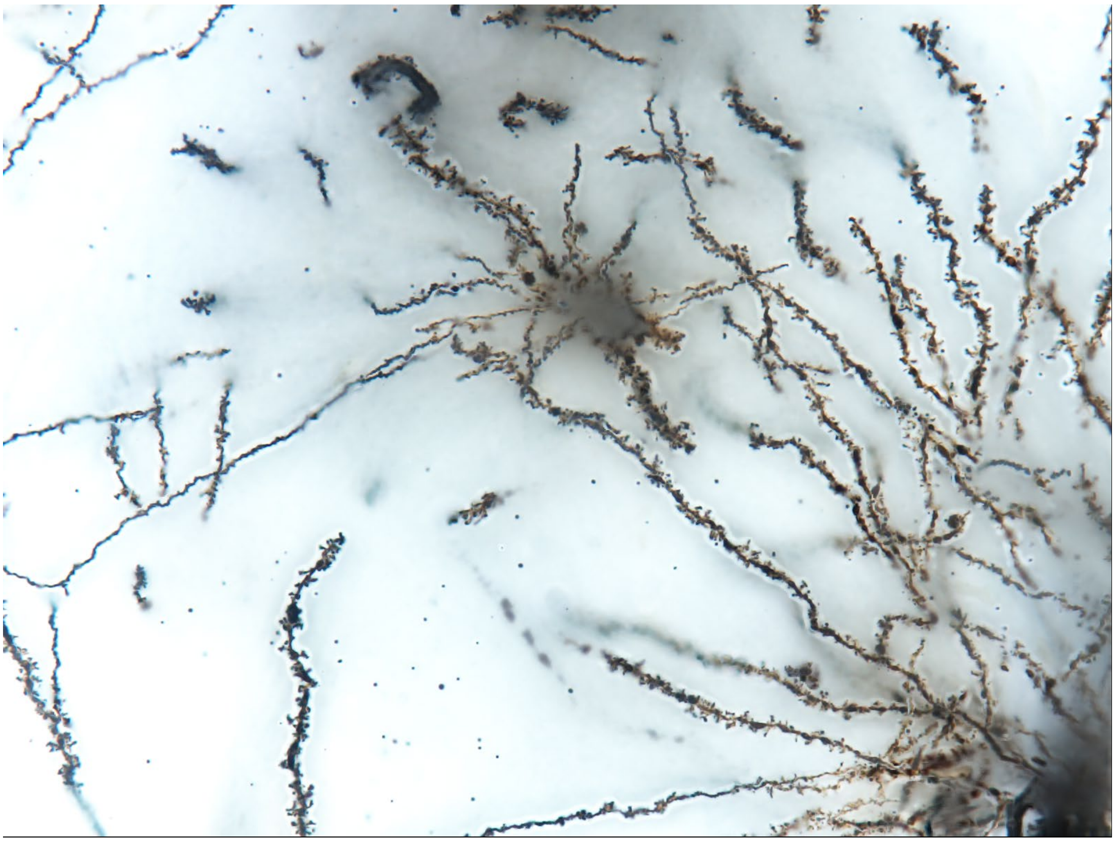

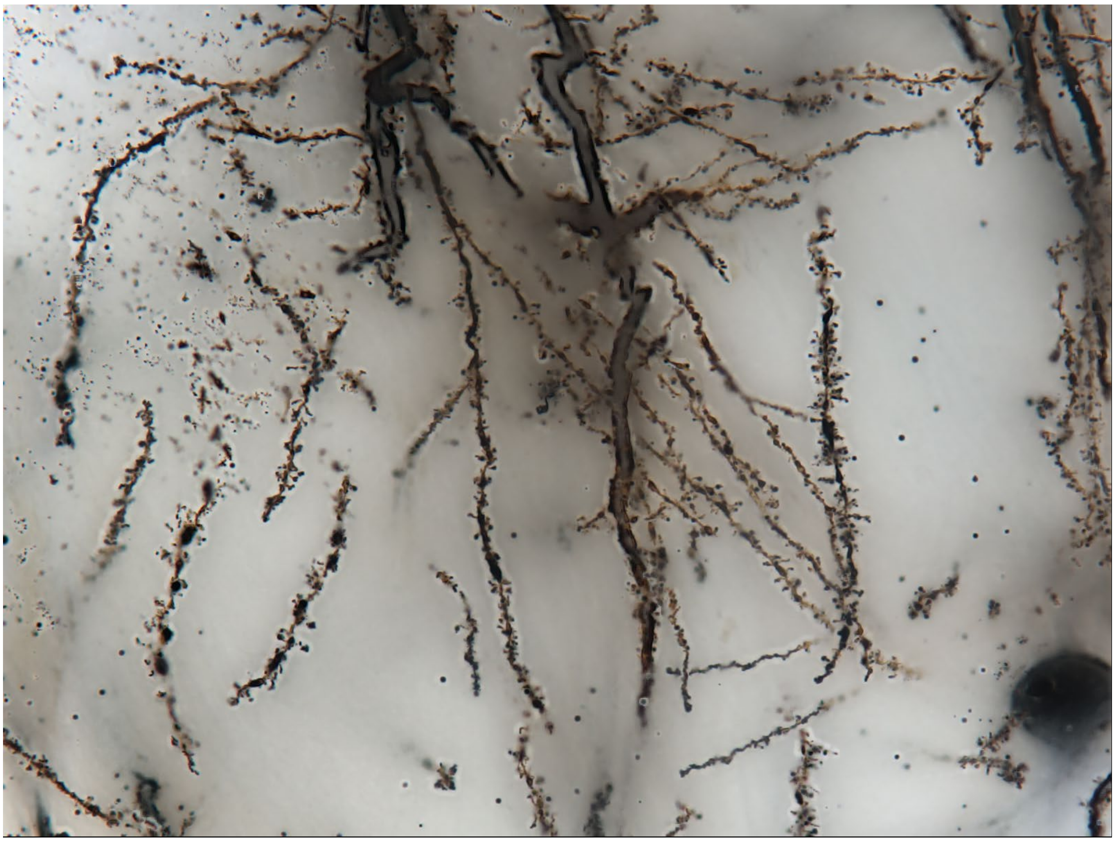

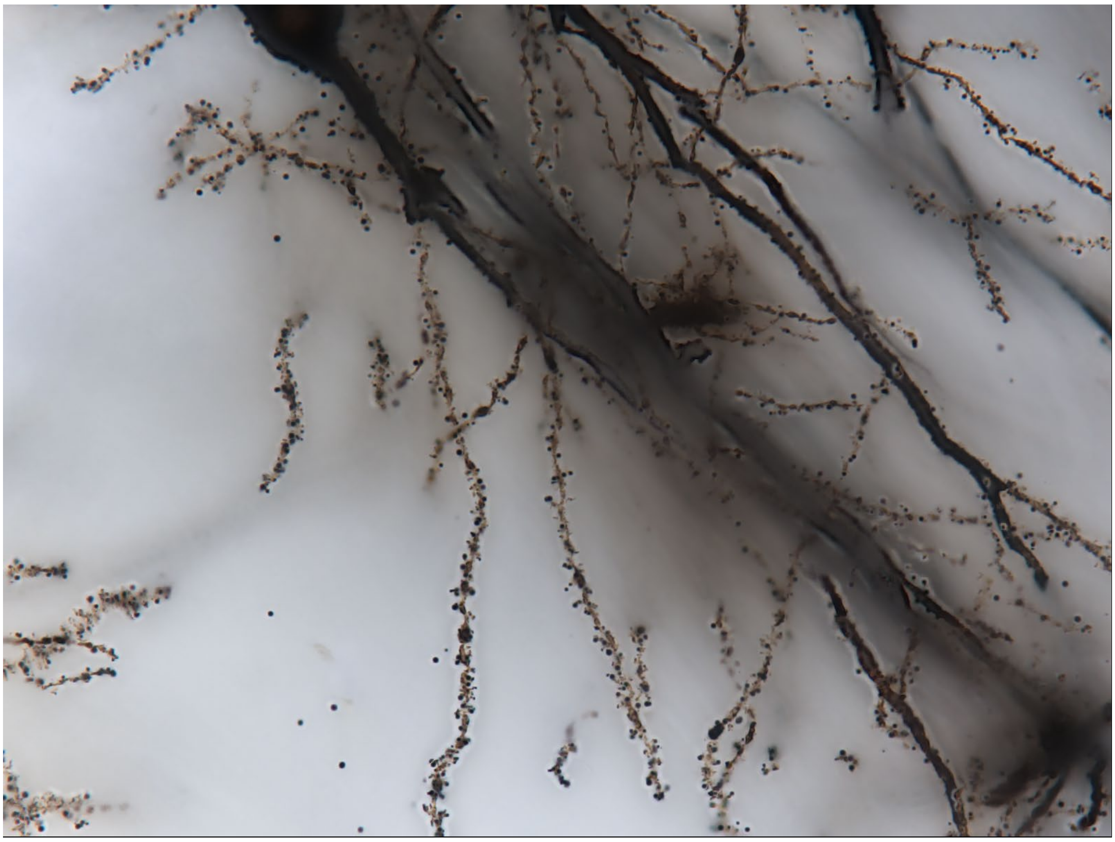

original pictures for basal spines of AIM2-OE group in Figure 2P and Q

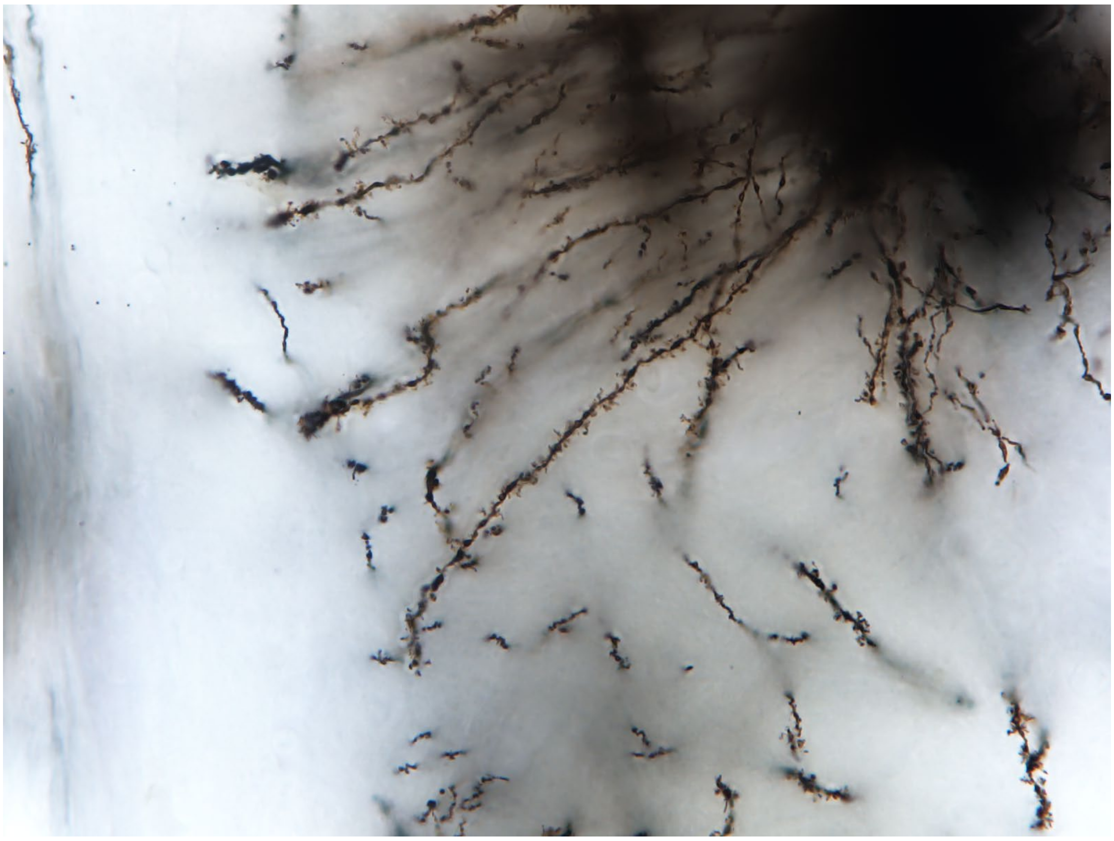

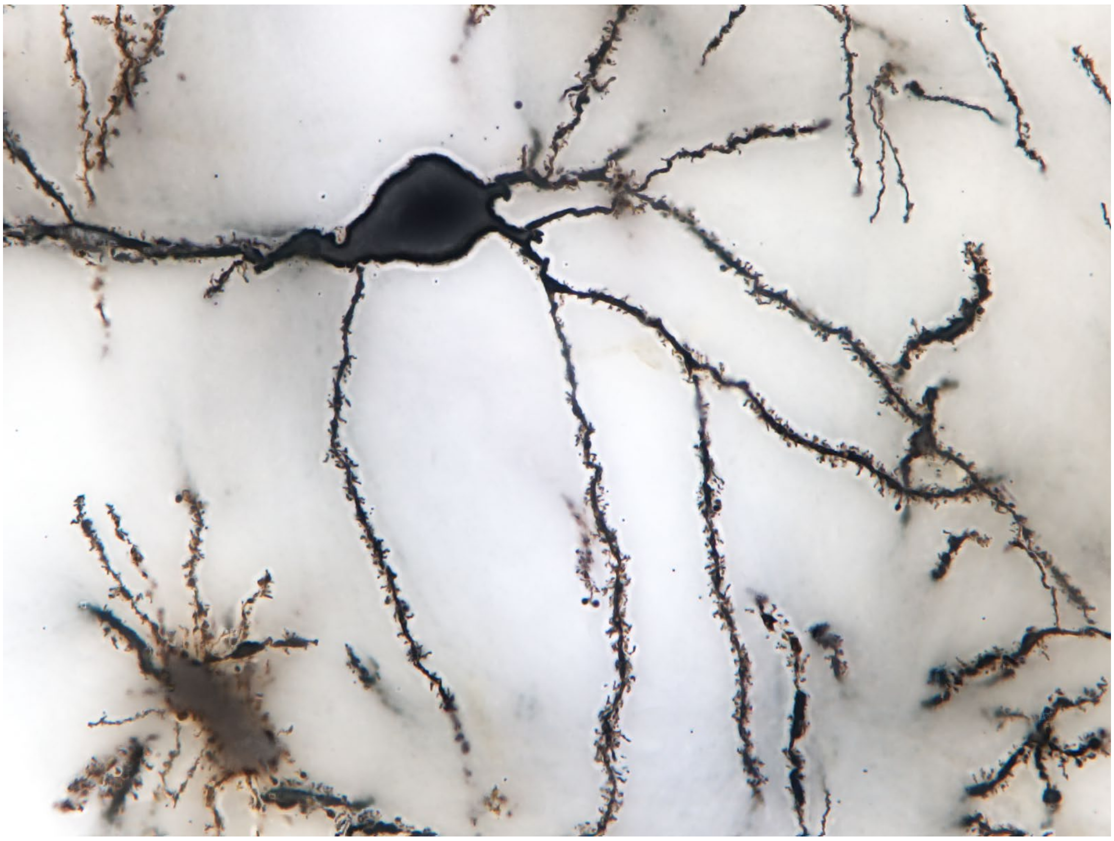

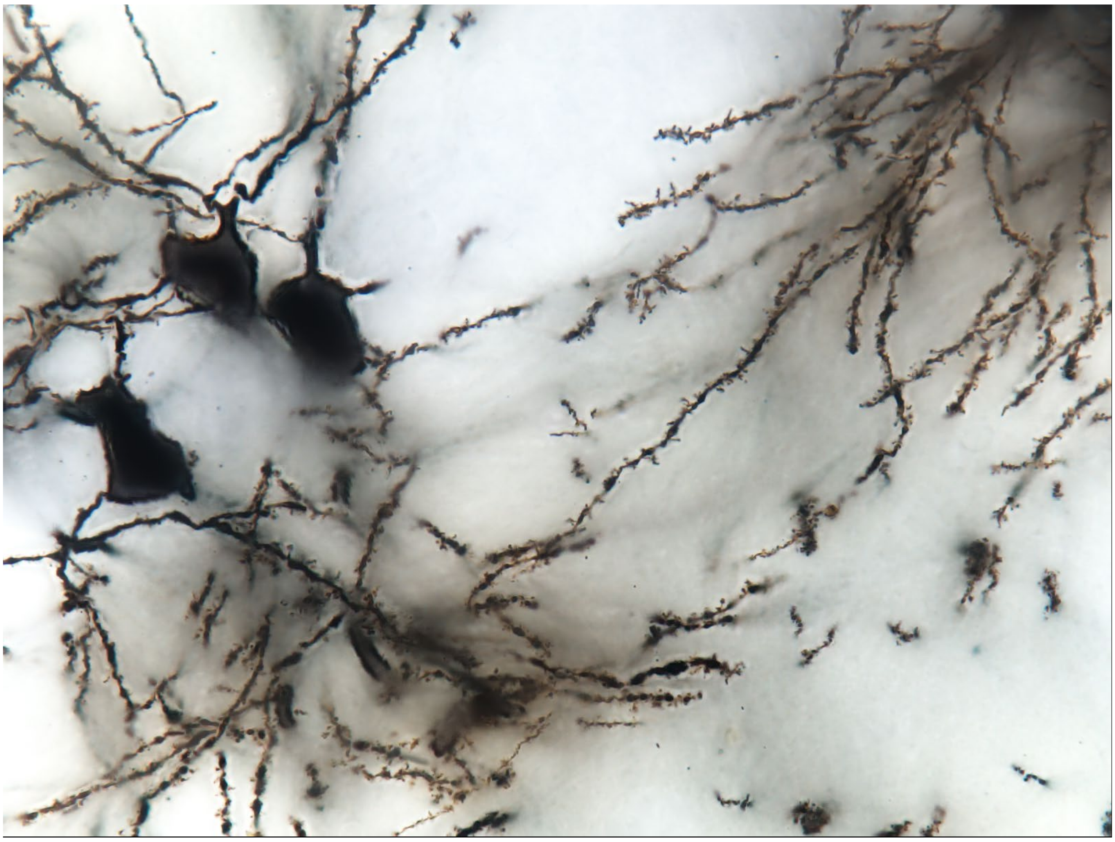

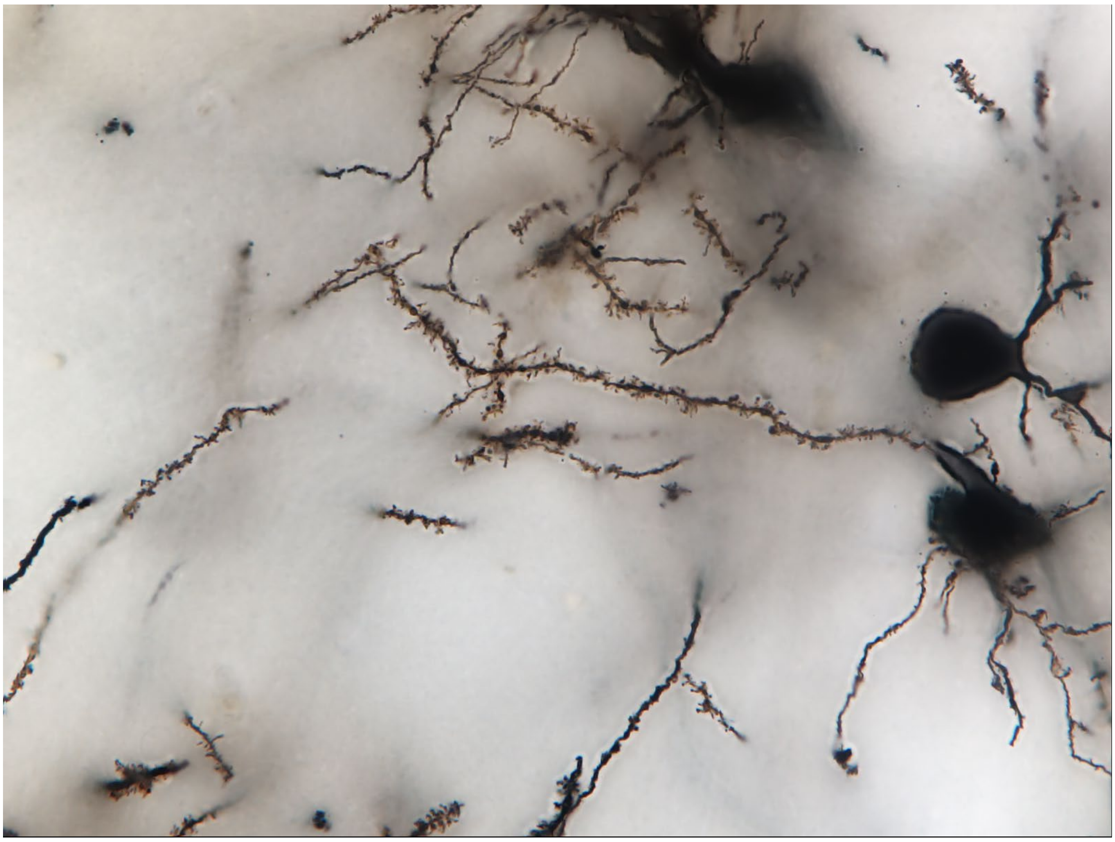

original pictures for Figure 4D

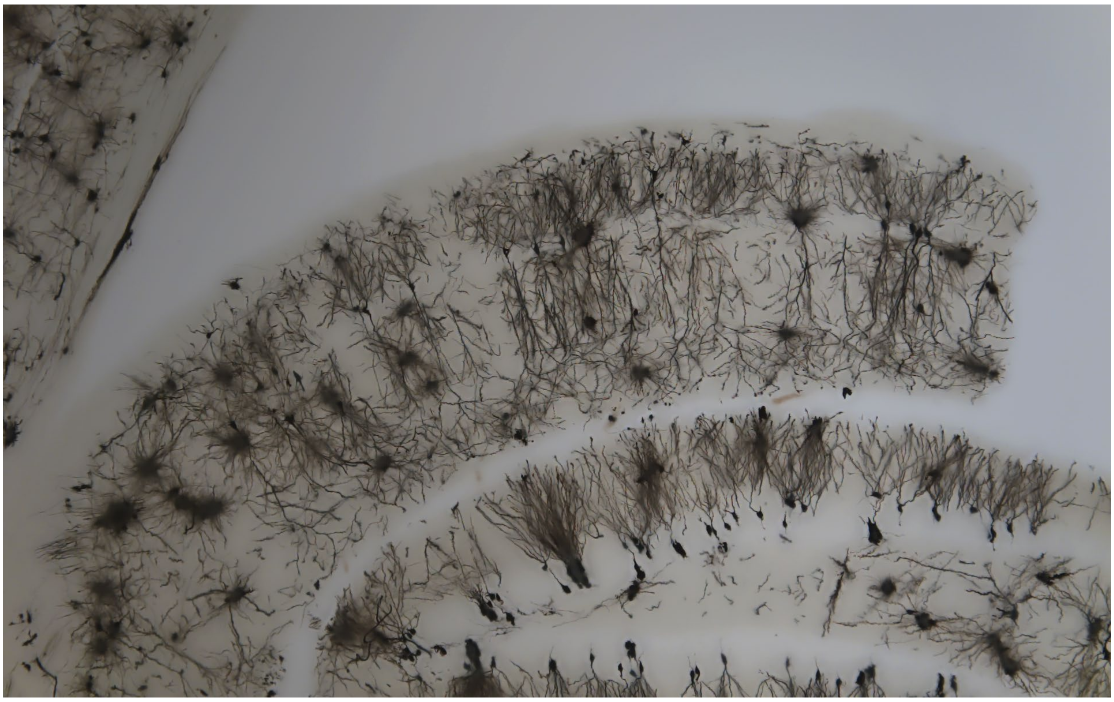

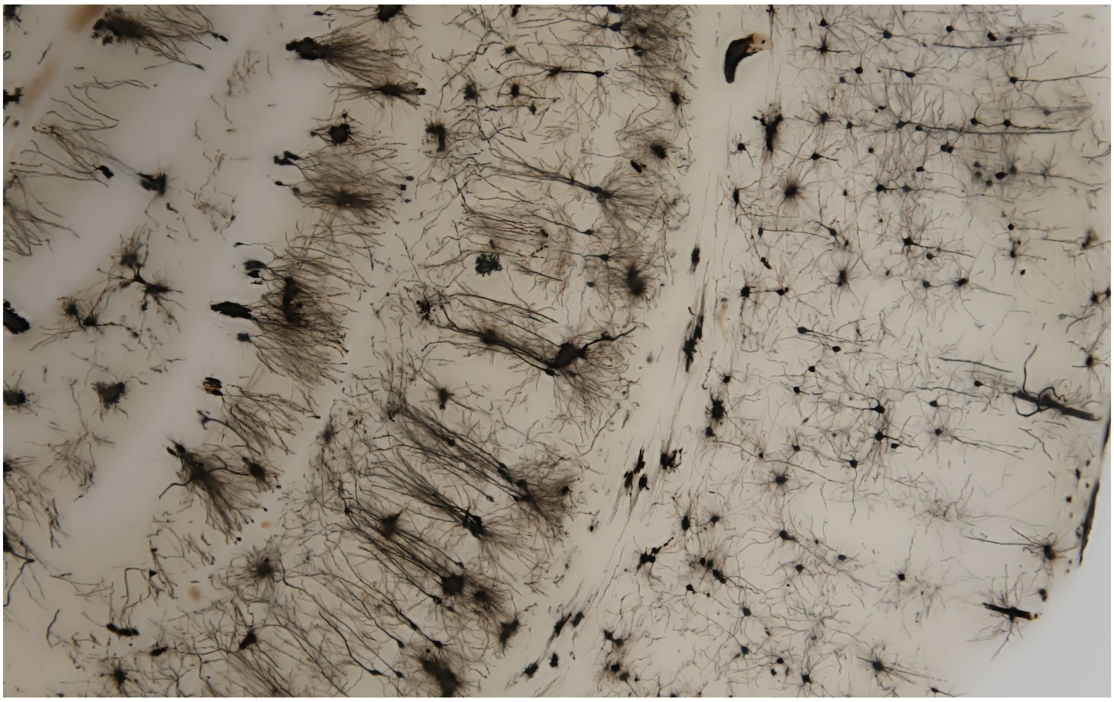

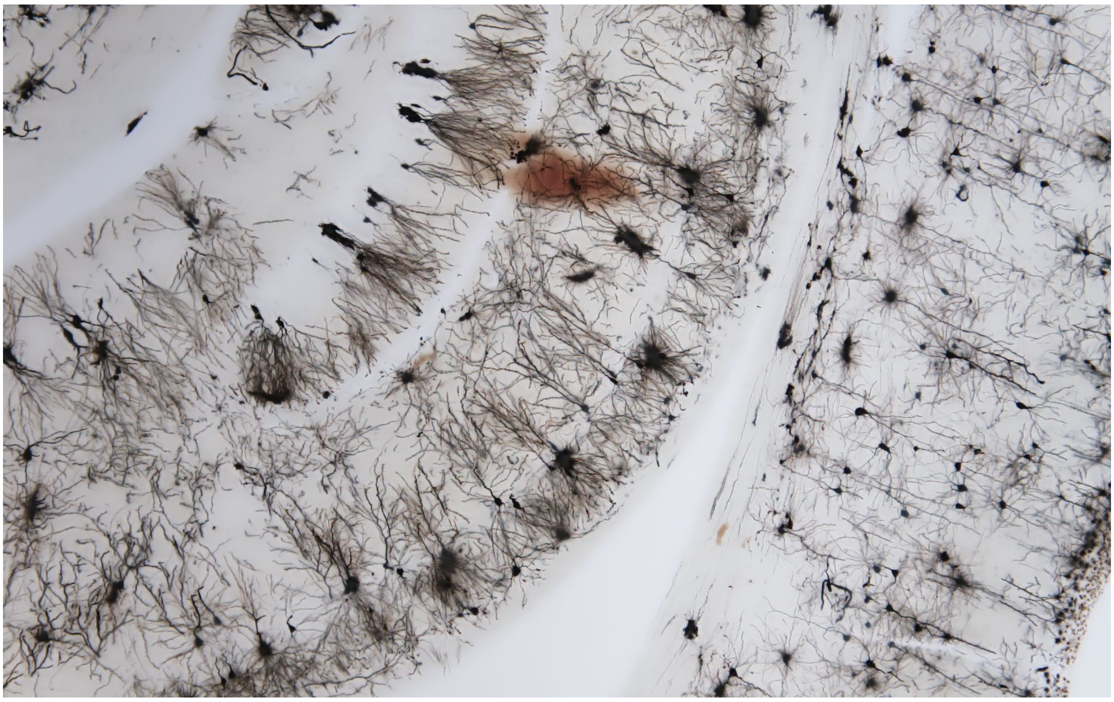

original pictures for WT-PBS group in Figure 4F

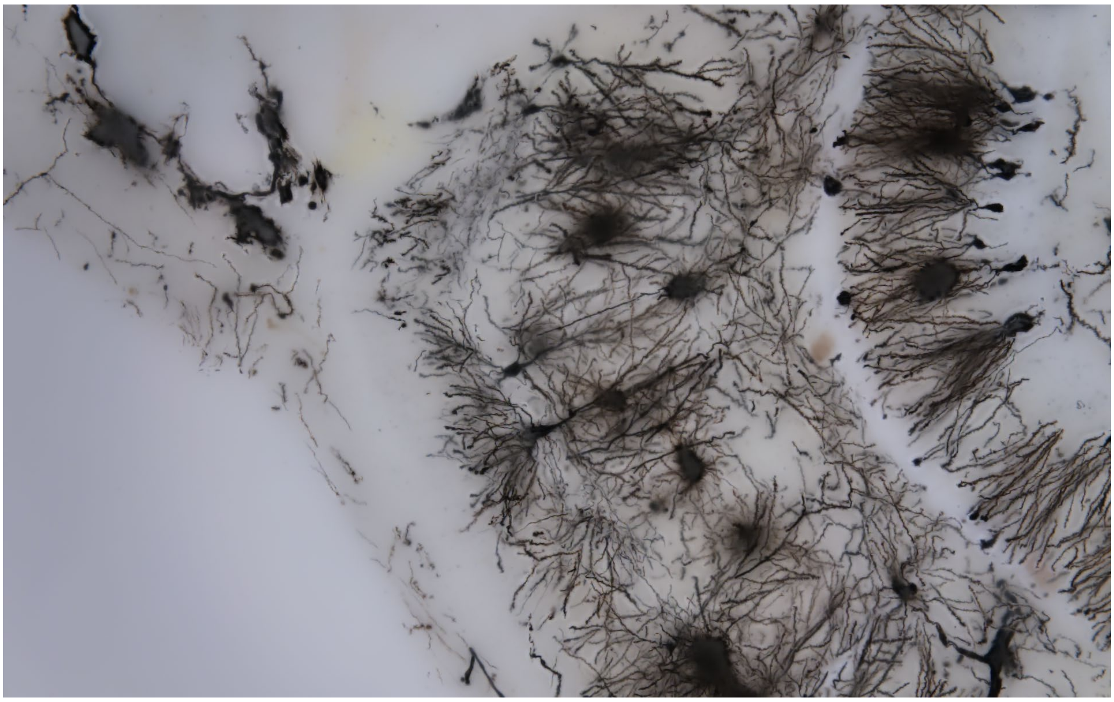

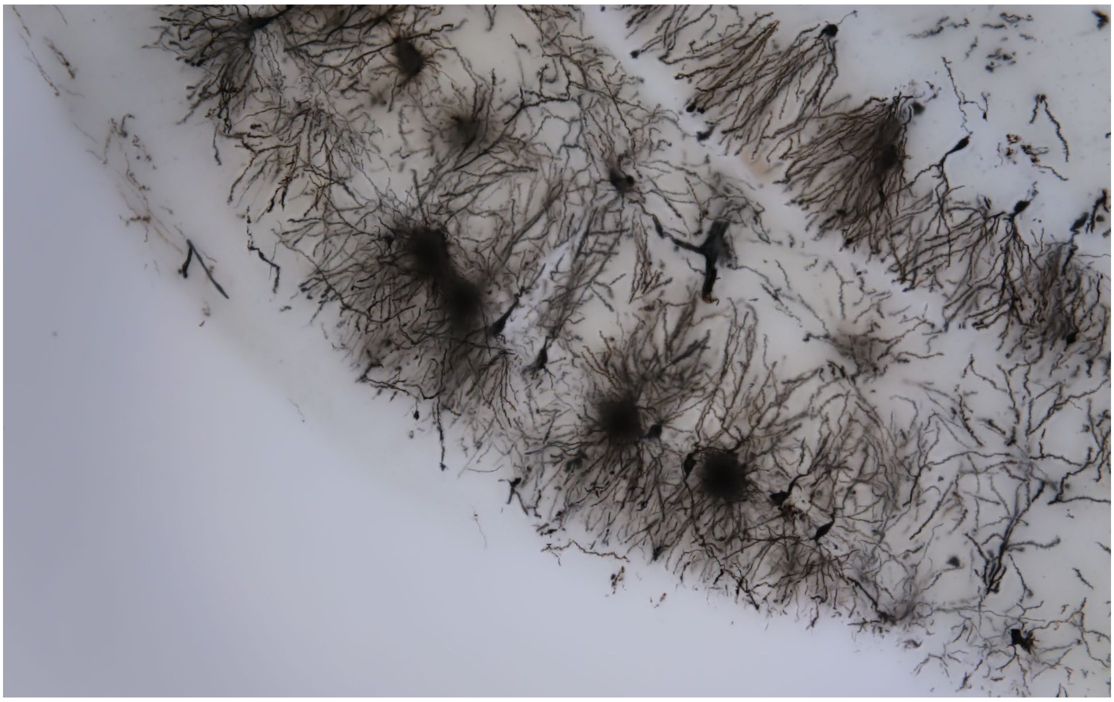

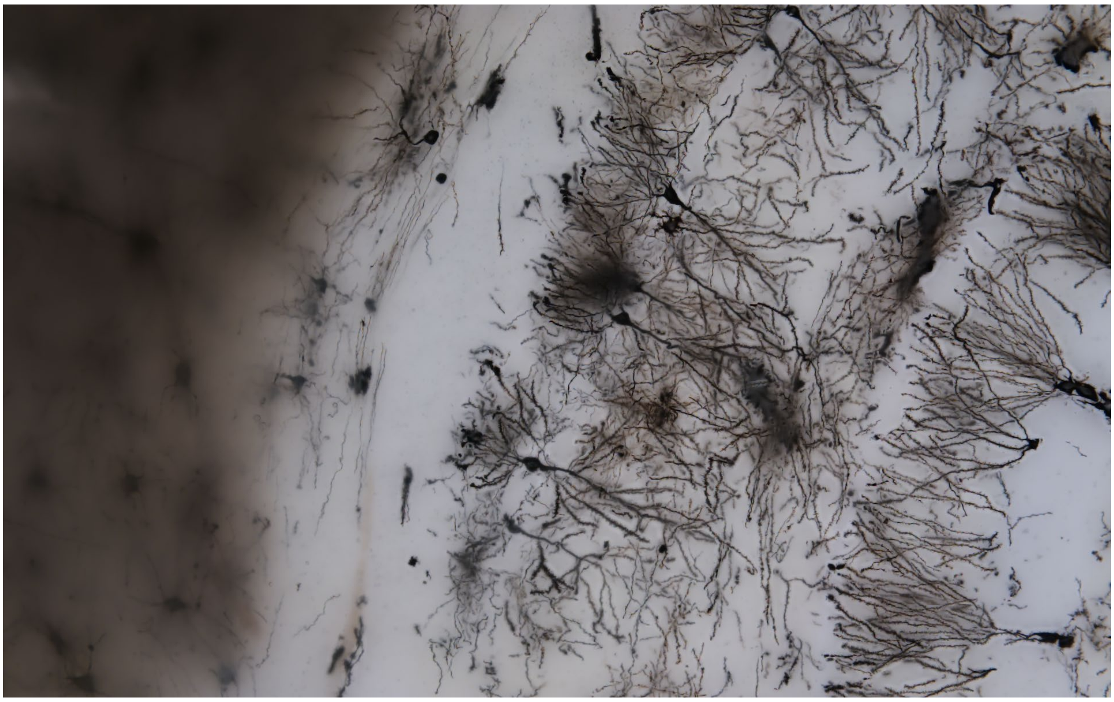

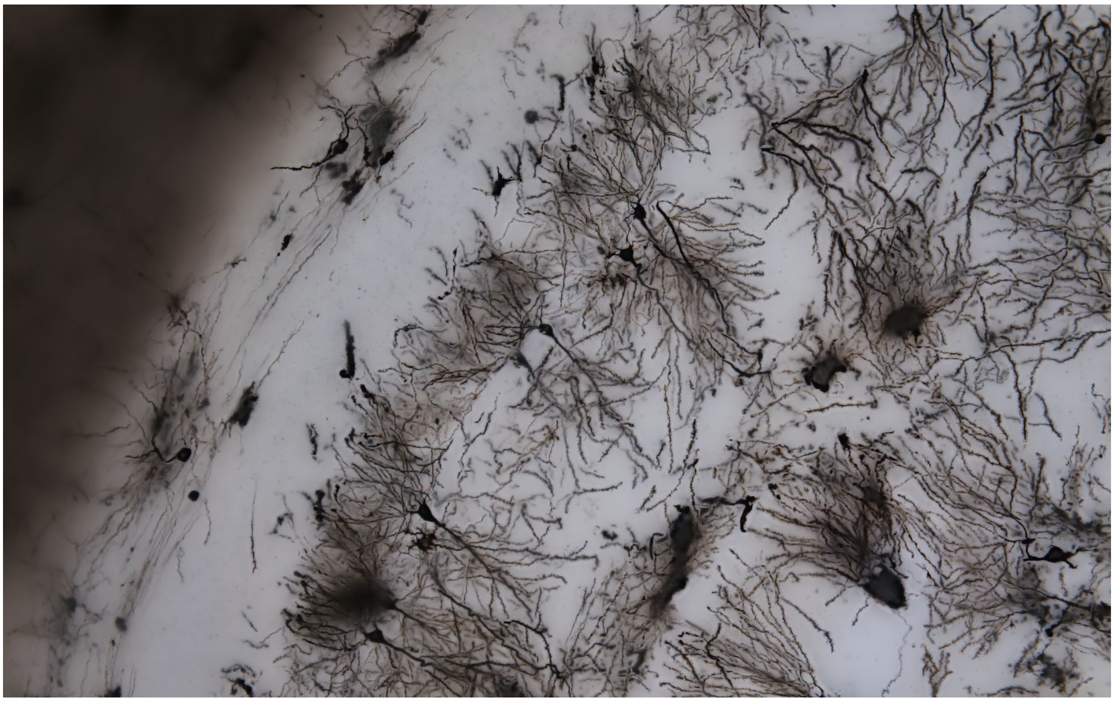

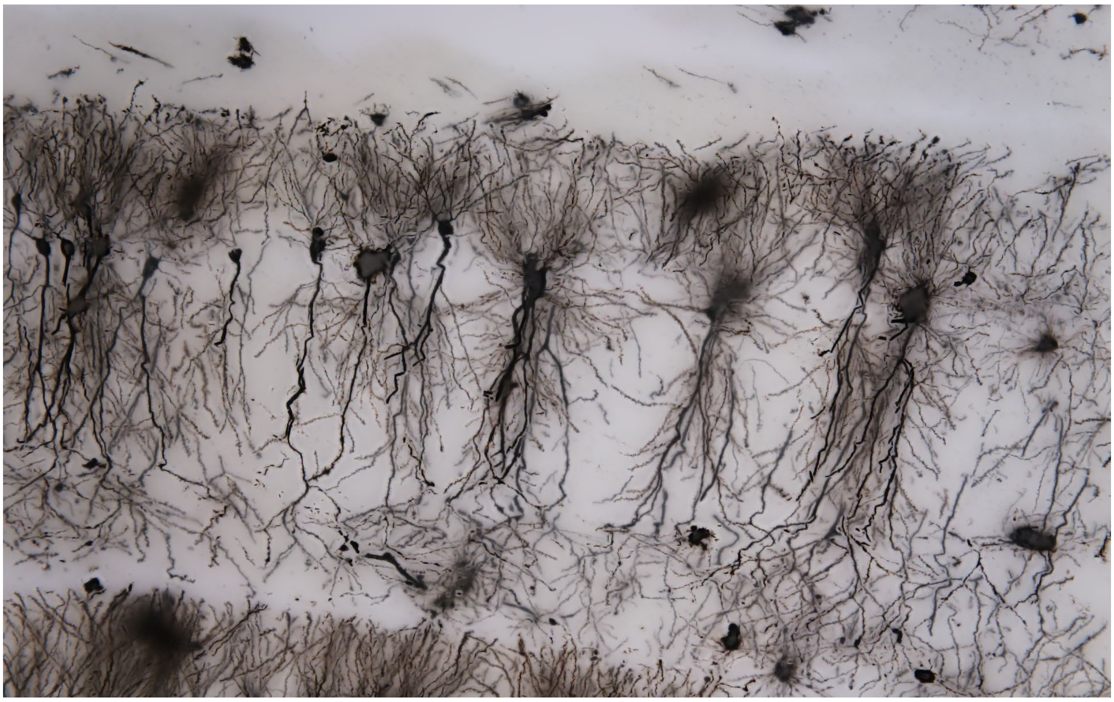

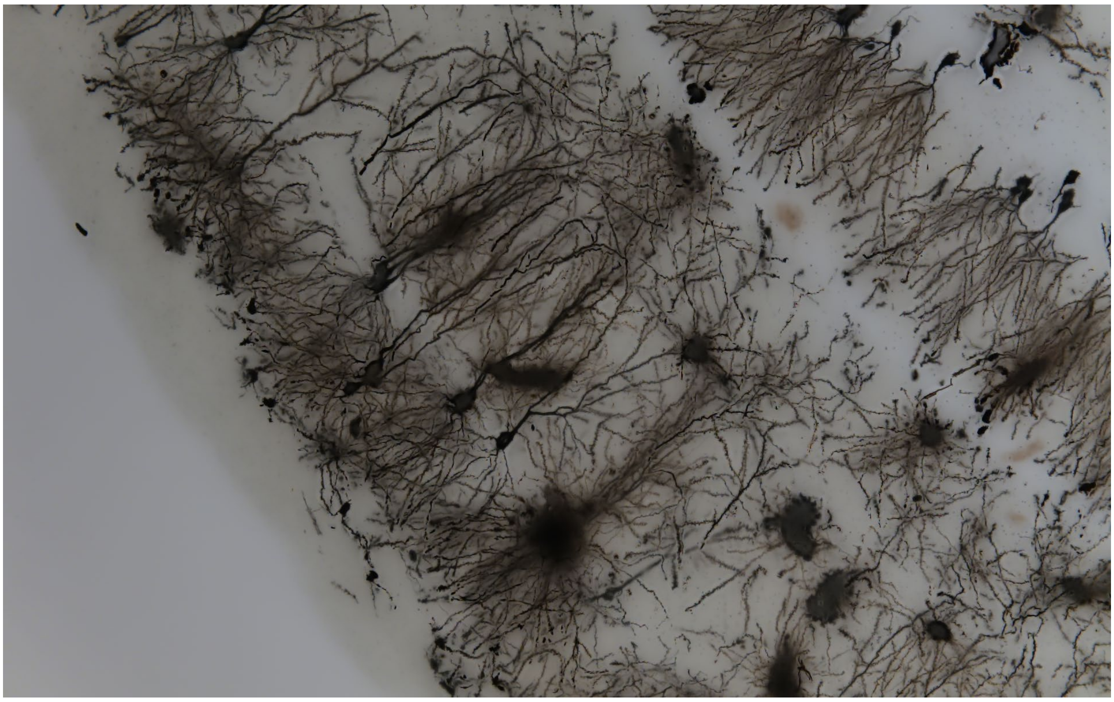

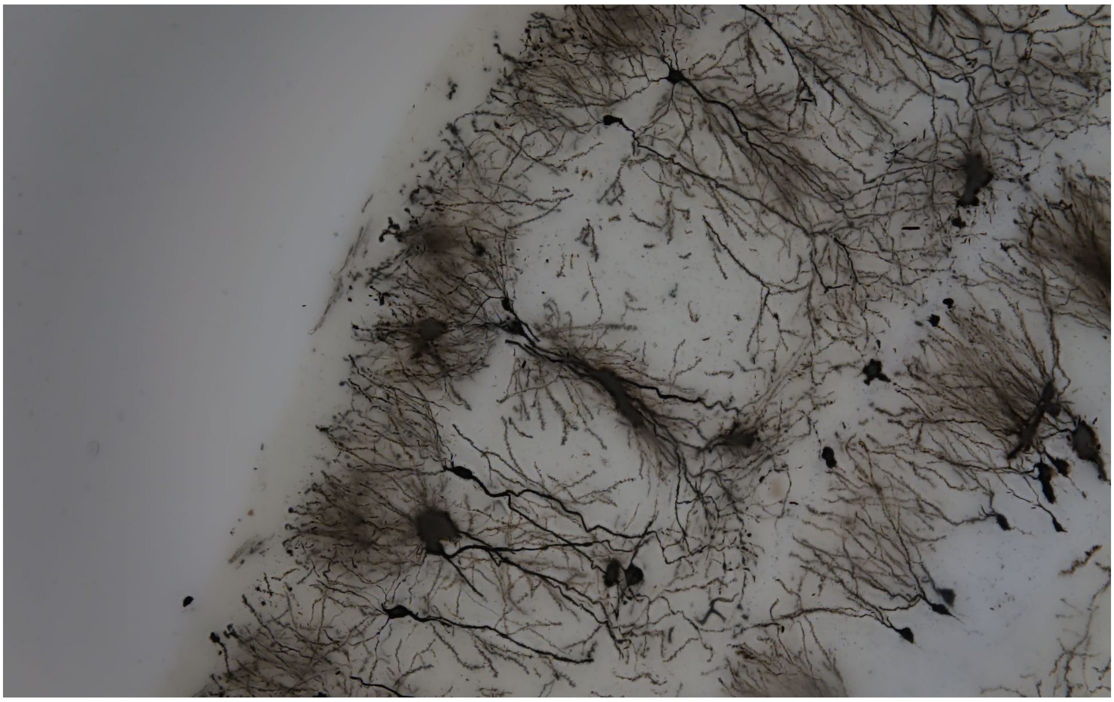

original pictures for WT-Aβ group in Figure 4F

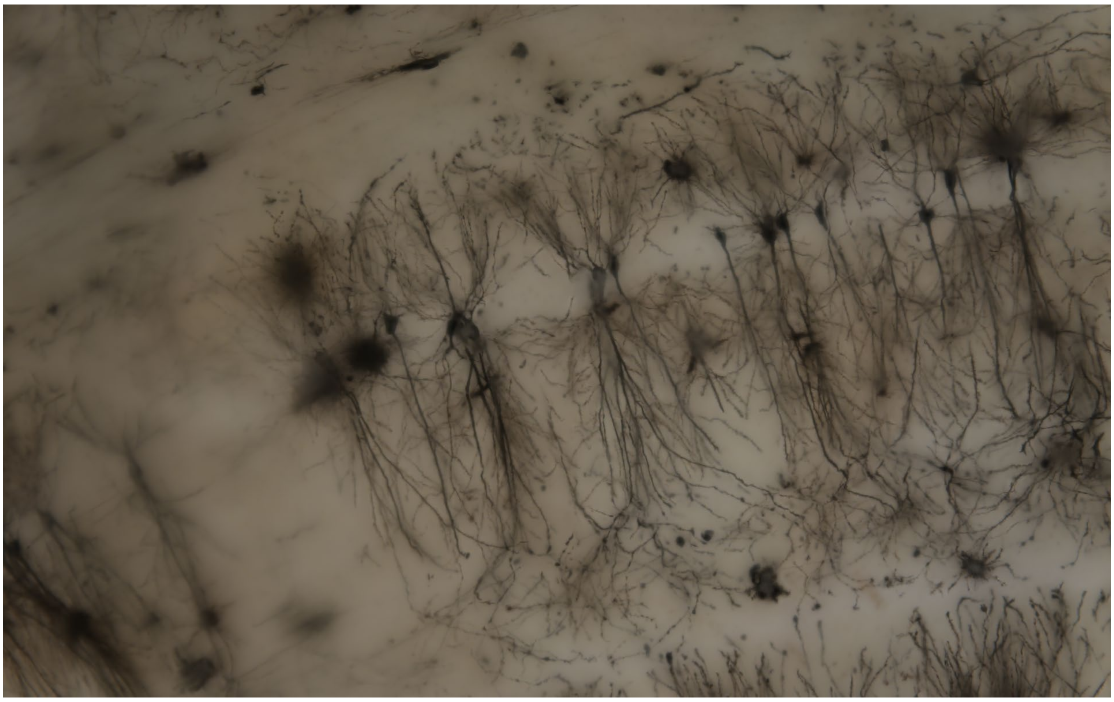

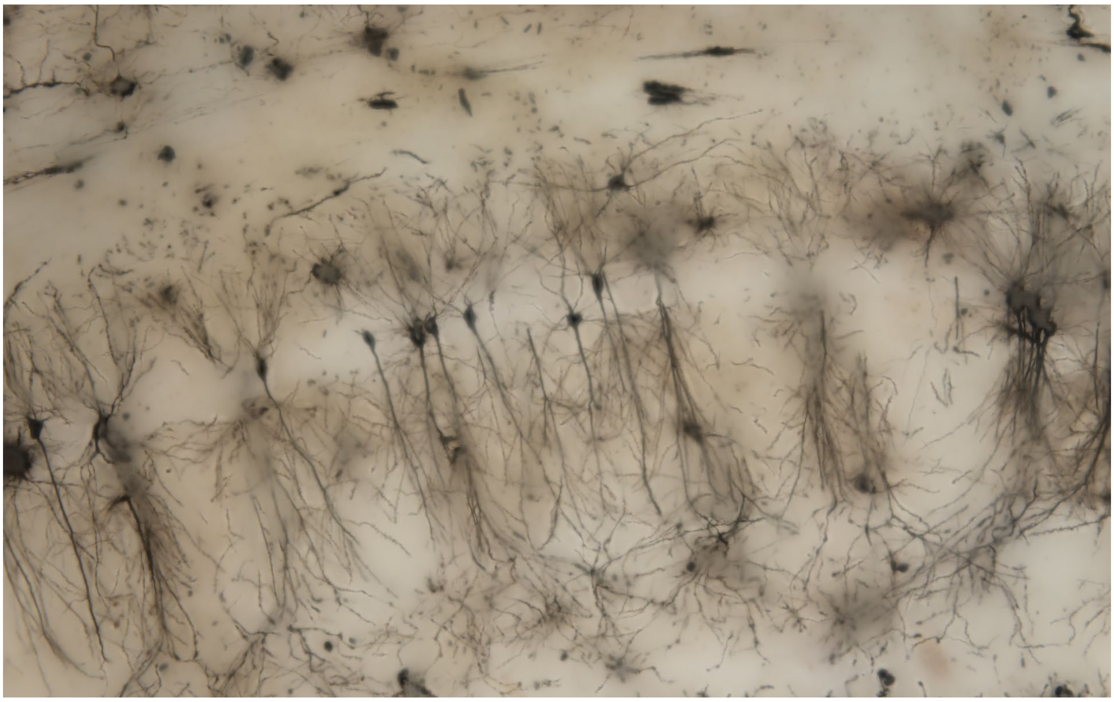

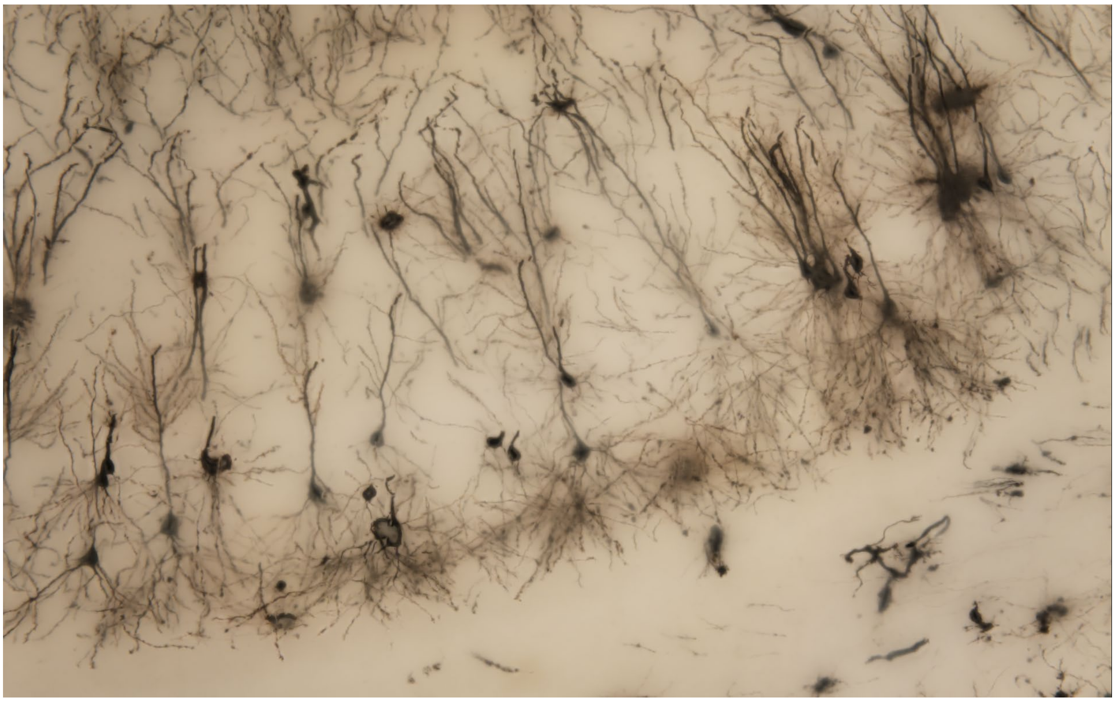

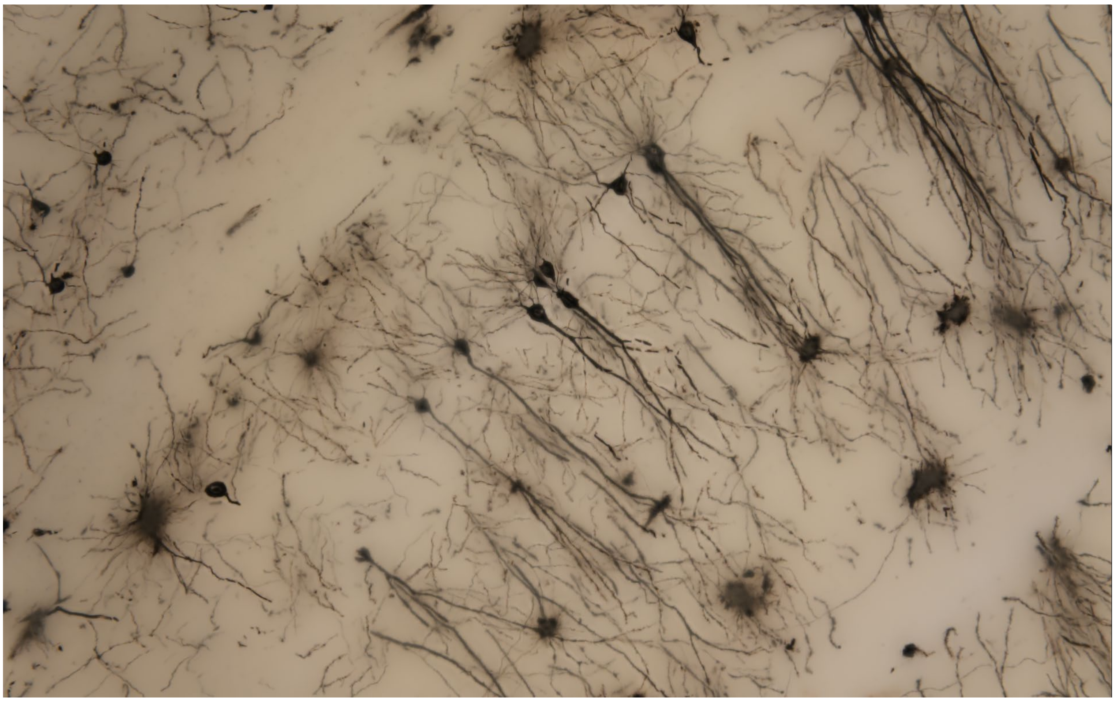

original pictures for cKO-Aβ group in Figure 4F

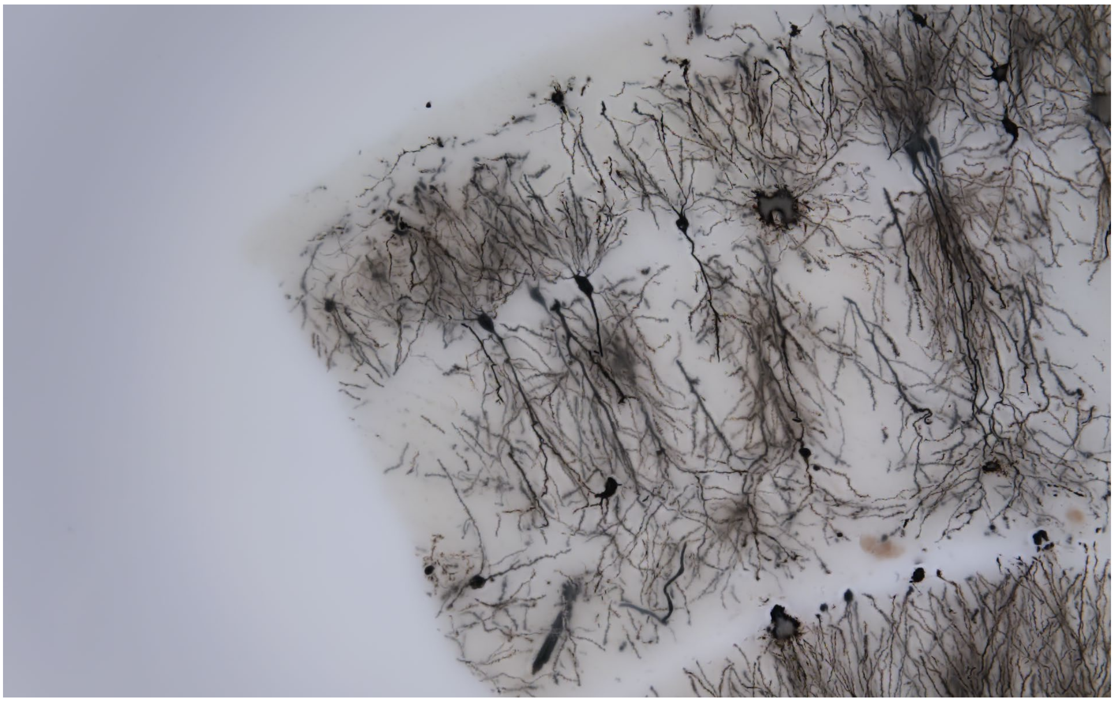

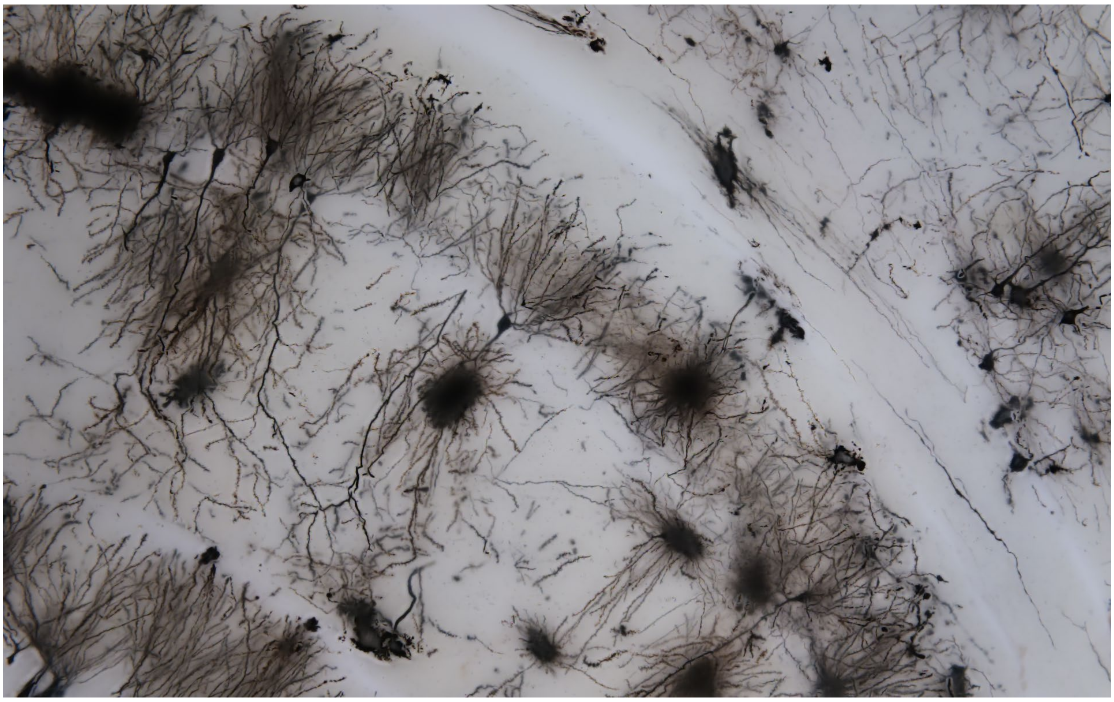

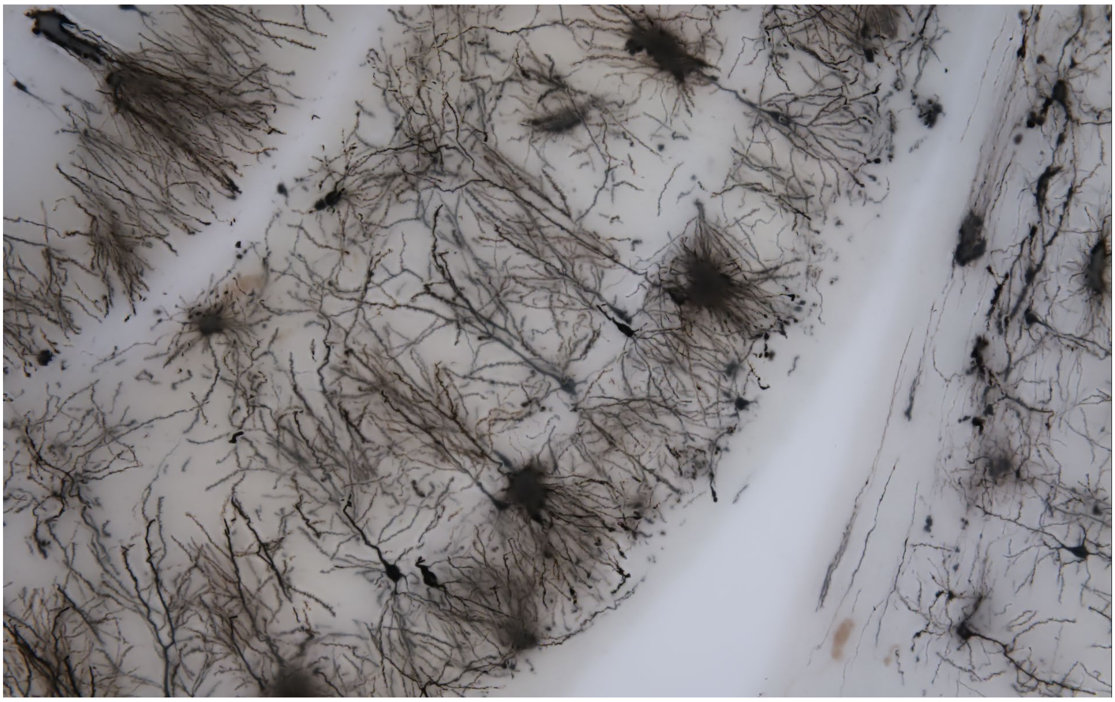
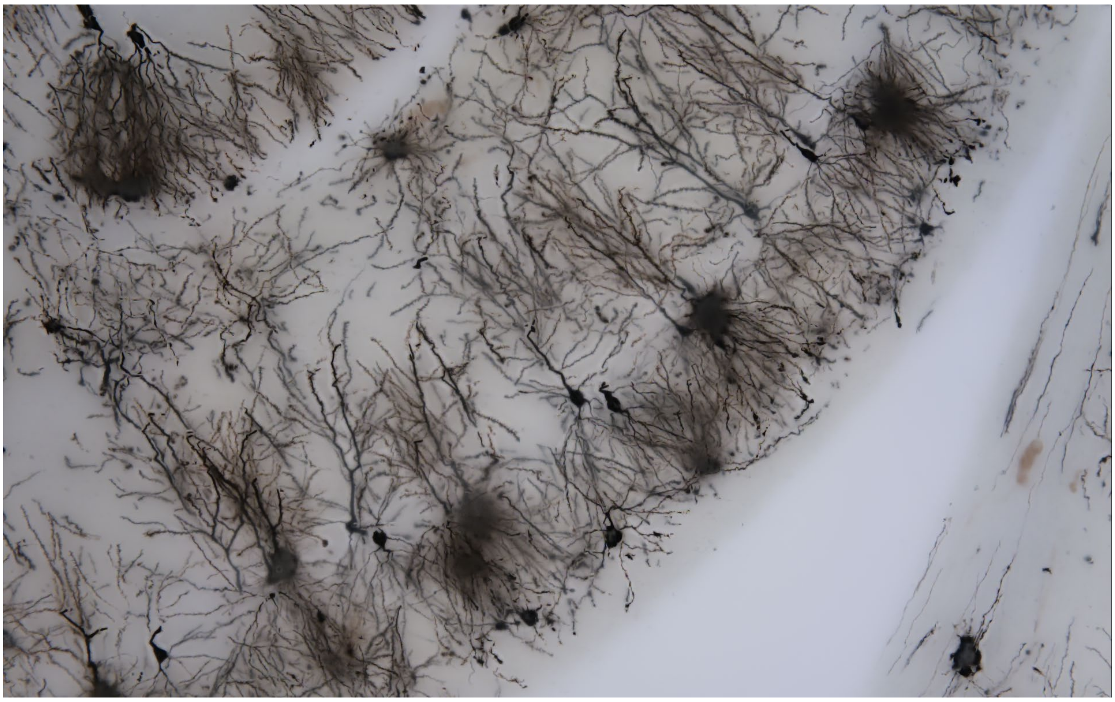

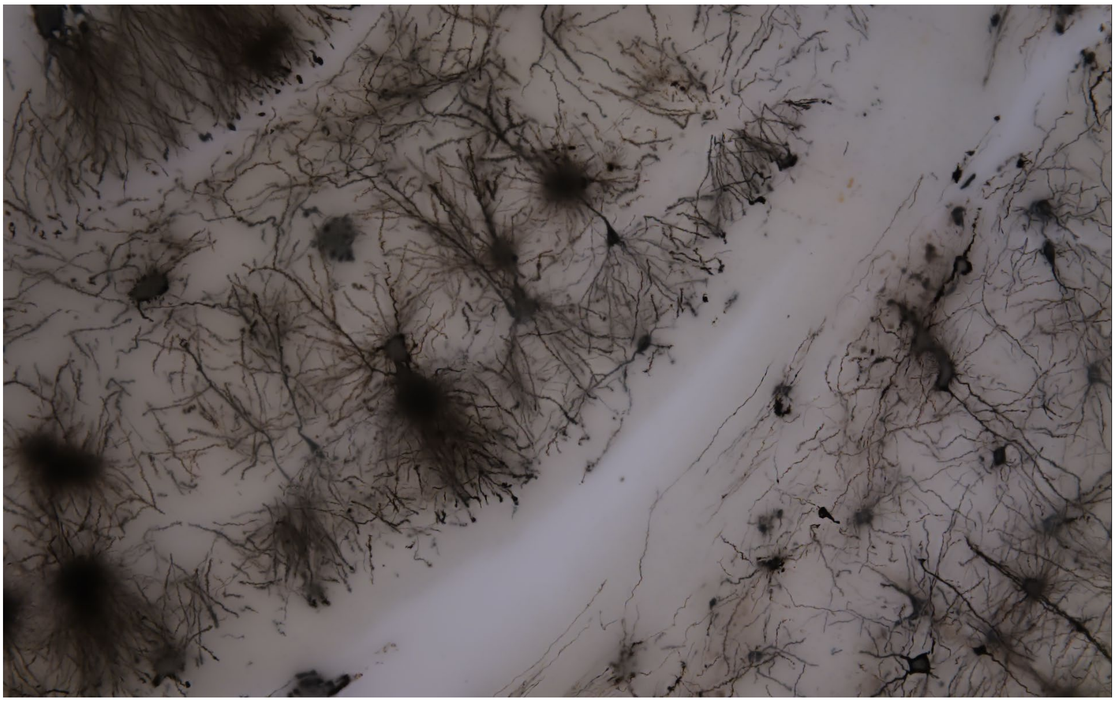
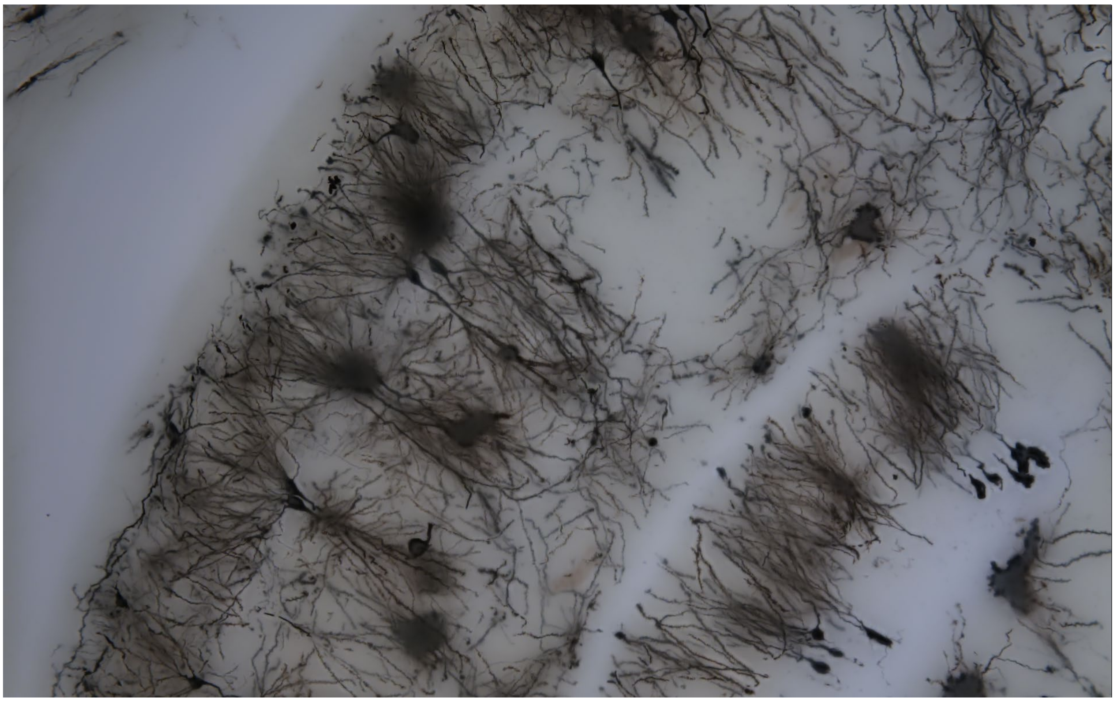

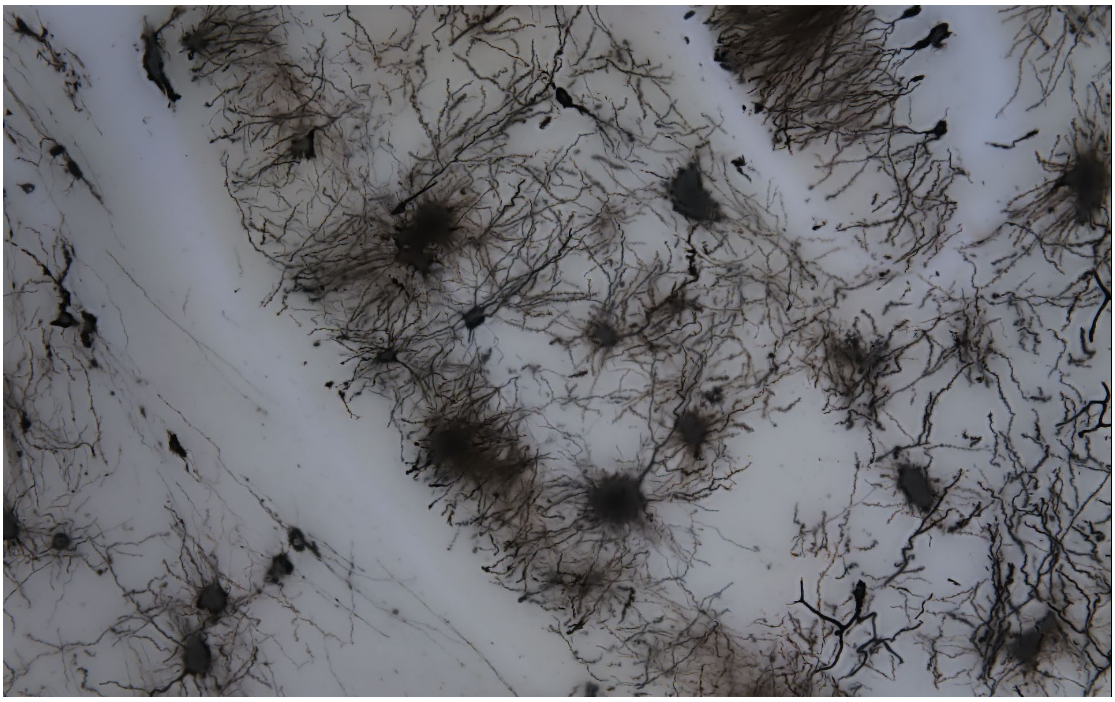

original pictures for apical spines of WT-PBS group in Figure 4G and H

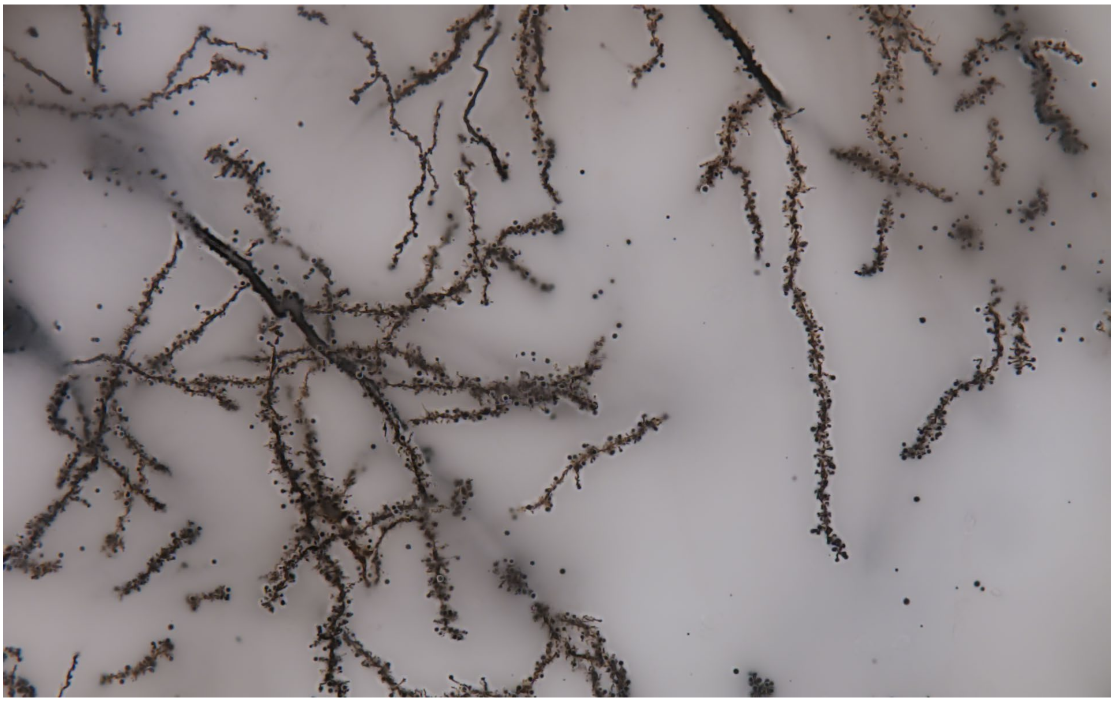

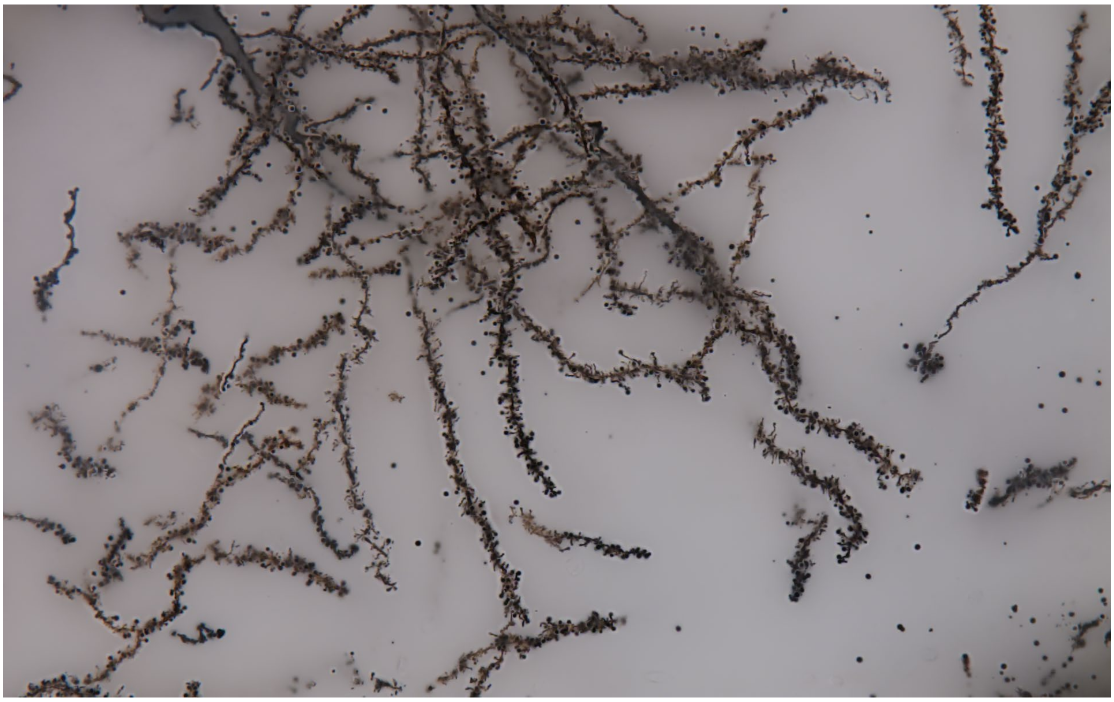

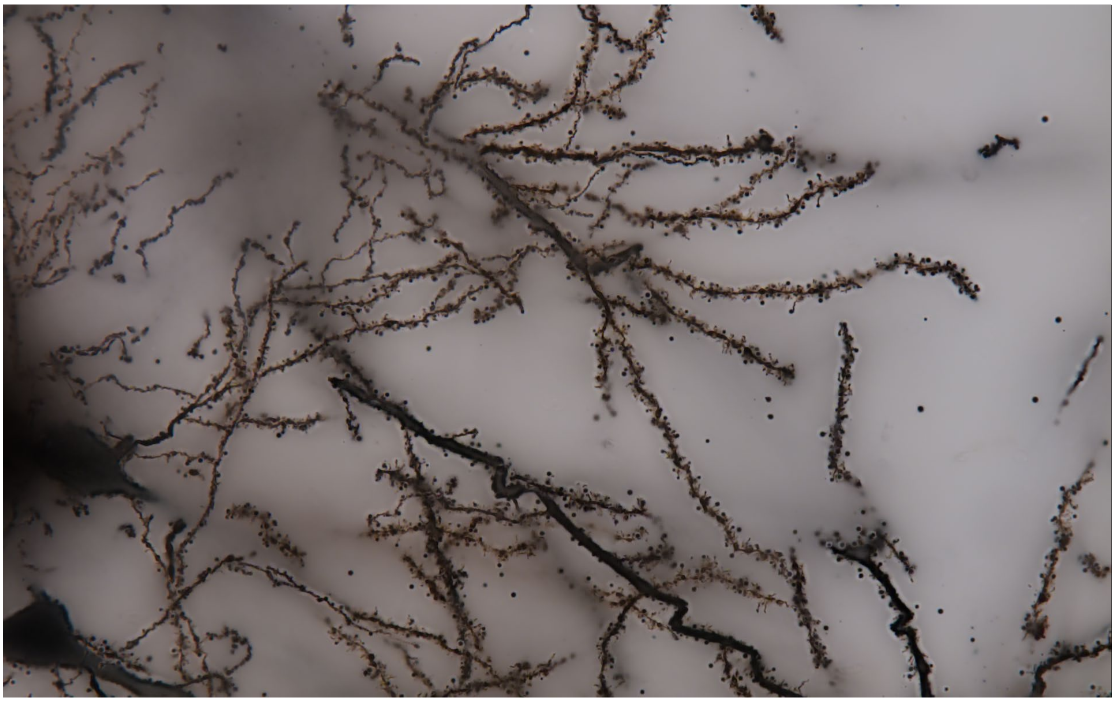

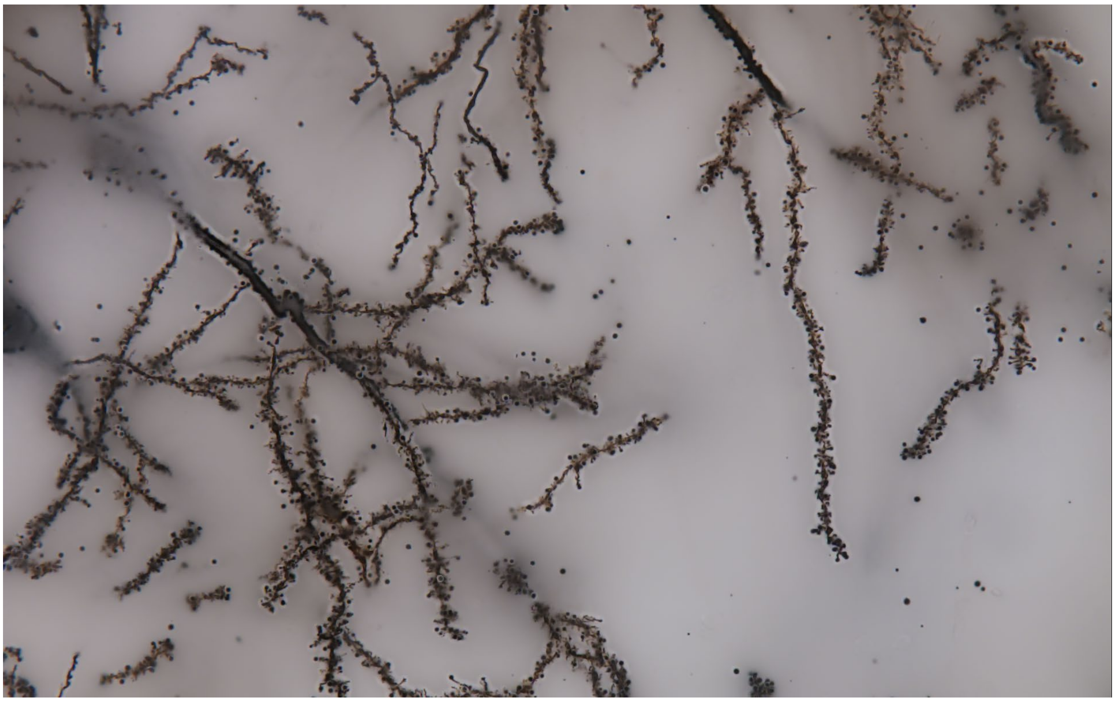
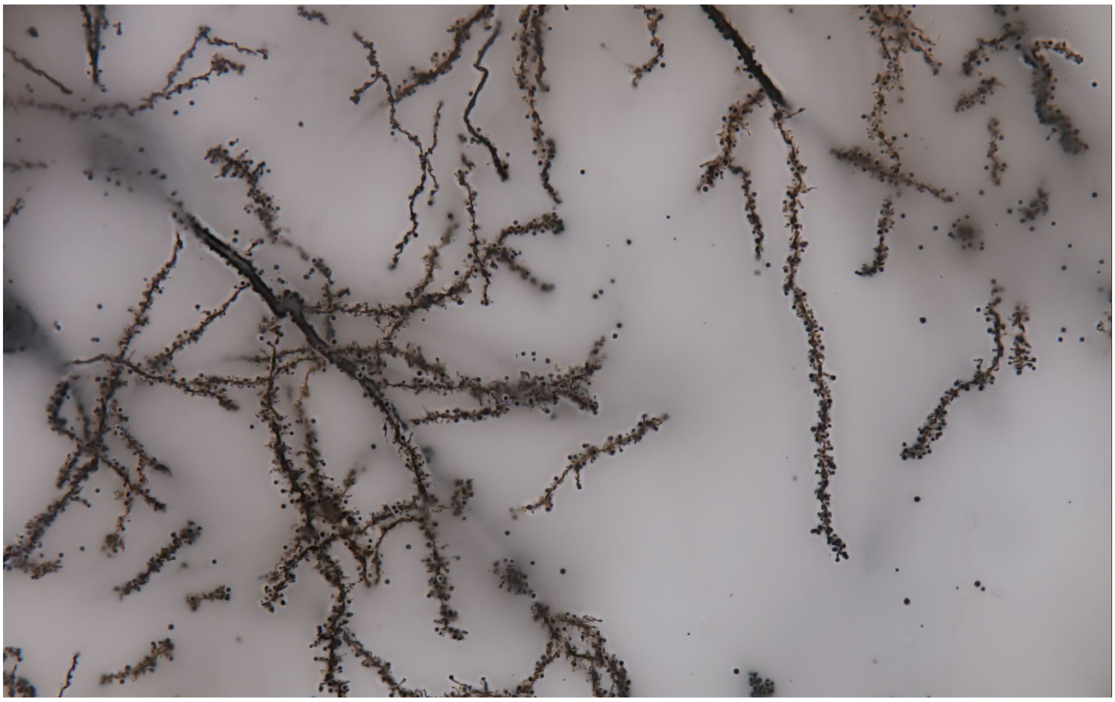

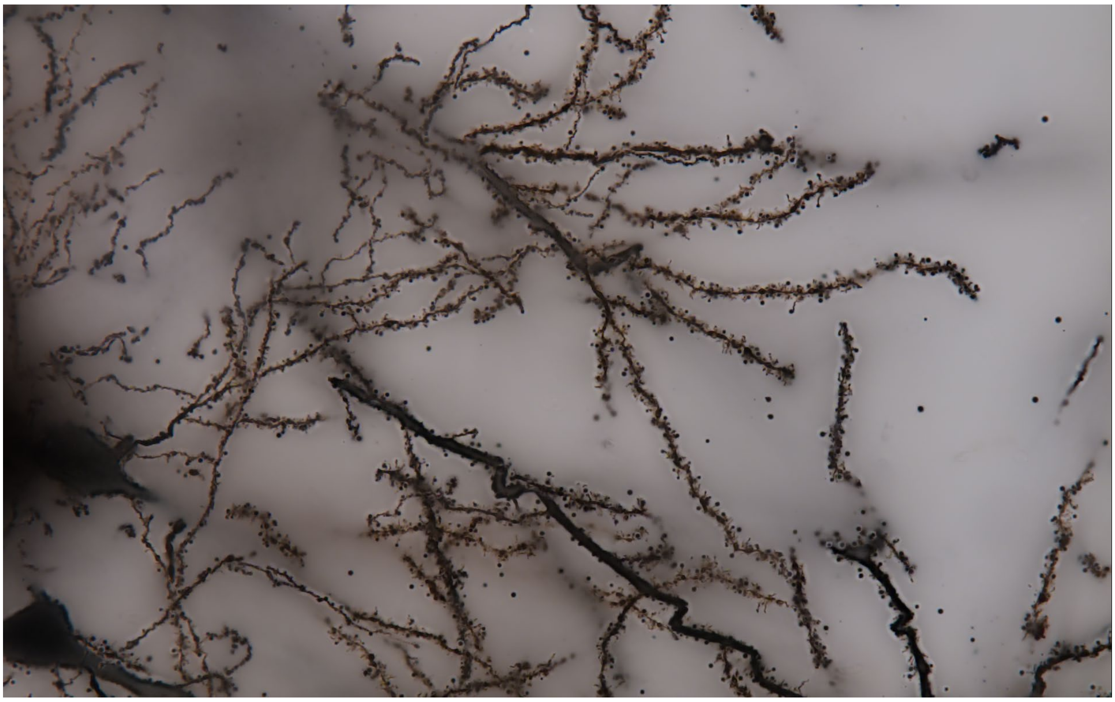
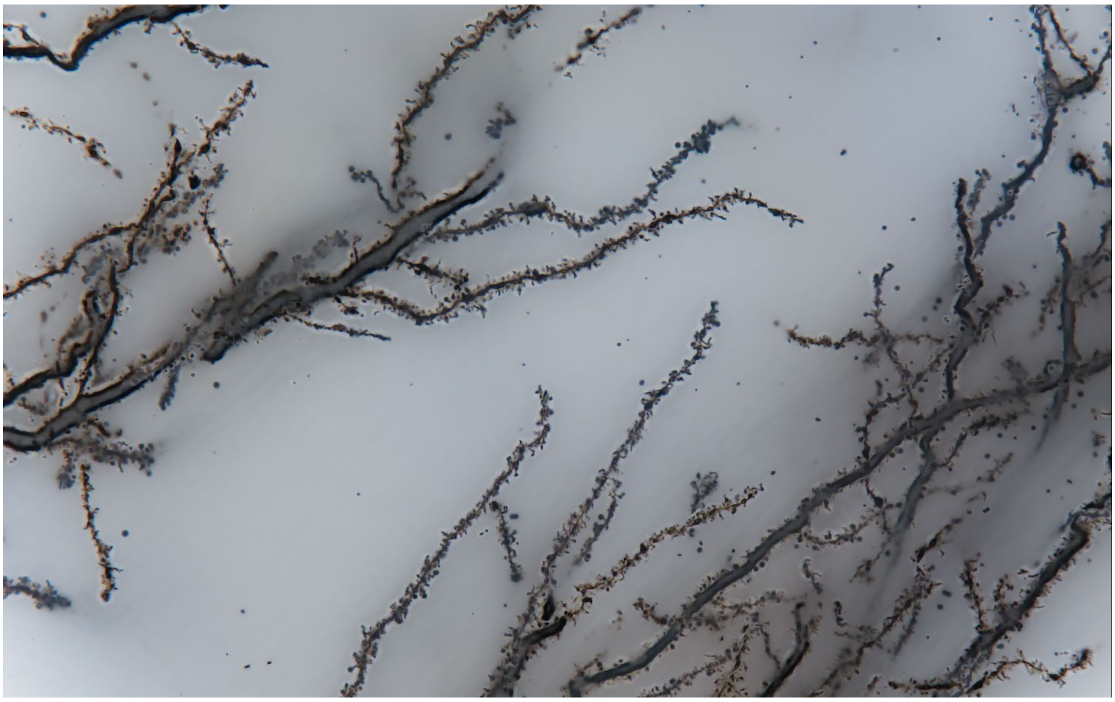

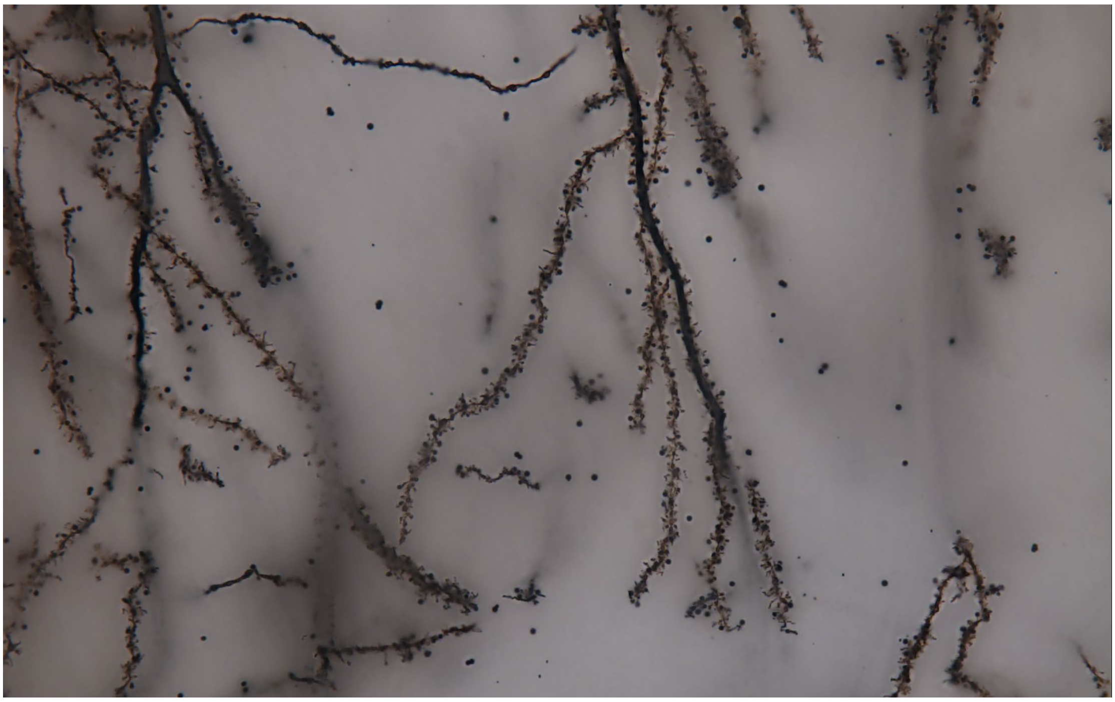

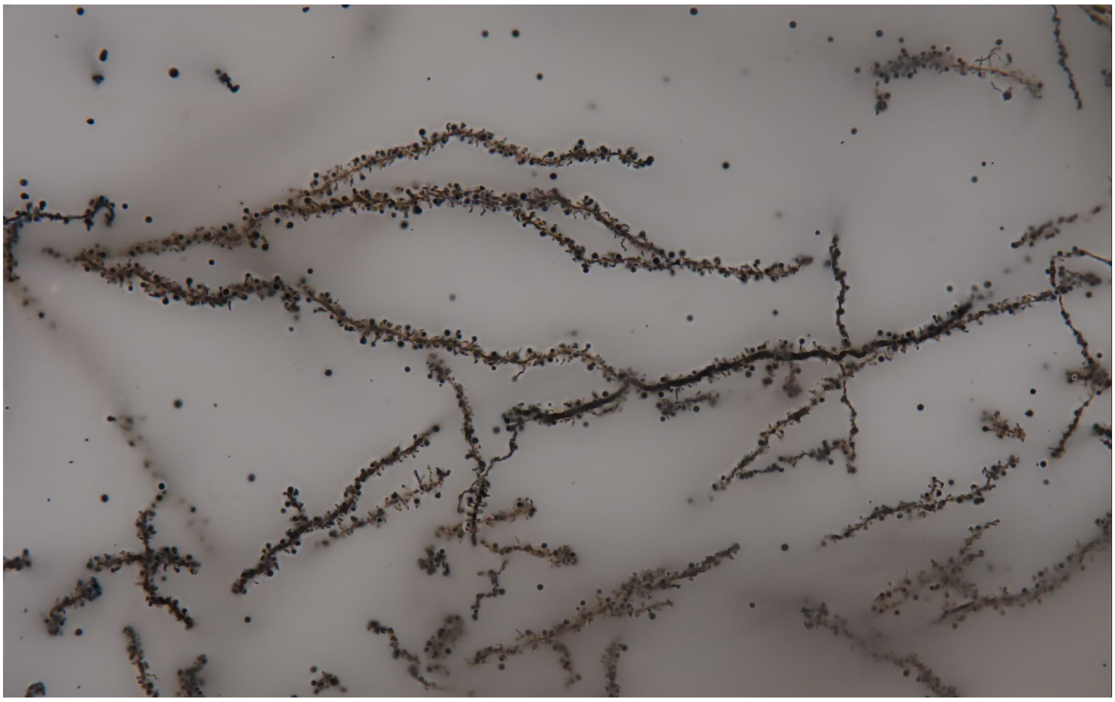

original pictures for apical spines of WT-Aβ group in Figure 4G and H

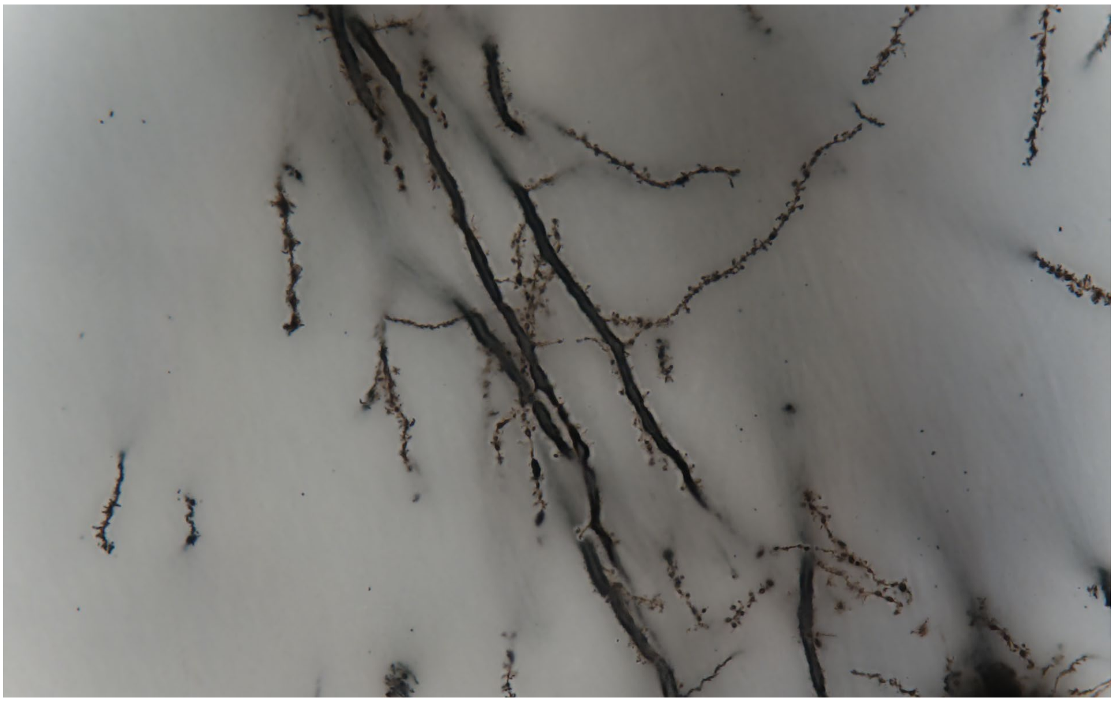

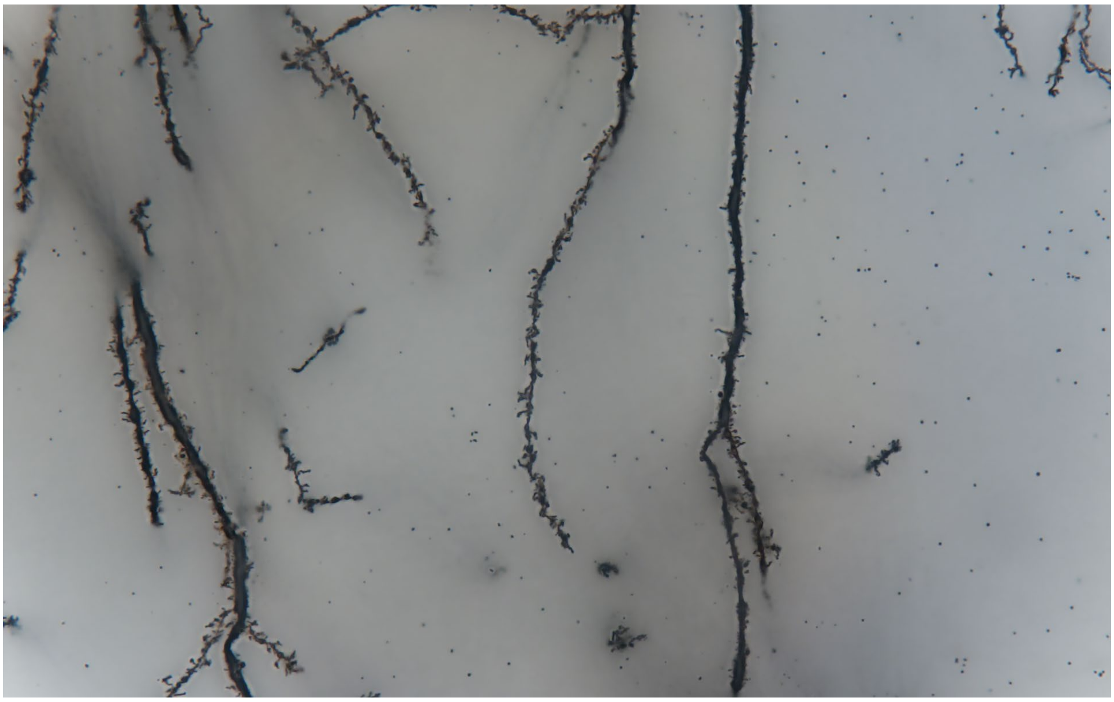

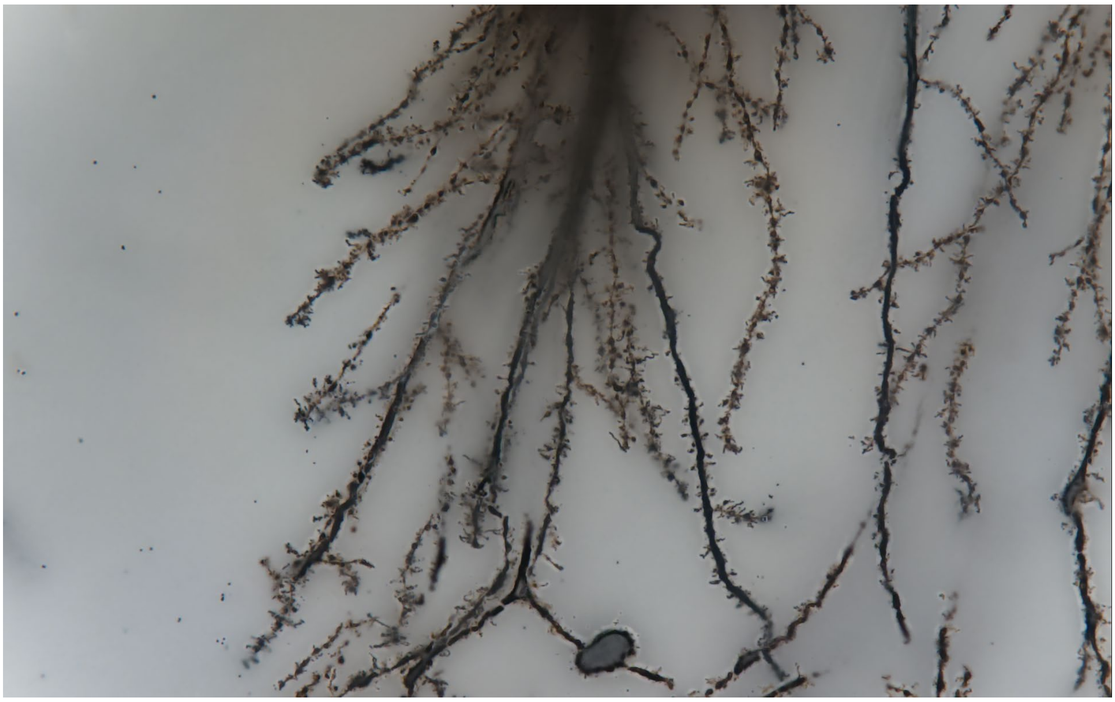

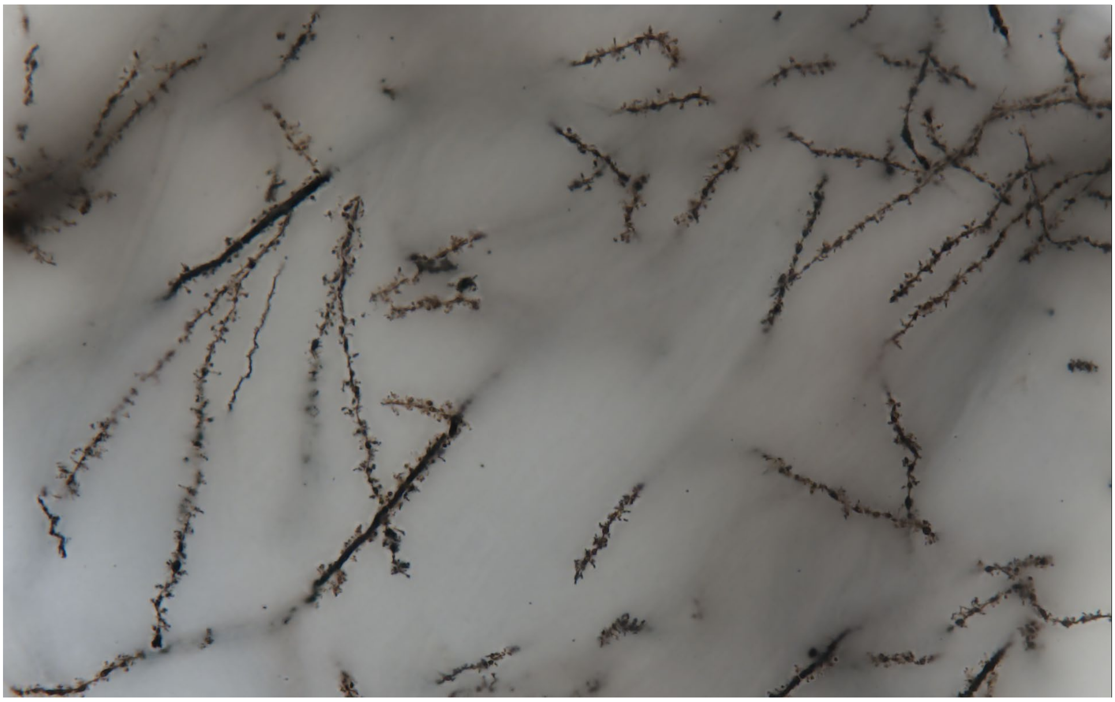

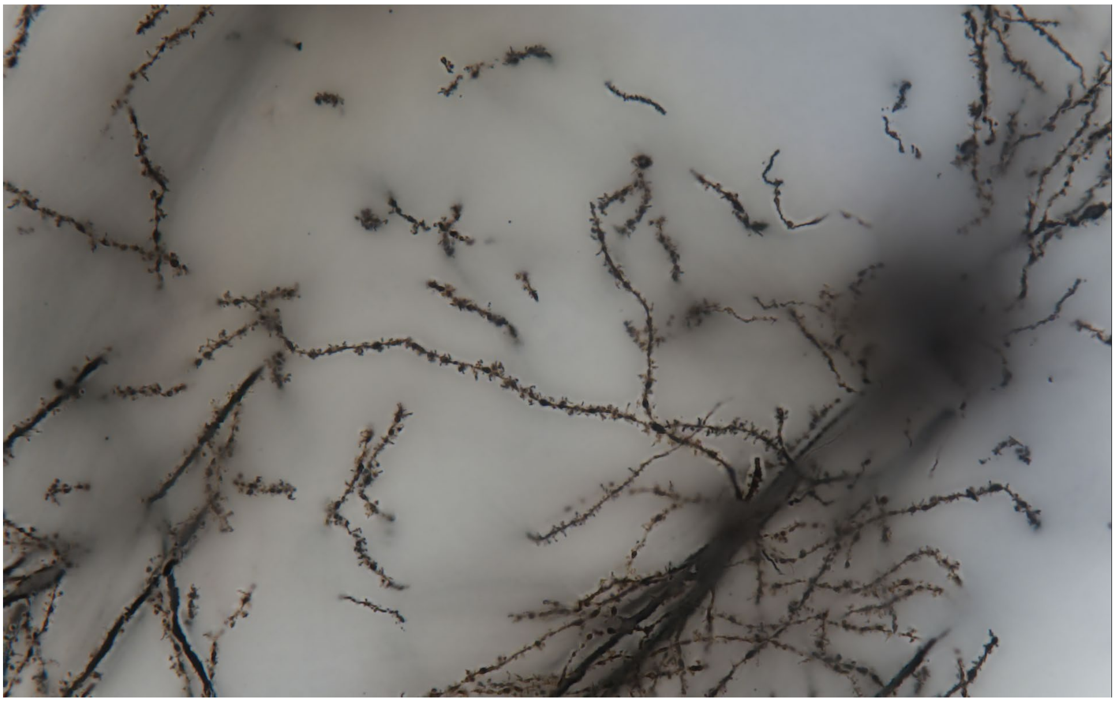

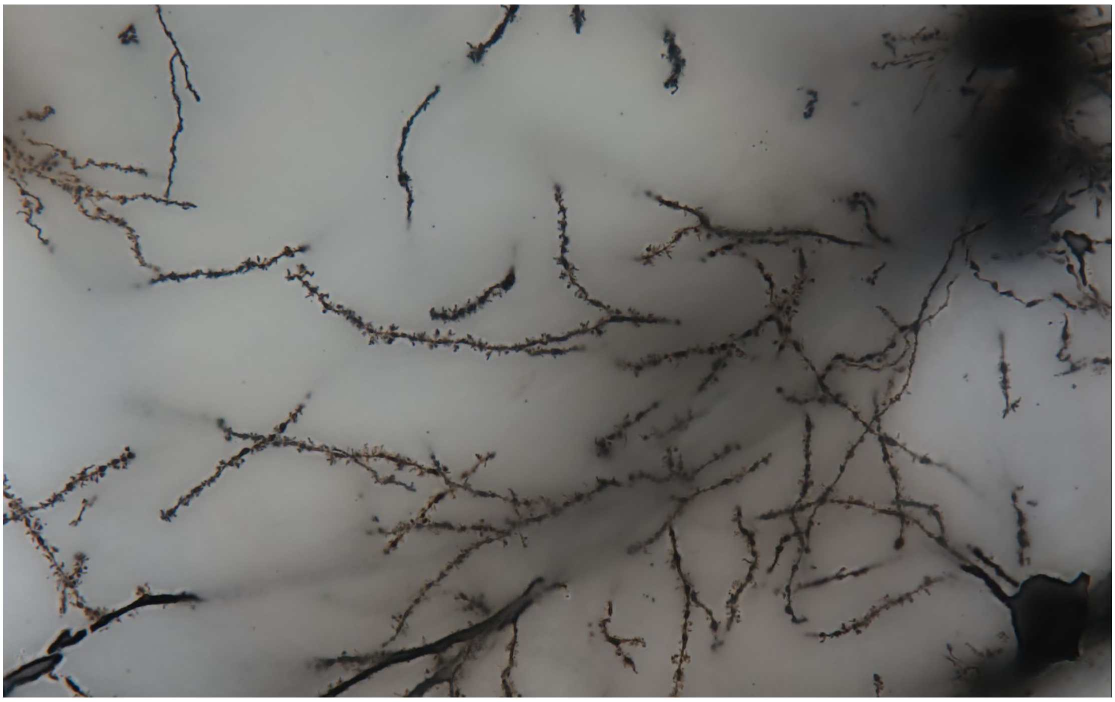

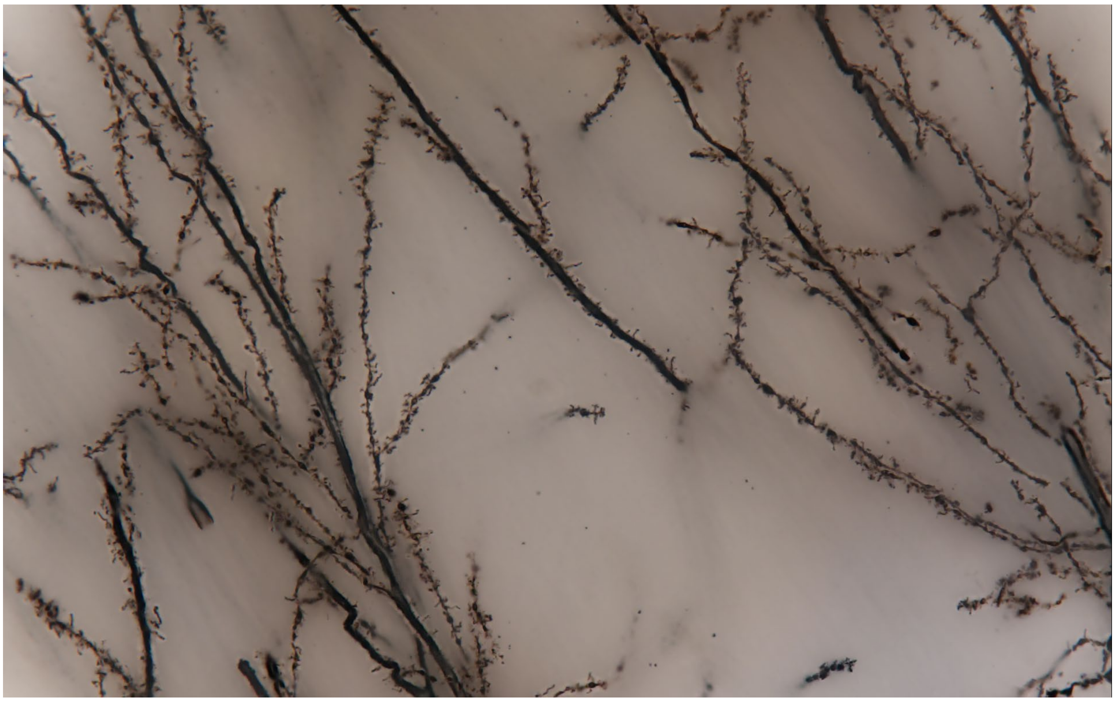
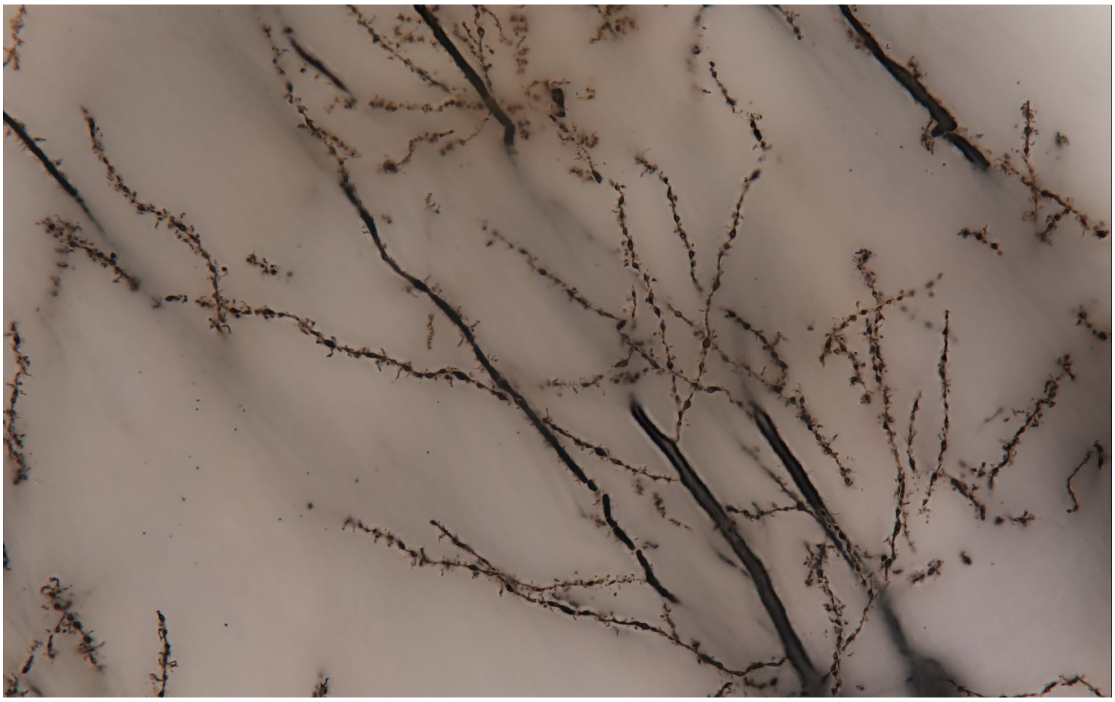

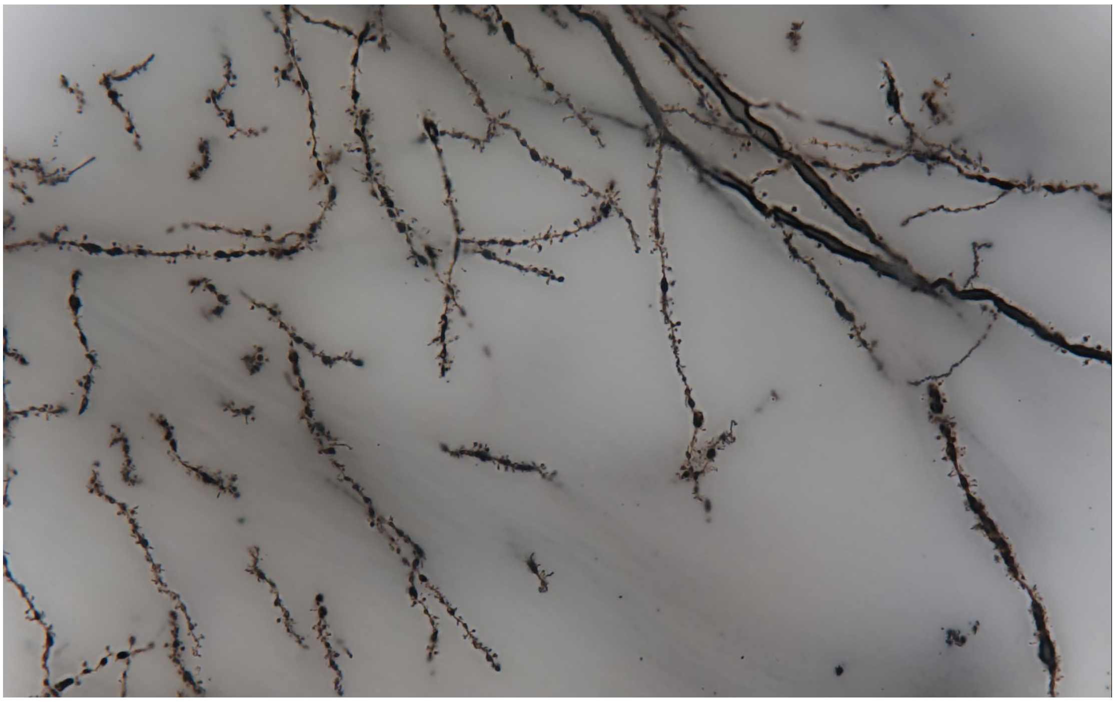
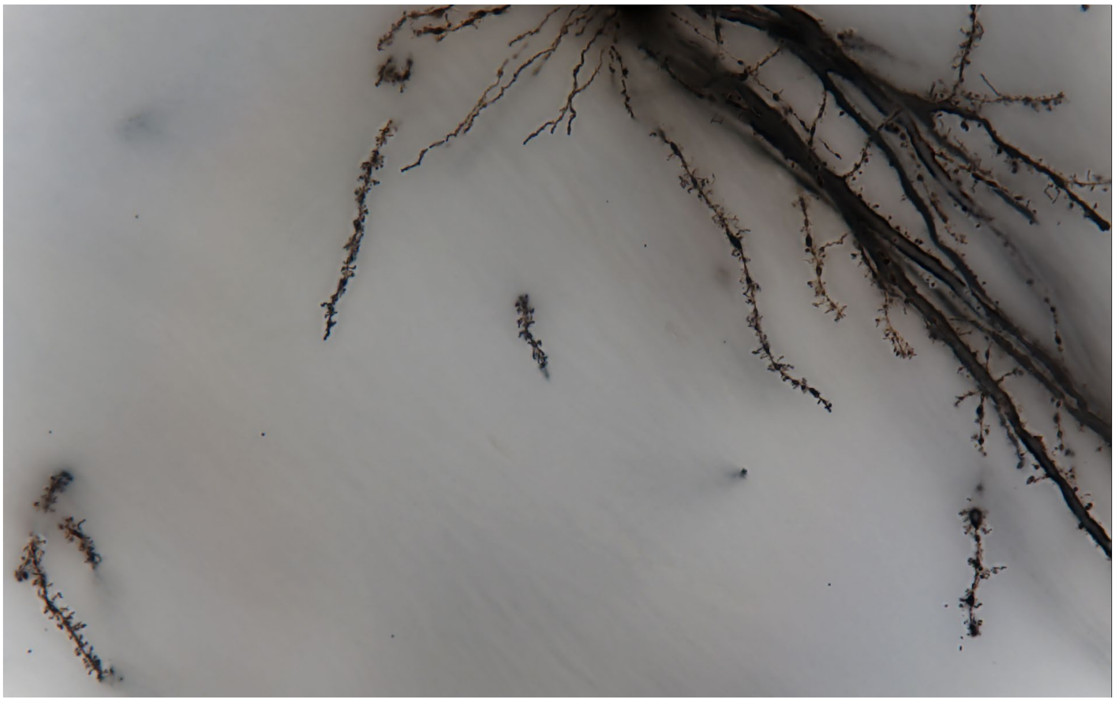

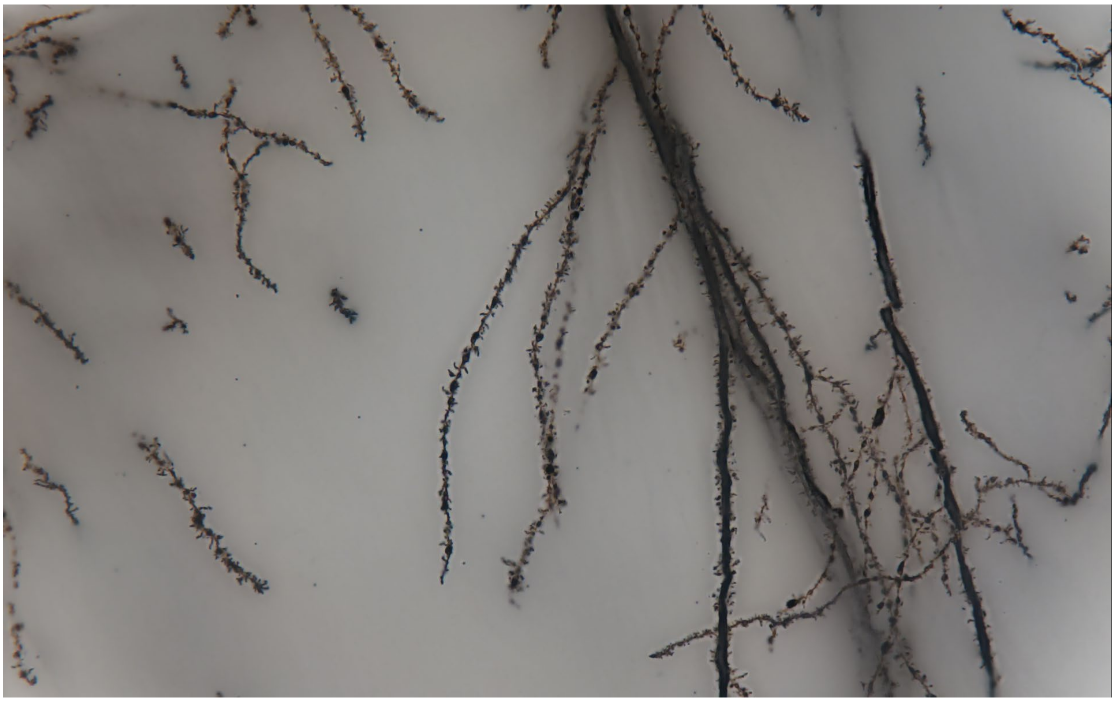

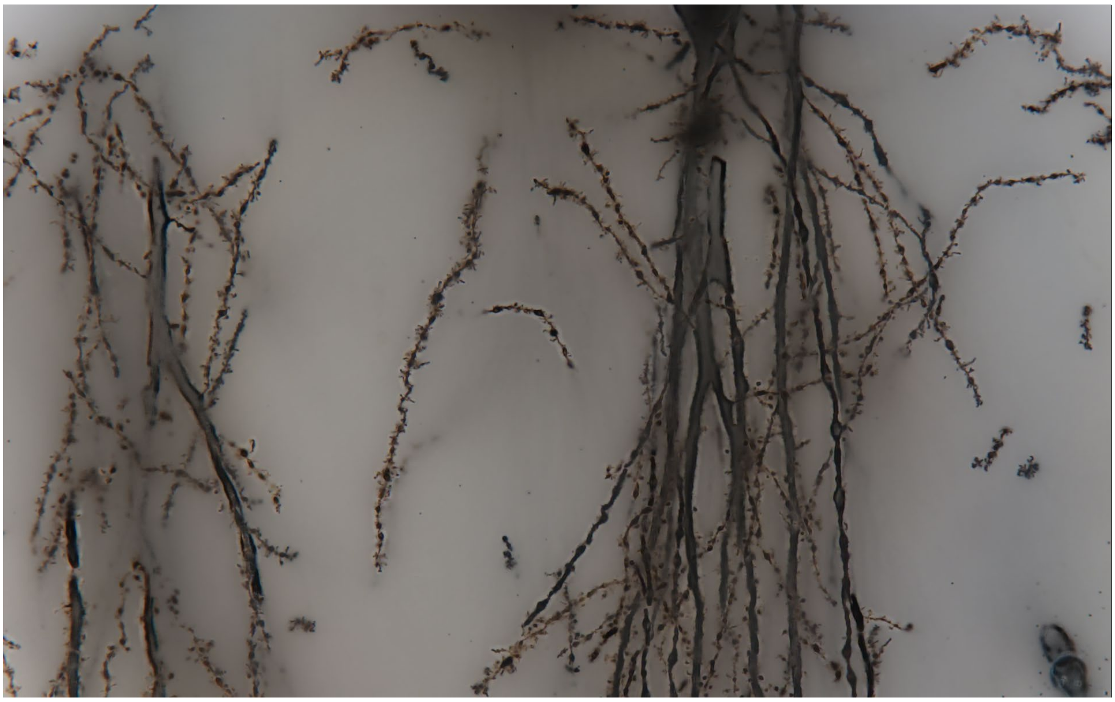

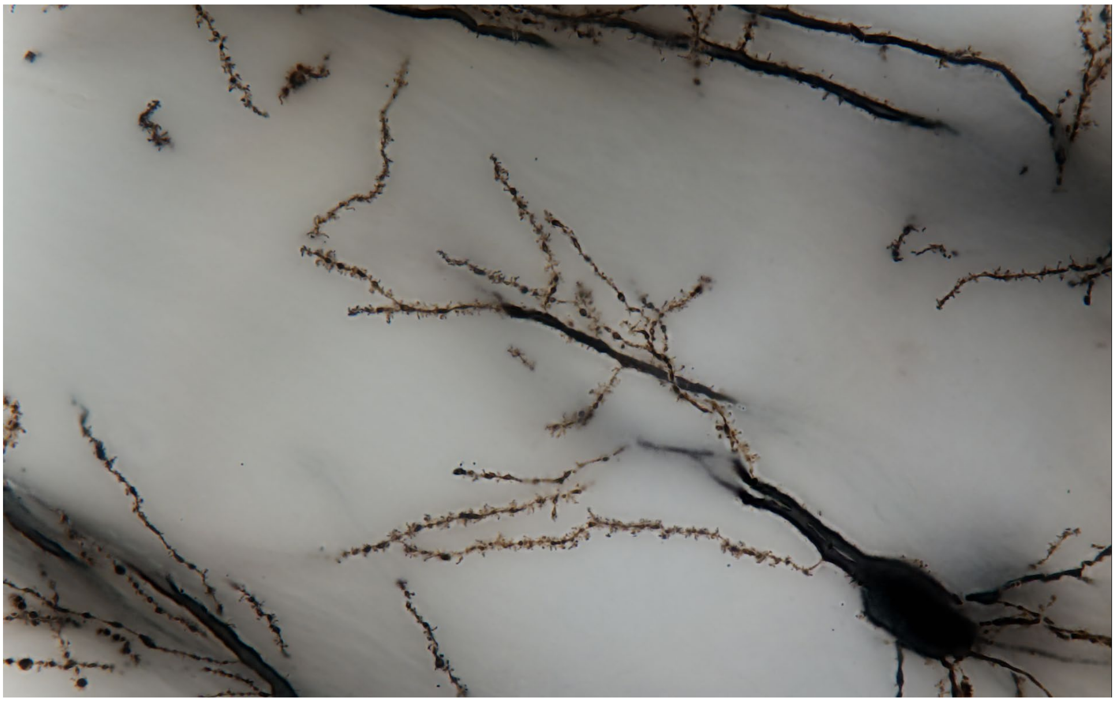
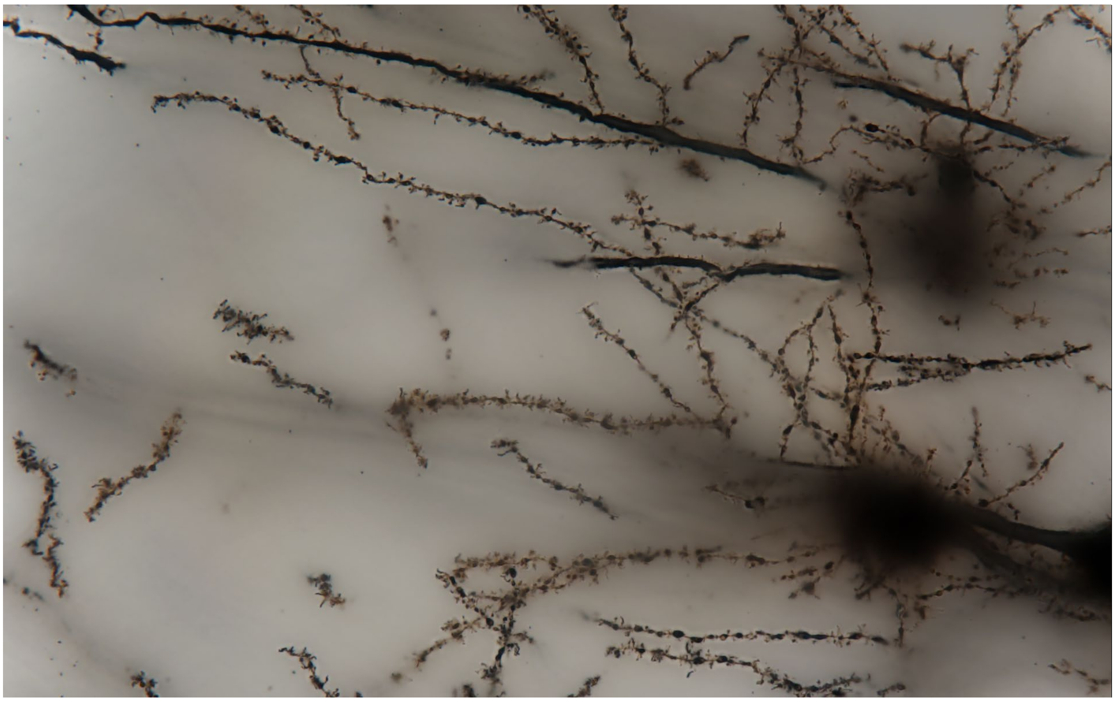

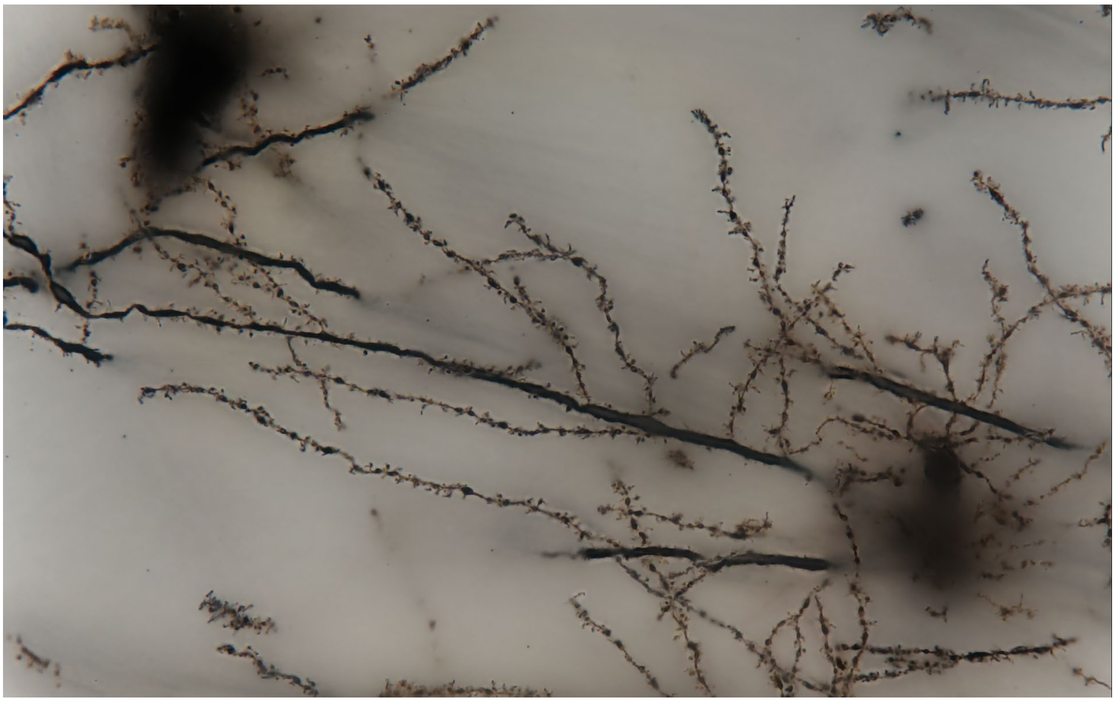

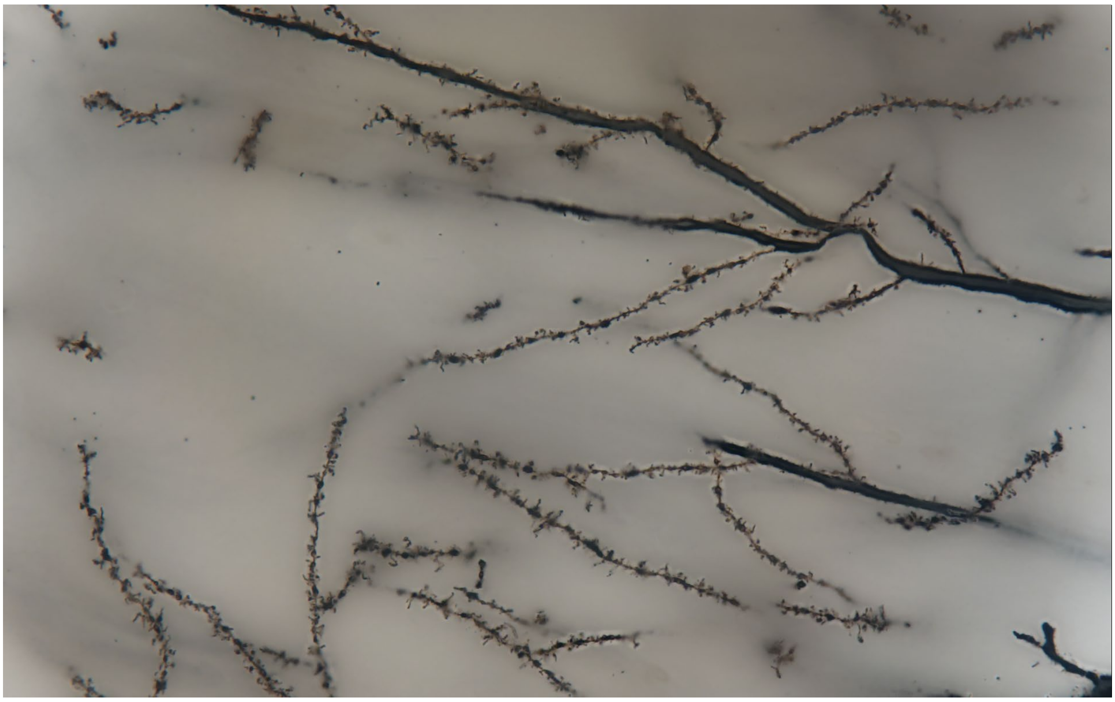

original pictures for apical spines of cKO-Aβ group in Figure 4G and H

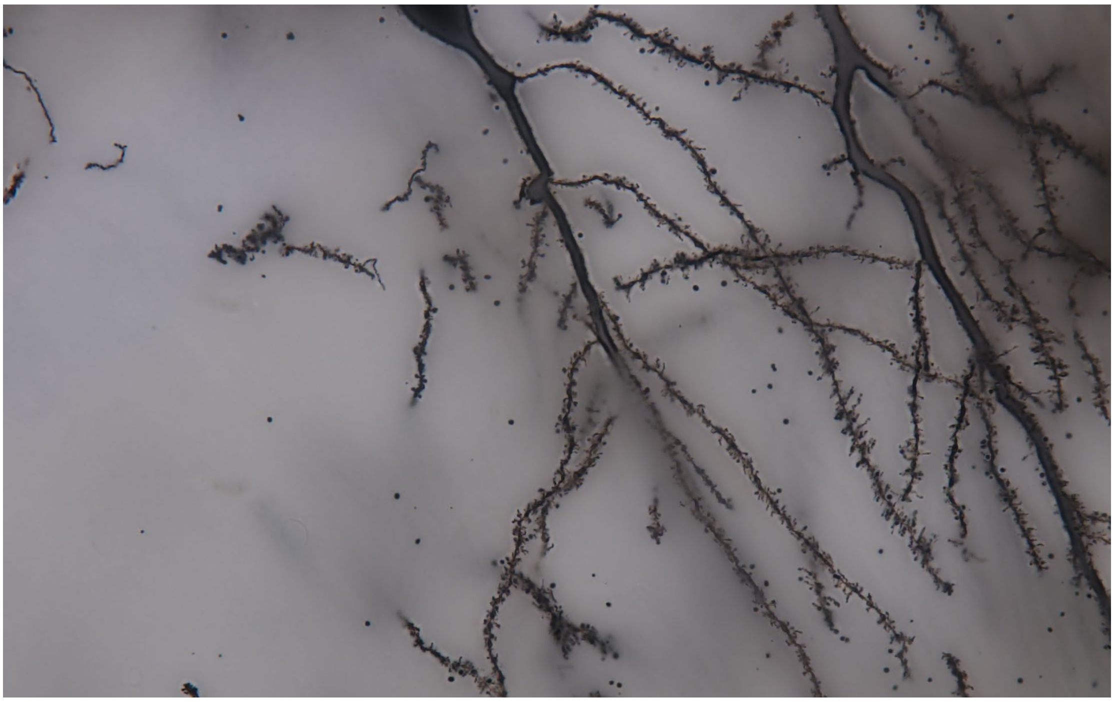

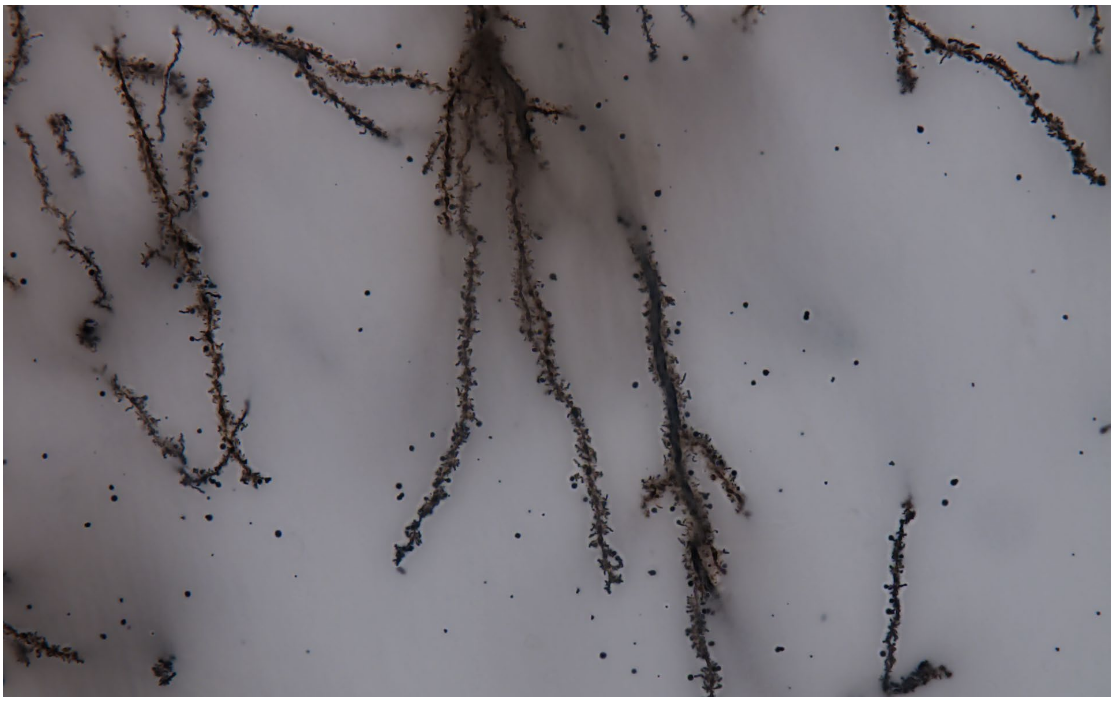

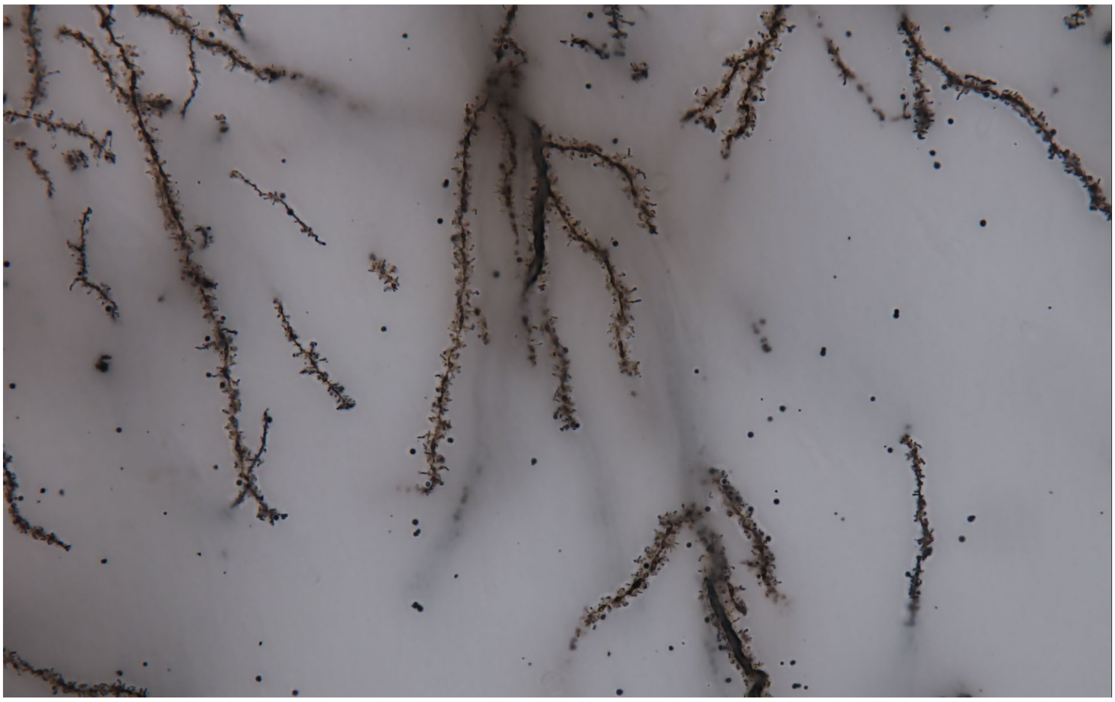

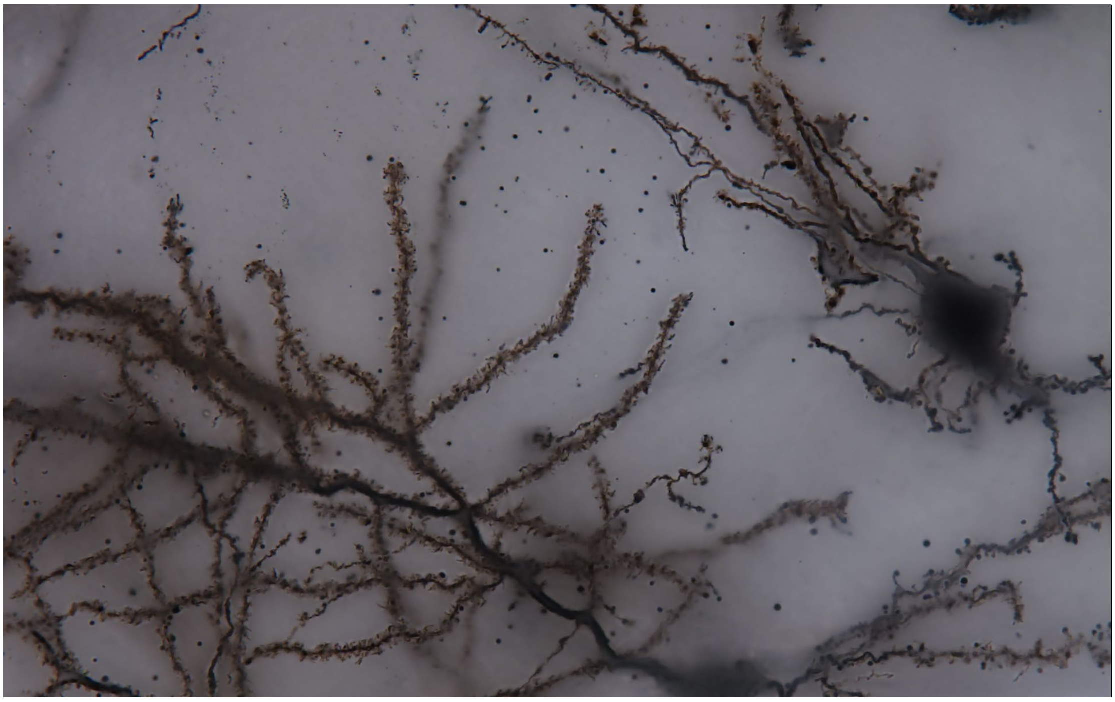

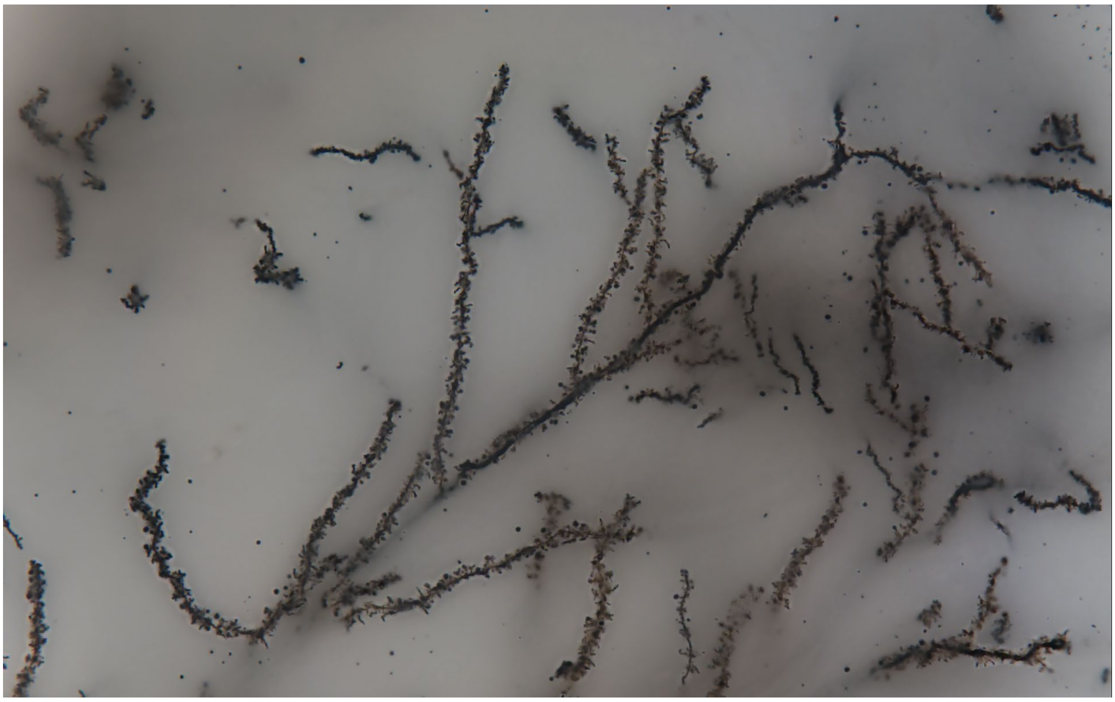
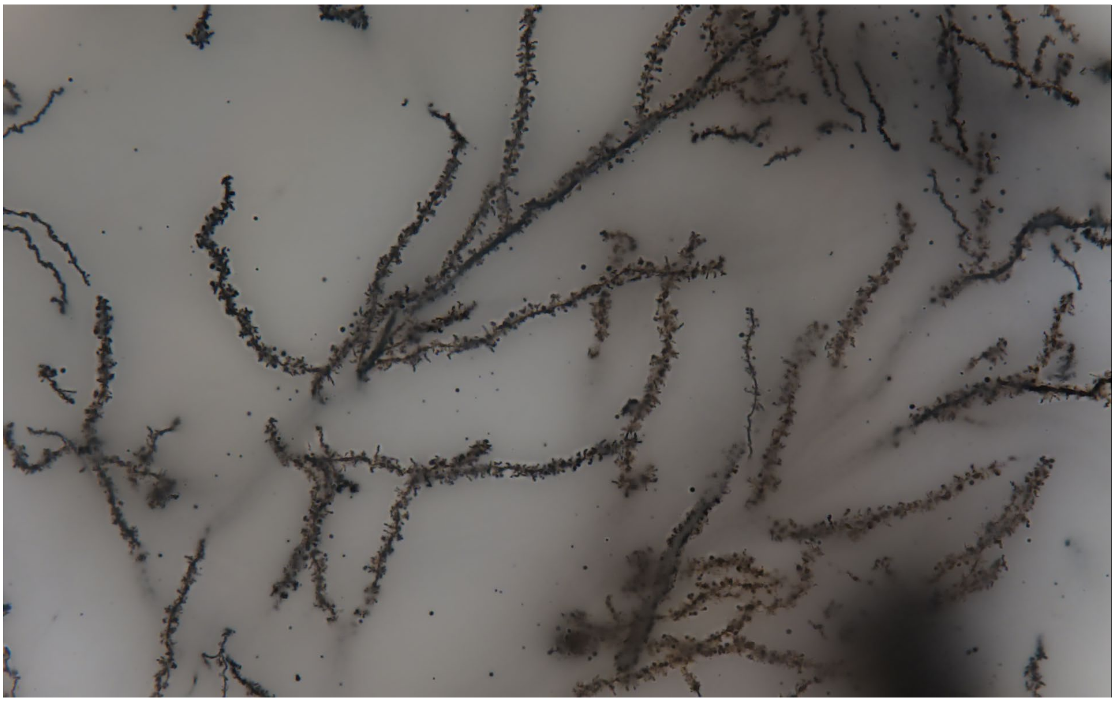

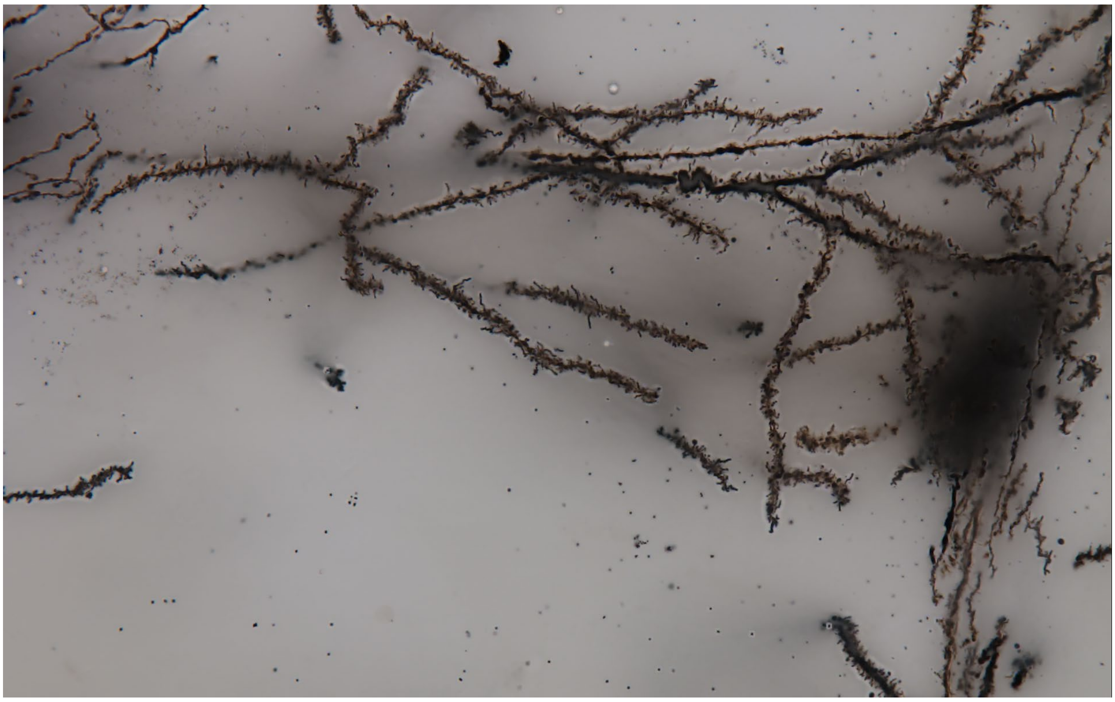

original pictures for basal spines of WT-PBS group in Figure 4G and H

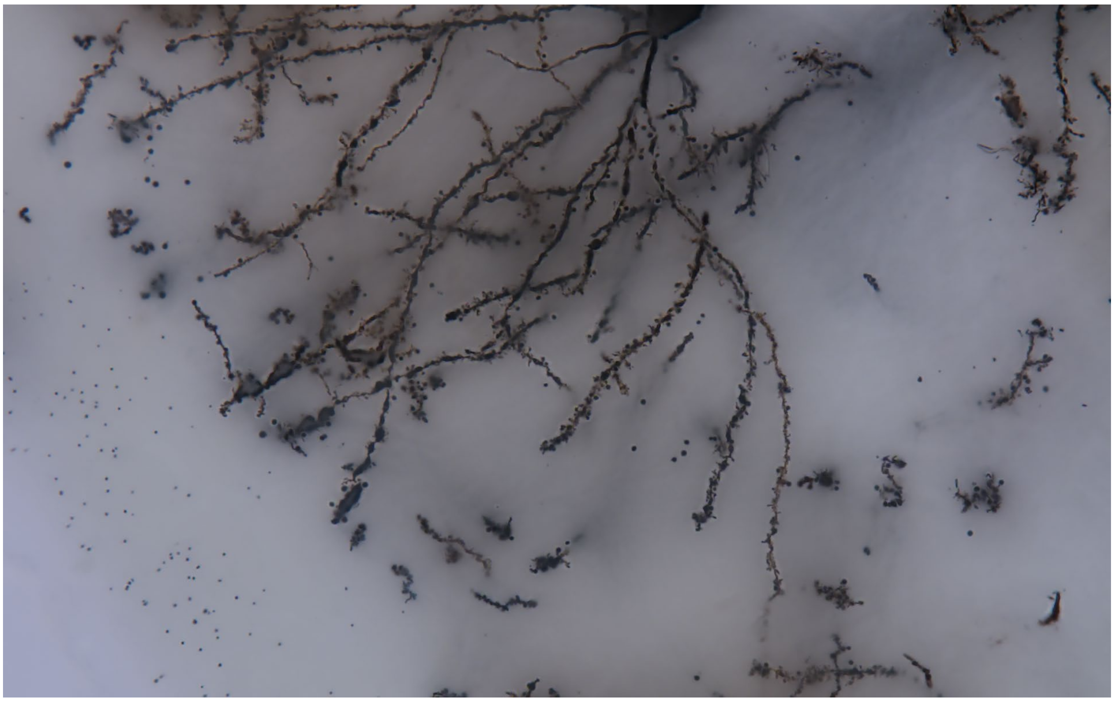

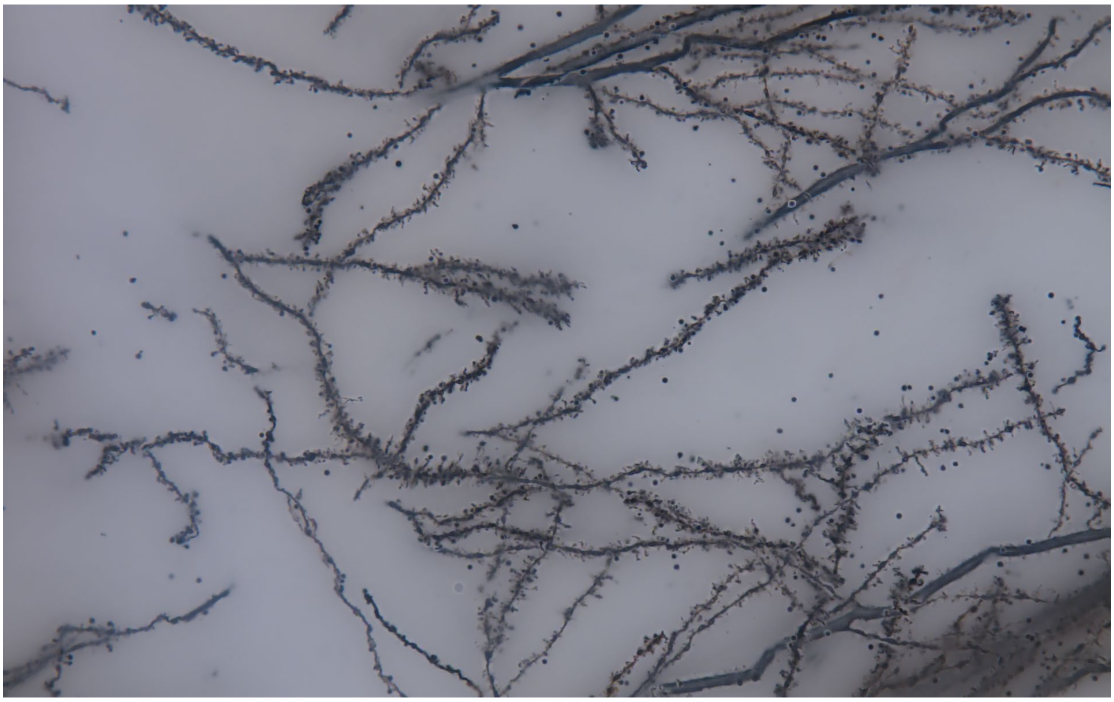

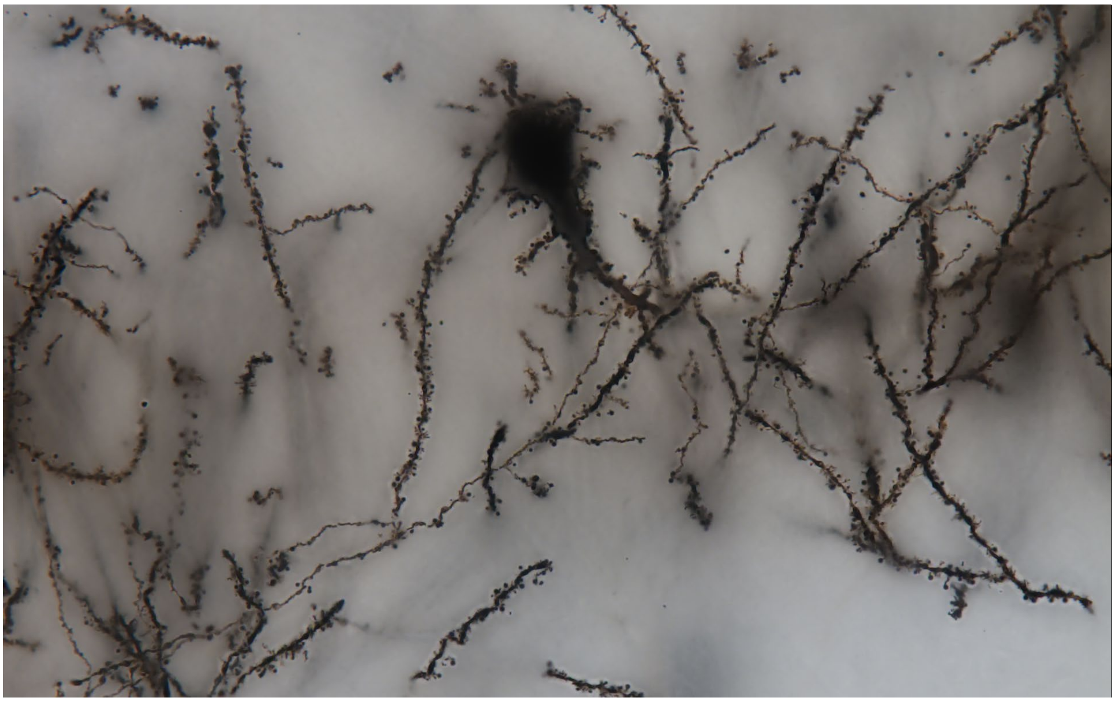

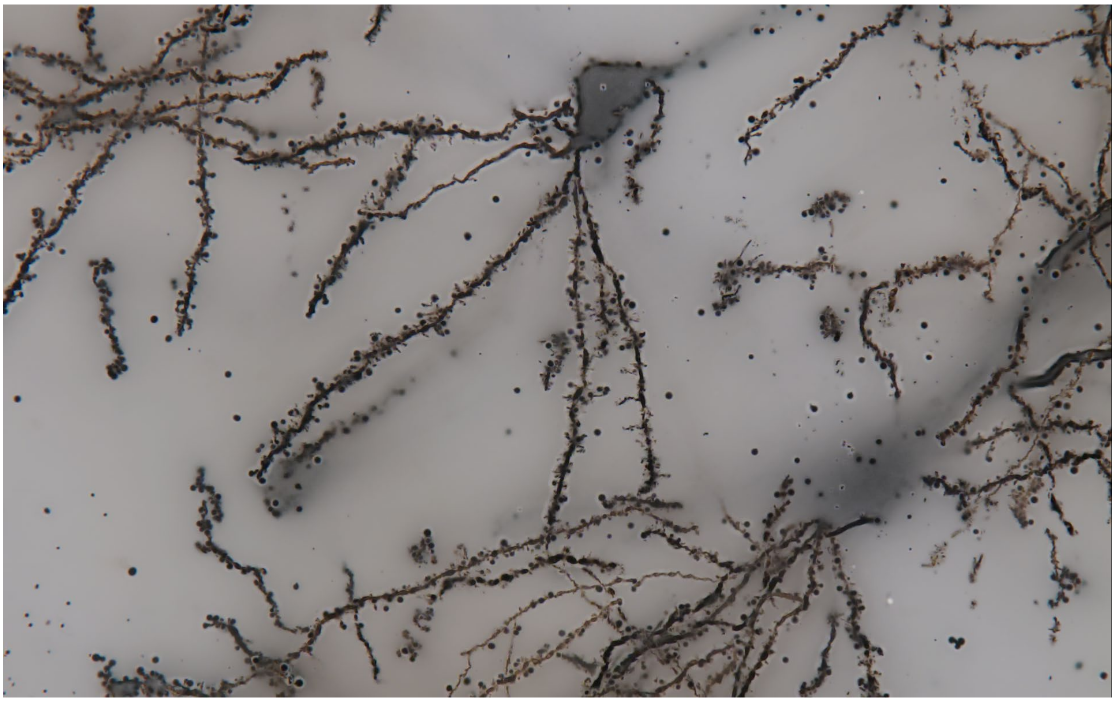

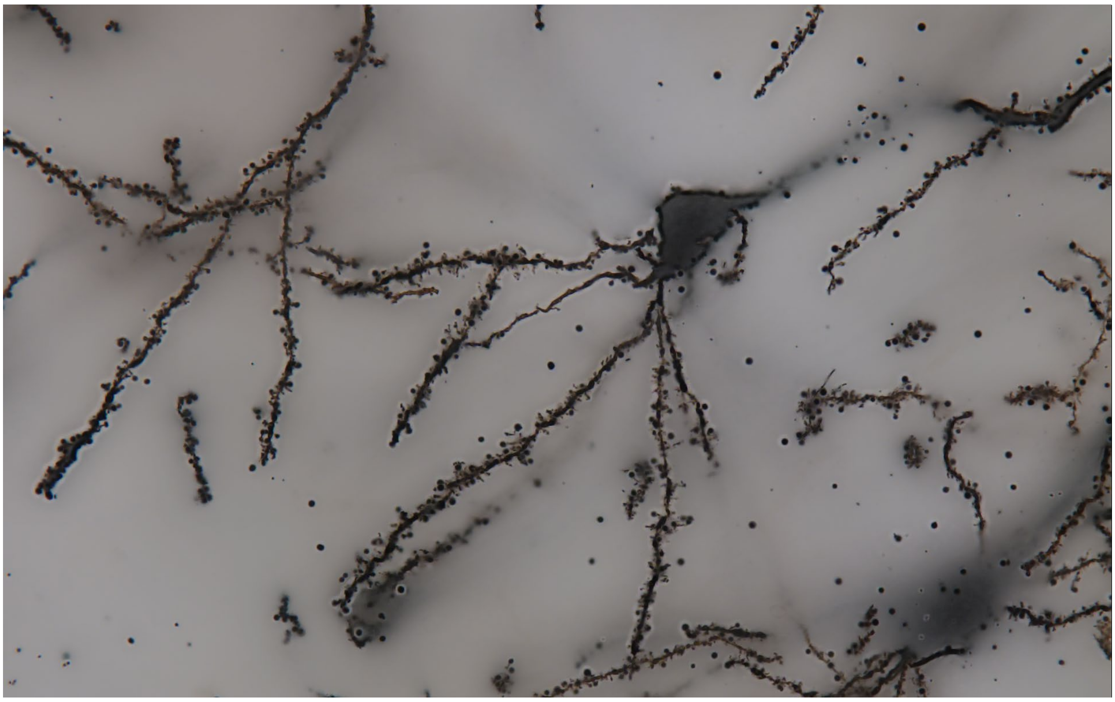

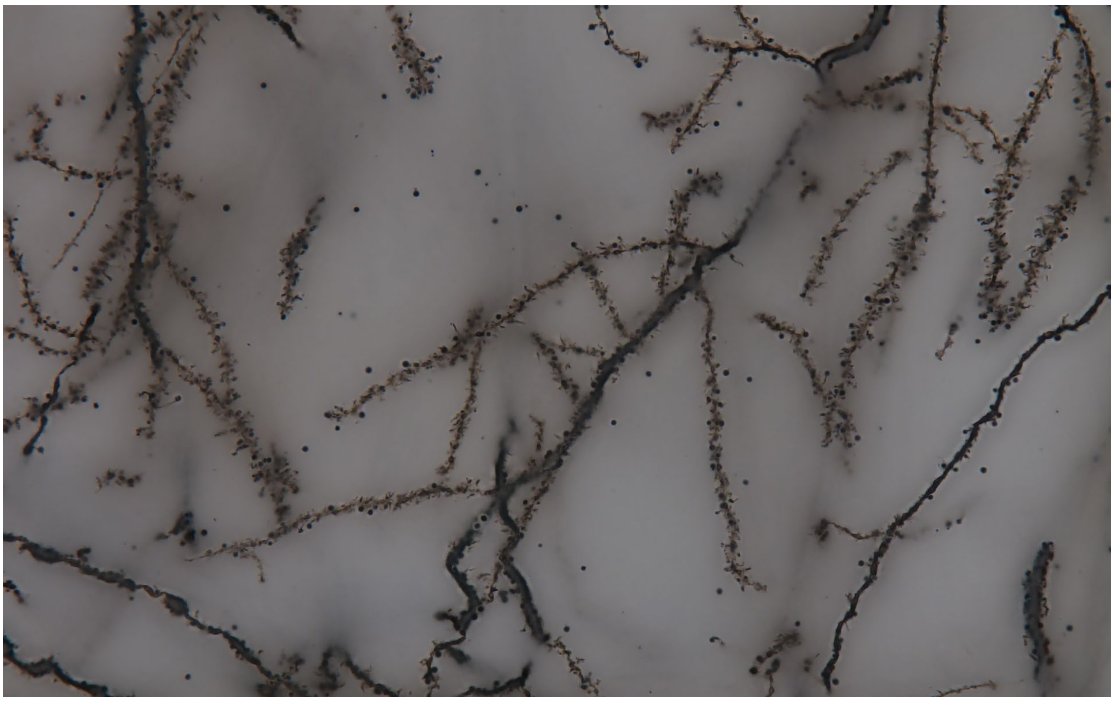

original pictures for basal spines of WT-Aβ group in Figure 4G and H

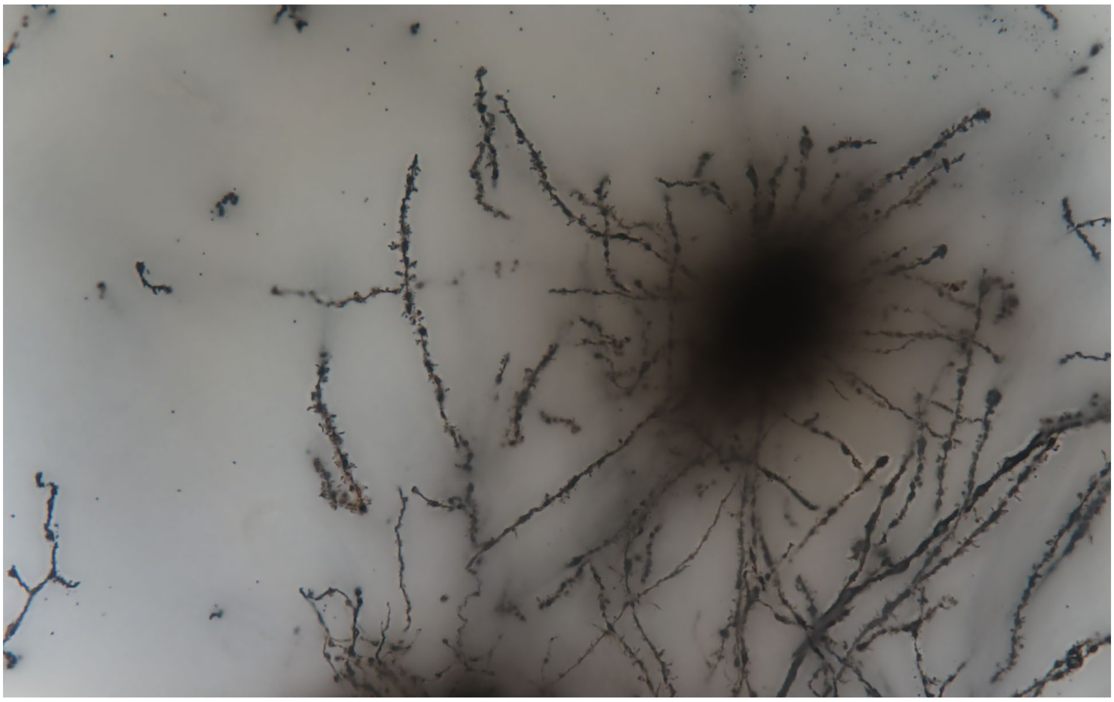

-

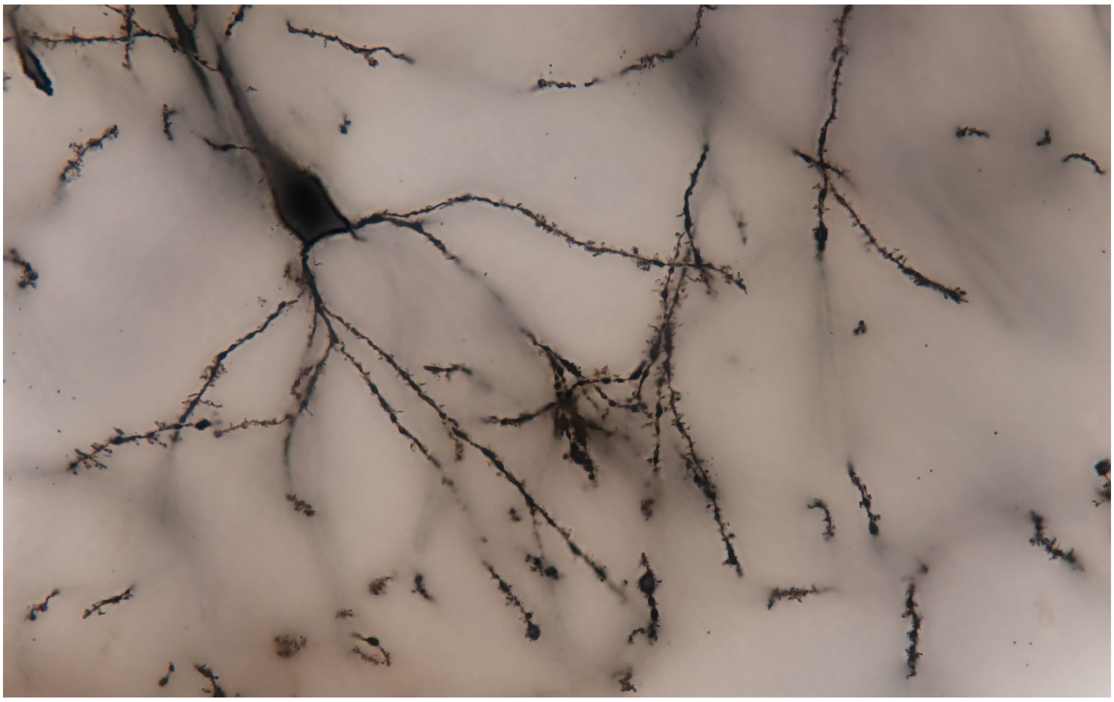

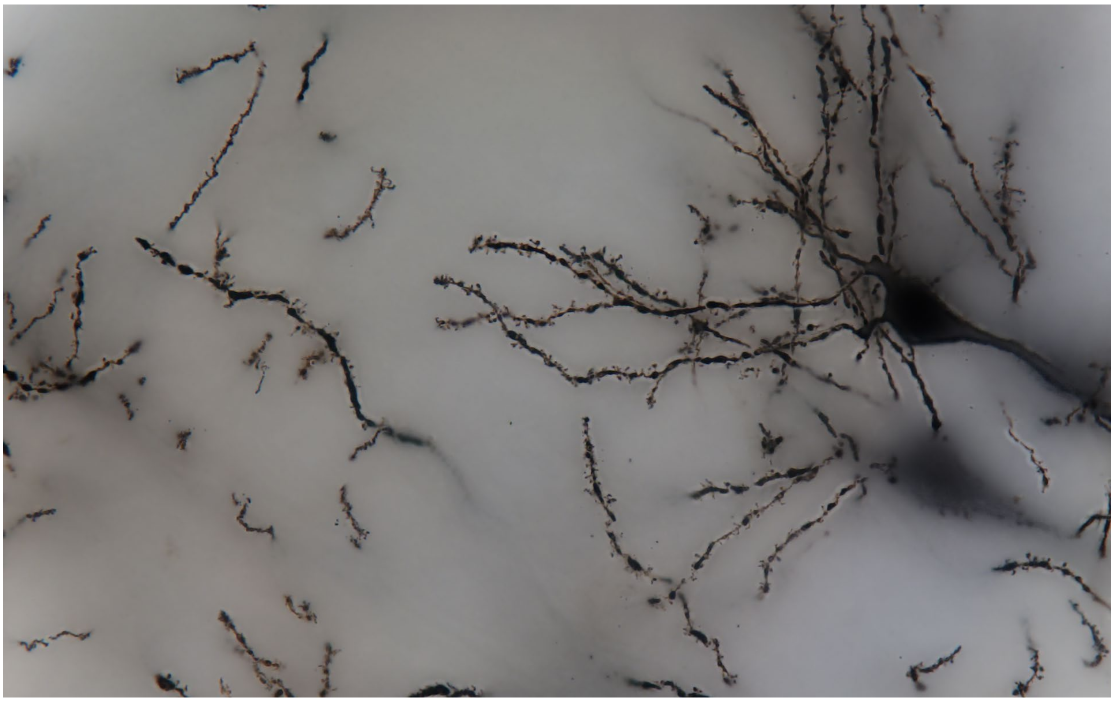

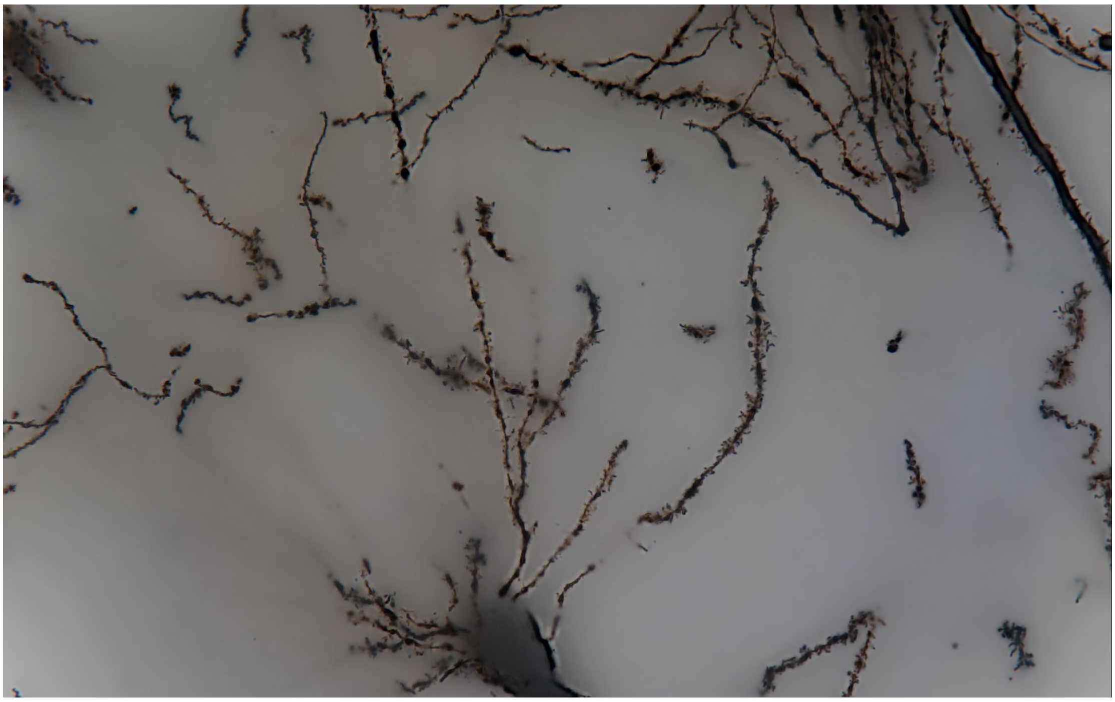

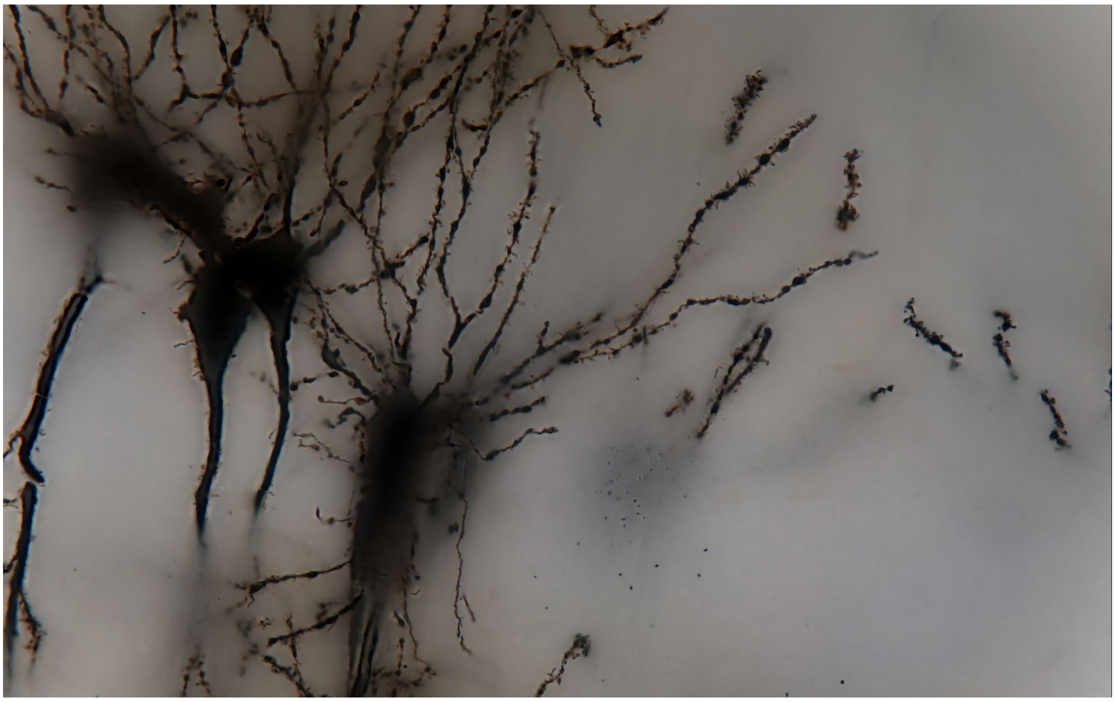

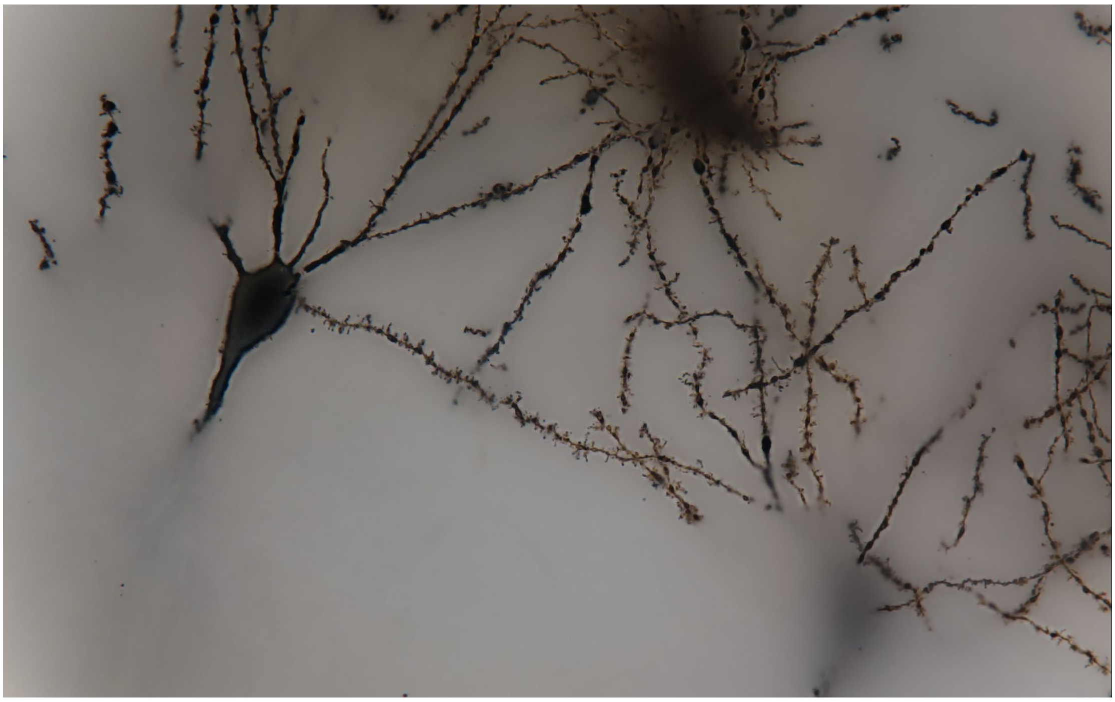

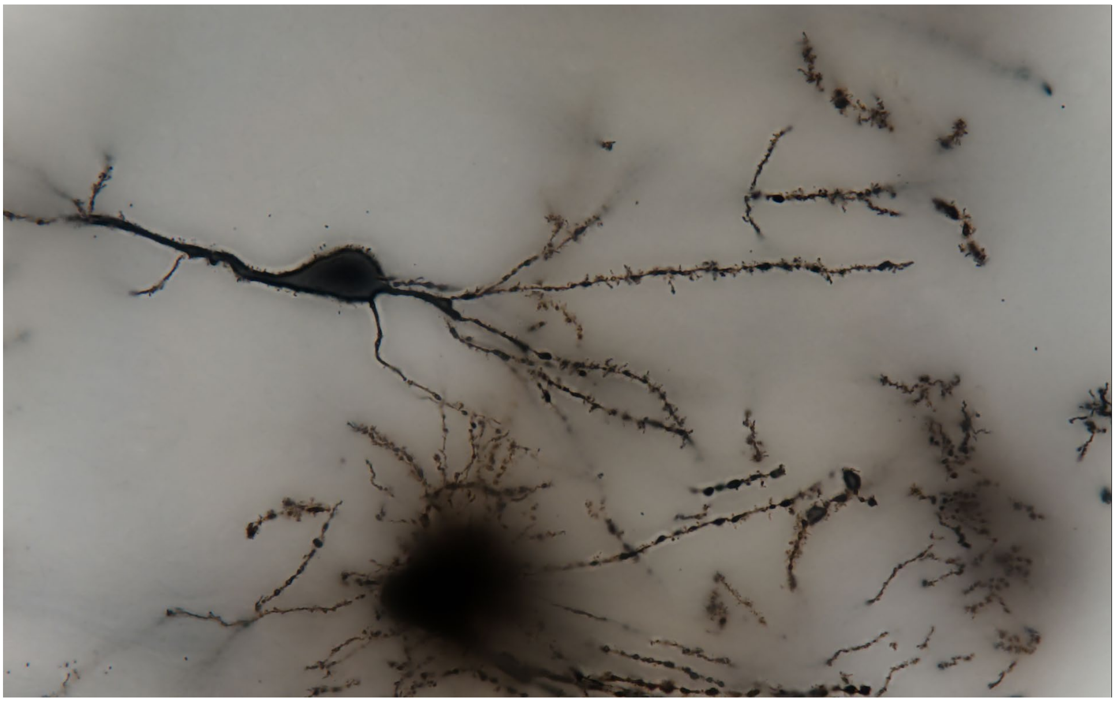

original pictures for basal spines of cKO-Aβ group in Figure 4G and H

.

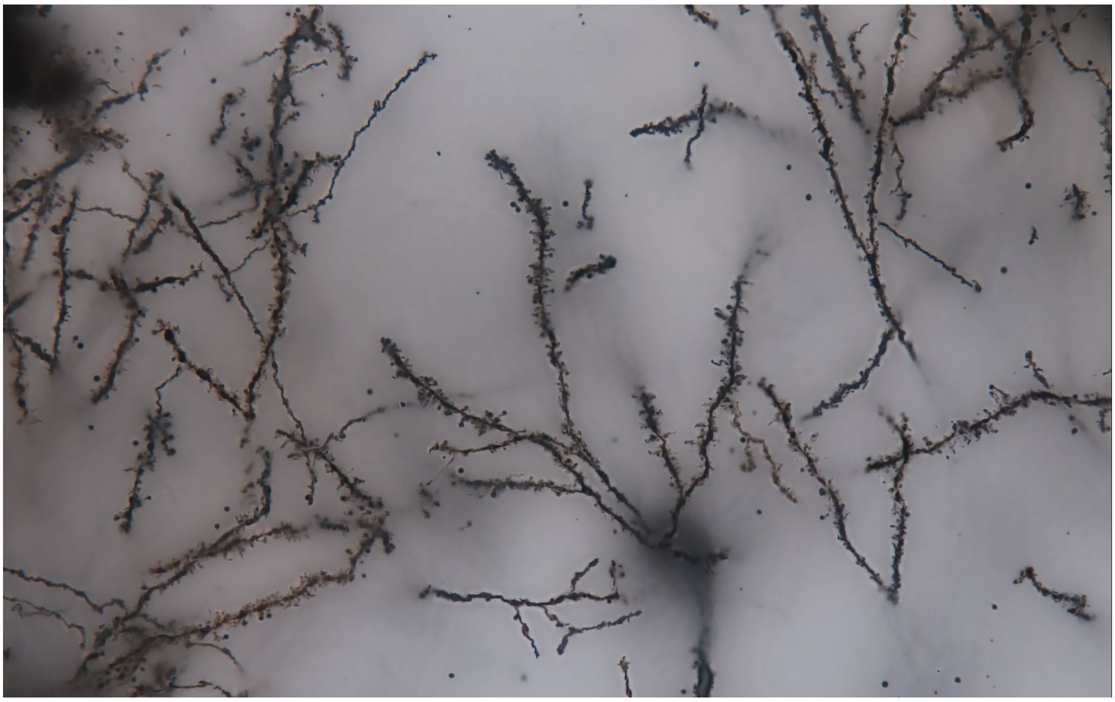

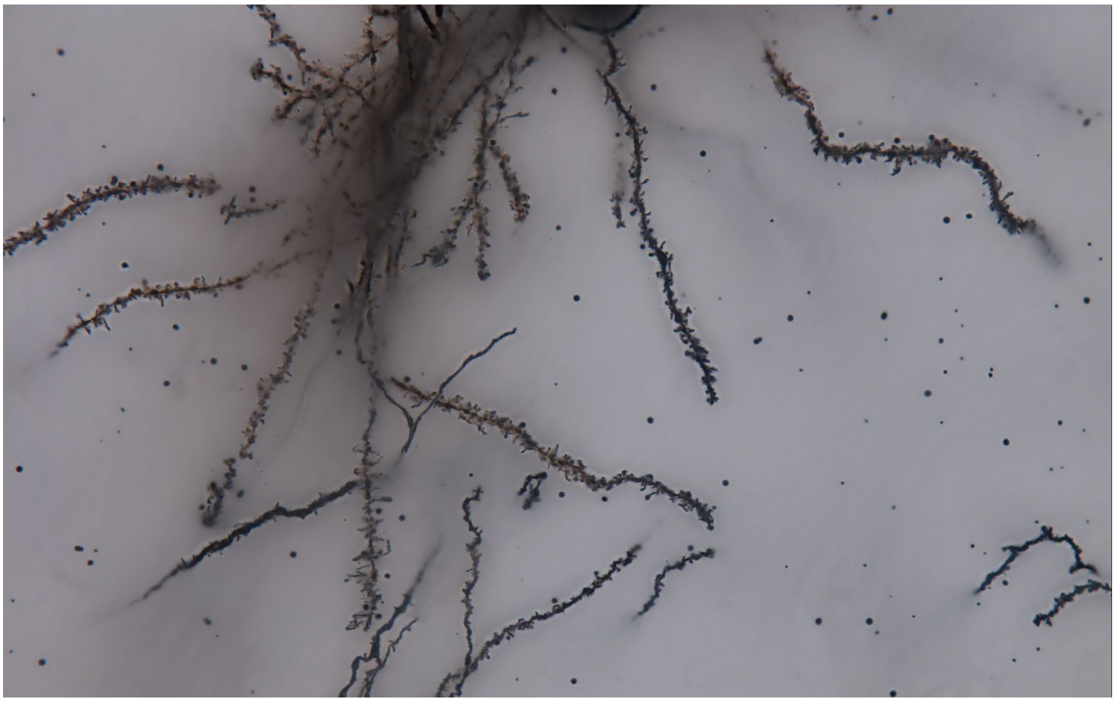

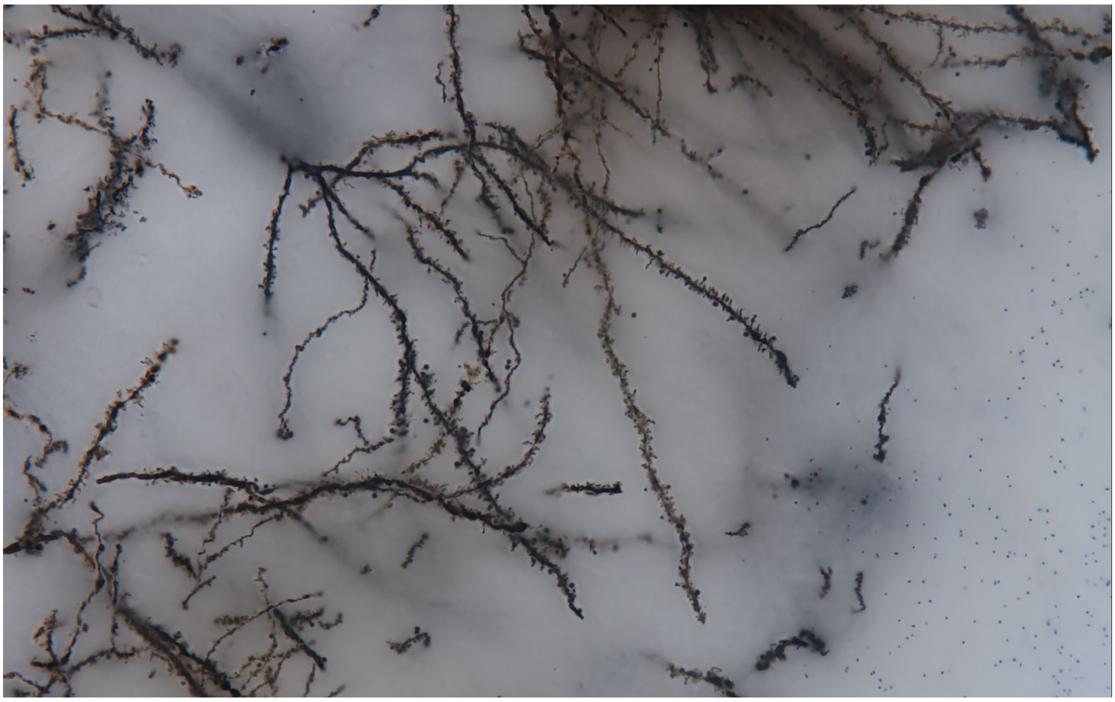

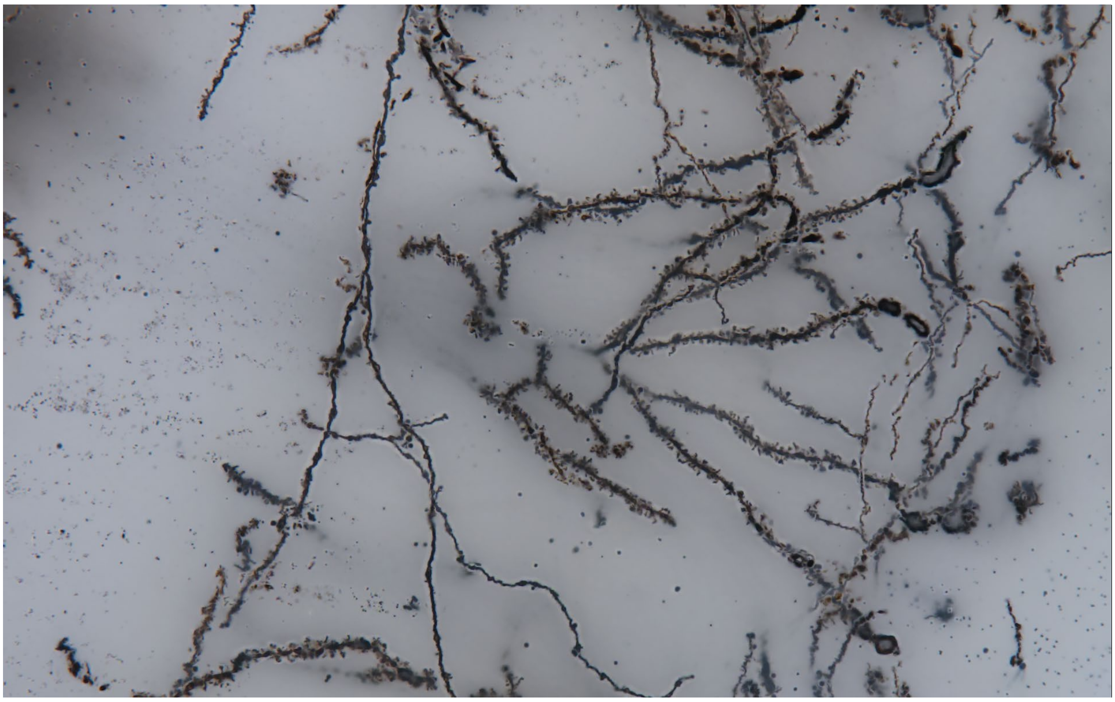

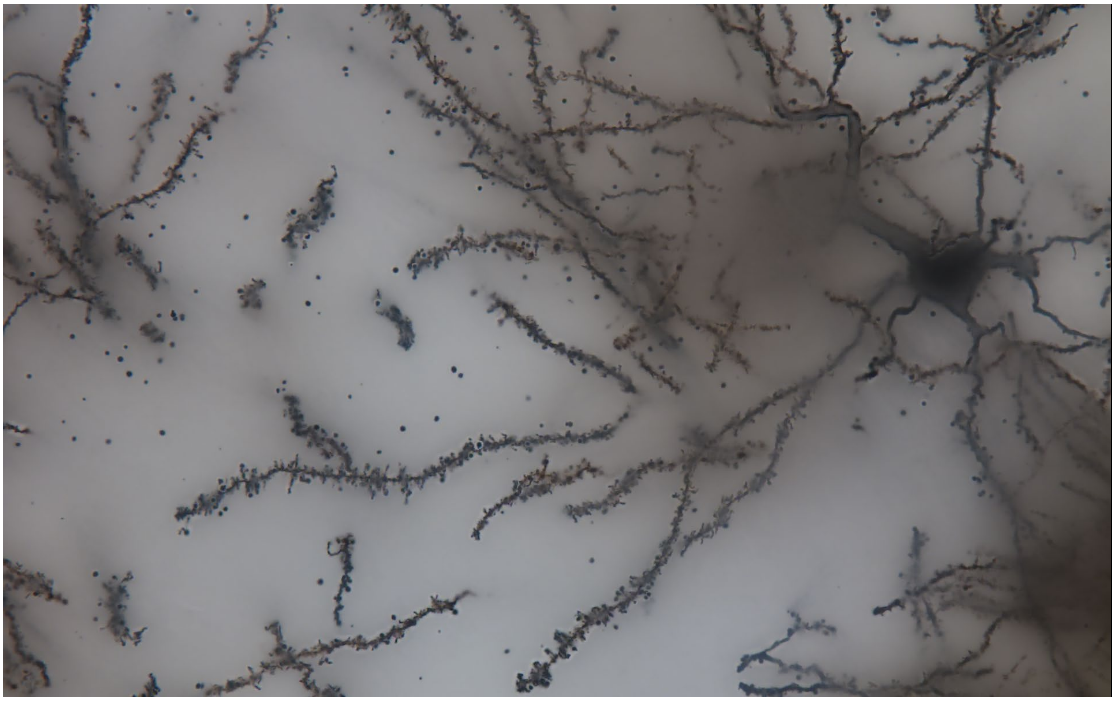

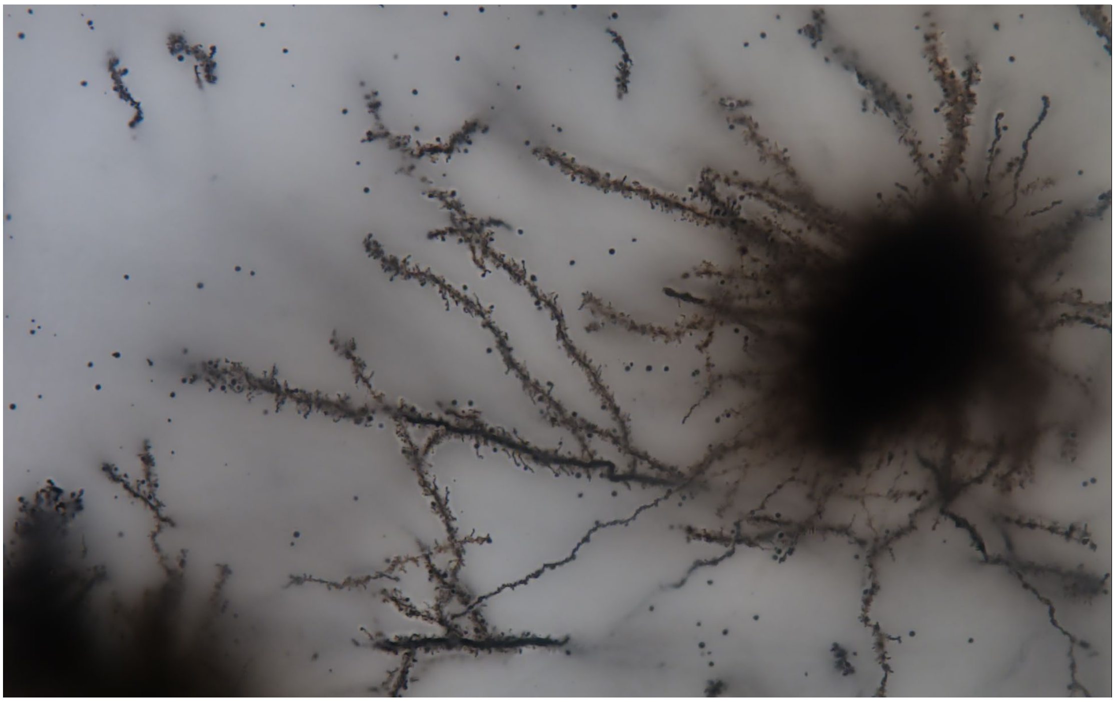

Supplement: Supplementary file 2 — Data S2. [file CNS-30-e14555-s003.pdf]
