# Supplementary material for: Conditional knockout of AIM2 in microglia ameliorates synaptic plasticity and spatial memory deficits in a mouse model of Alzheimer's disease
Source: CNS Neurosci Ther. 2023 Dec 17;30(6):e14555. doi: 10.1111/cns.14555 (PMC11163192; doi:10.1111/cns.14555)

Full unedited gel/blot for Figure 1A

PSD-95（95kDa）

MAP-2（200kDa）

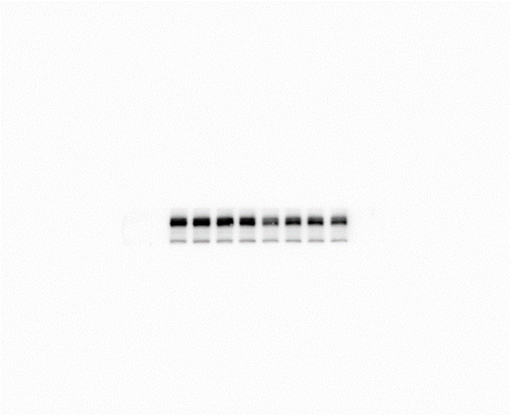

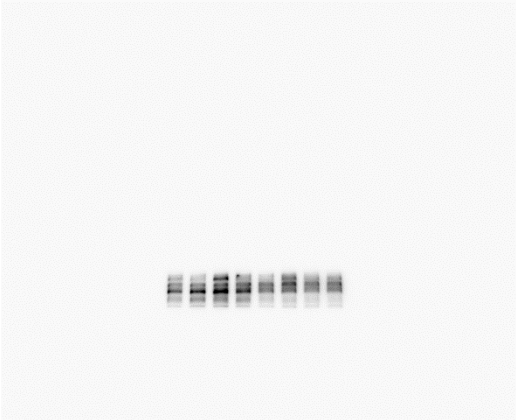

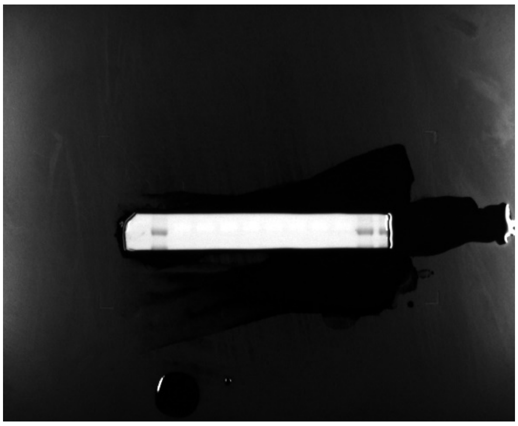

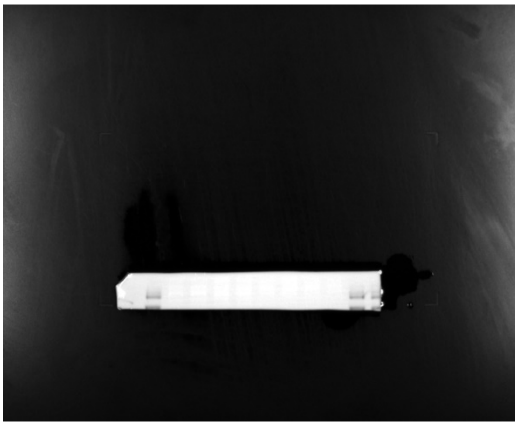

β-actin（42kDa）

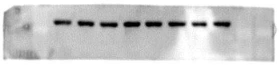

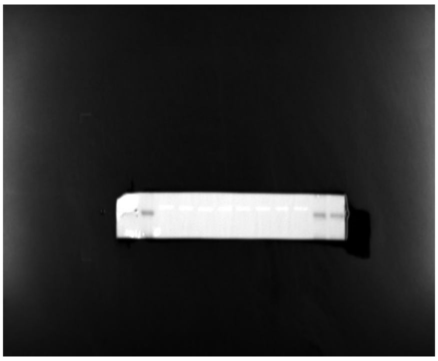

Full unedited gel/blot for Figure 1K

AIM2（39kDa）

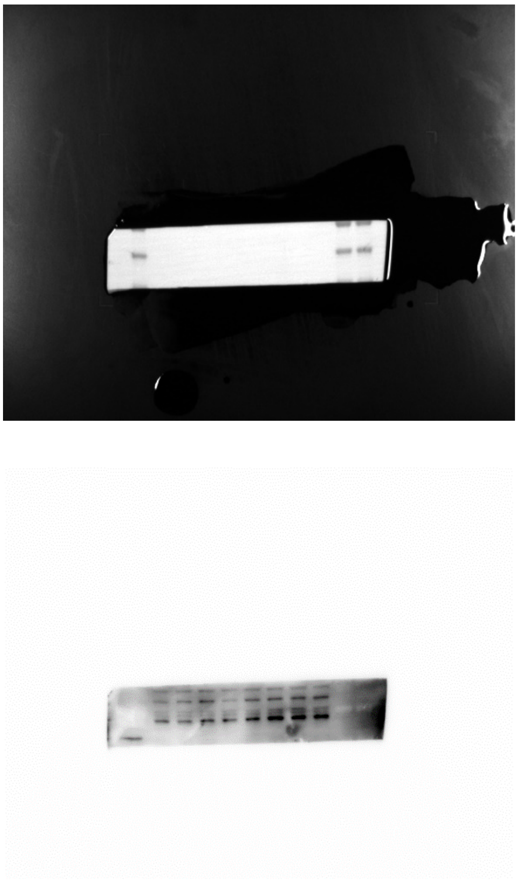

β-actin（42kDa）

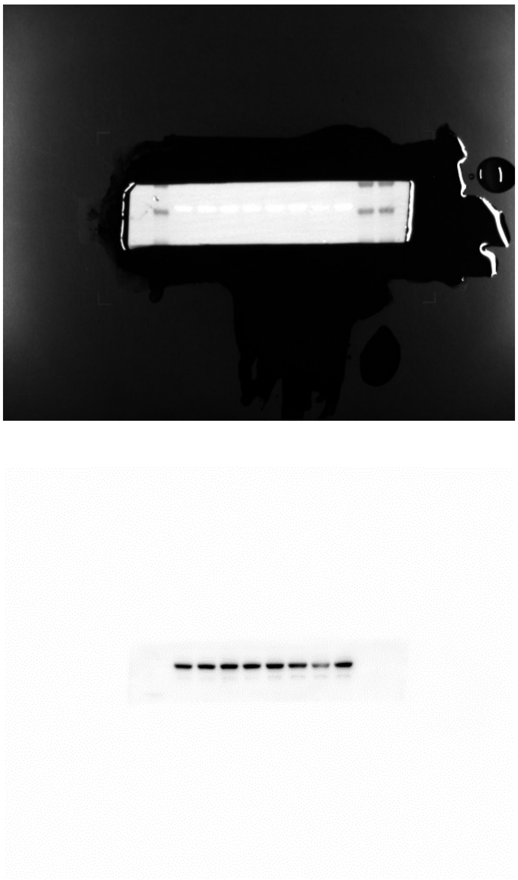

Full unedited gel/blot for Figure 2J

PSD-95（95kDa）

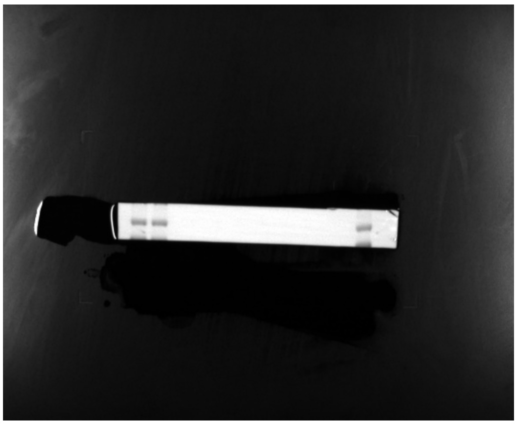

MAP-2（200kDa）

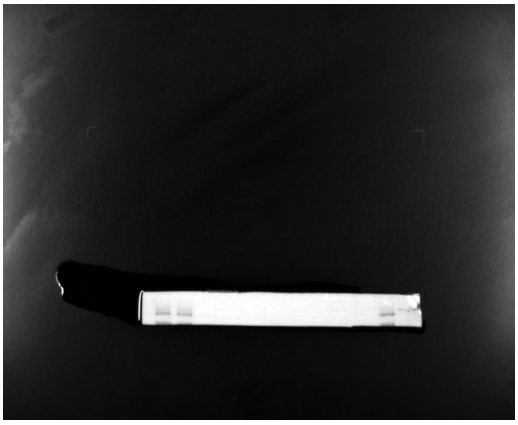

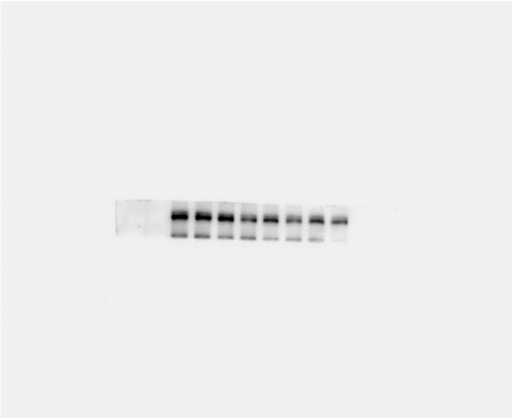

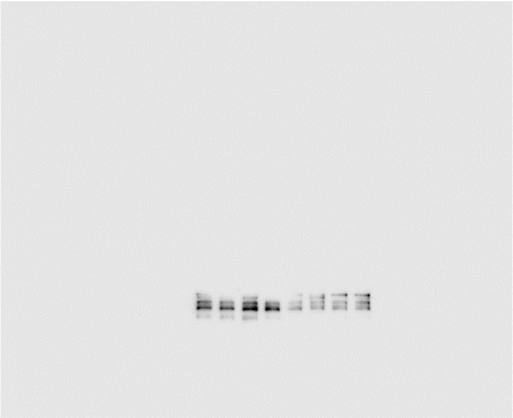

β-actin（42kDa）

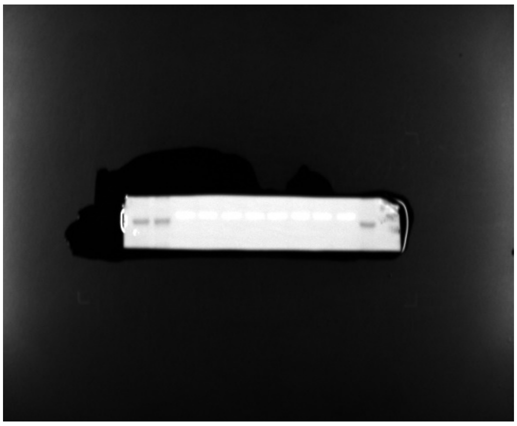

Full unedited gel/blot for Figure 4A

PSD-95（95kDa）

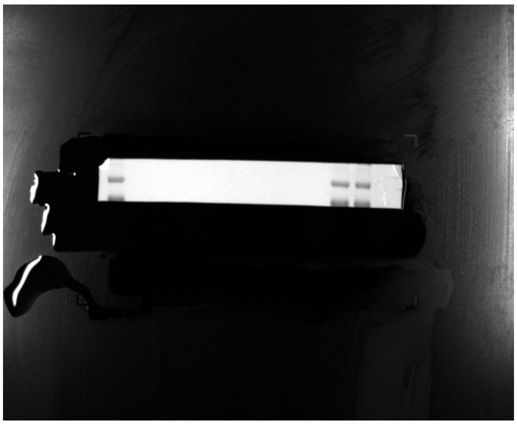

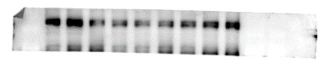

MAP-2（200kDa）

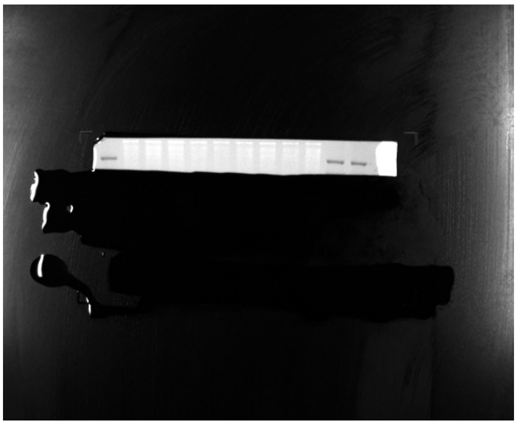

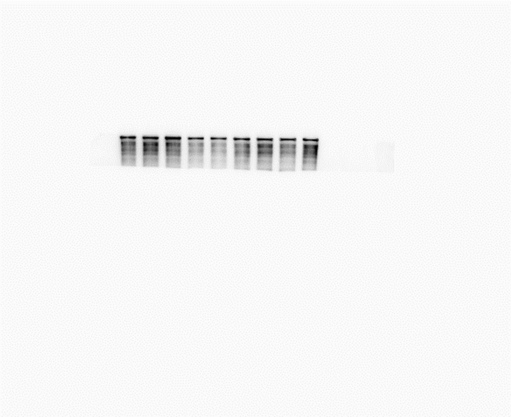

β-actin（42kDa）

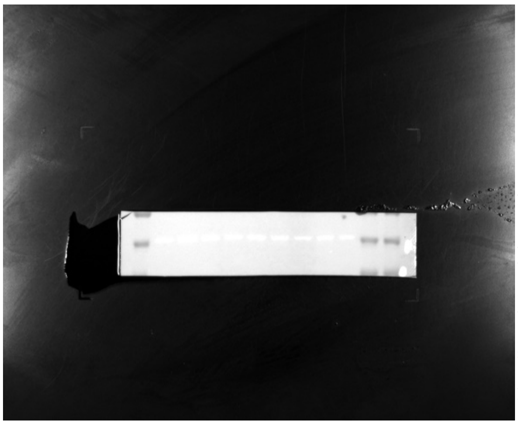

Full unedited gel/blot for Figure S2C

AIM2（39kDa）

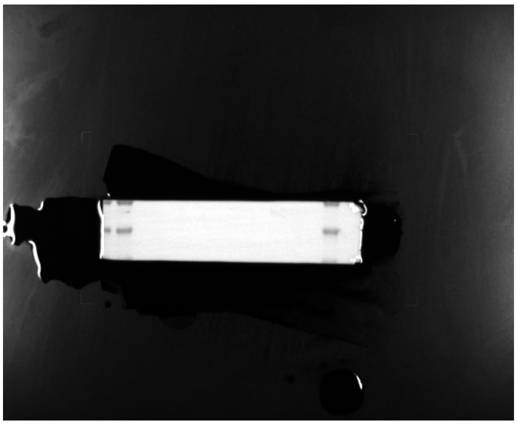

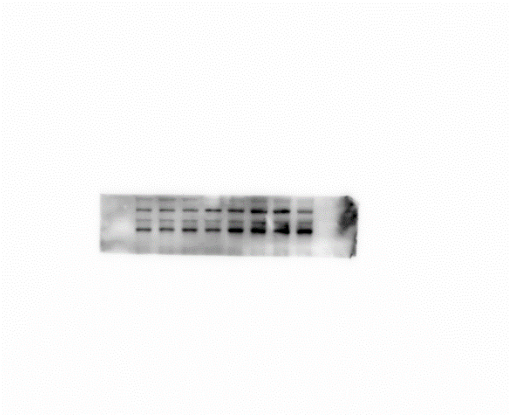

β-actin（42kDa）

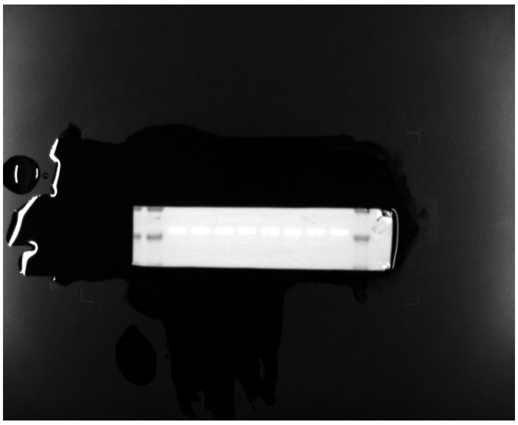

Full unedited gel/blot for Figure6

PSD-95（95kDa）

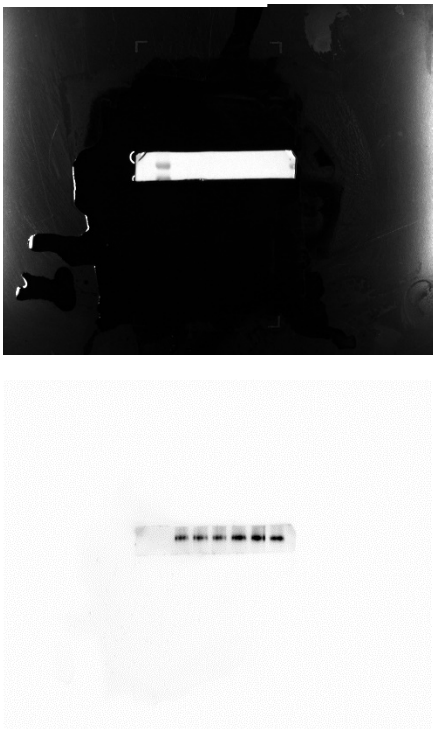

MAP-2（200kDa）

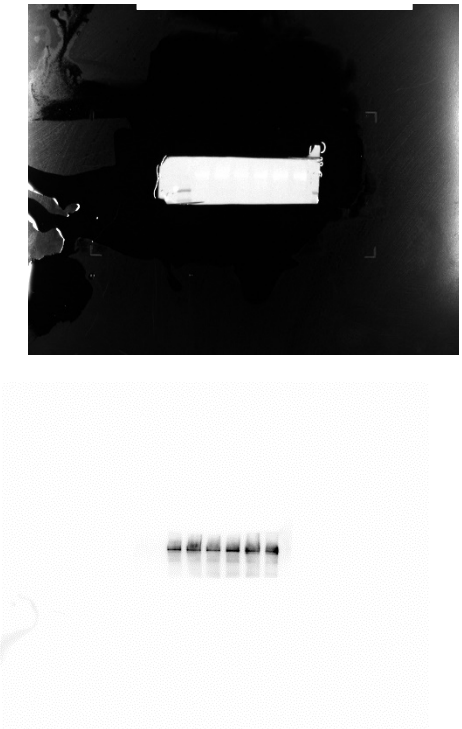

β-actin（42kDa）

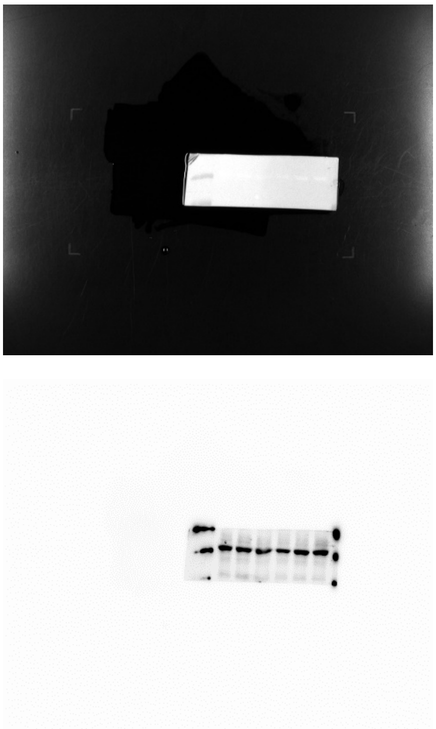

Supplement: Supplementary file 3 — Data S3. [file CNS-30-e14555-s002.pdf]
